# Increasing incidence rates of sexually transmitted infections from 2010 to 2019: an analysis of temporal trends by geographical regions and age groups from the 2019 Global Burden of Disease Study

**DOI:** 10.1186/s12879-022-07544-7

**Published:** 2022-06-26

**Authors:** Min Du, Wenxin Yan, Wenzhan Jing, Chenyuan Qin, Qiao Liu, Min Liu, Jue Liu

**Affiliations:** 1 Department of Epidemiology and Biostatistics, School of Public Health, Peking University, Beijing, China; 2 Institute for Global Health and Development, Peking University, Beijing, 100871 China

**Keywords:** Syphilis, Chlamydia, Gonorrhea, Trichomoniasis, Genital herpes

## Abstract

**Background:**

World Health Organization announced its goal of ending sexually transmitted infection (STI) epidemics by 2030. To provide a reference for tailored prevention strategies, we analyzed trends and differences in STIs by geographical regions and age groups from 1990 to 2019.

**Methods:**

Annual number of new infections and age-standardized incidence rates (ASRs) of syphilis, chlamydia, gonorrhea, trichomoniasis, and genital herpes were recorded from the 2019 Global Burden of Disease study. We quantified the temporal trends of STIs by calculating changes in new infections and estimated annual percentage changes (EAPCs) of ASR.

**Results:**

The ASRs of syphilis, chlamydia, trichomoniasis, and genital herpes increased by 1.70% (95% confidence interval [CI], 1.62–1.78%), 0.29% (95% CI 0.04–0.54%), 0.27% (95% CI 0.03–0.52%), and 0.40% (95% CI 0.36–0.44%) per year from 2010 to 2019 worldwide, respectively, while that of gonorrhea did not. The American regions had the greatest increase in ASR for syphilis (tropical Latin America: EAPC, 5.72; 95% CI 5.11–6.33), chlamydia (high-income North America: EAPC, 1.23; 95% CI 0.73–1.73), and gonorrhea (high-income North America: EAPC, 0.77; 95% CI 0.12–1.41). Additionally, southern sub-Saharan Africa and East Asia had the greatest increase in ASR for trichomoniasis (EAPC, 0.88; 95% CI 0.57–1.20) and genital herpes (EAPC, 1.44; 95% CI 0.83–2.06), respectively. In the most recent years, the population with the greatest incidence of syphilis tended to be younger globally (25–29 years in 2010 vs. 20–24 years in 2019) but older in North Africa and Middle East (20–24 year vs. 25–29 years); with chlamydia tended to be older in southern sub-Saharan Africa (25–29 years vs. 30–34 years) but younger in Australasia (40–44 years vs. 25–29 years); with genital herpes tended to be older in high-income North America (20–24 years vs. 25–29 years) and South Asia (25–29 years vs. 30–34 years).

**Conclusions:**

Syphilis, chlamydia, trichomoniasis, and genital herpes showed a trend of increasing ASR from 2010 to 2019. The differences in trends by geographical regions and age groups point to the need for more targeted prevention strategies in key regions and populations.

**Supplementary Information:**

The online version contains supplementary material available at 10.1186/s12879-022-07544-7.

## Background

Sexually transmitted infections (STIs), including syphilis, chlamydia, gonorrhea, trichomoniasis, and genital herpes, are globally a major public health problem. *Chlamydia trachomatis* and *Neisseria gonorrhoeae (N. gonorrhoeae)* infections have a detrimental influence, including disseminated infection, tubal-factor infertility, pelvic inflammation, and ectopic pregnancy [1–4]. The risk of acquiring or transmitting human immunodeficiency virus infection may greatly increase due to the presence of STIs [3]. Generally, STIs severely impair the quality of life, with physical, psychological, and social consequences [3]. Although chlamydia, gonorrhea, and syphilis as bacterial STIs and trichomoniasis as a parasitic STI can be cured using existing drug regimens, drug resistance may threaten the possibility of them being cured [1]. For example, uncomplicated gonococcal infections may become uncurable when strains with multi-drug and extensive drug resistance become dominant [2]. In addition, antiviral drugs only mediate, not cure, genital herpes infections [1].

Therefore, preventive approaches have become preferable for inhibiting the acquisition of STIs [1]. Developing vaccines is a priority, but vaccinations are not yet available [2]. In 2016, the World Health Organization (WHO)’s *Global Health Sector Strategy on Sexually Transmitted Infections* pointed out that *N. gonorrhoeae* infections require immediate action for control due to the rising risk of untreatable gonorrhea and co-infection with other STIs [3]. This report also recognized the increasing rate of chlamydial infection in adolescents [3]. The WHO highlighted a 2030 goal of ending STI epidemics, with key targets including a 90% reduction in *Treponema pallidum* (*T. pallidum*) incidence and *N. gonorrhoeae* incidence globally (from the 2018 global baseline) [5]. Given that the regional and age differences in STI burden may vary, the WHO proposed a priority focus on obtaining better data on STI burdens by age group to assess progress toward STI control [5]. Moreover, the immense burden of STIs may be distributed disproportionately at the geographical and age levels due to income, health services, lack of treatment, and stigmatization [1]. However, a description of the latest trend characteristics of STIs at the geographical and age levels is lacking.

To identify priority areas for action, we described the current epidemic status and features of 5 STIs, including syphilis, chlamydia, gonorrhea, trichomoniasis, and genital herpes, and further analyzed the temporal trends from 1990 to 2019 by geographical (global, regional, and national level) regions and age groups using data from the 2019 Global Burden of Disease (GBD) study [6]. This study sought to provide a comprehensive perspective reference on targeted control strategies by compiling an extensive and complementary report on the landscape and long-term trends in global, regional, and age differences in STI incidence.

## Methods

### Data source

The Institute for Health Metrics and Evaluation at the University of Washington in Seattle, WA, USA, coordinated the GBD study [6]. To quantify the comparative magnitude of health losses because of diseases by sex, age, region, and country over time, the GBD study made a systematic and scientific effort [6]. We obtained data on the annual number of incident cases and incidence rates of five STIs, including syphilis, chlamydia, gonorrhea, trichomonas, and genital herpes (International Classification of Diseases codes were shown in Additional file 1: Table S1) from 1990 to 2019 based on different sex, age, region, and country groups from the Global Health Data Exchange website established by the GBD group [6, 7].

The GBD study used a series of models to estimate data to make it possible to compare STIs between different countries. The specific methodological approaches to estimate STI incidence were described elsewhere [8]. In brief, incidence data from National Ministry of Health websites, published studies, antenatal clinic surveillance reports, the GBD collaborator network, and case-notification data from locations with mandatory centralized reporting were reviewed to estimate the incidence of STIs. The GBD team estimated each STI’s incidence with a Bayesian meta-regression model by location, year, age, and sex [8].

### Regions and demographics

There were 204 countries or territories that reported STIs from 1990 to 2019. Based on the Sociodemographic Index (SDI), 204 countries or territories were classified into 5 regions, including low, low-middle, middle, high-middle, and high SDI regions, respectively. GBD researchers developed SDI as a composite indicator of total fertility rate among those aged < 25 years, education level for those aged ≥ 15 years, and lag distributed income per capita [9]. Additionally, based on epidemiological homogeneity and geographical contiguity, 204 countries or territories were also separated into 21 regions (the high-income Asia Pacific, Central Asia, and others) in the GBD study [10]. We designed age groups with intervals of 5 years from < 5 years old to ≥ 95 years old for this study.

### Data analysis

We used the absolute number with 95% uncertainty intervals (UIs) and age-standardized incidence rates (ASRs) with 95% UIs of new STIs to show the epidemic status of STIs. The absolute number of incident cases reflected the real occurrence of STIs in each country or territory. ASRs were calculated by applying the age-specific rates for each location, sex, and year to a GBD World Standard Population group [8, 11]. After considering that standardization is important for comparing populations with varying age structures or over time, we extracted ASRs [8, 11].

The relative changes from 1990 to 2019 were calculated by using the formula $\frac{{{\text{incident}}\;{\text{cases}}_{2019} - {\text{incident}}\;{\text{cases}}_{1990} }}{{{\text{incident}}\;{\text{cases}}_{1990} }} \times 100\% .$ Estimated annual percentage changes (EAPCs) as a summary measure of the ASR trend over a specified time interval were widely used [11–13]. The natural logarithm of ASR was fitted to a regression line, i.e., y = α + βx + ε, where y = ln (ASR) and x = calendar year [11–13]. Then, the EAPC (95% CI) was calculated as 100 × (e^β^ − 1) [11–13]. When the EAPC estimation and its 95% CI were both > 0 (or both < 0), the trend of ASR was increasing (or decreasing) [11]. Because the global incidence rate of almost all 5 STIs had highest and lowest peaks of ASR in 2000 and 2010, respectively, and we wanted to represent the details of the past decade’s changes in ASR, we chose 2000 and 2010 as the cutoff time points, then calculated EAPC estimations of 3 time intervals (1990 to 2000, 2000 to 2010, and 2010 to 2019) (Additional file 1: Fig. S1). All statistics were performed using R version 4.0 (R Foundation for Statistical Computing, Vienna, Austria).

## Results

### Global and national trends in STI incidence

The numbers of incident cases and positive growth for the 5 STIs from 1990 to 2019 globally were shown in Additional file 1: Table S2. Specifically, the top two countries in terms of incident cases in 2019 were India and China (Additional file 1: Fig. S2 and Table S3). Of 204 countries or territories, there were 163 (79.90%), 168 (82.35%), 149 (73.04%), 189 (92.65%), and 161 (78.92%) countries or territories that experienced positive growth in the number of syphilis, chlamydial, gonococcal, trichomoniasis, and genital herpes incident cases, respectively. Qatar had the greatest growth in incident case numbers among the five STIs from 1990 to 2019 (Additional file 1: Fig. S2 and Table S3).

The global ASRs of syphilis, chlamydia, gonorrhea, trichomoniasis, and genital herpes were 178.48, 2883.87, 1124.39, 4327.29, and 1021.68 per 1000,000 people in 2019 (Tables 1, 2, 3, 4 and 5). Globally, there was initially an increasing trend in the ASR of syphilis from 1990 to 2000. Subsequently, it decreased from 2000 to 2010; however, in the past decade, it increased on average by 1.25% (95% CI 1.06–1.45%) per year from 159.41 per 100,000 people in 2010 to 178.48 per 100,000 people in 2019 (Table 1, Additional file 1: Fig. S1, and Table S4). The trend in the ASR of chlamydia (2010 to 2019: EAPC, 0.40%; 95% CI 0.36–0.44%) from 1990 to 2019 was similar to that of syphilis (Table 2, Additional file 1: Fig. S1, and Table S4). The ASR of trichomoniasis increased from 2000 to 2010; subsequently, it continued to increase on average by 0.27% (95% CI 0.03–0.52%) per year from 4232.24 per 100,000 people in 2010 to 4327.29 per 100,000 people in 2019 worldwide (Table 3 and Additional file 1: Fig. S1). Although there was a downward trend in the ASR of gonococcal infections from 2010 to 2019, an upward trend was seen from 2017 to 2019 (Table 4, Additional file 1: Fig. S1 and Table S4).

**Table 1:** The age-standardized incidence rates (ASRs, per 100,000 population) of syphilis in 1990, 2010 and 2019, and their temporal trends

Characteristics	Age-standardized incidence rate (ASR, per 100,000)			Estimated annual percentage change (EAPC)	
	2000 (95% UI)	2010 (95% UI)	2019 (95% UI)	EAPC (95% CI) 2000–2010	EAPC (95% CI) 2010–2019
Global	166.27 (125.58, 213.78)	159.41 (121.81, 205.63)	178.48 (134.94, 232.34)	− 0.46 (− 0.59, − 0.33)	1.25 (1.06, 1.45)
Sex					
Female	123.28 (93.98, 157.74)	104.90 (81.64, 132.72)	124.98 (96.68, 160.25)	− 1.69 (− 1.99, − 1.39)	1.85 (1.61, 2.10)
Male	208.66 (157.31, 272.68)	213.38 (161.03, 279.86)	231.31 (171.88, 305.30)	0.21 (0.16, 0.27)	0.94 (0.75, 1.14)
SDI region					
Low	408.24 (317.03, 512.29)	373.06 (293.80, 466.79)	372.53 (287.40, 475.57)	− 0.92 (− 1.00, − 0.85)	− 0.06 (− 0.24, 0.12)
Low-middle	228.72 (171.63, 295.61)	203.86 (153.43, 264.27)	214.66 (160.77, 280.80)	− 1.19 (− 1.54, − 0.84)	0.61 (0.39, 0.84)
Middle	124.45 (93.46, 162.90)	116.52 (87.37, 152.69)	130.13 (96.65, 171.91)	− 0.68 (− 0.75, − 0.62)	1.26 (1.15, 1.36)
High-middle	85.37 (64.10, 112.78)	81.58 (62.10, 105.68)	90.96 (68.81, 118.20)	− 0.49 (− 0.74, − 0.24)	1.11 (0.81, 1.40)
High	72.67 (54.73, 97.16)	73.13 (55.00, 97.19)	74.05 (55.61, 98.92)	0.04 (− 0.24, 0.32)	0.15 (0.12, 0.17)
GBD region					
Andean Latin America	231.87 (181.22, 298.52)	212.91 (167.45, 267.34)	207.84 (158.51, 270.68)	− 0.88 (− 0.98, − 0.77)	− 0.26 (− 0.41, − 0.10)
Australasia	65.15 (48.70, 86.86)	63.91 (47.51, 84.28)	64.07 (47.75, 85.84)	− 0.20 (− 0.26, − 0.13)	0.04 (0.01, 0.08)
Caribbean	175.76 (134.33, 227.29)	185.51 (152.20, 230.58)	191.99 (156.21, 235.61)	0.53 (0.17, 0.88)	0.31 (0.15, 0.47)
Central Asia	55.81 (42.53, 73.31)	51.99 (40.35, 67.92)	52.10 (40.26, 67.34)	− 0.75 (− 0.79, − 0.71)	− 0.02 (− 0.07, 0.04)
Central Europe	42.88 (32.62, 56.88)	42.96 (32.80, 56.18)	43.28 (32.96, 56.54)	0.02 (− 0.07, 0.10)	0.12 (0.06, 0.18)
Central Latin America	116.20 (87.60, 152.30)	111.23 (85.79, 142.84)	111.77 (86.08, 145.02)	− 0.48 (− 0.85, − 0.11)	− 0.03 (− 0.13, 0.07)
Central sub-Saharan Africa	1159.65 (878.24, 1470.11)	1107.12 (887.02, 1363.33)	1048.40 (803.52, 1344.82)	− 0.48 (− 0.79, − 0.16)	− 0.69 (− 0.89, − 0.48)
East Asia	87.51 (65.45, 116.19)	84.94 (63.67, 111.98)	93.43 (69.27, 123.92)	− 0.31 (− 0.75, 0.13)	0.82 (0.39, 1.26)
Eastern Europe	53.09 (40.96, 68.94)	47.19 (36.45, 61.64)	47.97 (37.05, 62.56)	− 1.27 (− 1.39, − 1.14)	0.21 (0.12, 0.29)
Eastern sub-Saharan Africa	581.30 (465.28, 718.82)	468.94 (371.65, 583.69)	492.63 (386.33, 619.27)	− 2.19 (− 2.40, − 1.98)	0.52 (0.29, 0.75)
High-income Asia Pacific	75.79 (57.24, 101.33)	79.38 (59.37, 106.03)	81.36 (60.92, 109.14)	0.48 (0.40, 0.56)	0.33 (0.24, 0.41)
High-income North America	73.46 (55.70, 97.50)	71.22 (54.17, 93.80)	71.76 (54.06, 95.06)	− 0.38 (− 1.31, 0.55)	0.11 (0.08, 0.14)
North Africa and Middle East	81.84 (61.87, 106.20)	83.58 (63.19, 109.66)	84.20 (62.65, 111.70)	0.21 (− 0.06, 0.47)	0.22 (− 0.07, 0.51)
Oceania	483.40 (365.20, 613.56)	415.00 (310.55, 544.19)	415.80 (301.62, 551.31)	− 1.60 (− 2.22, − 0.98)	− 0.11 (− 0.28, 0.07)
South Asia	228.99 (168.89, 302.01)	190.05 (140.16, 251.93)	190.99 (140.95, 254.13)	− 1.88 (− 2.66, − 1.08)	0.04 (− 0.32, 0.41)
Southeast Asia	103.49 (77.69, 136.06)	101.44 (75.73, 133.27)	99.61 (73.45, 131.74)	− 0.20 (− 0.30, − 0.11)	− 0.14 (− 0.29, 0.02)
Southern Latin America	116.11 (90.76, 146.47)	127.42 (105.05, 152.95)	131.89 (106.80, 163.69)	0.99 (0.22, 1.76)	0.08 (− 0.28, 0.44)
Southern sub-Saharan Africa	628.79 (491.31, 792.21)	545.88 (421.45, 698.25)	665.35 (496.08, 872.21)	− 1.37 (− 1.72, − 1.01)	2.50 (2.00, 2.99)
Tropical Latin America	123.86 (91.26, 166.52)	86.45 (68.59, 107.11)	139.68 (109.18, 174.26)	− 3.92 (− 6.76, − 0.99)	5.72 (5.11, 6.33)
Western Europe	67.68 (50.38, 90.60)	67.39 (50.28, 90.33)	67.63 (50.12, 90.19)	− 0.05 (− 0.06, − 0.04)	0.07 (0.04, 0.11)
Western sub-Saharan Africa	440.34 (336.85, 558.49)	427.84 (322.78, 551.62)	427.97 (318.21, 556.32)	− 0.30 (− 0.35, − 0.25)	0.08 (− 0.05, 0.21)

*EAPC* estimated annual percentage change, *CIs* confidence intervals, *UIs* uncertainty interval

**Table 2:** The age-standardized incidence rates (ASRs, per 100,000 population) of chlamydial infection in 1990, 2010 and 2019, and their temporal trends

Characteristics	Age-standardized incidence rate (ASR, per 100,000)			Estimated annual percentage change (EAPC)	
	2000 (95% UI)	2010 (95% UI)	2019 (95% UI)	EAPC (95% CI) 2000–2010	EAPC (95% CI) 2010–2019
Global	3083.25 (2319.33, 4006.82)	2767.15 (2088.69, 3618.82)	2883.87 (2161.21, 3762.80)	− 1.13 (− 1.48, − 0.77)	0.29 (0.04, 0.54)
Sex					
Female	2779.49 (2122.28, 3604.37)	2526.31 (1930.64, 3269.70)	2677.33 (2027.51, 3505.38)	− 1.00 (− 1.35, − 0.65)	0.47 (0.18, 0.77)
Male	3381.46 (2515.44, 4448.60)	3005.84 (2239.03, 3952.39)	3088.09 (2286.90, 4039.30)	− 1.22 (− 1.58, − 0.86)	0.14 (− 0.07, 0.35)
SDI region					
Low	2308.12 (1749.61, 3006.30)	2331.70 (1757.81, 3047.03)	2359.48 (1770.16, 3098.25)	0.10 (− 0.02, 0.21)	0.18 (0.06, 0.30)
Low-middle	2624.46 (1979.48, 3412.47)	2438.40 (1836.01, 3172.65)	2524.34 (1894.78, 3281.85)	− 0.78 (− 0.86, − 0.69)	0.29 (0.16, 0.42)
Middle	3770.57 (2837.26, 4896.10)	3375.38 (2557.28, 4396.95)	3477.61 (2601.94, 4553.28)	− 1.16 (− 1.58, − 0.73)	0.17 (− 0.09, 0.43)
High-middle	3540.95 (2659.46, 4617.09)	3001.79 (2256.41, 3935.33)	3262.50 (2430.97, 4273.94)	− 1.69 (− 2.26, − 1.12)	0.60 (0.08, 1.12)
High	1183.75 (894.38, 1549.34)	1161.48 (887.98, 1507.09)	1241.28 (936.52, 1617.46)	− 0.22 (− 0.26, − 0.18)	0.53 (0.29, 0.77)
GBD region					
Andean Latin America	2601.00 (2007.77, 3307.74)	2642.91 (1998.76, 3426.46)	2520.26 (1870.63, 3304.31)	0.17 (0.11, 0.22)	− 0.20 (− 0.63, 0.23)
Australasia	977.68 (753.29, 1252.00)	985.92 (771.49, 1230.37)	1051.10 (784.76, 1364.78)	0.11 (0.06, 0.15)	0.74 (0.48, 1.00)
Caribbean	4379.71 (3271.68, 5704.71)	4369.90 (3279.83, 5704.69)	4340.05 (3228.30, 5660.33)	− 0.02 (− 0.03, − 0.02)	− 0.06 (− 0.08, − 0.05)
Central Asia	5331.46 (3994.38, 6919.09)	5276.06 (3993.52, 6850.22)	5271.28 (3946.14, 6857.60)	− 0.11 (− 0.13, − 0.09)	− 0.01 (− 0.05, 0.03)
Central Europe	3324.84 (2502.79, 4375.42)	3312.67 (2459.05, 4356.74)	3308.59 (2464.68, 4333.19)	− 0.04 (− 0.04, − 0.03)	0.00 (− 0.02, 0.02)
Central Latin America	3855.76 (2909.64, 5035.82)	4059.99 (3077.44, 5321.02)	3850.00 (2903.85, 5041.57)	0.53 (0.35, 0.72)	− 0.36 (− 0.68, − 0.04)
Central sub-Saharan Africa	2127.03 (1594.66, 2789.84)	2135.70 (1590.05, 2831.89)	2111.27 (1568.66, 2799.00)	0.04 (− 0.01, 0.09)	− 0.08 (− 0.20, 0.04)
East Asia	4718.45 (3538.74, 6159.79)	3528.61 (2665.50, 4618.89)	4128.96 (3082.92, 5375.67)	− 2.92 (− 4.06, − 1.76)	1.09 (0.05, 2.14)
Eastern Europe	3653.06 (2719.44, 4788.43)	3626.07 (2707.62, 4722.05)	3625.05 (2701.70, 4753.04)	− 0.08 (− 0.09, − 0.07)	0.02 (− 0.01, 0.05)
Eastern sub-Saharan Africa	3262.62 (2483.19, 4208.26)	3247.74 (2450.72, 4253.65)	3223.05 (2422.13, 4236.64)	− 0.05 (− 0.05, − 0.04)	− 0.05 (− 0.16, 0.06)
High-income Asia Pacific	1030.44 (773.81, 1346.38)	1028.94 (790.04, 1323.87)	1025.36 (767.38, 1355.75)	− 0.03 (− 0.10, 0.03)	− 0.10 (− 0.22, 0.02)
High-income North America	867.76 (657.02, 1140.15)	643.55 (489.35, 838.99)	740.09 (554.75, 968.78)	− 3.16 (− 3.71, − 2.61)	1.23 (0.73, 1.73)
North Africa and Middle East	3495.74 (2682.90, 4517.80)	3431.73 (2619.81, 4453.92)	3264.95 (2470.04, 4246.76)	− 0.18 (− 0.39, 0.03)	− 0.51 (− 0.57, − 0.44)
Oceania	3831.27 (2924.20, 4862.43)	3666.20 (2903.32, 4526.87)	3701.31 (2826.22, 4759.19)	− 0.46 (− 0.56, − 0.35)	0.05 (− 0.04, 0.14)
South Asia	1612.88 (1202.99, 2108.48)	1570.86 (1173.82, 2051.51)	1711.93 (1270.93, 2239.34)	− 0.29 (− 0.46, − 0.13)	0.83 (0.65, 1.01)
Southeast Asia	4251.31 (3200.02, 5557.87)	4275.19 (3216.34, 5592.15)	4252.09 (3180.39, 5558.88)	0.06 (0.03, 0.09)	− 0.10 (− 0.17, − 0.02)
Southern Latin America	886.82 (672.61, 1145.16)	902.80 (675.47, 1179.79)	908.41 (686.00, 1182.24)	0.18 (0.06, 0.30)	0.04 (0.02, 0.07)
Southern sub-Saharan Africa	5890.00 (4593.74, 7540.13)	6053.46 (4671.09, 7788.73)	5324.43 (4039.48, 6940.61)	0.32 (− 0.09, 0.73)	− 0.98 (− 1.62, − 0.34)
Tropical Latin America	4311.13 (3250.51, 5635.92)	3908.58 (2956.68, 5120.29)	4102.22 (3075.72, 5393.45)	− 1.03 (− 1.14, − 0.91)	0.68 (0.26, 1.10)
Western Europe	434.50 (335.70, 560.53)	437.91 (341.46, 554.49)	429.93 (327.62, 559.92)	0.08 (− 0.05, 0.22)	− 0.27 (− 0.43, − 0.12)
Western sub-Saharan Africa	2307.63 (1760.38, 2997.46)	2549.90 (1935.86, 3323.90)	2316.40 (1741.46, 3047.29)	1.04 (0.58, 1.50)	− 0.66 (− 1.31, − 0.01)

*EAPC* estimated annual percentage change, *CIs* confidence intervals, *UIs* uncertainty interval

**Table 3:** The age-standardized incidence rates (ASRs, per 100,000 population) of gonococcal infection in 1990, 2010 and 2019, and their temporal trends

Characteristics	Age-standardized incidence rate (ASR, per 100,000)			Estimated annual percentage change (EAPC)		
	2000 (95% UI)	2010 (95% UI)	2019 (95% UI)	EAPC (95% CI) 2000–2010		EAPC (95% CI) 2010–2019
Global	1171.43 (910.47, 1519.18)	1165.77 (902.49, 1511.66)	1124.39 (872.97, 1441.08)	− 0.04 (− 0.24, 0.16)		− 0.51 (− 0.79, − 0.24)
Sex						
Female	828.37 (643.70, 1076.90)	768.22 (588.52, 1016.10)	746.62 (573.55, 970.89)	− 0.76 (− 0.97, − 0.54)		− 0.45 (− 0.61, − 0.29)
Male	1508.73 (1172.48, 1962.99)	1557.15 (1204.37, 2036.79)	1494.21 (1154.10, 1922.58)	0.33 (0.12, 0.53)		− 0.56 (− 0.93, − 0.20)
SDI region						
Low	1166.82 (935.68, 1459.39)	1176.01 (928.73, 1513.26)	1121.20 (876.87, 1461.59)	0.08 (− 0.05, 0.21)		− 0.62 (− 0.96, − 0.28)
Low-middle	1191.93 (924.61, 1539.36)	1239.01 (951.61, 1640.00)	1141.32 (883.23, 1474.10)	0.41 (0.12, 0.70)		− 1.09 (− 1.59, − 0.59)
Middle	1274.29 (981.92, 1686.96)	1257.37 (965.23, 1652.80)	1225.96 (936.47, 1618.37)	− 0.14 (− 0.33, 0.05)		− 0.36 (− 0.57, − 0.14)
High-middle	1263.43 (961.25, 1672.92)	1188.51 (915.23, 1581.35)	1167.61 (896.78, 1523.30)	− 0.62 (− 0.77, − 0.47)		− 0.31 (− 0.46, − 0.16)
High	460.13 (365.01, 577.98)	462.08 (364.66, 590.01)	458.63 (365.83, 581.52)	0.07 (0.03, 0.11)		− 0.27 (− 0.51, − 0.03)
GBD region						
Andean Latin America	245.05 (179.18, 335.93)	258.33 (183.00, 362.86)	254.51 (180.90, 363.56)	0.55 (0.23, 0.87)		− 0.19 (− 0.21, − 0.16)
Australasia	315.24 (240.84, 418.93)	310.52 (235.06, 414.23)	306.23 (230.23, 408.85)	− 0.14 (− 0.26, − 0.02)		− 0.23 (− 0.32, − 0.14)
Caribbean	1263.01 (869.10, 1904.58)	1181.67 (813.27, 1764.55)	1199.47 (829.79, 1791.33)	− 0.70 (− 0.74, − 0.66)		0.11 (− 0.14, 0.35)
Central Asia	2494.48 (1716.54, 3708.69)	2311.97 (1606.38, 3398.98)	2269.24 (1542.52, 3364.37)	− 0.80 (− 0.93, − 0.68)		− 0.30 (− 0.41, − 0.18)
Central Europe	1892.05 (1434.88, 2528.26)	1836.45 (1390.90, 2457.00)	1816.54 (1375.73, 2454.40)	− 0.30 (− 0.35, − 0.26)		− 0.24 (− 0.40, − 0.09)
Central Latin America	791.13 (615.74, 1012.26)	771.55 (601.30, 994.23)	772.79 (604.45, 993.38)	− 0.27 (− 0.29, − 0.25)		0.01 (− 0.07, 0.08)
Central sub-Saharan Africa	1240.68 (892.67, 1744.89)	1228.93 (881.89, 1747.13)	1187.74 (842.19, 1721.81)	− 0.10 (− 0.14, − 0.05)		− 0.45 (− 0.56, − 0.34)
East Asia	1159.74 (822.14, 1624.76)	1076.75 (761.02, 1511.46)	1088.78 (765.14, 1508.29)	− 0.74 (− 0.88, − 0.59)		0.00 (− 0.18, 0.18)
Eastern Europe	2186.94 (1573.90, 3078.48)	2088.52 (1507.23, 2915.64)	2115.43 (1511.49, 2972.55)	− 0.48 (− 0.50, − 0.46)		− 0.02 (− 0.31, 0.28)
Eastern sub-Saharan Africa	1439.49 (1167.23, 1797.00)	1403.81 (1095.47, 1833.70)	1363.62 (1054.26, 1804.90)	− 0.26 (− 0.46, − 0.05)		− 0.39 (− 0.54, − 0.23)
High-income Asia Pacific	672.03 (533.51, 839.84)	677.88 (535.85, 851.64)	671.08 (530.73, 845.19)	0.09 (0.03, 0.15)		− 0.18 (− 0.28, − 0.07)
High-income North America	337.54 (252.09, 454.62)	337.54 (249.57, 451.52)	375.03 (277.70, 501.02)	0.00 (− 0.17, 0.17)		0.77 (0.12, 1.41)
North Africa and Middle East	1225.85 (933.31, 1655.77)	1184.24 (871.00, 1643.77)	1164.03 (853.33, 1642.04)	− 0.35 (− 0.47, − 0.22)		− 0.18 (− 0.22, − 0.15)
Oceania	1750.69 (1202.14, 2571.57)	1881.77 (1281.88, 2824.04)	1693.15 (1139.36, 2590.01)	0.75 (0.47, 1.03)		− 0.44 (− 1.36, 0.48)
South Asia	1174.18 (856.28, 1634.05)	1300.55 (915.31, 1834.15)	1134.27 (814.83, 1598.03)	1.07 (0.63, 1.51)		− 1.77 (− 2.60, − 0.93)
Southeast Asia	1375.22 (1096.70, 1731.81)	1333.75 (1060.26, 1671.92)	1335.19 (1061.49, 1687.72)	− 0.32 (− 0.48, − 0.15)		0.04 (− 0.07, 0.14)
Southern Latin America	436.87 (314.28, 613.75)	431.22 (308.17, 609.46)	430.95 (309.16, 610.91)	− 0.13 (− 0.22, − 0.05)		− 0.06 (− 0.17, 0.05)
Southern sub-Saharan Africa	4515.26 (3453.11, 5939.45)	4175.61 (3174.47, 5507.98)	3869.55 (2945.69, 5086.97)	− 0.79 (− 1.17, − 0.41)		− 0.69 (− 0.99, − 0.39)
Tropical Latin America	1027.67 (707.44, 1473.02)	855.73 (600.57, 1207.29)	912.93 (631.86, 1293.01)	− 1.98 (− 2.19, − 1.76)		0.73 (0.47, 0.99)
Western Europe	134.69 (107.29, 171.12)	128.75 (101.92, 163.61)	130.45 (104.25, 165.78)	− 0.46 (− 0.57, − 0.34)		− 0.01 (− 0.22, 0.19)
Western sub-Saharan Africa	1394.78 (1130.39, 1732.43)	1346.86 (1073.43, 1706.65)	1318.35 (1046.77, 1674.62)	− 0.37 (− 0.38, − 0.35)		− 0.31 (− 0.50, − 0.12)

*EAPC* estimated annual percentage change, *CIs* confidence intervals, *UIs* uncertainty interval

**Table 4:** The age-standardized incidence rates (ASRs, per 100,000 population) of trichomoniasis in 1990, 2010 and 2019, and their temporal trends

Characteristics	Age-standardized incidence rate (ASR, per 100,000)			Estimated annual percentage change (EAPC)	
	2000 (95% UI)	2010 (95% UI)	2019 (95% UI)	EAPC (95% CI) 2000–2010	EAPC (95% CI) 2010–2019
Global	4186.35 (3083.69, 5458.95)	4232.24 (3112.87, 5529.40)	4327.29 (3176.53, 5645.76)	0.11 (0.06, 0.17)	0.27 (0.03, 0.52)
Sex					
Female	3526.77 (2562.30, 4650.06)	3608.56 (2616.58, 4769.16)	3781.34 (2719.77, 5010.59)	0.24 (0.15, 0.33)	0.61 (0.03, 1.20)
Male	4843.00 (3586.19, 6349.38)	4860.24 (3590.68, 6370.79)	4879.67 (3610.00, 6366.99)	0.03 (0.01, 0.06)	0.03 (0.00, 0.05)
SDI region					
Low	5540.55 (4147.65, 7180.19)	5632.89 (4192.13, 7352.95)	5748.06 (4236.67, 7502.65)	0.18 (0.07, 0.28)	0.28 (0.21, 0.36)
Low-middle	3959.13 (2933.69, 5145.98)	3983.53 (2945.85, 5204.16)	4051.78 (2978.09, 5277.78)	0.07 (0.01, 0.12)	0.17 (− 0.05, 0.39)
Middle	4194.99 (3072.36, 5464.94)	4226.63 (3091.59, 5510.28)	4267.97 (3116.15, 5565.46)	0.07 (− 0.02, 0.16)	0.15 (− 0.23, 0.53)
High-middle	3758.28 (2749.51, 4933.69)	3778.10 (2759.45, 4945.88)	3829.67 (2796.45, 4987.86)	0.06 (− 0.03, 0.14)	0.19 (− 0.18, 0.56)
High	3390.04 (2488.12, 4442.00)	3435.48 (2531.95, 4500.49)	3481.39 (2552.86, 4554.16)	0.14 (0.13, 0.14)	0.15 (0.12, 0.17)
GBD region					
Andean Latin America	4409.82 (3281.53, 5701.06)	4416.95 (3282.09, 5783.87)	4490.13 (3298.29, 5860.57)	0.02 (− 0.01, 0.05)	0.27 (0.12, 0.42)
Australasia	2810.75 (2081.79, 3694.06)	2800.73 (2106.70, 3638.95)	2803.25 (2080.30, 3648.48)	− 0.03 (− 0.13, 0.06)	0.00 (− 0.06, 0.07)
Caribbean	5078.91 (3766.07, 6603.44)	5119.07 (3811.77, 6655.74)	5228.27 (3855.21, 6815.48)	0.08 (0.07, 0.10)	0.35 (0.15, 0.55)
Central Asia	3944.22 (2947.10, 5122.80)	3992.17 (2955.47, 5189.20)	3954.09 (2912.62, 5161.83)	0.13 (0.00, 0.25)	− 0.15 (− 0.38, 0.07)
Central Europe	3392.01 (2515.84, 4402.16)	3426.77 (2541.46, 4479.62)	3463.90 (2553.06, 4533.86)	0.11 (0.01, 0.20)	0.08 (− 0.04, 0.19)
Central Latin America	6252.66 (4613.33, 8102.32)	6360.64 (4688.26, 8240.41)	6519.08 (4766.17, 8493.79)	0.18 (0.10, 0.26)	0.40 (0.20, 0.61)
Central sub-Saharan Africa	5399.63 (4023.69, 7068.97)	5496.66 (4097.74, 7186.84)	5500.77 (4075.69, 7157.30)	0.19 (0.15, 0.24)	0.04 (− 0.03, 0.10)
East Asia	4338.44 (3155.02, 5683.44)	4351.29 (3144.73, 5706.38)	4403.83 (3195.76, 5762.80)	0.04 (− 0.13, 0.21)	0.22 (− 0.46, 0.91)
Eastern Europe	3119.98 (2253.81, 4096.27)	3112.86 (2249.59, 4069.94)	3170.49 (2289.66, 4152.30)	− 0.03 (− 0.13, 0.08)	0.18 (0.16, 0.21)
Eastern sub-Saharan Africa	10,094.23 (7664.31, 12,884.63)	9874.58 (7306.38, 12,767.00)	10,014.29 (7336.36, 12,908.25)	− 0.23 (− 0.25, − 0.21)	0.23 (0.14, 0.32)
High-income Asia Pacific	3172.89 (2336.66, 4167.99)	3194.38 (2356.89, 4183.53)	3247.76 (2375.14, 4239.47)	0.07 (− 0.02, 0.17)	0.19 (0.15, 0.22)
High-income North America	4235.13 (3067.24, 5535.79)	4222.63 (3069.95, 5530.79)	4224.42 (3049.86, 5536.13)	− 0.03 (− 0.05, − 0.01)	0.00 (− 0.06, 0.05)
North Africa and Middle East	3887.06 (2933.92, 4956.65)	3749.78 (2798.51, 4820.06)	3633.77 (2699.73, 4675.87)	− 0.38 (− 0.41, − 0.34)	− 0.32 (− 0.41, − 0.22)
Oceania	7012.73 (5245.51, 9124.38)	7438.01 (5725.31, 9515.68)	7046.62 (5258.47, 9082.33)	0.61 (0.34, 0.88)	− 0.53 (− 0.73, − 0.32)
South Asia	3050.51 (2233.86, 3995.18)	2998.82 (2201.06, 3932.54)	2989.08 (2182.21, 3904.48)	− 0.17 (− 0.22, − 0.12)	− 0.13 (− 0.45, 0.19)
Southeast Asia	4424.34 (3261.56, 5793.71)	4465.33 (3286.15, 5855.01)	4455.60 (3257.20, 5836.66)	0.10 (0.01, 0.19)	− 0.09 (− 0.21, 0.02)
Southern Latin America	2922.52 (2188.21, 3790.97)	2945.02 (2179.04, 3856.93)	2963.95 (2200.04, 3863.44)	0.08 (0.06, 0.10)	0.08 (0.06, 0.11)
Southern sub-Saharan Africa	7869.53 (5829.00, 10,111.74)	7377.11 (5434.66, 9545.41)	7838.36 (5733.84, 10,163.46)	− 0.67 (− 0.88, − 0.46)	0.88 (0.57, 1.20)
Tropical Latin America	5719.73 (4146.26, 7461.74)	5722.70 (4207.23, 7461.10)	5889.37 (4255.92, 7701.51)	0.01 (0.00, 0.02)	0.41 (0.15, 0.68)
Western Europe	2386.75 (1779.99, 3115.39)	2414.84 (1787.52, 3153.00)	2404.86 (1790.97, 3136.99)	0.13 (0.11, 0.15)	− 0.05 (− 0.09, − 0.01)
Western sub-Saharan Africa	7524.26 (5616.51, 9763.05)	7585.92 (5625.56, 9895.69)	7656.26 (5652.53, 9981.63)	0.10 (− 0.01, 0.20)	0.16 (0.05, 0.26)

*EAPC* estimated annual percentage change, *CIs* confidence intervals, *UIs* uncertainty interval

**Table 5:** The age-standardized incidence rates (ASRs, per 100,000 population) of genital herpes in 1990, 2010 and 2019, and their temporal trends

Characteristics	Age-standardized incidence rate (ASR, per 100,000)			Estimated annual percentage change (EAPC)	
	2000 (95% UI)	2010 (95% UI)	2019 (95% UI)	EAPC (95% CI) 2000–2010	EAPC (95% CI) 2010–2019
Global	991.27 (852.64, 1142.47)	986.64 (841.22, 1145.20)	1021.68 (869.15, 1191.20)	− 0.06 (− 0.11, − 0.01)	0.40 (0.36, 0.44)
Sex					
Female	1238.59 (1072.00, 1419.65)	1238.26 (1060.33, 1433.82)	1272.13 (1084.26, 1479.17)	− 0.01 (− 0.03, 0.01)	0.31 (0.23, 0.39)
Male	749.35 (640.03, 875.01)	740.92 (628.78, 867.04)	778.33 (657.90, 917.03)	− 0.13 (− 0.21, − 0.05)	0.57 (0.53, 0.62)
SDI region					
Low	1385.60 (1211.14, 1571.60)	1312.87 (1133.33, 1502.86)	1332.24 (1139.14, 1537.39)	− 0.55 (− 0.83, − 0.26)	0.24 (− 0.05, 0.53)
Low-middle	938.71 (807.42, 1087.82)	951.73 (812.74, 1106.29)	951.22 (810.95, 1108.43)	0.13 (0.08, 0.17)	− 0.03 (− 0.11, 0.05)
Middle	951.20 (815.23, 1102.17)	953.66 (809.95, 1112.22)	983.59 (834.60, 1149.82)	0.00 (− 0.13, 0.12)	0.35 (0.27, 0.43)
High-middle	855.83 (730.79, 993.95)	833.25 (709.52, 975.73)	871.68 (739.91, 1022.12)	− 0.28 (− 0.45, − 0.10)	0.53 (0.36, 0.69)
High	902.05 (782.05, 1038.57)	880.25 (751.06, 1028.58)	862.23 (729.35, 1013.89)	− 0.24 (− 0.51, 0.03)	− 0.23 (− 0.36, − 0.09)
GBD region					
Andean Latin America	1808.88 (1617.65, 2034.55)	1749.32 (1503.70, 2018.59)	1741.01 (1491.84, 2008.59)	− 0.35 (− 0.60, − 0.11)	− 0.04 (− 0.06, − 0.03)
Australasia	856.23 (742.03, 974.46)	791.10 (660.24, 951.37)	794.25 (666.11, 945.00)	− 0.84 (− 3.34, 1.72)	0.05 (0.03, 0.07)
Caribbean	1620.51 (1376.55, 1878.36)	1601.68 (1362.27, 1867.08)	1581.10 (1347.15, 1842.73)	− 0.12 (− 0.17, − 0.07)	− 0.12 (− 0.20, − 0.04)
Central Asia	692.41 (582.67, 822.21)	691.45 (582.84, 825.47)	689.14 (579.50, 820.98)	− 0.01 (− 0.05, 0.03)	− 0.06 (− 0.08, − 0.03)
Central Europe	487.70 (412.95, 579.24)	495.02 (415.75, 587.74)	494.18 (415.42, 589.23)	0.16 (0.12, 0.20)	− 0.04 (− 0.09, 0.01)
Central Latin America	1415.31 (1219.73, 1624.93)	1497.17 (1283.99, 1729.06)	1502.79 (1282.43, 1746.05)	0.58 (0.39, 0.76)	0.05 (0.02, 0.08)
Central sub-Saharan Africa	2260.52 (1999.82, 2541.53)	2248.05 (1981.58, 2537.15)	2245.43 (1973.68, 2530.90)	− 0.06 (− 0.09, − 0.03)	0.01 (− 0.02, 0.03)
East Asia	754.28 (635.50, 890.60)	674.75 (568.73, 799.31)	761.71 (640.16, 907.48)	− 1.13 (− 1.69, − 0.57)	1.44 (0.83, 2.06)
Eastern Europe	933.07 (784.87, 1098.77)	935.55 (789.61, 1101.35)	936.47 (787.28, 1100.87)	0.03 (0.03, 0.03)	0.00 (− 0.02, 0.01)
Eastern sub-Saharan Africa	2211.21 (1958.75, 2490.58)	1922.35 (1681.28, 2184.93)	1939.71 (1662.66, 2250.26)	− 1.44 (− 1.90, − 0.98)	0.25 (− 0.30, 0.80)
High-income Asia Pacific	687.72 (578.75, 808.82)	683.45 (579.15, 805.96)	680.41 (571.07, 805.51)	− 0.06 (− 0.12, 0.01)	− 0.06 (− 0.08, − 0.03)
High-income North America	1191.51 (1018.26, 1390.79)	1107.42 (943.33, 1294.02)	1077.77 (906.01, 1267.90)	− 0.72 (− 1.25, − 0.19)	− 0.29 (− 0.35, − 0.22)
North Africa and Middle East	791.01 (678.65, 918.85)	794.76 (676.33, 934.87)	799.14 (675.26, 948.70)	0.04 (− 0.04, 0.13)	0.06 (0.04, 0.09)
Oceania	1514.66 (1369.53, 1676.40)	1427.76 (1207.15, 1672.31)	1428.70 (1205.75, 1669.20)	− 0.64 (− 1.15, − 0.13)	− 0.04 (− 0.09, 0.02)
South Asia	624.83 (526.79, 742.79)	636.08 (536.12, 755.44)	626.23 (527.02, 744.30)	0.18 (0.13, 0.24)	− 0.18 (− 0.24, − 0.13)
Southeast Asia	1108.52 (949.31, 1288.23)	1090.30 (918.74, 1280.13)	1088.42 (915.94, 1276.73)	− 0.17 (− 0.21, − 0.12)	− 0.06 (− 0.10, − 0.01)
Southern Latin America	1240.75 (1132.94, 1356.88)	1216.34 (1035.64, 1422.25)	1216.03 (1031.26, 1419.28)	− 0.20 (− 0.31, − 0.10)	− 0.01 (− 0.04, 0.02)
Southern sub-Saharan Africa	2288.51 (2021.83, 2578.41)	2377.66 (2119.19, 2664.61)	2275.42 (1990.26, 2605.72)	0.40 (0.25, 0.54)	− 0.54 (− 0.78, − 0.29)
Tropical Latin America	1978.72 (1699.57, 2270.38)	2116.18 (1819.55, 2430.14)	1911.52 (1637.13, 2214.89)	0.71 (0.59, 0.84)	− 1.21 (− 1.71, − 0.71)
Western Europe	717.19 (637.90, 807.91)	730.51 (622.92, 856.30)	696.52 (586.28, 821.86)	0.19 (0.14, 0.24)	− 0.54 (− 0.91, − 0.16)
Western sub-Saharan Africa	1689.49 (1478.73, 1919.29)	1645.61 (1415.01, 1897.67)	1639.92 (1404.48, 1895.52)	− 0.28 (− 0.36, − 0.19)	− 0.04 (− 0.06, − 0.01)

*EAPC* estimated annual percentage change, *CIs* confidence intervals, *UIs* uncertainty interval

Furthermore, the top countries or territories with the highest ASRs of syphilis, chlamydia, gonorrhea, trichomoniasis, and genital herpes were the Central African Republic, South Africa, South Africa, United Republic of Tanzania, and Zimbabwe, respectively, in 2019 (Additional file 1: Table S3). There were 109, 51, 15, 81, and 204 countries or territories with a trend of increasing ASRs of syphilis, chlamydia, gonorrhea, trichomoniasis, and genital herpes from 2010 to 2019, respectively (Additional file 1: Table S3 and Fig. S2). The greatest increases in ASRs of syphilis, chlamydia, gonorrhea, trichomoniasis, and genital herpes were noted for Brazil (EAPC, 6.23; 95% CI 5.51–6.92), the Marshall Islands (EAPC, 9.85; 95% CI 8.26–11.48), the United Kingdom (EAPC, 1.93; 95% CI 0.71–3.16), Lebanon (EAPC, 2.29; 95% CI 1.49–3.10), and Ethiopia (EAPC, 3.05; 95% CI 1.60–4.53), respectively (Additional file 1: Table S3 and Fig. S2).

### Differences in STI incidence across 5 SDI regions

**Fig. 1:**
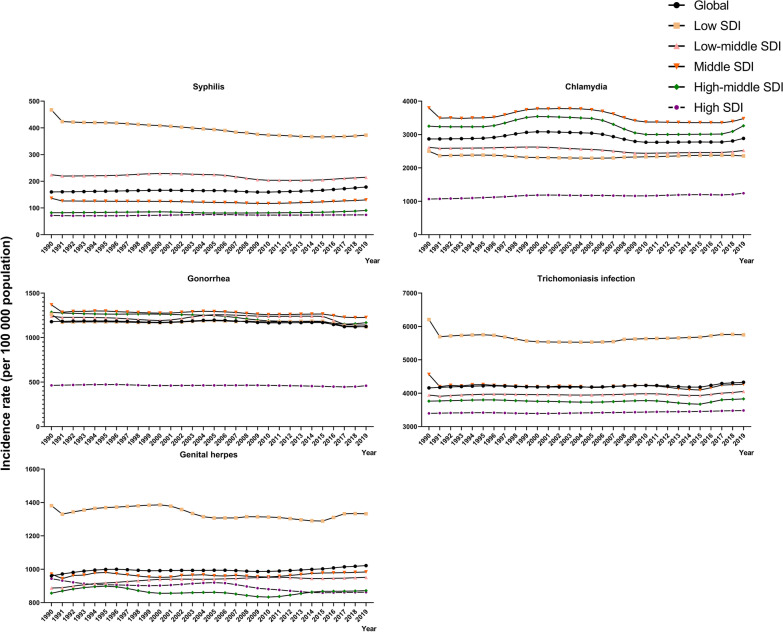
The trends in the incidence rate of sexually transmitted infections by SDI regions from 1990 to 2019

The top 3 regions with growth in the case numbers of 5 STIs included low, low-middle, and middle SDI regions (Additional file 1: Table S1). The ASRs of syphilis, trichomoniasis, and genital herpes were greatest in low SDI regions from 1990 to 2019 (syphilis, 372.53 per 100,000 people; trichomoniasis, 5748.06 per 100,000 people; genital herpes, 1332.24 per 100,000 people in 2019), while the ASRs of chlamydia and gonorrhea were highest in middle SDI regions from 1990 to 2019 (chlamydia, 3477.61 per 100,000 people; gonorrhea, 1225.96 per 100,000 people in 2019) (Tables 1, 2, 3, 4 and 5 and Fig. 1). The SDI regions with a trend of the greatest increase in ASR for syphilis (EAPC, 1.26; 95% CI 1.15–1.36), chlamydia (EAPC, 0.60; 95% CI 0.08–1.12), trichomoniasis (EAPC, 0.28; 95% CI 0.21–0.36), and genital herpes (EAPC, 0.53; 95% CI 0.36–0.69) from 2010 to 2019 were the middle, high-middle, low, and high-middle SDI regions, respectively. The specific values of the trends in 5 STIs in SDI regions are shown in Tables 1, 2, 3, 4 and 5. The age characteristics of ASRs of the 5 STIs in SDI regions are presented in Additional file 1: Fig. S3.

### Differences in STI incidence across 21 GBD regions

The numbers of incident cases of syphilis, chlamydia, gonorrhea, trichomoniasis, and genital herpes were highest in South Asia (3.73 million), East Asia (69.21 million), South Asia (22.79 million), East Asia (81.21 million), and East Asia (12.10 million) in 2019, respectively (Additional file 1: Table S1). Greater growth in numbers of syphilis, chlamydia, gonorrhea, trichomoniasis, and genital herpes incident cases was seen in western sub-Saharan Africa (133.05%), western sub-Saharan Africa (152.59%), central sub-Saharan Africa (139.40%), central sub-Saharan Africa (157.30%), and western sub-Saharan Africa (151.97%) from 1990 to 2019 (Additional file 1: Table S1).

**Fig. 2:**
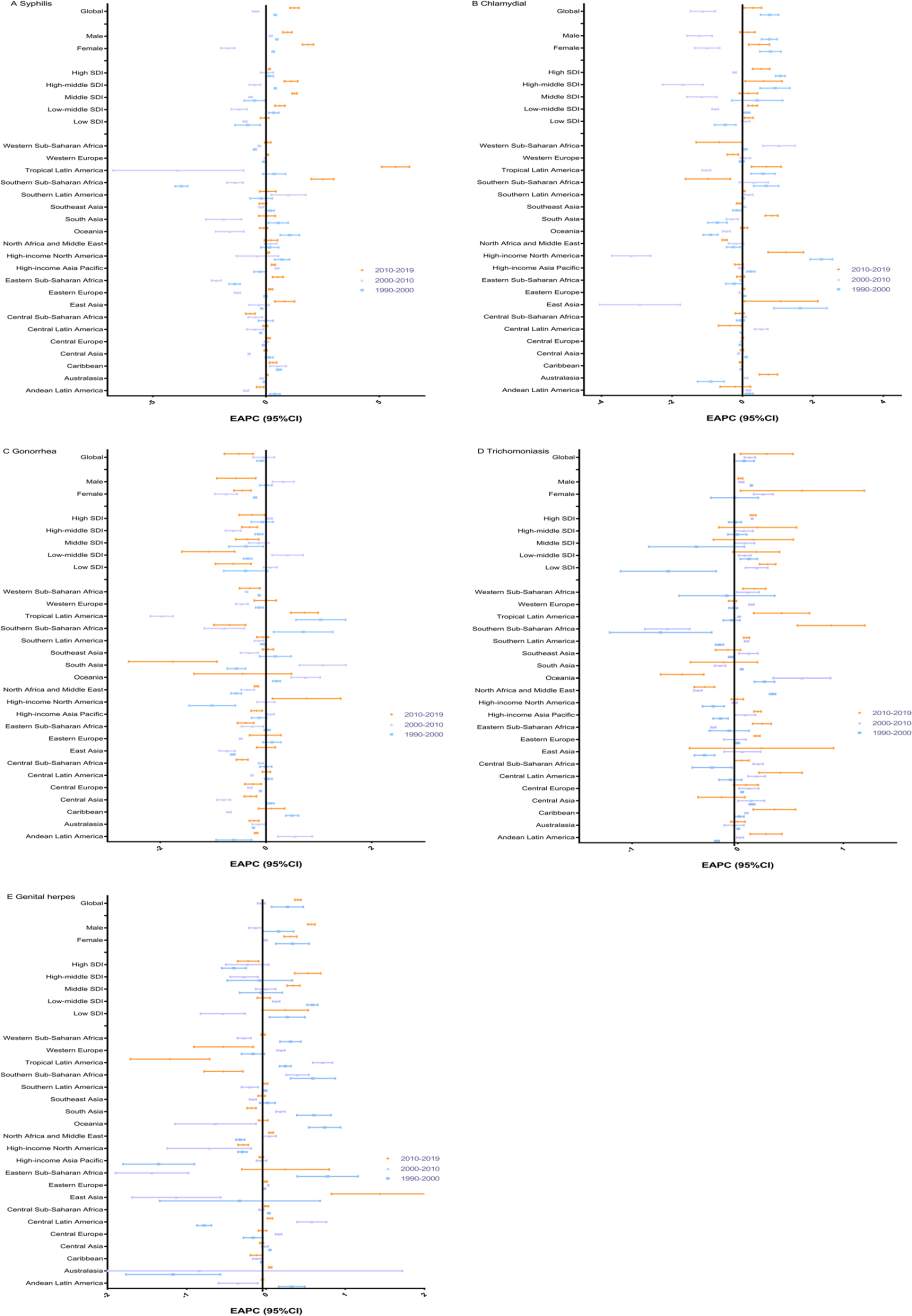
EAPCs of ASRs on STIs from 1990 to 2000, 2000 to 2010 and 2010 to 2019 by region. **A** syphilis, **B** chlamydia, **C** gonorrhea, **D** trichomoniasis, **E** genital herpes; *EAPCs* estimated annual percentage changes, *ASR* age-standardized incidence rate, *CI* confidence interval, *SDI* sociodemographic index

The GBD regions with the highest ASRs of syphilis, chlamydia, gonorrhea, trichomoniasis, and genital herpes were central sub-Saharan Africa (1048.40 per 100,000 people), southern sub-Saharan Africa (5324.43 per 100,000 people), southern sub-Saharan Africa (3869.56 per 100,000 people), and southern sub-Saharan Africa (2275.42 per 100,000 people) in 2019 (Tables 1, 2, 3, 4 and 5). From 2010 to 2019, 11, 6, 2, 10, and 5 GBD regions had a trend of increasing ASR for syphilis, chlamydia, gonorrhea, trichomoniasis, and genital herpes, respectively (Tables 1, 2, 3, 4 and 5). Among them, tropical Latin America had the greatest increasing trend (EAPC, 5.72; 95% CI 5.11–6.33) in syphilis cases (Table 1, Fig. 2A); meanwhile, high-income North America had the greatest increase in ASR for chlamydia (EAPC, 1.23; 95% CI 0.73–1.73) (Table 2 and Fig. 2B) and gonorrhea (EAPC, 0.77; 95% CI 0.12–1.41) (Table 3 and Fig. 2C), southern sub-Saharan Africa had the greatest increase in ASR for trichomoniasis (EAPC, 0.88; 95% CI 0.57–1.20) (Table 4 and Fig. 2D), and East Asia had the greatest increase in ASR for genital herpes (EAPC, 1.44; 95% CI 0.83–2.06) (Table 5 and Fig. 2E).

**Fig. 3:**
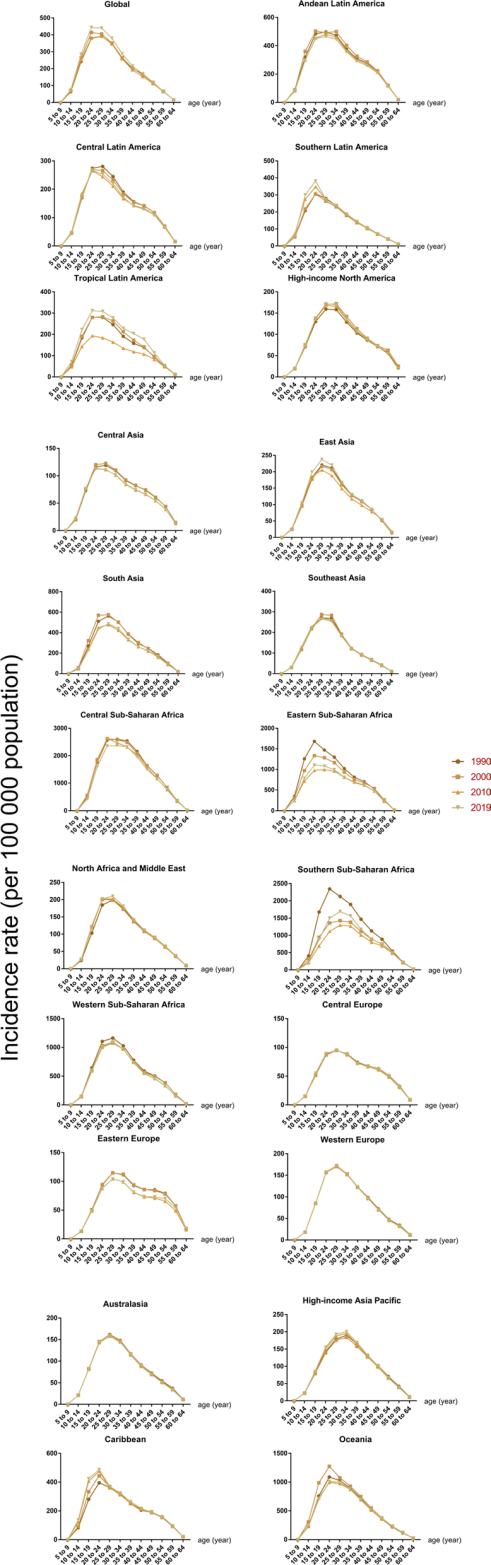
Incidence rate of syphilis by age groups and GBD regions, from 1990 to 2019

**Fig. 4:**
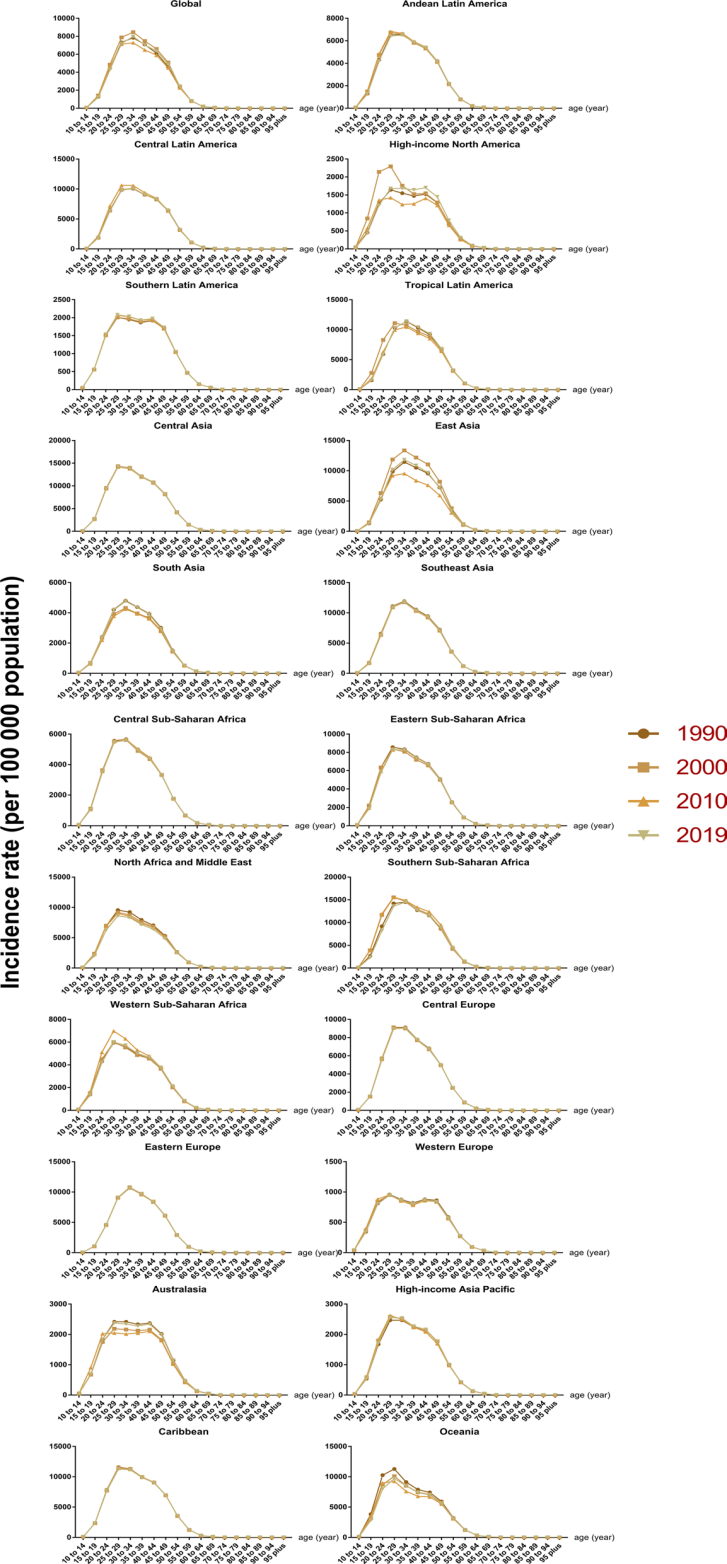
Incidence rate of chlamydia by age groups and GBD regions, from 1990 to 2019

In the most recent years, the population with the highest incidence of syphilis tended to be younger globally (25–29 years in 2010 vs. 20–24 years in 2019) but older in North Africa and the Middle East (20–24 years in 2010 vs. 25–29 years in 2019) in 2019 (Fig. 3). Additionally, for chlamydia, population tended to be older in southern sub-Saharan Africa (25–29 years in 2010 vs. 30–34 years in 2019) but younger in Australasia (40–44 years in 2010 vs. 25–29 years in 2019) (Fig. 4). Among gonorrhea and trichomoniasis cases, the characteristics of age were not recorded (Additional file 1: Figs. S4, S5). Population with genital herpes tended to be older in high-income North America (20–24 years in 2010 vs. 25–29 years in 2019) and South Asia (25–29 years in 2019 vs. 30–34 years in 2019) in 2019 (Additional file 1: Fig. S6).

## Discussion

To our knowledge, this is the first study to assess the long-term trends and differences in incidence rates of STIs, including syphilis, chlamydia, gonorrhea, trichomoniasis, and genital herpes, by geographical (global, regional, and national) regions and age groups using data from the 2019 GBD study. This paper focused on trends in STI incidence over the past decade at regional and age levels, highlighting that targeting prevention strategies to regions and age groups is vital for controlling STIs in the future.

We found that the ASRs of syphilis, trichomoniasis, and genital herpes were highest in low SDI regions from 1990 to 2019, while those of chlamydia and gonorrhea were highest in middle SDI regions from 1990 to 2019. The above information indicates that STIs remain epidemic in lower-income regions. Our study examines the trend of STIs from 2010 to 2019. We found that the trend of ASRs of syphilis, chlamydia, trichomoniasis, and genital herpes was increasing worldwide from 2010 to 2019. Notably, the greatest increase in ASRs of syphilis (tropical Latin America), chlamydia (high-income North America), and gonorrhea (high-income North America) all occurred in American regions. Tropical Latin America includes Brazil and Paraguay, which are both middle SDI regions. The SDI region with the greatest increase in ASR for syphilis was the middle SDI region, and Brazil. Swayze et al. observed that annual cases of syphilis in pregnant women increased between January 1, 2010, and December 31, 2018, in Brazil [14]. Brazil reported 724,310 acquired syphilis cases from 2011 to 2019 [15]. Meanwhile, the greatest increase in ASRs of chlamydia and gonorrhea occurred in high-income North America. Routine public health surveillance reported that chlamydia incidence rates increased from 394.9 per 100,000 people to 476.2 per 100,000 people and gonorrhea case rates increased from 78.0 per 100,000 people to 143.5 per 100,000 people from 2007 to 2016 in Winnipeg, Canada [16]. In other words, chlamydia was widespread geographically and gonorrhea was in a growth phase with geographic dispersion in Winnipeg, Canada, from 2007 to 2016 [16].

Furthermore, this study found that the population with the highest incidence of different STIs seemed to have changed by 2019 compared to that known 10 years ago. For syphilis, patients tended to be younger globally but older in North Africa and the Middle East. From 2014 to 2018, the population with a high incidence was aged 25–34 years but in 2019 was aged 20–24 years in China [17]. However, many studies have reported that the population with a high incidence of syphilis may be older in countries of North Africa and the Middle East [18]. Additionally, chlamydia population tended to be older in southern sub-Saharan Africa but younger in Australasia. There were some studies on age-related changes in the incidence of chlamydia in some countries of Australasia, including Australia [19, 20] and New Zealand [21]. Population with genital herpes tended to be older in high-income North America and South Asia in 2019. Few studies found that the percentages of patients with the herpes simplex virus were greater among older age groups in Eastern India [22] and Canada [23]. Previous studies usually divided age groups as < 20, 20–24, 25–44, and > 44 years, respectively, so it was found that the population with a high incidence was those aged 25–44 years, and is difficult to compare the change in the age group with a high incidence [24, 25]. The WHO proposed that there should be a priority focus on obtaining better STI data by age groups because of the need to measure progress toward STI control [5]. Our study not only highlighted the need for age-specific control measures in different regions, but also the need to strengthen STI surveillance in different age groups.

To control STIs, first, we should pay attention to reducing the percentages of high-risk sexual behaviors, especially among the youth. Peltzer et al. recorded increases among female adolescents in the prevalence rates of ever having sexual intercourse (from 24.4 to 36.4%) and having multiple sexual partners (from 13.6 to 16.9%) from 2007 to 2018 in Argentina [26]. Sharma et al. [27] reported that high-risk sexual behaviors have increased among adolescent boys (from 64 to 70%) and young men (from 18 to 27%) from 2005 to 2015 in India. It is very important to strengthen sexual health education in schools, including functional knowledge and skills to practice, adopt, and maintain healthy behaviors for preventing STIs [28, 29]. As most adolescents use mobile phones in their day-to-day lives, professional apps that provide an anonymous, free, and voluntary platform for information were considered [30, 31]. Additionally, getting treatment for partners may be effective in reducing STI incidence in youth [32]. Second, effective STI prevention interventions include screening, contact tracing of sexual partners, and promoting effective barrier contraception [33]. STI counseling and testing lead to positive sexual behavior changes [34]. Therefore, improving the quality rather than quantity of primary health care is particularly important. For example, incorporating sexual pleasure or security considerations within programming may have a positive impact on condom use, which has direct implications for reductions in STIs [35]. Moreover, the screening and testing of some high-risk populations, including men, transgender female sex workers, bisexual people, and other men who have sex with men are important regardless of self-reported sites of potential exposure [36, 37]. For bisexual people and men who have sex with men, the need for systematic multisite screening, regardless of symptoms, is also crucial [38]. Therefore, when resources are sufficient, monitoring work should be carried out among different populations according to location [36].

The data used for analysis were extracted from the 2019 GBD. We supplemented a comprehensive understanding of the incidence of STIs and further tried to develop global targeted prevention strategies. However, our study had several limitations. First, its main limitation is the accuracy and robustness of GBD estimates. The quality and quantity of GBD data largely depend on the data used in the modeling [11]. If countries lacked sufficient national systematic surveillance and population-based studies, the model may have a margin of bias. Furthermore, data derived from national epidemiological surveillance systems may be highly heterogeneous because of the differences in the quality of clinical and laboratory diagnostics, laboratory quality, and reporting standards among different countries, so the plausibility of the results would be influenced [12]. Second, because GBD data was estimated by modeling, an ecological fallacy may exist. Finally, sex is an important aspect in the spread of STIs, we only provided the trend of five STIs in female and male at global level, but did not conduct an analysis in depth. The analysis of STIs according to sex between different regions and age groups should be strengthened in the future.

## Conclusions

In this study, we found that the global trends of STIs—including syphilis, chlamydia, trichomoniasis, and genital herpes—all showed increasing ASRs from 2010 to 2019, which indicates a big challenge for controlling STIs in the future. Additionally, the differences in trends existed by geographical regions and age groups. American regions faced a big burden with a trend of the greatest increase in ASR for syphilis, chlamydia, and gonorrhea. Southern sub-Saharan Africa and East Asia had the greatest increase in ASR for trichomoniasis, and genital herpes, respectively. Moreover, in the most recent years, the population with high incidence rates of syphilis, chlamydia, and genital herpes tended to be younger or older in some regions in 2019. Therefore, a better understanding of STI epidemiological patterns is urgently needed for planning and implementing prevention and control strategies in key regions and population groups. Furthermore, to reduce the burden of STIs, it is essential to strengthen sexual health education and developing STI surveillance for key regions and populations.

## Supplementary Information

**Additional file 1: Table S1.** The GBD 2019 ICD Codes for STIs. **Figure S1.** The global incidence rate of sexually transmitted infections during 1990 to 2019. **Table S2.** The number of incident cases and changes of sexually transmitted infections in 1990 and 2019. **Figure S2.** Global trends in the incidence of sexually transmitted infections among 204 countries and territories. EAPC estimated annual percentage change. **Table S3.** The number of incident cases and age-standardized incidence rates (ASR, per 100,000 population) of sexually transmitted infections in 1990, 2000, 2010 and 2019, and their temporal trends among 204 countries or territories. **Table S4.** The age-standardized incidence rates (ASR, per 100,000 population) of sexually transmitted infections, and their temporal trends from 1990 to 2019. **Figure S3.** Incidence rate of STIs by age group and SDI region in 1990, 2000, 2010 and 2019. A: syphilis, B: chlamydia, C: gonorrhea, D: trichomoniasis, E: genital herpes; SDI socio-demographic index. **Figure S4.** Incidence rate of gonorrhoea by age and GBD region in 1990, 2000, 2010 and 2019. GBD: Global Burden of Disease Study. **Figure S5.** Incidence rate of trichomoniasis by age and GBD region in 1990, 2000, 2010 and 2019. GBD: Global Burden of Disease Study. **Figure S6.** Incidence rate of genital herpes by age and GBD region in 1990, 2000, 2010 and 2019. GBD: Global Burden of Disease Study.

## Data Availability

The datasets used in the present study are available in GBD 2019. This data can be found here: http://ghdx.healthdata.org/gbd-results-tool.

## Abbreviations

STIs: Sexually transmitted infections
GBD: Global Burden of Disease
EAPCs: Estimated annual percentage changes
ASR: Age-standardized incidence rate
WHO: World Health Organization
