# Supplementary material for: Increasing incidence rates of sexually transmitted infections from 2010 to 2019: an analysis of temporal trends by geographical regions and age groups from the 2019 Global Burden of Disease Study
Source: BMC Infect Dis. 2022 Jun 26;22:574. doi: 10.1186/s12879-022-07544-7 (PMC9233762; doi:10.1186/s12879-022-07544-7)
Supplement: Supplementary file 1 — Additional file 1: Table S1. The GBD 2019 ICD Codes for STIs. Figure S1. The global incidence rate of sexually transmitted infections during 1990 to 2019. Table S2. The number of incident cases and changes of sexually transmitted infections in 1990 and 2019. Figure S2. Global trends in the incidence of sexually transmitted infections among 204 countries and territories. EAPC estimated annual percentage change. Table S3. The number of incident cases and age-standardized incidence rates (ASR, per 100,000 population) of sexually transmitted infections in 1990, 2000, 2010 and 2019, and their temporal trends among 204 countries or territories. Table S4. The age-standardized incidence rates (ASR, per 100,000 population) of sexually transmitted infections, and their temporal trends from 1990 to 2019. Figure S3. Incidence rate of STIs by age group and SDI region in 1990, 2000, 2010 and 2019. A: syphilis, B: chlamydia, C: gonorrhea, D: trichomoniasis, E: genital herpes; SDI socio-demographic index. Figure S4. Incidence rate of gonorrhoea by age and GBD region in 1990, 2000, 2010 and 2019. GBD: Global Burden of Disease Study. Figure S5. Incidence rate of trichomoniasis by age and GBD region in 1990, 2000, 2010 and 2019. GBD: Global Burden of Disease Study. Figure S6. Incidence rate of genital herpes by age and GBD region in 1990, 2000, 2010 and 2019. GBD: Global Burden of Disease Study. [file 12879_2022_7544_MOESM1_ESM.docx]

**Table S1 The** **GBD 2019 ICD Codes for STIs.**

| Cause | ICD10 | ICD10 Used in Hospital/Claims Analyses | ICD9 | ICD9 Used in Hospital/Claims Analyses |
| --- | --- | --- | --- | --- |
| Syphilis | A50-A53.9, I98.0, K67.2, M73.1-M73.8 | A50-A52.9, I98.0 | 090-097.9 | 090-096.8 |
| Chlamydial infection | A55-A56.8, K67.0, N74.4 | A55-A56.11, K67.0, N74.4 | 099.41, 099.5 | 099.41-099.5 |
| Gonococcal infection | A54-A54.9, K67.1, M73.0, N74.3 | A54-A54.29, K67.1, N74.3 | 098-098.9 | 098-098.39 |
| Trichomoniasis | A59-A59.9 | A59-A59.9 | 131-131.9 | 131-131.9 |
| Genital herpes | A60-A60.9 | A60-A60.9 | 054.1, 054.11-054.19 | 054.1-054.19 |


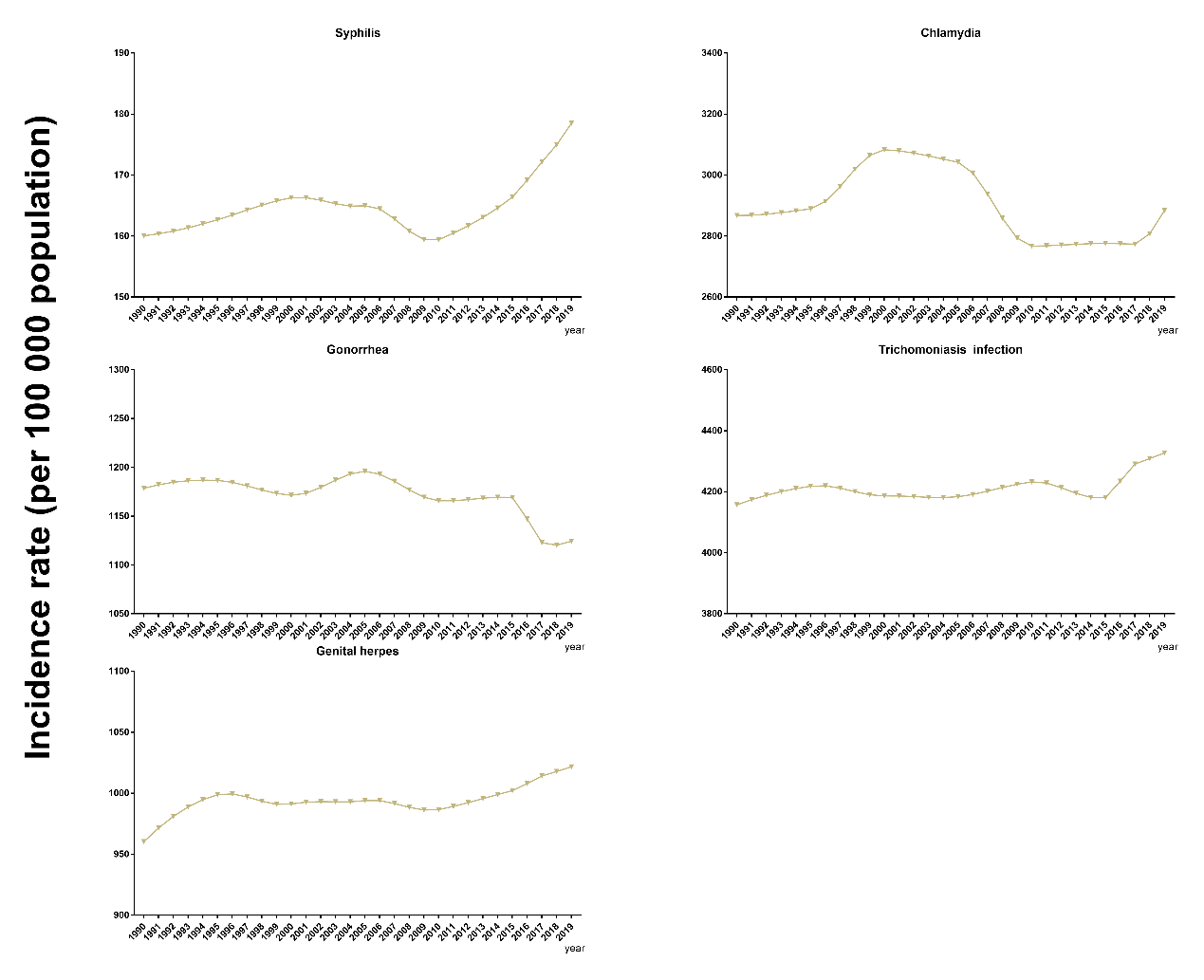


**Figure S1 The global incidence rate of sexually transmitted infections during 1990 to 2019.**

**Table S2 The number of incident cases and changes of sexually transmitted infections in 1990 and 2019**

| Characteristics | Number of incident cases (thousands) |  |  |
| --- | --- | --- | --- |
|  | 1990 (95% UI) | 2019 (95% UI) | Percentage change (%) 1990-2019 |
| **Syphilis** |  |  |  |
| Global | 8845.22 (6562.51, 11588.86) | 14114.11 (10648.49, 18415.97) | 59.57 |
| Sex |  |  |  |
| Female | 3355.45 (2502.21, 4388.06) | 4824.86 (3725.83, 6182.48) | 43.79 |
| Male | 5489.77 (4066.36, 7208.42) | 9289.25 (6919.20, 12282.79) | 69.21 |
| SDI region |  |  |  |
| Low | 2267.02 (1720.58, 2889.82) | 4116.13 (3146.92, 5287.15) | 81.57 |
| Low-middle | 2459.57 (1804.25, 3253.48) | 4042.00 (3000.00, 5301.94) | 64.34 |
| Middle | 2477.83 (1809.89, 3280.76) | 3310.60 (2471.37, 4388.12) | 33.61 |
| High-middle | 1011.91 (754.28, 1333.58) | 1348.09 (1026.99, 1774.22) | 33.22 |
| High | 623.30 (471.90, 826.55) | 721.92 (548.59, 951.88) | 15.82 |
| GBD region |  |  |  |
| Andean Latin America | 83.04 (61.30, 110.51) | 138.14 (104.70, 180.53) | 66.36 |
| Australasia | 14.10 (10.46, 18.96) | 17.71 (13.32, 23.46) | 25.63 |
| Caribbean | 61.44 (45.70, 81.46) | 92.37 (75.16, 113.65) | 50.34 |
| Central Asia | 37.39 (28.05, 49.21) | 51.63 (39.50, 66.88) | 38.08 |
| Central Europe | 54.15 (41.35, 71.11) | 47.32 (36.32, 61.63) | -12.62 |
| Central Latin America | 195.78 (143.30, 259.92) | 296.13 (227.21, 384.26) | 51.25 |
| Central Sub-Saharan Africa | 580.89 (435.01, 749.57) | 1332.45 (1017.53, 1723.69) | 129.38 |
| East Asia | 1224.26 (895.45, 1631.22) | 1459.54 (1092.02, 1958.96) | 19.22 |
| Eastern Europe | 125.74 (96.17, 165.15) | 102.35 (78.85, 136.35) | -18.60 |
| Eastern Sub-Saharan Africa | 1181.60 (916.25, 1480.69) | 1992.07 (1551.25, 2495.06) | 68.59 |
| High-income Asia Pacific | 145.87 (110.95, 192.31) | 136.11 (103.15, 181.01) | -6.69 |
| High-income North America | 205.18 (154.80, 273.09) | 252.31 (192.72, 331.60) | 22.97 |
| North Africa and Middle East | 262.02 (189.03, 348.87) | 562.03 (415.47, 750.43) | 114.50 |
| Oceania | 28.67 (20.98, 37.65) | 57.91 (42.04, 76.97) | 102.03 |
| South Asia | 2332.44 (1691.29, 3098.81) | 3725.19 (2730.12, 4965.90) | 59.71 |
| Southeast Asia | 487.64 (352.37, 649.91) | 722.67 (535.24, 961.37) | 48.20 |
| Southern Latin America | 58.65 (43.84, 77.44) | 88.79 (71.68, 110.38) | 51.40 |
| Southern Sub-Saharan Africa | 502.74 (381.66, 648.48) | 571.16 (420.69, 751.87) | 13.61 |
| Tropical Latin America | 190.64 (139.63, 255.94) | 332.79 (259.02, 415.93) | 74.57 |
| Western Europe | 269.53 (201.47, 355.93) | 263.00 (199.22, 348.20) | -2.42 |
| Western Sub-Saharan Africa | 803.45 (591.41, 1043.07) | 1872.43 (1377.02, 2458.53) | 133.05 |
| **Chlamydial infection** | |  |  |
| Global | 151695.68 (113998.56, 199144.01) | 232534.84 (174269.19, 303009.11) | 53.29 |
| Sex |  |  |  |
| Female | 67716.18 (51485.41, 88169.74) | 106935.60 (81029.30, 139409.31) | 57.92 |
| Male | 83979.50 (62121.37, 111433.61) | 125599.24 (93186.99, 164518.16) | 49.56 |
| SDI region |  |  |  |
| Low | 10987.51 (8267.17, 14395.59) | 23881.03 (17864.30, 31402.29) | 117.35 |
| Low-middle | 26592.63 (19958.90, 34845.68) | 46203.05 (34745.36, 60364.04) | 73.74 |
| Middle | 64740.67 (48605.29, 85278.85) | 91514.83 (68525.50, 118889.12) | 41.36 |
| High-middle | 39816.12 (29638.64, 52282.05) | 52103.68 (38909.69, 68070.89) | 30.86 |
| High | 9453.54 (7136.58, 12326.73) | 12557.26 (9503.56, 16097.73) | 32.83 |
| GBD region |  |  |  |
| Andean Latin America | 896.76 (665.31, 1171.19) | 1671.14 (1240.74, 2196.71) | 86.35 |
| Australasia | 233.48 (174.59, 303.88) | 304.57 (228.75, 392.63) | 30.45 |
| Caribbean | 1552.05 (1161.08, 2032.96) | 2116.06 (1579.50, 2760.07) | 36.34 |
| Central Asia | 3523.02 (2629.40, 4678.71) | 5347.63 (4011.01, 7000.18) | 51.79 |
| Central Europe | 4199.57 (3126.67, 5503.93) | 3749.00 (2796.76, 4867.00) | -10.73 |
| Central Latin America | 5921.99 (4464.42, 7722.56) | 10188.17 (7689.51, 13366.11) | 72.04 |
| Central Sub-Saharan Africa | 975.90 (724.48, 1295.62) | 2459.76 (1808.21, 3278.84) | 152.05 |
| East Asia | 53473.62 (39843.02, 70577.94) | 69208.69 (51730.09, 90227.82) | 29.43 |
| Eastern Europe | 8720.28 (6515.05, 11356.62) | 8021.15 (5945.68, 10566.80) | -8.02 |
| Eastern Sub-Saharan Africa | 5176.09 (3899.01, 6751.61) | 11947.26 (8919.21, 15716.64) | 130.82 |
| High-income Asia Pacific | 1900.40 (1430.40, 2459.84) | 1818.45 (1377.70, 2352.24) | -4.31 |
| High-income North America | 2125.36 (1589.06, 2797.12) | 2640.55 (2004.91, 3404.76) | 24.24 |
| North Africa and Middle East | 11189.88 (8450.63, 14569.26) | 22026.56 (16586.65, 28782.38) | 96.84 |
| Oceania | 263.63 (202.41, 332.98) | 497.73 (376.55, 646.71) | 88.80 |
| South Asia | 17312.76 (12939.60, 22983.08) | 32645.93 (24256.65, 42944.98) | 88.57 |
| Southeast Asia | 19519.57 (14690.93, 25768.43) | 31419.20 (23507.05, 40988.40) | 60.96 |
| Southern Latin America | 429.76 (326.76, 554.61) | 638.05 (481.36, 826.91) | 48.47 |
| Southern Sub-Saharan Africa | 2674.33 (2022.74, 3491.08) | 4537.13 (3412.07, 5945.06) | 69.65 |
| Tropical Latin America | 6153.27 (4576.43, 8178.21) | 10160.67 (7608.14, 13245.88) | 65.13 |
| Western Europe | 1765.79 (1345.72, 2296.88) | 1821.28 (1387.74, 2326.50) | 3.14 |
| Western Sub-Saharan Africa | 3688.15 (2764.90, 4870.38) | 9315.85 (6989.82, 12315.14) | 152.59 |
| **Gonococcal infection** | |  |  |
| Global | 67732.22 (51820.12, 89251.61) | 87951.95 (68461.02, 112961.84) | 29.85 |
| Sex |  |  |  |
| Female | 24249.60 (18176.84, 32574.08) | 28632.70 (22146.97, 37000.15) | 18.07 |
| Male | 43482.63 (33232.93, 57318.57) | 59319.26 (45872.40, 76110.80) | 36.42 |
| SDI region |  |  |  |
| Low | 6493.86 (4954.93, 8505.53) | 13311.88 (10136.01, 17794.05) | 104.99 |
| Low-middle | 14601.88 (11111.17, 19326.71) | 22100.89 (17018.65, 28800.09) | 51.36 |
| Middle | 26685.20 (19832.41, 36187.31) | 30185.57 (23252.89, 39609.01) | 13.12 |
| High-middle | 16032.64 (12328.77, 21132.25) | 15652.96 (12166.99, 20103.29) | -2.37 |
| High | 3881.18 (3109.10, 4948.05) | 4193.30 (3379.69, 5265.41) | 8.04 |
| GBD region |  |  |  |
| Andean Latin America | 104.32 (71.40, 154.59) | 170.41 (120.24, 244.58) | 63.35 |
| Australasia | 67.59 (51.53, 88.83) | 81.25 (62.30, 106.35) | 20.21 |
| Caribbean | 479.03 (322.38, 733.91) | 575.18 (399.66, 855.10) | 20.07 |
| Central Asia | 1835.57 (1220.28, 2750.02) | 2192.50 (1500.13, 3278.28) | 19.44 |
| Central Europe | 2257.75 (1729.32, 3053.13) | 1671.53 (1310.71, 2183.02) | -25.96 |
| Central Latin America | 1421.82 (1088.63, 1879.11) | 2060.35 (1606.45, 2650.38) | 44.91 |
| Central Sub-Saharan Africa | 671.99 (455.63, 994.90) | 1608.74 (1094.81, 2396.95) | 139.40 |
| East Asia | 18170.09 (12540.69, 26214.00) | 14929.58 (10824.81, 20281.77) | -17.83 |
| Eastern Europe | 4660.52 (3431.44, 6400.48) | 3608.59 (2639.90, 4898.40) | -22.57 |
| Eastern Sub-Saharan Africa | 2711.81 (2005.51, 3677.88) | 6055.17 (4502.20, 8305.34) | 123.29 |
| High-income Asia Pacific | 1248.93 (995.48, 1561.99) | 1051.07 (855.02, 1317.75) | -15.84 |
| High-income North America | 1064.05 (795.09, 1413.07) | 1266.29 (956.62, 1679.17) | 19.01 |
| North Africa and Middle East | 4782.86 (3445.48, 6760.76) | 7664.76 (5628.62, 10813.33) | 60.25 |
| Oceania | 122.08 (80.19, 188.98) | 244.93 (161.32, 377.42) | 100.63 |
| South Asia | 14044.60 (9932.30, 20014.49) | 22793.16 (16284.78, 32261.40) | 62.29 |
| Southeast Asia | 6751.48 (5297.40, 8562.08) | 9590.32 (7623.98, 12122.15) | 42.05 |
| Southern Latin America | 219.50 (155.93, 311.84) | 289.06 (209.85, 400.24) | 31.69 |
| Southern Sub-Saharan Africa | 2398.06 (1788.86, 3210.42) | 3316.11 (2520.35, 4404.29) | 38.28 |
| Tropical Latin America | 1577.37 (1080.32, 2290.51) | 2111.25 (1470.97, 2974.90) | 33.85 |
| Western Europe | 530.59 (427.60, 661.07) | 518.61 (422.09, 638.67) | -2.26 |
| Western Sub-Saharan Africa | 2612.18 (2040.39, 3385.85) | 6153.10 (4781.85, 8077.31) | 135.55 |
| Trichomoniasis |  |  |  |
| Global | 205446.49 (151261.12, 273107.88) | 354466.58 (260117.34, 461359.68) | 72.53 |
| Sex |  |  |  |
| Female | 87515.34 (64115.94, 116821.70) | 153839.49 (110414.26, 202493.34) | 75.79 |
| Male | 117931.15 (87212.23, 157404.94) | 200627.08 (147915.66, 262592.84) | 70.12 |
| SDI region |  |  |  |
| Low | 23740.30 (17708.37, 31547.14) | 50979.93 (37726.77, 67927.47) | 114.74 |
| Low-middle | 35897.74 (26582.21, 48091.83) | 70589.53 (51840.01, 93425.62) | 96.64 |
| Middle | 69999.74 (51501.40, 93455.03) | 114940.44 (83136.09, 149423.60) | 64.20 |
| High-middle | 44639.06 (32782.48, 58981.20) | 66379.85 (48427.61, 85845.12) | 48.70 |
| High | 31046.53 (22769.39, 40437.30) | 40190.36 (29714.89, 51420.48) | 29.45 |
| GBD region |  |  |  |
| Andean Latin America | 1373.84 (1020.52, 1838.42) | 2880.37 (2107.80, 3789.12) | 109.66 |
| Australasia | 622.14 (460.35, 812.40) | 906.34 (682.76, 1150.98) | 45.68 |
| Caribbean | 1617.28 (1209.99, 2133.14) | 2592.94 (1917.98, 3351.10) | 60.33 |
| Central Asia | 2331.22 (1729.71, 3107.71) | 3894.82 (2866.22, 5146.67) | 67.07 |
| Central Europe | 4495.78 (3303.58, 5858.50) | 4655.82 (3465.49, 5986.45) | 3.56 |
| Central Latin America | 8362.00 (6196.65, 11160.86) | 17017.78 (12379.44, 22233.78) | 103.51 |
| Central Sub-Saharan Africa | 2148.80 (1591.39, 2878.02) | 5528.89 (4084.66, 7375.25) | 157.30 |
| East Asia | 54526.57 (39873.29, 72786.08) | 81206.63 (59123.78, 105278.99) | 48.93 |
| Eastern Europe | 7777.40 (5685.26, 10115.93) | 7876.49 (5747.57, 10200.43) | 1.27 |
| Eastern Sub-Saharan Africa | 13108.52 (9806.23, 17367.56) | 31208.96 (23048.28, 41455.55) | 138.08 |
| High-income Asia Pacific | 6317.33 (4573.29, 8212.48) | 7022.47 (5251.12, 8975.23) | 11.16 |
| High-income North America | 13540.65 (9769.64, 17819.07) | 16698.20 (12367.48, 21352.39) | 23.32 |
| North Africa and Middle East | 9866.86 (7396.20, 13071.46) | 23134.99 (16947.80, 30356.56) | 134.47 |
| Oceania | 362.86 (274.02, 486.88) | 861.99 (639.72, 1133.49) | 137.56 |
| South Asia | 27503.13 (20138.86, 37028.79) | 54130.23 (39501.79, 71561.39) | 96.81 |
| Southeast Asia | 17805.82 (13166.51, 23884.88) | 32948.13 (23834.02, 43183.15) | 85.04 |
| Southern Latin America | 1417.84 (1049.53, 1850.02) | 2152.14 (1602.60, 2788.57) | 51.79 |
| Southern Sub-Saharan Africa | 3670.48 (2709.32, 4823.33) | 6299.96 (4588.35, 8296.73) | 71.64 |
| Tropical Latin America | 7809.61 (5668.28, 10461.71) | 14878.42 (10701.26, 19485.21) | 90.51 |
| Western Europe | 10272.00 (7743.45, 13211.34) | 11847.99 (8931.24, 14996.21) | 15.34 |
| Western Sub-Saharan Africa | 10516.36 (7889.17, 14018.11) | 26723.00 (19848.35, 35685.96) | 154.11 |
| **Genital herpes** |  |  |  |
| Global | 53051.59 (45029.38, 61934.04) | 80784.43 (68810.96, 94200.33) | 52.28 |
| Sex |  |  |  |
| Female | 32826.29 (28066.35, 38105.21) | 49547.19 (42417.73, 57406.65) | 50.94 |
| Male | 20225.30 (16909.53, 23827.90) | 31237.24 (26476.07, 36801.24) | 54.45 |
| SDI region |  |  |  |
| Low | 6984.24 (5997.04, 8117.01) | 15423.85 (13096.08, 18113.34) | 120.84 |
| Low-middle | 9713.83 (8187.31, 11491.74) | 17879.52 (15120.82, 20828.79) | 84.06 |
| Middle | 17684.07 (14969.97, 20668.81) | 24944.85 (21164.99, 29188.02) | 41.06 |
| High-middle | 10560.19 (8891.01, 12403.57) | 12906.30 (10984.14, 15223.24) | 22.22 |
| High | 8069.12 (6890.10, 9374.77) | 8141.61 (6945.86, 9549.75) | 0.90 |
| GBD region |  |  |  |
| Andean Latin America | 705.57 (617.73, 809.55) | 1168.68 (999.05, 1351.49) | 65.64 |
| Australasia | 206.02 (167.18, 248.79) | 211.46 (177.78, 249.82) | 2.64 |
| Caribbean | 615.58 (522.37, 718.82) | 760.27 (648.32, 887.50) | 23.50 |
| Central Asia | 469.49 (388.29, 560.09) | 692.11 (579.27, 828.18) | 47.42 |
| Central Europe | 621.99 (526.10, 735.17) | 556.25 (467.26, 664.59) | -10.57 |
| Central Latin America | 2644.87 (2282.09, 3066.33) | 3977.09 (3390.14, 4624.08) | 50.37 |
| Central Sub-Saharan Africa | 1273.31 (1099.50, 1457.05) | 3167.91 (2749.28, 3626.10) | 148.79 |
| East Asia | 10330.94 (8538.86, 12261.69) | 12099.98 (10180.78, 14394.45) | 17.12 |
| Eastern Europe | 2150.80 (1812.01, 2553.29) | 1851.02 (1569.09, 2189.07) | -13.94 |
| Eastern Sub-Saharan Africa | 3848.31 (3300.77, 4454.20) | 8539.28 (7223.96, 10055.09) | 121.90 |
| High-income Asia Pacific | 1460.50 (1315.96, 1637.98) | 1136.25 (961.33, 1349.95) | -22.20 |
| High-income North America | 3617.02 (3045.41, 4256.01) | 3622.26 (3095.93, 4232.98) | 0.15 |
| North Africa and Middle East | 2743.40 (2324.38, 3250.38) | 5291.00 (4457.33, 6313.04) | 92.86 |
| Oceania | 94.14 (78.70, 112.43) | 200.39 (168.56, 236.72) | 112.86 |
| South Asia | 5979.32 (4980.66, 7170.08) | 11993.16 (10070.18, 14347.08) | 100.58 |
| Southeast Asia | 5397.90 (4591.20, 6334.95) | 7868.72 (6643.66, 9246.56) | 45.77 |
| Southern Latin America | 621.61 (568.04, 679.99) | 822.20 (701.75, 955.58) | 32.27 |
| Southern Sub-Saharan Africa | 1230.30 (1055.21, 1423.56) | 1997.94 (1730.62, 2288.97) | 62.39 |
| Tropical Latin America | 3179.56 (2705.36, 3700.49) | 4545.92 (3914.56, 5251.34) | 42.97 |
| Western Europe | 2867.22 (2439.42, 3369.26) | 2739.26 (2334.58, 3227.83) | -4.46 |
| Western Sub-Saharan Africa | 2993.75 (2534.34, 3511.28) | 7543.31 (6368.75, 8870.60) | 151.97 |


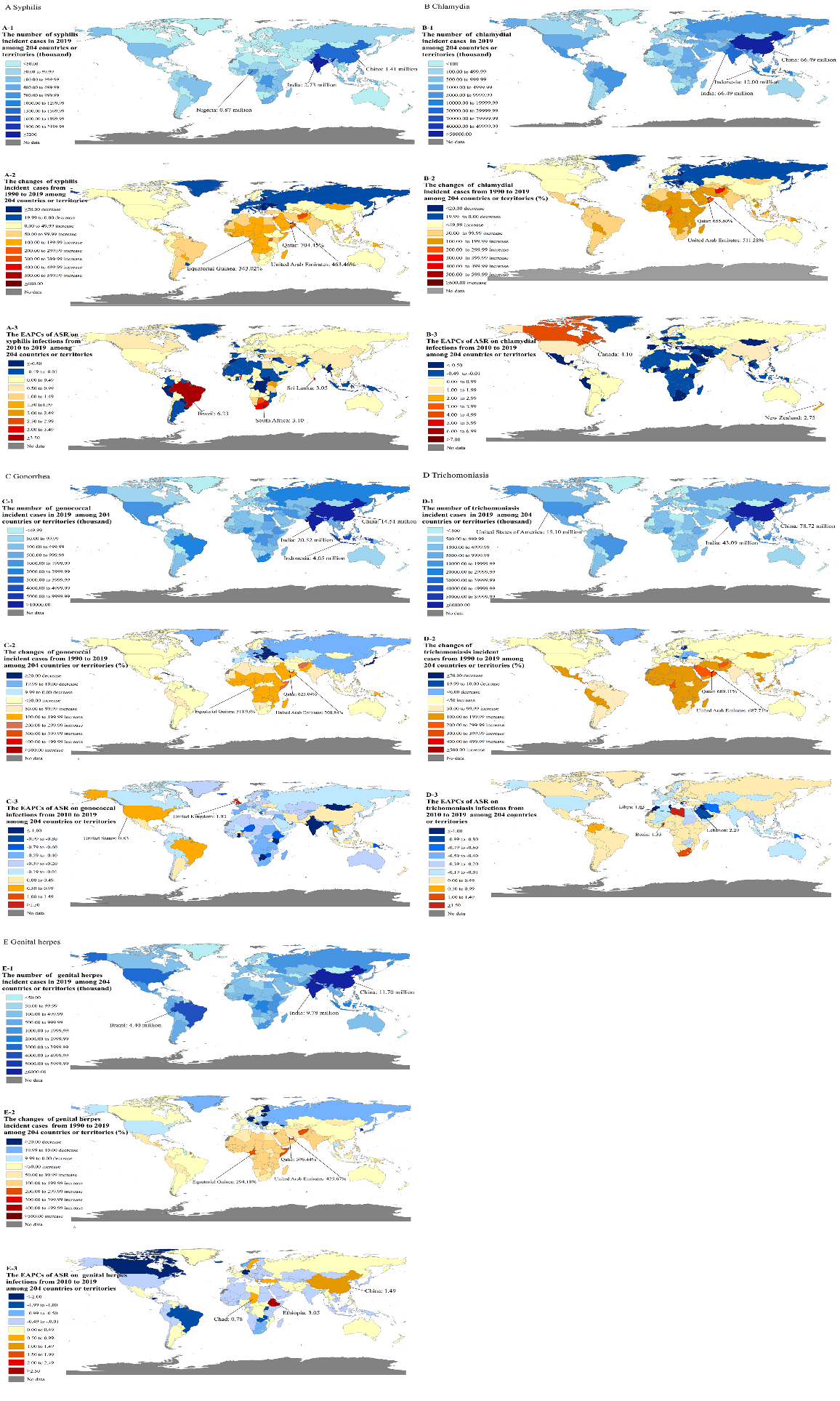


**Figure S2 Global trends in the incidence of sexually transmitted infections among 204 countries and territories. EAPC estimated annual percentage change**

**Table S3 The number of incident cases and age-standardized incidence rates (ASR, per 100 000 population) of sexually transmitted infections in 1990, 2000, 2010 and 2019, and their temporal trends among 204 countries or territories.**

| Characteristics | Number of incident cases (thousands) | | | Age-standardized incidence rate (ASR, per 100 000) | | | | |  |  |  |
| --- | --- | --- | --- | --- | --- | --- | --- | --- | --- | --- | --- |
|  | 1990 (95% UI) | 2019 (95% UI) | Percentage change (%) 1990-2019 | 1990 (95% UI) | 2000 (95% UI) | 2010 (95% UI) | 2019 (95% UI) | EAPC (95% CI) 1990-2019 | EAPC (95% CI) 1990-2000 | EAPC (95% CI) 2000-2010 | EAPC (95% CI) 2010-2019 |
| **Syphilis** |  |  |  |  |  |  |  |  |  |  |  |
| Afghanistan | 9.01 (6.55, 12.10) | 37.63 (26.80, 50.40) | 317.54 | 95.10 (70.08, 126.09) | 98.88 (72.35, 131.13) | 103.44 (78.79, 133.94) | 103.30 (75.87, 137.53) | 0.25 (0.20, 0.30) | 0.38 (0.26, 0.50) | 0.46 (0.39, 0.53) | 0.02 (-0.26, 0.30) |
| Albania | 1.36 (1.02, 1.79) | 1.08 (0.82, 1.41) | -20.69 | 40.10 (30.59, 52.45) | 38.89 (29.93, 50.83) | 38.46 (29.53, 50.40) | 39.12 (29.65, 52.02) | -0.10 (-0.15, -0.06) | -0.28 (-0.34, -0.23) | -0.10 (-0.12, -0.09) | 0.24 (0.18, 0.29) |
| Algeria | 20.29 (14.66, 27.14) | 36.45 (27.10, 48.41) | 79.60 | 85.05 (63.24, 113.24) | 88.06 (65.99, 114.99) | 82.94 (62.60, 110.73) | 80.44 (60.14, 107.08) | -0.24 (-0.40, -0.09) | 0.33 (0.16, 0.50) | -0.60 (-1.15, -0.05) | -0.32 (-0.44, -0.21) |
| American Samoa | 0.15 (0.11, 0.21) | 0.17 (0.13, 0.23) | 10.41 | 305.73 (226.70, 408.00) | 301.66 (225.02, 399.27) | 300.62 (223.44, 398.31) | 305.40 (226.00, 402.22) | -0.03 (-0.05, -0.01) | -0.12 (-0.17, -0.08) | -0.04 (-0.04, -0.03) | 0.14 (0.03, 0.24) |
| Andorra | 0.05 (0.03, 0.06) | 0.05 (0.04, 0.07) | 18.53 | 70.27 (52.27, 93.58) | 68.75 (50.82, 94.31) | 67.84 (50.16, 90.77) | 67.71 (50.17, 90.19) | -0.16 (-0.18, -0.13) | -0.28 (-0.35, -0.21) | -0.07 (-0.14, 0.00) | 0.06 (-0.03, 0.14) |
| Angola | 117.22 (87.59, 153.33) | 307.09 (227.63, 405.07) | 161.98 | 1230.46 (933.24, 1593.06) | 1194.07 (944.43, 1487.06) | 1102.25 (832.46, 1426.32) | 1100.14 (821.74, 1451.68) | -0.50 (-0.55, -0.45) | -0.30 (-0.38, -0.23) | -0.82 (-0.94, -0.71) | 0.01 (-0.05, 0.07) |
| Antigua and Barbuda | 0.08 (0.06, 0.11) | 0.13 (0.10, 0.17) | 52.50 | 134.12 (100.65, 177.76) | 128.44 (98.46, 168.29) | 129.16 (99.36, 168.50) | 133.89 (100.93, 175.64) | -0.04 (-0.09, 0.02) | -0.45 (-0.49, -0.40) | 0.07 (0.02, 0.11) | 0.37 (0.27, 0.47) |
| Argentina | 42.11 (31.38, 55.35) | 70.78 (57.28, 88.27) | 68.09 | 130.58 (97.10, 171.59) | 124.18 (98.39, 154.76) | 150.57 (124.77, 178.36) | 154.39 (125.58, 191.78) | 0.54 (0.21, 0.87) | -0.60 (-1.33, 0.13) | 2.08 (0.73, 3.44) | -0.05 (-0.42, 0.33) |
| Armenia | 1.80 (1.36, 2.31) | 1.40 (1.06, 1.85) | -22.26 | 50.90 (38.93, 65.55) | 49.77 (38.05, 64.10) | 44.00 (33.36, 57.70) | 44.97 (34.08, 59.58) | -0.70 (-0.80, -0.61) | -0.25 (-0.49, 0.00) | -1.29 (-1.36, -1.22) | 0.22 (0.13, 0.32) |
| Australia | 11.63 (8.62, 15.66) | 14.99 (11.29, 19.87) | 28.91 | 65.01 (48.42, 87.06) | 64.80 (48.44, 86.21) | 63.56 (47.33, 84.15) | 63.62 (47.49, 85.10) | -0.11 (-0.13, -0.09) | -0.04 (-0.08, 0.00) | -0.20 (-0.26, -0.13) | 0.02 (0.01, 0.02) |
| Austria | 5.49 (4.04, 7.37) | 5.50 (4.08, 7.35) | 0.10 | 67.11 (49.88, 89.89) | 65.99 (48.56, 89.42) | 65.32 (48.27, 87.29) | 66.22 (48.24, 89.03) | -0.08 (-0.10, -0.05) | -0.18 (-0.22, -0.14) | -0.10 (-0.14, -0.06) | 0.14 (0.11, 0.18) |
| Azerbaijan | 4.28 (3.18, 5.65) | 5.93 (4.51, 7.83) | 38.43 | 56.57 (43.01, 73.98) | 55.04 (42.00, 72.75) | 50.74 (38.60, 66.62) | 51.25 (39.22, 67.69) | -0.50 (-0.58, -0.42) | -0.27 (-0.43, -0.12) | -0.83 (-1.04, -0.63) | 0.11 (0.10, 0.12) |
| Bahamas | 0.51 (0.37, 0.68) | 0.74 (0.57, 0.93) | 45.12 | 175.63 (129.99, 231.29) | 172.28 (130.30, 226.33) | 190.49 (150.28, 238.05) | 180.80 (140.30, 229.80) | 0.40 (0.29, 0.51) | -0.20 (-0.21, -0.18) | 1.06 (0.81, 1.30) | -0.43 (-0.81, -0.05) |
| Bahrain | 0.61 (0.43, 0.83) | 1.74 (1.28, 2.34) | 185.85 | 96.67 (71.40, 130.12) | 95.73 (70.57, 130.24) | 98.98 (73.15, 132.33) | 95.56 (71.54, 127.39) | 0.08 (0.03, 0.13) | -0.10 (-0.15, -0.06) | 0.37 (0.26, 0.48) | -0.40 (-0.53, -0.28) |
| Bangladesh | 257.42 (185.75, 340.37) | 370.37 (275.66, 493.42) | 43.88 | 252.70 (188.67, 331.70) | 270.28 (204.40, 350.51) | 214.66 (160.03, 286.96) | 213.44 (160.21, 283.87) | -1.13 (-1.34, -0.93) | 0.59 (0.03, 1.16) | -2.40 (-2.63, -2.17) | -0.07 (-0.14, 0.01) |
| Barbados | 0.31 (0.23, 0.40) | 0.33 (0.25, 0.42) | 7.19 | 113.65 (86.32, 148.32) | 112.65 (85.57, 149.25) | 112.48 (85.59, 146.53) | 112.43 (85.60, 147.68) | -0.04 (-0.06, -0.03) | -0.10 (-0.15, -0.04) | -0.01 (-0.10, 0.08) | 0.01 (-0.01, 0.03) |
| Belarus | 4.82 (3.72, 6.24) | 3.98 (3.06, 5.19) | -17.55 | 44.46 (34.34, 57.71) | 43.62 (33.23, 56.41) | 40.02 (31.21, 51.98) | 40.97 (31.55, 52.64) | -0.44 (-0.51, -0.37) | -0.18 (-0.20, -0.17) | -0.89 (-1.04, -0.73) | 0.25 (0.12, 0.38) |
| Belgium | 6.86 (5.07, 9.21) | 6.75 (5.08, 9.02) | -1.64 | 67.39 (49.42, 90.36) | 66.68 (48.92, 90.06) | 65.38 (48.33, 86.25) | 65.82 (48.90, 88.09) | -0.13 (-0.15, -0.12) | -0.11 (-0.16, -0.07) | -0.20 (-0.23, -0.17) | 0.05 (0.00, 0.10) |
| Belize | 0.23 (0.17, 0.31) | 0.59 (0.43, 0.78) | 155.75 | 132.25 (100.13, 174.35) | 128.42 (96.97, 170.12) | 124.50 (95.70, 159.56) | 130.45 (98.62, 172.68) | -0.15 (-0.20, -0.10) | -0.28 (-0.34, -0.22) | -0.32 (-0.46, -0.17) | 0.41 (0.18, 0.64) |
| Benin | 10.54 (7.73, 13.82) | 27.31 (19.96, 36.60) | 159.04 | 254.39 (190.70, 330.02) | 265.24 (217.42, 321.05) | 188.74 (145.74, 245.37) | 231.72 (173.74, 307.84) | -1.11 (-1.49, -0.72) | 0.47 (0.36, 0.58) | -3.62 (-4.00, -3.24) | 2.71 (1.65, 3.78) |
| Bermuda | 0.10 (0.07, 0.13) | 0.09 (0.07, 0.11) | -14.82 | 149.45 (113.30, 196.59) | 145.36 (110.84, 191.79) | 147.63 (110.95, 195.14) | 149.53 (112.69, 195.63) | 0.05 (0.02, 0.08) | -0.27 (-0.31, -0.24) | 0.16 (0.10, 0.23) | 0.20 (0.13, 0.27) |
| Bhutan | 1.94 (1.39, 2.60) | 2.65 (1.92, 3.55) | 36.77 | 303.28 (221.76, 398.51) | 343.57 (259.67, 439.56) | 297.38 (217.80, 395.01) | 299.74 (219.22, 397.51) | -0.47 (-0.64, -0.30) | 1.17 (0.75, 1.58) | -1.54 (-1.87, -1.21) | 0.10 (0.08, 0.12) |
| Bolivia (Plurinational State of) | 15.49 (11.35, 21.10) | 30.20 (23.40, 38.71) | 94.94 | 255.28 (189.85, 338.75) | 272.54 (204.74, 357.34) | 242.95 (194.15, 303.54) | 245.51 (192.64, 314.14) | -0.52 (-0.65, -0.39) | 0.53 (0.00, 1.06) | -1.22 (-1.32, -1.11) | 0.09 (0.05, 0.13) |
| Bosnia and Herzegovina | 1.95 (1.48, 2.56) | 1.26 (0.97, 1.66) | -35.29 | 39.01 (29.62, 51.16) | 38.64 (29.25, 51.06) | 38.49 (29.12, 50.82) | 38.90 (29.64, 51.74) | 0.03 (0.00, 0.06) | -0.10 (-0.25, 0.05) | -0.04 (-0.15, 0.06) | 0.16 (0.11, 0.21) |
| Botswana | 11.03 (8.61, 13.55) | 15.56 (11.27, 21.06) | 41.02 | 835.96 (649.73, 1030.53) | 620.62 (497.26, 761.19) | 467.94 (375.77, 580.70) | 586.19 (427.66, 779.65) | -1.89 (-2.44, -1.33) | -2.93 (-4.03, -1.82) | -2.80 (-3.12, -2.47) | 2.95 (2.54, 3.35) |
| Brazil | 183.00 (134.20, 245.90) | 315.94 (245.61, 397.01) | 72.65 | 117.15 (87.05, 156.31) | 121.79 (89.74, 163.72) | 81.11 (63.65, 100.76) | 136.77 (106.75, 171.04) | -0.81 (-1.73, 0.12) | 0.36 (-0.16, 0.87) | -4.41 (-7.31, -1.43) | 6.22 (5.52, 6.92) |
| Brunei Darussalam | 0.22 (0.16, 0.30) | 0.40 (0.30, 0.54) | 80.64 | 77.17 (57.71, 102.61) | 74.42 (56.59, 97.56) | 74.98 (56.74, 99.27) | 75.59 (56.78, 100.85) | -0.02 (-0.07, 0.03) | -0.36 (-0.41, -0.30) | 0.08 (0.00, 0.17) | 0.11 (0.08, 0.15) |
| Bulgaria | 3.14 (2.40, 4.14) | 2.33 (1.80, 3.05) | -25.76 | 36.24 (27.52, 47.64) | 36.16 (27.94, 47.48) | 35.68 (27.10, 46.92) | 36.07 (27.75, 47.24) | -0.03 (-0.05, -0.01) | -0.02 (-0.04, 0.00) | -0.15 (-0.17, -0.14) | 0.16 (0.10, 0.22) |
| Burkina Faso | 26.14 (19.21, 34.49) | 71.08 (55.28, 90.13) | 171.96 | 333.26 (248.03, 439.26) | 309.22 (238.26, 395.16) | 343.08 (268.48, 433.14) | 340.63 (264.25, 430.58) | 0.35 (0.23, 0.47) | -0.73 (-0.81, -0.64) | 1.09 (1.03, 1.16) | 0.01 (-0.15, 0.18) |
| Burundi | 20.72 (15.42, 27.00) | 38.26 (28.52, 49.94) | 84.68 | 426.92 (324.83, 546.67) | 471.87 (374.02, 591.38) | 365.88 (283.26, 468.26) | 359.61 (274.65, 468.28) | -0.85 (-1.15, -0.55) | 1.10 (0.56, 1.65) | -2.64 (-3.33, -1.95) | -0.17 (-0.27, -0.07) |
| Cabo Verde | 1.63 (1.17, 2.20) | 3.50 (2.55, 4.67) | 114.36 | 518.99 (381.94, 687.21) | 534.28 (391.90, 700.81) | 548.48 (405.53, 720.10) | 555.54 (413.98, 734.41) | 0.17 (0.10, 0.24) | 0.26 (0.12, 0.39) | 0.28 (-0.10, 0.66) | 0.04 (-0.11, 0.19) |
| Cambodia | 3.94 (2.88, 5.28) | 7.56 (5.55, 10.11) | 91.88 | 42.06 (31.08, 55.81) | 43.17 (32.49, 56.98) | 38.85 (29.19, 50.73) | 41.91 (31.29, 55.80) | -0.29 (-0.43, -0.14) | 0.25 (0.20, 0.30) | -1.12 (-1.47, -0.76) | 1.10 (0.56, 1.64) |
| Cameroon | 76.78 (57.59, 100.29) | 186.33 (133.68, 250.24) | 142.70 | 785.78 (590.37, 1022.57) | 706.96 (569.89, 870.87) | 645.95 (486.87, 829.10) | 628.82 (457.38, 832.52) | -0.84 (-0.96, -0.73) | -1.14 (-1.48, -0.79) | -0.92 (-1.02, -0.83) | -0.17 (-0.40, 0.06) |
| Canada | 15.54 (11.61, 20.38) | 17.77 (13.61, 23.27) | 14.31 | 51.88 (38.93, 67.69) | 50.90 (38.31, 67.09) | 48.16 (36.87, 63.38) | 51.47 (38.57, 68.45) | -0.09 (-0.21, 0.02) | -0.18 (-0.23, -0.13) | -0.57 (-0.81, -0.34) | 0.82 (0.61, 1.03) |
| Central African Republic | 31.38 (22.97, 40.79) | 66.91 (50.14, 86.12) | 113.23 | 1227.03 (921.46, 1586.78) | 1264.68 (971.52, 1601.03) | 1203.53 (933.03, 1553.69) | 1264.81 (978.77, 1613.28) | -0.22 (-0.30, -0.14) | 0.26 (0.04, 0.48) | -0.52 (-0.69, -0.34) | 0.23 (-0.24, 0.71) |
| Chad | 26.05 (19.05, 34.18) | 67.93 (49.48, 90.89) | 160.73 | 499.06 (368.10, 653.57) | 530.57 (414.83, 660.89) | 470.31 (362.00, 594.01) | 475.46 (354.01, 630.82) | -0.39 (-0.52, -0.26) | 0.62 (0.55, 0.69) | -1.27 (-1.55, -1.00) | 0.18 (0.12, 0.24) |
| Chile | 12.67 (9.29, 17.05) | 14.15 (10.96, 18.67) | 11.67 | 88.01 (65.96, 117.23) | 95.13 (70.65, 124.92) | 69.96 (55.13, 89.13) | 75.80 (59.00, 100.47) | -1.13 (-1.50, -0.76) | 0.80 (0.51, 1.09) | -3.06 (-4.37, -1.73) | 0.53 (-0.11, 1.18) |
| China | 1183.22 (864.09, 1577.08) | 1411.51 (1055.48, 1896.97) | 19.29 | 89.23 (66.57, 118.21) | 87.36 (65.26, 116.11) | 84.73 (63.52, 111.74) | 93.44 (69.26, 124.09) | -0.09 (-0.22, 0.05) | -0.21 (-0.24, -0.17) | -0.32 (-0.77, 0.14) | 0.84 (0.39, 1.29) |
| Colombia | 64.79 (47.21, 86.26) | 94.04 (73.01, 121.23) | 45.14 | 189.71 (141.47, 248.12) | 187.72 (140.96, 245.98) | 189.31 (148.91, 238.48) | 185.95 (144.45, 239.93) | 0.01 (-0.03, 0.04) | -0.11 (-0.13, -0.08) | 0.09 (-0.02, 0.19) | -0.25 (-0.47, -0.02) |
| Comoros | 2.47 (1.83, 3.21) | 3.35 (2.52, 4.34) | 35.41 | 581.57 (442.19, 741.30) | 509.54 (390.08, 653.01) | 428.49 (325.04, 547.89) | 449.28 (342.43, 582.51) | -1.22 (-1.40, -1.05) | -1.31 (-1.62, -1.00) | -1.80 (-1.87, -1.72) | 0.53 (0.27, 0.78) |
| Congo | 24.28 (18.06, 32.30) | 55.12 (42.55, 69.41) | 127.04 | 1076.27 (807.83, 1395.99) | 1048.83 (799.59, 1356.69) | 1014.06 (792.32, 1278.33) | 1026.97 (790.87, 1292.81) | -0.22 (-0.25, -0.18) | -0.25 (-0.33, -0.18) | -0.35 (-0.38, -0.33) | 0.15 (0.12, 0.18) |
| Cook Islands | 0.06 (0.04, 0.08) | 0.05 (0.04, 0.06) | -15.79 | 300.94 (223.28, 397.82) | 295.61 (217.95, 393.63) | 292.03 (214.95, 386.59) | 296.94 (217.94, 394.89) | -0.07 (-0.09, -0.04) | -0.17 (-0.22, -0.11) | -0.12 (-0.13, -0.12) | 0.20 (0.11, 0.29) |
| Costa Rica | 3.59 (2.63, 4.81) | 5.84 (4.39, 7.72) | 62.67 | 115.56 (87.36, 152.74) | 114.11 (86.94, 150.03) | 114.46 (86.12, 150.86) | 115.09 (86.43, 152.28) | -0.02 (-0.03, -0.01) | -0.13 (-0.13, -0.12) | 0.03 (0.00, 0.07) | 0.07 (-0.02, 0.15) |
| Croatia | 2.02 (1.56, 2.61) | 1.58 (1.21, 2.05) | -21.99 | 39.54 (30.33, 51.29) | 39.23 (30.00, 51.70) | 38.99 (29.59, 50.94) | 39.40 (30.06, 51.35) | -0.01 (-0.03, 0.01) | -0.07 (-0.11, -0.04) | -0.06 (-0.07, -0.06) | 0.16 (0.10, 0.22) |
| Cuba | 16.48 (12.05, 22.19) | 14.27 (11.17, 18.35) | -13.42 | 134.56 (100.98, 178.03) | 144.16 (109.08, 186.68) | 122.46 (93.47, 159.03) | 128.18 (98.91, 166.96) | -0.40 (-0.64, -0.16) | 0.73 (0.32, 1.15) | -1.67 (-2.51, -0.83) | 0.42 (0.23, 0.61) |
| Cyprus | 0.55 (0.41, 0.73) | 0.91 (0.67, 1.22) | 66.70 | 67.30 (50.41, 89.07) | 66.96 (49.49, 89.74) | 65.89 (48.54, 87.90) | 65.54 (48.02, 87.73) | -0.12 (-0.14, -0.11) | -0.07 (-0.13, 0.00) | -0.17 (-0.24, -0.09) | -0.04 (-0.06, -0.02) |
| Czechia | 4.12 (3.19, 5.38) | 3.97 (3.04, 5.23) | -3.45 | 39.45 (30.38, 51.67) | 39.34 (30.18, 51.35) | 39.36 (29.81, 51.56) | 39.67 (30.05, 52.16) | 0.02 (0.01, 0.04) | -0.02 (-0.04, -0.01) | 0.00 (-0.02, 0.02) | 0.12 (0.07, 0.17) |
| Côte d'Ivoire | 49.20 (36.91, 63.28) | 99.26 (71.83, 131.11) | 101.77 | 419.99 (323.17, 536.33) | 366.63 (291.58, 456.34) | 367.48 (272.57, 481.86) | 373.96 (276.50, 492.66) | -0.23 (-0.37, -0.09) | -1.26 (-1.60, -0.92) | 0.07 (-0.18, 0.32) | 0.25 (0.13, 0.37) |
| Democratic People's Republic of Korea | 19.12 (14.23, 25.39) | 25.62 (19.20, 33.55) | 33.96 | 88.19 (65.99, 117.79) | 88.85 (66.68, 118.05) | 89.00 (66.43, 117.38) | 90.45 (67.52, 119.03) | 0.07 (0.06, 0.08) | 0.06 (0.05, 0.07) | 0.01 (-0.03, 0.06) | 0.18 (0.17, 0.18) |
| Democratic Republic of the Congo | 393.17 (295.15, 504.55) | 865.11 (660.10, 1114.71) | 120.03 | 1130.08 (865.77, 1450.43) | 1148.63 (861.88, 1472.44) | 1109.38 (909.32, 1334.90) | 1017.04 (784.79, 1299.03) | -0.54 (-0.67, -0.40) | 0.08 (-0.35, 0.50) | -0.36 (-0.81, 0.09) | -1.07 (-1.33, -0.82) |
| Denmark | 3.37 (2.48, 4.50) | 3.27 (2.50, 4.26) | -3.10 | 63.37 (46.52, 84.58) | 63.13 (46.97, 85.23) | 60.44 (44.60, 79.94) | 61.87 (46.08, 81.38) | -0.19 (-0.23, -0.15) | -0.05 (-0.09, 0.00) | -0.45 (-0.60, -0.29) | 0.22 (0.16, 0.29) |
| Djibouti | 1.05 (0.77, 1.38) | 2.70 (2.03, 3.54) | 156.29 | 223.01 (170.47, 288.59) | 216.06 (169.96, 268.74) | 211.28 (161.78, 270.73) | 211.38 (161.96, 277.25) | -0.06 (-0.18, 0.05) | -0.29 (-0.68, 0.10) | -0.26 (-0.83, 0.31) | 0.13 (-0.03, 0.30) |
| Dominica | 0.16 (0.12, 0.21) | 0.14 (0.11, 0.19) | -8.40 | 215.16 (160.17, 286.73) | 211.76 (157.87, 278.22) | 217.21 (162.94, 286.63) | 207.79 (155.37, 276.13) | 0.00 (-0.04, 0.05) | -0.16 (-0.21, -0.11) | 0.28 (0.24, 0.32) | -0.43 (-0.62, -0.24) |
| Dominican Republic | 13.37 (9.74, 17.92) | 23.25 (18.16, 29.31) | 73.91 | 179.04 (134.21, 235.23) | 170.93 (129.00, 225.72) | 177.64 (138.28, 224.66) | 200.23 (157.74, 250.41) | 0.14 (0.01, 0.27) | -0.49 (-0.69, -0.29) | 0.41 (0.26, 0.55) | 0.79 (0.09, 1.50) |
| Ecuador | 22.54 (16.60, 30.40) | 41.56 (30.88, 54.91) | 84.36 | 225.96 (170.79, 298.92) | 222.40 (169.70, 291.78) | 223.49 (167.90, 295.70) | 224.85 (168.01, 295.05) | -0.02 (-0.04, 0.00) | -0.17 (-0.22, -0.12) | 0.05 (-0.04, 0.14) | 0.09 (0.06, 0.12) |
| Egypt | 28.63 (21.25, 37.70) | 55.97 (41.39, 74.45) | 95.53 | 53.36 (40.26, 70.17) | 49.94 (39.19, 64.70) | 51.30 (38.68, 67.55) | 54.05 (40.12, 71.04) | 0.21 (0.12, 0.31) | -0.59 (-0.90, -0.27) | 0.26 (0.20, 0.33) | 0.64 (0.50, 0.78) |
| El Salvador | 4.86 (3.61, 6.44) | 6.01 (4.44, 7.99) | 23.81 | 96.62 (73.04, 126.78) | 93.99 (71.02, 124.64) | 88.91 (67.54, 117.11) | 90.82 (68.28, 118.45) | -0.31 (-0.36, -0.26) | -0.28 (-0.29, -0.27) | -0.57 (-0.81, -0.33) | 0.22 (0.15, 0.29) |
| Equatorial Guinea | 4.43 (3.33, 5.75) | 19.62 (14.73, 24.71) | 343.02 | 1191.36 (905.65, 1521.90) | 1134.33 (858.26, 1467.67) | 1165.55 (900.61, 1475.35) | 1241.57 (954.50, 1539.81) | 0.09 (-0.05, 0.22) | -0.50 (-0.66, -0.34) | 0.29 (-0.07, 0.65) | 0.64 (0.54, 0.73) |
| Eritrea | 13.13 (9.78, 17.16) | 27.81 (20.82, 37.01) | 111.77 | 485.35 (365.19, 629.31) | 472.59 (377.38, 595.87) | 426.18 (334.94, 539.72) | 409.50 (307.34, 543.34) | -0.67 (-0.74, -0.60) | -0.20 (-0.32, -0.08) | -1.05 (-1.14, -0.96) | -0.37 (-0.59, -0.15) |
| Estonia | 0.77 (0.60, 1.01) | 0.59 (0.46, 0.77) | -23.61 | 47.98 (36.99, 63.11) | 45.08 (34.90, 58.91) | 45.41 (34.91, 59.28) | 46.64 (35.75, 61.25) | -0.12 (-0.20, -0.05) | -0.68 (-0.83, -0.53) | 0.07 (0.02, 0.12) | 0.31 (0.23, 0.38) |
| Eswatini | 5.36 (3.81, 7.01) | 5.86 (4.39, 7.93) | 9.44 | 695.22 (517.85, 880.24) | 544.49 (438.96, 667.38) | 501.50 (395.37, 626.94) | 476.87 (363.27, 633.03) | -1.12 (-1.32, -0.93) | -2.46 (-2.56, -2.35) | -0.78 (-0.89, -0.67) | -0.29 (-0.69, 0.11) |
| Ethiopia | 217.58 (154.23, 296.76) | 241.27 (171.40, 328.76) | 10.89 | 455.01 (328.38, 614.72) | 323.18 (242.78, 426.01) | 220.56 (163.79, 294.64) | 230.48 (170.01, 308.55) | -2.67 (-2.98, -2.36) | -3.14 (-3.94, -2.33) | -3.93 (-4.76, -3.10) | -0.32 (-1.34, 0.71) |
| Fiji | 2.51 (1.84, 3.35) | 2.92 (2.17, 3.84) | 16.30 | 310.06 (229.47, 411.13) | 316.56 (237.00, 410.17) | 313.49 (236.32, 413.59) | 309.10 (229.14, 406.37) | -0.05 (-0.10, 0.00) | 0.22 (0.16, 0.29) | -0.10 (-0.16, -0.04) | -0.20 (-0.38, -0.02) |
| Finland | 3.47 (2.58, 4.64) | 3.20 (2.40, 4.24) | -7.64 | 67.30 (49.75, 90.06) | 66.67 (50.02, 89.64) | 66.19 (48.80, 88.69) | 66.52 (48.70, 89.15) | -0.05 (-0.06, -0.03) | -0.09 (-0.11, -0.08) | -0.07 (-0.09, -0.06) | 0.08 (0.05, 0.10) |
| France | 39.20 (29.34, 52.90) | 37.60 (28.23, 48.70) | -4.07 | 66.54 (49.70, 89.88) | 66.46 (49.10, 89.23) | 65.32 (48.45, 88.20) | 65.46 (48.04, 86.05) | -0.09 (-0.11, -0.07) | -0.01 (-0.04, 0.01) | -0.18 (-0.24, -0.11) | 0.03 (0.02, 0.03) |
| Gabon | 10.41 (7.88, 13.41) | 18.59 (13.73, 24.17) | 78.66 | 1156.94 (894.51, 1481.55) | 1197.77 (958.70, 1498.51) | 976.94 (746.57, 1275.49) | 997.35 (744.01, 1290.40) | -0.97 (-1.14, -0.80) | 0.26 (-0.02, 0.55) | -2.17 (-2.35, -2.00) | 0.19 (0.03, 0.35) |
| Gambia | 3.49 (2.52, 4.61) | 8.30 (5.97, 11.20) | 137.99 | 370.83 (278.29, 484.34) | 385.44 (299.45, 482.92) | 351.07 (262.68, 463.55) | 356.74 (264.63, 472.28) | -0.40 (-0.53, -0.27) | 0.33 (0.06, 0.60) | -0.97 (-1.49, -0.45) | 0.25 (0.12, 0.38) |
| Georgia | 3.22 (2.44, 4.21) | 1.89 (1.48, 2.42) | -41.13 | 57.12 (43.15, 75.45) | 64.38 (48.82, 84.11) | 54.55 (43.16, 68.34) | 55.06 (42.90, 70.63) | -0.62 (-0.82, -0.43) | 1.04 (0.46, 1.63) | -1.76 (-2.22, -1.29) | 0.12 (0.08, 0.17) |
| Germany | 54.76 (40.71, 71.76) | 48.71 (36.81, 65.51) | -11.05 | 65.59 (48.81, 86.43) | 64.92 (47.64, 86.49) | 63.42 (46.93, 84.08) | 64.73 (47.90, 87.94) | -0.12 (-0.15, -0.09) | -0.12 (-0.19, -0.05) | -0.24 (-0.27, -0.21) | 0.27 (0.20, 0.33) |
| Ghana | 58.58 (42.71, 77.11) | 133.75 (102.79, 170.19) | 128.31 | 418.31 (312.64, 549.01) | 423.27 (319.43, 543.74) | 397.42 (304.69, 497.51) | 395.38 (305.77, 504.74) | -0.29 (-0.35, -0.24) | 0.14 (0.12, 0.16) | -0.67 (-0.75, -0.60) | -0.05 (-0.07, -0.02) |
| Greece | 4.48 (3.84, 5.18) | 5.92 (4.47, 7.75) | 32.31 | 43.58 (37.27, 50.45) | 45.82 (34.54, 60.66) | 66.88 (49.09, 89.90) | 66.64 (49.01, 88.32) | 3.38 (2.50, 4.26) | 1.07 (-3.44, 5.80) | 3.46 (1.70, 5.26) | -0.07 (-0.10, -0.03) |
| Greenland | 0.04 (0.03, 0.05) | 0.03 (0.03, 0.04) | -18.25 | 60.06 (45.24, 80.05) | 58.51 (44.12, 77.53) | 57.28 (42.89, 76.48) | 57.04 (42.92, 74.54) | -0.17 (-0.18, -0.15) | -0.26 (-0.30, -0.21) | -0.24 (-0.29, -0.19) | -0.04 (-0.09, 0.01) |
| Grenada | 0.16 (0.12, 0.22) | 0.23 (0.18, 0.30) | 42.96 | 198.20 (148.08, 264.15) | 195.94 (147.80, 255.99) | 213.92 (163.50, 273.60) | 208.70 (159.66, 269.70) | 0.32 (0.23, 0.41) | -0.13 (-0.21, -0.05) | 0.93 (0.73, 1.14) | -0.37 (-0.54, -0.20) |
| Guam | 0.49 (0.36, 0.66) | 0.51 (0.38, 0.68) | 4.10 | 311.52 (229.47, 413.43) | 304.71 (227.62, 403.99) | 304.36 (225.41, 404.56) | 311.32 (231.47, 416.33) | -0.02 (-0.05, 0.02) | -0.22 (-0.25, -0.18) | -0.01 (-0.03, 0.01) | 0.24 (0.16, 0.31) |
| Guatemala | 7.66 (5.71, 10.07) | 20.33 (15.08, 26.67) | 165.63 | 108.97 (82.73, 142.16) | 105.95 (80.86, 137.83) | 101.14 (77.32, 131.28) | 105.49 (80.53, 137.87) | -0.28 (-0.33, -0.22) | -0.30 (-0.39, -0.21) | -0.48 (-0.63, -0.33) | 0.36 (0.15, 0.57) |
| Guinea | 24.15 (17.64, 31.21) | 48.11 (36.09, 62.50) | 99.25 | 447.22 (333.64, 574.11) | 468.55 (358.54, 604.13) | 405.44 (303.03, 523.61) | 408.14 (309.71, 529.10) | -0.46 (-0.61, -0.30) | 0.53 (0.21, 0.85) | -1.54 (-1.87, -1.21) | 0.15 (0.07, 0.22) |
| Guinea-Bissau | 5.61 (4.07, 7.38) | 9.32 (6.81, 12.65) | 66.12 | 592.04 (435.22, 760.01) | 698.36 (535.47, 899.58) | 517.82 (387.76, 665.09) | 474.29 (349.55, 634.60) | -1.13 (-1.54, -0.73) | 1.76 (1.28, 2.25) | -3.00 (-3.77, -2.22) | -0.90 (-1.15, -0.65) |
| Guyana | 1.29 (0.94, 1.70) | 1.30 (0.98, 1.70) | 0.97 | 158.11 (118.51, 206.30) | 158.20 (120.55, 204.13) | 158.91 (120.99, 204.81) | 154.38 (117.46, 200.76) | -0.08 (-0.12, -0.04) | -0.02 (-0.16, 0.11) | 0.05 (-0.11, 0.21) | -0.36 (-0.47, -0.26) |
| Haiti | 14.04 (10.30, 18.32) | 34.65 (27.93, 42.43) | 146.70 | 229.94 (173.08, 297.05) | 251.60 (191.84, 323.12) | 273.23 (224.89, 346.24) | 256.53 (206.97, 312.83) | 0.39 (0.16, 0.62) | 0.75 (0.12, 1.39) | 0.85 (-0.39, 2.11) | -0.62 (-1.10, -0.15) |
| Honduras | 4.06 (3.00, 5.38) | 9.39 (7.11, 12.47) | 131.31 | 98.79 (74.84, 129.90) | 97.72 (74.40, 127.92) | 89.10 (69.81, 116.16) | 91.02 (69.95, 119.83) | -0.48 (-0.57, -0.39) | -0.12 (-0.15, -0.08) | -0.94 (-1.34, -0.54) | 0.13 (-0.08, 0.33) |
| Hungary | 4.19 (3.24, 5.45) | 3.72 (2.83, 4.86) | -11.07 | 40.11 (30.80, 52.45) | 40.13 (30.48, 52.97) | 40.28 (30.72, 53.19) | 40.16 (30.77, 52.22) | 0.02 (0.01, 0.02) | 0.00 (-0.03, 0.03) | 0.04 (0.03, 0.05) | -0.01 (-0.07, 0.04) |
| Iceland | 0.18 (0.13, 0.24) | 0.22 (0.17, 0.30) | 23.11 | 68.06 (50.55, 90.54) | 67.17 (49.76, 90.05) | 66.80 (49.33, 89.26) | 66.99 (49.96, 89.04) | -0.08 (-0.10, -0.06) | -0.14 (-0.16, -0.12) | 0.03 (-0.05, 0.10) | 0.07 (-0.04, 0.17) |
| India | 1792.14 (1299.51, 2379.41) | 2729.03 (2009.27, 3626.66) | 52.28 | 212.13 (155.34, 280.75) | 225.79 (165.59, 298.64) | 177.94 (131.33, 234.42) | 179.56 (132.72, 237.45) | -0.86 (-1.17, -0.56) | 0.75 (0.17, 1.34) | -2.38 (-3.30, -1.45) | 0.07 (-0.32, 0.46) |
| Indonesia | 169.85 (122.90, 226.07) | 242.16 (179.06, 326.25) | 42.57 | 89.27 (65.83, 118.61) | 88.90 (65.38, 118.77) | 86.45 (63.44, 115.44) | 85.37 (62.83, 114.79) | -0.20 (-0.22, -0.18) | -0.06 (-0.17, 0.05) | -0.29 (-0.31, -0.26) | -0.05 (-0.15, 0.06) |
| Iran (Islamic Republic of) | 22.79 (16.10, 31.20) | 44.32 (32.52, 61.43) | 94.49 | 43.30 (31.57, 58.72) | 40.84 (29.95, 55.07) | 42.10 (31.02, 56.62) | 45.90 (33.72, 61.88) | 0.21 (0.07, 0.36) | -0.60 (-0.77, -0.44) | 0.30 (0.24, 0.36) | 1.07 (0.87, 1.27) |
| Iraq | 12.94 (9.43, 17.43) | 38.30 (27.75, 50.81) | 195.87 | 83.46 (62.00, 111.02) | 83.96 (62.74, 111.08) | 82.74 (61.51, 111.38) | 82.57 (61.04, 108.90) | -0.06 (-0.08, -0.04) | 0.04 (0.02, 0.07) | -0.18 (-0.23, -0.12) | -0.04 (-0.08, 0.00) |
| Ireland | 2.34 (1.74, 3.12) | 3.00 (2.24, 4.03) | 28.14 | 66.00 (48.94, 88.07) | 66.02 (48.83, 88.04) | 64.85 (47.81, 87.24) | 65.11 (47.88, 87.66) | -0.09 (-0.11, -0.06) | 0.00 (-0.01, 0.01) | -0.16 (-0.28, -0.05) | 0.04 (0.00, 0.08) |
| Israel | 3.16 (2.31, 4.24) | 5.69 (4.24, 7.63) | 80.04 | 65.00 (47.92, 86.44) | 65.18 (48.21, 88.83) | 64.44 (48.03, 87.12) | 65.21 (48.13, 88.10) | -0.02 (-0.04, -0.01) | 0.02 (-0.03, 0.07) | -0.12 (-0.17, -0.07) | 0.16 (0.13, 0.19) |
| Italy | 42.79 (31.68, 56.46) | 36.04 (27.52, 47.37) | -15.78 | 73.12 (54.11, 96.57) | 72.15 (53.53, 95.26) | 69.85 (51.81, 93.77) | 70.49 (52.14, 92.97) | -0.21 (-0.24, -0.18) | -0.14 (-0.22, -0.07) | -0.33 (-0.35, -0.30) | 0.14 (0.01, 0.28) |
| Jamaica | 4.41 (3.18, 5.91) | 5.56 (4.28, 7.01) | 26.11 | 182.38 (135.54, 240.93) | 180.80 (136.32, 238.73) | 206.46 (161.45, 260.88) | 179.42 (139.74, 224.69) | 0.49 (0.31, 0.67) | -0.10 (-0.13, -0.06) | 1.39 (0.94, 1.85) | -1.08 (-2.01, -0.14) |
| Japan | 105.99 (80.63, 140.93) | 86.80 (65.87, 114.76) | -18.10 | 81.68 (61.15, 109.39) | 79.33 (59.26, 105.82) | 79.40 (59.47, 106.32) | 81.83 (61.32, 109.33) | 0.03 (-0.02, 0.08) | -0.27 (-0.38, -0.15) | 0.01 (0.01, 0.01) | 0.41 (0.28, 0.53) |
| Jordan | 3.65 (2.60, 5.01) | 12.87 (9.46, 17.14) | 252.21 | 101.46 (74.67, 134.95) | 101.55 (74.59, 137.06) | 100.40 (73.50, 133.65) | 101.68 (75.52, 134.61) | -0.02 (-0.04, 0.00) | 0.01 (-0.02, 0.04) | -0.11 (-0.15, -0.07) | 0.14 (0.09, 0.19) |
| Kazakhstan | 8.65 (6.49, 11.50) | 9.21 (7.10, 11.93) | 6.48 | 51.27 (39.07, 67.41) | 52.00 (39.42, 68.24) | 48.56 (37.78, 60.96) | 48.52 (37.47, 62.57) | -0.29 (-0.38, -0.21) | 0.12 (0.08, 0.15) | -0.72 (-1.06, -0.38) | 0.00 (-0.04, 0.04) |
| Kenya | 106.89 (78.13, 142.35) | 191.61 (140.94, 253.60) | 79.25 | 510.66 (379.39, 673.10) | 451.37 (338.23, 588.81) | 321.86 (243.84, 414.93) | 374.83 (282.67, 492.33) | -1.60 (-1.97, -1.23) | -1.18 (-1.31, -1.05) | -3.50 (-4.17, -2.83) | 2.12 (1.46, 2.78) |
| Kiribati | 0.27 (0.20, 0.36) | 0.46 (0.35, 0.59) | 67.72 | 357.29 (262.98, 468.05) | 350.98 (263.84, 457.63) | 359.96 (275.01, 457.08) | 367.36 (278.20, 468.80) | 0.11 (0.06, 0.17) | -0.18 (-0.22, -0.14) | 0.26 (0.11, 0.40) | 0.17 (0.07, 0.26) |
| Kuwait | 1.97 (1.41, 2.69) | 4.87 (3.58, 6.64) | 147.25 | 91.16 (67.49, 121.28) | 92.54 (67.64, 123.22) | 88.23 (66.05, 116.90) | 84.24 (62.24, 111.54) | -0.29 (-0.37, -0.20) | 0.19 (0.07, 0.32) | -0.48 (-0.62, -0.34) | -0.50 (-0.56, -0.43) |
| Kyrgyzstan | 2.00 (1.49, 2.66) | 2.90 (2.20, 3.83) | 45.32 | 46.91 (35.55, 61.78) | 48.04 (36.47, 63.04) | 42.29 (32.50, 55.02) | 42.59 (32.66, 56.00) | -0.56 (-0.69, -0.43) | 0.23 (0.19, 0.27) | -1.31 (-1.77, -0.86) | 0.08 (-0.01, 0.17) |
| Lao People's Democratic Republic | 2.85 (2.09, 3.82) | 6.10 (4.43, 8.19) | 114.34 | 75.60 (56.06, 101.36) | 76.23 (56.84, 100.03) | 77.53 (57.55, 102.50) | 77.19 (57.08, 103.06) | 0.13 (0.12, 0.15) | 0.09 (0.04, 0.14) | 0.17 (0.14, 0.21) | 0.08 (-0.07, 0.24) |
| Latvia | 1.36 (1.07, 1.73) | 0.84 (0.65, 1.10) | -38.34 | 49.25 (38.49, 63.04) | 48.28 (37.21, 62.28) | 44.94 (34.64, 58.48) | 45.99 (35.56, 59.97) | -0.36 (-0.44, -0.29) | -0.20 (-0.23, -0.18) | -0.75 (-0.97, -0.53) | 0.24 (0.20, 0.28) |
| Lebanon | 2.55 (1.85, 3.43) | 4.62 (3.39, 6.15) | 81.15 | 83.84 (61.30, 112.72) | 83.91 (61.94, 113.08) | 82.98 (60.68, 111.39) | 84.54 (62.36, 113.05) | 0.01 (-0.02, 0.03) | 0.01 (-0.01, 0.03) | -0.12 (-0.12, -0.11) | 0.19 (0.11, 0.28) |
| Lesotho | 8.55 (6.28, 11.42) | 9.05 (6.71, 12.31) | 5.85 | 488.89 (368.00, 630.76) | 450.02 (345.52, 573.45) | 422.07 (328.68, 532.16) | 387.46 (291.29, 521.42) | -0.63 (-0.82, -0.44) | -0.80 (-0.95, -0.65) | -0.62 (-1.46, 0.22) | -0.55 (-1.23, 0.13) |
| Liberia | 10.91 (8.16, 14.54) | 34.10 (25.95, 43.12) | 212.48 | 640.21 (477.03, 851.89) | 624.56 (460.40, 817.62) | 661.84 (521.74, 867.85) | 650.19 (500.71, 822.06) | 0.12 (-0.03, 0.26) | -0.29 (-0.57, -0.01) | 0.60 (-0.03, 1.23) | -0.12 (-0.29, 0.05) |
| Libya | 3.37 (2.43, 4.52) | 6.83 (5.09, 9.13) | 102.82 | 87.51 (64.18, 115.95) | 84.67 (62.54, 113.18) | 84.52 (63.38, 111.78) | 84.35 (62.82, 111.89) | -0.09 (-0.12, -0.06) | -0.35 (-0.39, -0.31) | 0.00 (-0.02, 0.02) | -0.03 (-0.05, 0.00) |
| Lithuania | 1.77 (1.35, 2.29) | 1.22 (0.96, 1.57) | -30.76 | 46.15 (35.62, 60.41) | 44.90 (34.38, 57.59) | 44.21 (34.26, 57.40) | 44.90 (35.13, 57.54) | -0.14 (-0.17, -0.11) | -0.30 (-0.38, -0.22) | -0.16 (-0.30, -0.02) | 0.16 (0.13, 0.19) |
| Luxembourg | 0.29 (0.21, 0.38) | 0.42 (0.31, 0.56) | 45.73 | 70.16 (52.06, 93.68) | 68.36 (50.88, 92.00) | 66.55 (49.69, 87.95) | 66.95 (49.91, 89.20) | -0.23 (-0.27, -0.19) | -0.28 (-0.42, -0.14) | -0.27 (-0.29, -0.24) | 0.08 (0.06, 0.10) |
| Madagascar | 90.29 (67.84, 116.24) | 194.73 (154.23, 241.74) | 115.68 | 809.63 (622.47, 1026.49) | 834.05 (673.34, 1028.79) | 724.47 (571.18, 910.57) | 717.43 (574.29, 895.14) | -0.88 (-1.07, -0.69) | 0.08 (-0.93, 1.10) | -1.44 (-1.65, -1.22) | 0.14 (-0.15, 0.42) |
| Malawi | 57.16 (46.40, 69.12) | 105.88 (81.75, 135.30) | 85.24 | 646.69 (533.85, 782.47) | 753.38 (623.98, 912.56) | 610.64 (480.46, 773.88) | 592.24 (461.41, 755.16) | -0.82 (-1.07, -0.57) | 1.44 (0.98, 1.90) | -2.19 (-3.10, -1.27) | -0.15 (-0.44, 0.15) |
| Malaysia | 12.73 (9.26, 17.17) | 24.88 (18.11, 33.36) | 95.46 | 69.91 (51.42, 93.57) | 70.07 (51.69, 93.82) | 67.71 (50.64, 90.00) | 69.67 (51.56, 92.81) | -0.07 (-0.12, -0.01) | 0.02 (0.01, 0.04) | -0.36 (-0.51, -0.20) | 0.48 (0.29, 0.68) |
| Maldives | 0.14 (0.10, 0.19) | 0.58 (0.41, 0.82) | 309.04 | 74.32 (55.64, 99.04) | 72.62 (54.02, 97.18) | 74.77 (55.32, 98.49) | 85.37 (62.76, 114.95) | 0.46 (0.29, 0.64) | -0.22 (-0.23, -0.20) | 0.24 (-0.04, 0.52) | 1.64 (1.44, 1.84) |
| Mali | 36.80 (27.02, 47.65) | 101.09 (74.29, 131.13) | 174.66 | 489.49 (362.31, 630.60) | 471.96 (362.91, 595.81) | 501.74 (389.78, 624.27) | 495.85 (371.41, 635.47) | 0.26 (0.18, 0.33) | -0.31 (-0.55, -0.06) | 0.63 (0.40, 0.87) | -0.08 (-0.17, 0.00) |
| Malta | 0.25 (0.18, 0.33) | 0.26 (0.20, 0.35) | 6.65 | 64.99 (48.20, 86.98) | 65.24 (48.27, 86.98) | 64.42 (47.58, 85.89) | 65.56 (48.70, 88.05) | -0.01 (-0.02, 0.01) | 0.04 (0.04, 0.04) | -0.13 (-0.19, -0.07) | 0.18 (0.15, 0.21) |
| Marshall Islands | 0.12 (0.09, 0.17) | 0.19 (0.14, 0.25) | 52.41 | 291.93 (217.30, 389.06) | 263.05 (199.01, 347.44) | 297.36 (233.29, 366.49) | 309.28 (232.03, 407.96) | 0.42 (-0.08, 0.93) | -1.01 (-1.40, -0.61) | 1.24 (-0.82, 3.34) | 0.12 (-0.88, 1.13) |
| Mauritania | 9.88 (7.25, 12.95) | 18.89 (13.80, 25.15) | 91.25 | 519.76 (379.93, 679.84) | 501.03 (374.26, 654.67) | 489.76 (373.04, 626.44) | 472.46 (349.68, 616.87) | -0.29 (-0.37, -0.21) | -0.38 (-0.57, -0.18) | -0.22 (-0.47, 0.03) | -0.22 (-0.51, 0.08) |
| Mauritius | 1.20 (0.86, 1.64) | 1.36 (1.01, 1.78) | 13.24 | 95.68 (70.67, 129.15) | 95.05 (69.95, 125.92) | 103.30 (76.78, 133.32) | 104.79 (77.14, 138.22) | 0.41 (0.31, 0.50) | -0.07 (-0.09, -0.05) | 0.86 (0.45, 1.28) | 0.20 (0.11, 0.28) |
| Mexico | 70.80 (51.82, 94.38) | 94.67 (71.39, 125.48) | 33.71 | 82.94 (62.14, 110.12) | 79.03 (59.22, 104.80) | 66.66 (50.24, 88.62) | 71.27 (53.86, 94.54) | -0.83 (-1.02, -0.63) | -0.47 (-0.51, -0.43) | -1.80 (-2.82, -0.76) | 0.68 (0.40, 0.96) |
| Micronesia (Federated States of) | 0.35 (0.26, 0.47) | 0.41 (0.31, 0.53) | 17.74 | 353.74 (259.62, 469.53) | 349.68 (258.62, 461.46) | 385.02 (293.75, 493.05) | 373.75 (282.16, 488.38) | 0.41 (0.30, 0.52) | -0.11 (-0.14, -0.09) | 1.00 (0.56, 1.44) | -0.28 (-0.49, -0.08) |
| Monaco | 0.02 (0.01, 0.02) | 0.02 (0.01, 0.03) | 5.87 | 65.81 (48.22, 87.95) | 66.51 (49.53, 89.56) | 65.45 (48.45, 87.65) | 65.84 (48.83, 88.59) | -0.07 (-0.09, -0.05) | 0.11 (0.07, 0.14) | -0.17 (-0.18, -0.15) | 0.02 (-0.08, 0.11) |
| Mongolia | 1.89 (1.38, 2.56) | 4.08 (3.27, 5.05) | 115.40 | 86.91 (64.91, 115.77) | 88.06 (65.98, 116.07) | 119.80 (96.15, 153.91) | 124.71 (100.26, 153.70) | 1.55 (1.25, 1.86) | 0.13 (0.11, 0.15) | 3.29 (1.88, 4.73) | 0.20 (-0.10, 0.51) |
| Montenegro | 0.26 (0.20, 0.34) | 0.25 (0.19, 0.32) | -5.52 | 39.93 (30.27, 52.63) | 39.67 (30.49, 51.98) | 39.35 (30.07, 51.00) | 39.82 (30.45, 51.90) | -0.02 (-0.04, 0.00) | -0.07 (-0.07, -0.06) | -0.08 (-0.09, -0.07) | 0.18 (0.12, 0.24) |
| Morocco | 47.24 (33.65, 62.82) | 69.80 (50.80, 92.26) | 47.74 | 183.82 (132.44, 242.11) | 188.85 (145.72, 240.60) | 225.01 (172.27, 285.88) | 181.17 (131.36, 239.01) | 0.31 (-0.08, 0.70) | -0.09 (-1.72, 1.57) | 1.82 (0.80, 2.85) | -1.42 (-3.12, 0.30) |
| Mozambique | 148.49 (116.30, 187.02) | 214.34 (164.03, 268.39) | 44.34 | 1201.03 (942.88, 1524.64) | 1015.86 (786.88, 1287.45) | 845.09 (665.68, 1059.28) | 779.20 (600.81, 969.32) | -1.55 (-1.73, -1.37) | -1.68 (-2.36, -1.00) | -1.89 (-1.99, -1.80) | -0.45 (-1.20, 0.30) |
| Myanmar | 85.18 (60.78, 114.75) | 121.61 (89.94, 159.01) | 42.76 | 203.26 (144.96, 270.78) | 205.50 (147.34, 274.91) | 236.87 (179.81, 301.61) | 209.69 (155.25, 274.47) | 0.26 (0.05, 0.47) | 0.08 (-0.04, 0.19) | 1.49 (1.11, 1.87) | -1.45 (-1.98, -0.93) |
| Namibia | 7.29 (5.40, 9.51) | 12.48 (9.25, 16.90) | 71.21 | 541.23 (409.03, 695.62) | 591.60 (451.64, 746.72) | 429.72 (326.50, 568.36) | 489.73 (366.10, 659.31) | -1.07 (-1.43, -0.72) | 0.94 (0.57, 1.32) | -3.27 (-4.25, -2.28) | 1.00 (0.16, 1.86) |
| Nauru | 0.03 (0.02, 0.04) | 0.03 (0.03, 0.05) | 12.94 | 304.88 (225.98, 405.07) | 301.58 (223.35, 398.00) | 300.97 (223.51, 403.85) | 304.85 (223.23, 404.66) | -0.01 (-0.03, 0.00) | -0.10 (-0.15, -0.04) | -0.02 (-0.03, -0.01) | 0.11 (0.03, 0.19) |
| Nepal | 43.53 (31.85, 57.45) | 75.05 (54.55, 100.81) | 72.41 | 239.30 (178.62, 313.19) | 237.27 (176.06, 313.49) | 227.25 (170.21, 298.74) | 227.53 (168.51, 304.29) | -0.24 (-0.27, -0.21) | -0.09 (-0.09, -0.09) | -0.46 (-0.49, -0.44) | 0.04 (-0.02, 0.10) |
| Netherlands | 11.00 (8.13, 14.62) | 10.20 (7.77, 13.57) | -7.31 | 67.55 (50.00, 89.05) | 67.16 (49.99, 89.52) | 65.38 (48.83, 86.64) | 66.32 (49.55, 89.61) | -0.14 (-0.16, -0.11) | -0.07 (-0.13, 0.00) | -0.28 (-0.30, -0.26) | 0.14 (0.11, 0.17) |
| New Zealand | 2.47 (1.82, 3.31) | 2.72 (2.05, 3.58) | 10.22 | 68.54 (50.55, 91.36) | 66.97 (49.97, 88.93) | 65.94 (48.67, 88.49) | 67.05 (49.59, 89.79) | -0.11 (-0.15, -0.08) | -0.23 (-0.25, -0.20) | -0.15 (-0.20, -0.09) | 0.25 (0.06, 0.44) |
| Nicaragua | 3.58 (2.63, 4.80) | 6.46 (4.88, 8.40) | 80.26 | 102.89 (78.18, 135.96) | 106.23 (79.97, 137.26) | 89.11 (69.92, 113.64) | 92.17 (70.92, 118.73) | -0.63 (-0.83, -0.43) | 0.34 (0.15, 0.52) | -1.79 (-2.57, -1.00) | 0.35 (0.26, 0.44) |
| Niger | 20.82 (15.24, 27.47) | 55.34 (40.22, 73.07) | 165.85 | 313.59 (233.61, 405.41) | 310.79 (234.01, 403.45) | 294.86 (220.97, 382.37) | 294.08 (219.33, 390.15) | -0.32 (-0.36, -0.27) | -0.10 (-0.24, 0.04) | -0.56 (-0.72, -0.40) | -0.05 (-0.14, 0.04) |
| Nigeria | 382.16 (278.08, 500.82) | 873.48 (633.45, 1165.57) | 128.56 | 465.28 (344.41, 609.32) | 430.26 (322.38, 562.49) | 428.89 (319.30, 564.41) | 430.52 (319.48, 565.75) | -0.19 (-0.29, -0.10) | -0.82 (-0.91, -0.73) | -0.03 (-0.08, 0.03) | 0.13 (-0.10, 0.36) |
| Niue | 0.01 (0.00, 0.01) | 0.00 (0.00, 0.01) | -25.62 | 299.90 (219.76, 398.62) | 297.94 (221.83, 394.63) | 298.47 (220.54, 399.52) | 301.66 (222.19, 401.23) | 0.01 (-0.01, 0.02) | -0.05 (-0.10, -0.01) | 0.02 (0.01, 0.04) | 0.11 (-0.02, 0.24) |
| North Macedonia | 0.83 (0.63, 1.09) | 0.90 (0.68, 1.19) | 8.83 | 38.89 (29.71, 51.14) | 38.75 (29.58, 50.66) | 39.02 (29.81, 50.96) | 39.53 (30.08, 52.11) | 0.07 (0.05, 0.08) | 0.02 (-0.05, 0.08) | 0.06 (0.05, 0.08) | 0.19 (0.13, 0.26) |
| Northern Mariana Islands | 0.19 (0.14, 0.26) | 0.13 (0.10, 0.17) | -31.61 | 330.60 (247.76, 437.36) | 319.06 (237.78, 418.20) | 316.09 (238.01, 417.32) | 320.44 (237.99, 426.34) | -0.12 (-0.14, -0.09) | -0.28 (-0.34, -0.21) | -0.09 (-0.19, 0.01) | 0.07 (-0.01, 0.15) |
| Norway | 3.20 (2.40, 4.28) | 3.81 (2.88, 5.02) | 19.04 | 74.03 (55.45, 98.62) | 74.41 (56.07, 98.61) | 73.76 (55.15, 98.11) | 74.28 (55.34, 98.22) | -0.03 (-0.05, -0.02) | 0.03 (-0.07, 0.12) | -0.10 (-0.15, -0.04) | 0.08 (0.03, 0.12) |
| Oman | 1.68 (1.19, 2.31) | 5.87 (4.07, 8.02) | 249.01 | 85.46 (62.63, 116.16) | 80.50 (59.24, 107.74) | 79.71 (58.75, 106.71) | 87.06 (63.95, 115.20) | 0.07 (-0.11, 0.25) | -0.59 (-0.80, -0.39) | -0.19 (-0.45, 0.06) | 0.69 (0.09, 1.30) |
| Pakistan | 237.42 (170.90, 321.93) | 548.10 (390.97, 750.32) | 130.86 | 240.58 (175.65, 320.97) | 213.85 (158.28, 282.48) | 254.16 (184.78, 340.07) | 245.63 (178.31, 331.61) | 0.53 (0.28, 0.77) | -1.08 (-1.66, -0.50) | 1.82 (1.03, 2.62) | -0.30 (-0.82, 0.21) |
| Palau | 0.05 (0.03, 0.07) | 0.05 (0.04, 0.07) | 10.22 | 274.80 (202.62, 370.03) | 279.41 (206.14, 372.50) | 275.39 (202.01, 366.83) | 289.44 (213.93, 385.69) | 0.16 (0.10, 0.22) | 0.24 (-0.08, 0.56) | -0.19 (-0.24, -0.15) | 0.59 (0.40, 0.77) |
| Palestine | 1.49 (1.06, 2.01) | 4.28 (3.08, 5.74) | 187.65 | 83.15 (61.38, 110.60) | 84.69 (62.37, 112.81) | 82.96 (62.16, 111.81) | 83.48 (61.35, 111.68) | -0.05 (-0.08, -0.02) | 0.18 (0.14, 0.22) | -0.22 (-0.26, -0.17) | 0.09 (0.05, 0.12) |
| Panama | 3.43 (2.49, 4.63) | 6.47 (5.06, 8.25) | 88.51 | 137.50 (102.52, 183.27) | 136.58 (102.42, 178.99) | 154.56 (124.22, 191.11) | 152.33 (119.58, 193.98) | 0.56 (0.44, 0.67) | -0.08 (-0.13, -0.03) | 1.29 (0.89, 1.69) | -0.18 (-0.33, -0.03) |
| Papua New Guinea | 20.29 (14.90, 26.49) | 45.70 (32.96, 60.89) | 125.18 | 482.87 (359.63, 628.61) | 547.47 (409.49, 704.98) | 441.15 (330.47, 579.80) | 438.48 (318.35, 580.46) | -0.90 (-1.14, -0.67) | 1.10 (0.53, 1.66) | -2.25 (-3.03, -1.46) | -0.21 (-0.40, -0.01) |
| Paraguay | 7.64 (5.54, 10.22) | 16.84 (12.15, 22.68) | 120.48 | 195.20 (143.73, 258.37) | 200.96 (149.22, 261.56) | 254.35 (197.60, 316.05) | 223.07 (162.72, 297.26) | 1.04 (0.75, 1.33) | 0.23 (-0.02, 0.48) | 2.48 (1.27, 3.70) | -1.08 (-2.01, -0.13) |
| Peru | 45.01 (33.20, 59.65) | 66.39 (50.65, 88.35) | 47.50 | 210.42 (158.76, 276.11) | 223.56 (177.58, 283.32) | 197.12 (157.11, 244.97) | 185.65 (141.98, 246.08) | -0.71 (-0.87, -0.56) | 0.55 (0.35, 0.76) | -1.27 (-1.46, -1.09) | -0.63 (-0.92, -0.35) |
| Philippines | 68.86 (49.87, 92.53) | 139.00 (101.05, 185.51) | 101.85 | 108.62 (80.14, 144.83) | 108.03 (79.98, 143.81) | 117.67 (86.44, 155.86) | 117.25 (85.95, 156.21) | 0.43 (0.35, 0.51) | -0.04 (-0.11, 0.03) | 0.90 (0.59, 1.20) | 0.09 (-0.07, 0.25) |
| Poland | 16.50 (12.52, 21.85) | 15.80 (11.94, 21.05) | -4.22 | 42.53 (32.18, 55.91) | 41.43 (31.29, 54.92) | 40.69 (30.76, 53.79) | 41.52 (31.38, 54.85) | -0.11 (-0.15, -0.07) | -0.26 (-0.27, -0.24) | -0.18 (-0.20, -0.17) | 0.30 (0.16, 0.44) |
| Portugal | 6.80 (5.01, 9.06) | 6.03 (4.56, 7.96) | -11.37 | 67.07 (49.18, 89.20) | 66.78 (49.80, 88.12) | 64.57 (47.83, 86.36) | 64.82 (47.55, 86.37) | -0.20 (-0.23, -0.17) | -0.05 (-0.20, 0.09) | -0.35 (-0.37, -0.33) | 0.08 (0.04, 0.12) |
| Puerto Rico | 5.53 (4.15, 7.25) | 4.84 (3.72, 6.39) | -12.44 | 149.74 (112.87, 196.77) | 145.71 (110.86, 193.66) | 147.52 (111.40, 195.74) | 149.14 (112.24, 198.00) | 0.02 (-0.01, 0.05) | -0.27 (-0.30, -0.24) | 0.13 (0.07, 0.19) | 0.14 (0.10, 0.17) |
| Qatar | 0.77 (0.55, 1.06) | 6.22 (4.35, 8.50) | 704.45 | 127.33 (94.66, 168.26) | 124.00 (94.05, 159.94) | 134.37 (98.64, 179.55) | 133.87 (97.50, 178.83) | 0.28 (0.18, 0.38) | -0.29 (-0.40, -0.18) | 0.93 (0.65, 1.20) | -0.08 (-0.14, -0.03) |
| Republic of Korea | 36.73 (28.10, 48.15) | 43.96 (33.24, 58.54) | 19.70 | 71.63 (55.42, 92.91) | 67.83 (51.67, 89.91) | 79.55 (59.46, 104.33) | 81.34 (61.08, 109.56) | 0.89 (0.72, 1.05) | -0.43 (-1.08, 0.22) | 1.69 (1.42, 1.96) | 0.26 (0.23, 0.29) |
| Republic of Moldova | 2.51 (1.91, 3.29) | 2.17 (1.70, 2.79) | -13.32 | 55.55 (42.42, 72.74) | 55.05 (42.12, 71.81) | 59.67 (46.96, 75.08) | 57.55 (45.46, 73.27) | 0.28 (0.19, 0.36) | -0.11 (-0.23, 0.00) | 0.84 (0.52, 1.17) | -0.33 (-0.49, -0.18) |
| Romania | 13.33 (10.04, 17.46) | 10.44 (8.02, 13.54) | -21.65 | 56.90 (42.63, 74.56) | 56.87 (42.57, 75.48) | 60.22 (45.64, 77.36) | 60.18 (45.59, 77.25) | 0.24 (0.16, 0.31) | -0.02 (-0.11, 0.08) | 0.60 (0.22, 0.98) | -0.02 (-0.03, 0.00) |
| Russian Federation | 80.29 (61.41, 105.81) | 73.58 (56.68, 97.81) | -8.36 | 50.52 (38.81, 66.16) | 49.72 (38.29, 64.85) | 48.42 (37.31, 63.49) | 49.31 (38.00, 64.25) | -0.13 (-0.16, -0.10) | -0.16 (-0.20, -0.12) | -0.28 (-0.34, -0.21) | 0.22 (0.12, 0.31) |
| Rwanda | 32.30 (24.14, 41.99) | 51.63 (38.96, 69.33) | 59.84 | 505.19 (384.71, 652.61) | 483.04 (372.90, 617.26) | 422.75 (329.95, 536.26) | 400.58 (308.41, 528.70) | -0.86 (-0.94, -0.78) | -0.48 (-0.54, -0.42) | -1.39 (-1.69, -1.08) | -0.67 (-1.04, -0.31) |
| Saint Kitts and Nevis | 0.06 (0.05, 0.09) | 0.10 (0.07, 0.13) | 52.20 | 156.33 (119.23, 203.09) | 151.36 (114.86, 198.48) | 151.71 (114.41, 198.03) | 152.37 (115.68, 202.36) | -0.09 (-0.13, -0.06) | -0.35 (-0.48, -0.21) | 0.03 (-0.04, 0.10) | 0.06 (0.01, 0.11) |
| Saint Lucia | 0.28 (0.20, 0.38) | 0.38 (0.29, 0.50) | 35.41 | 202.09 (151.42, 268.22) | 199.73 (149.92, 264.40) | 214.71 (166.07, 275.92) | 205.13 (155.67, 268.95) | 0.24 (0.15, 0.32) | -0.13 (-0.23, -0.04) | 0.76 (0.53, 0.98) | -0.47 (-0.78, -0.17) |
| Saint Vincent and the Grenadines | 0.19 (0.14, 0.25) | 0.18 (0.14, 0.23) | -3.50 | 170.61 (129.35, 221.05) | 154.90 (117.68, 202.01) | 154.89 (117.18, 203.79) | 154.23 (116.68, 203.36) | -0.38 (-0.49, -0.28) | -0.99 (-1.42, -0.56) | 0.02 (-0.02, 0.05) | -0.03 (-0.08, 0.02) |
| Samoa | 0.36 (0.26, 0.48) | 0.46 (0.34, 0.61) | 27.83 | 227.11 (167.18, 302.01) | 225.85 (167.12, 300.97) | 219.48 (162.79, 296.04) | 220.82 (163.07, 295.05) | -0.13 (-0.16, -0.10) | -0.06 (-0.07, -0.05) | -0.29 (-0.45, -0.14) | 0.06 (0.04, 0.09) |
| San Marino | 0.02 (0.01, 0.02) | 0.02 (0.01, 0.03) | 19.35 | 66.21 (49.22, 87.96) | 64.42 (47.59, 86.78) | 63.10 (46.82, 83.85) | 63.42 (47.01, 84.63) | -0.17 (-0.20, -0.14) | -0.28 (-0.29, -0.27) | -0.21 (-0.26, -0.16) | 0.08 (0.04, 0.12) |
| Sao Tome and Principe | 0.40 (0.29, 0.53) | 0.80 (0.59, 1.04) | 100.40 | 384.23 (284.77, 509.89) | 373.47 (279.12, 485.35) | 361.71 (275.16, 470.93) | 373.50 (280.85, 482.96) | -0.14 (-0.22, -0.05) | -0.28 (-0.31, -0.25) | -0.32 (-0.56, -0.08) | 0.38 (0.26, 0.49) |
| Saudi Arabia | 13.18 (9.48, 17.60) | 39.34 (28.64, 53.85) | 198.39 | 82.27 (60.28, 108.51) | 81.63 (60.27, 108.22) | 79.64 (58.95, 105.97) | 80.49 (59.14, 108.25) | -0.12 (-0.14, -0.10) | -0.09 (-0.11, -0.07) | -0.25 (-0.30, -0.20) | 0.10 (0.08, 0.12) |
| Senegal | 34.49 (24.88, 45.44) | 73.84 (52.85, 98.73) | 114.10 | 521.65 (383.40, 682.05) | 541.16 (403.16, 698.35) | 499.40 (363.61, 661.55) | 496.40 (362.53, 657.46) | -0.41 (-0.49, -0.32) | 0.32 (0.05, 0.60) | -0.84 (-0.90, -0.78) | -0.12 (-0.19, -0.05) |
| Serbia | 3.77 (2.92, 4.87) | 3.27 (2.50, 4.26) | -13.14 | 39.22 (30.01, 51.62) | 39.21 (30.08, 51.68) | 38.80 (29.47, 50.78) | 39.21 (29.71, 51.02) | -0.03 (-0.05, -0.01) | -0.05 (-0.13, 0.03) | -0.11 (-0.15, -0.07) | 0.14 (0.10, 0.19) |
| Seychelles | 0.06 (0.05, 0.09) | 0.09 (0.07, 0.12) | 44.87 | 82.80 (61.19, 111.42) | 81.06 (60.44, 108.91) | 81.72 (60.95, 109.00) | 83.08 (61.15, 111.53) | 0.04 (-0.01, 0.08) | -0.25 (-0.30, -0.19) | 0.08 (0.07, 0.10) | 0.31 (0.16, 0.46) |
| Sierra Leone | 12.99 (9.50, 17.00) | 32.05 (23.13, 43.38) | 146.74 | 387.56 (289.91, 503.90) | 370.48 (277.40, 476.87) | 367.31 (277.41, 488.90) | 380.76 (282.76, 504.20) | -0.03 (-0.11, 0.05) | -0.44 (-0.50, -0.39) | -0.10 (-0.21, 0.02) | 0.52 (0.39, 0.65) |
| Singapore | 2.92 (2.15, 3.95) | 4.94 (3.66, 6.73) | 68.90 | 76.98 (57.78, 102.39) | 76.65 (57.93, 101.91) | 77.11 (58.43, 101.55) | 76.83 (57.73, 103.32) | 0.02 (0.00, 0.04) | 0.00 (-0.06, 0.06) | 0.09 (0.01, 0.18) | 0.00 (-0.07, 0.07) |
| Slovakia | 1.89 (1.43, 2.45) | 1.96 (1.49, 2.59) | 3.59 | 35.07 (26.43, 45.38) | 35.11 (26.84, 46.21) | 34.87 (26.52, 46.39) | 35.55 (27.30, 47.08) | 0.02 (0.00, 0.04) | 0.01 (0.00, 0.02) | -0.07 (-0.11, -0.04) | 0.23 (0.21, 0.24) |
| Slovenia | 0.82 (0.63, 1.08) | 0.77 (0.58, 1.01) | -6.87 | 39.41 (30.04, 51.48) | 39.24 (29.62, 51.38) | 39.32 (30.08, 51.16) | 39.73 (30.11, 52.90) | 0.03 (0.02, 0.04) | -0.04 (-0.05, -0.03) | 0.02 (-0.01, 0.05) | 0.16 (0.10, 0.22) |
| Solomon Islands | 1.40 (1.03, 1.87) | 2.80 (2.08, 3.76) | 99.60 | 424.72 (313.86, 551.98) | 443.76 (329.30, 578.53) | 436.08 (324.67, 566.09) | 412.51 (307.55, 552.61) | -0.11 (-0.21, -0.01) | 0.45 (0.37, 0.52) | -0.18 (-0.26, -0.10) | -0.78 (-0.97, -0.59) |
| Somalia | 39.47 (30.71, 49.66) | 112.87 (85.74, 145.34) | 185.99 | 635.47 (493.71, 793.30) | 566.19 (447.67, 705.76) | 594.42 (467.17, 738.51) | 600.26 (461.91, 764.94) | -0.07 (-0.20, 0.05) | -1.21 (-1.30, -1.11) | 0.54 (0.45, 0.62) | 0.16 (0.09, 0.22) |
| South Africa | 428.84 (326.42, 551.86) | 465.08 (340.69, 612.57) | 8.45 | 1071.29 (818.26, 1373.64) | 687.48 (534.78, 867.05) | 587.03 (445.80, 756.89) | 750.50 (554.15, 989.90) | -1.34 (-1.97, -0.70) | -4.39 (-4.77, -4.00) | -1.51 (-1.97, -1.06) | 3.10 (2.47, 3.73) |
| South Sudan | 38.42 (28.46, 50.51) | 54.67 (41.27, 71.07) | 42.31 | 692.32 (529.91, 900.63) | 679.22 (522.36, 871.52) | 720.09 (577.32, 884.77) | 640.46 (486.72, 828.24) | 0.15 (0.03, 0.26) | -0.18 (-0.28, -0.08) | 0.58 (0.51, 0.65) | -0.90 (-1.59, -0.20) |
| Spain | 27.33 (20.36, 36.31) | 26.95 (20.51, 35.15) | -1.40 | 69.08 (51.37, 91.70) | 68.05 (50.57, 90.05) | 66.64 (49.61, 88.59) | 65.86 (48.88, 86.73) | -0.22 (-0.24, -0.20) | -0.17 (-0.32, -0.02) | -0.20 (-0.21, -0.19) | -0.14 (-0.16, -0.12) |
| Sri Lanka | 5.15 (3.84, 6.83) | 5.65 (4.25, 7.44) | 9.65 | 27.95 (21.12, 37.03) | 27.36 (20.77, 35.89) | 20.10 (15.41, 25.87) | 25.63 (19.09, 34.02) | -0.67 (-1.08, -0.26) | -0.19 (-0.25, -0.12) | -3.28 (-3.63, -2.93) | 3.05 (1.92, 4.18) |
| Sudan | 24.82 (18.11, 33.47) | 60.23 (43.56, 80.52) | 142.71 | 132.08 (96.79, 175.30) | 156.01 (115.54, 206.31) | 143.82 (108.36, 190.78) | 138.94 (103.26, 184.02) | -0.22 (-0.40, -0.04) | 1.46 (0.77, 2.15) | -0.85 (-1.45, -0.24) | -0.45 (-0.64, -0.26) |
| Suriname | 0.45 (0.33, 0.60) | 0.64 (0.50, 0.84) | 44.20 | 112.05 (85.01, 147.70) | 111.29 (84.47, 146.25) | 108.94 (83.21, 142.02) | 109.05 (82.51, 143.39) | -0.12 (-0.13, -0.10) | -0.07 (-0.08, -0.05) | -0.22 (-0.30, -0.15) | -0.04 (-0.12, 0.04) |
| Sweden | 5.94 (4.43, 7.87) | 6.54 (4.92, 8.68) | 10.23 | 71.20 (52.57, 93.79) | 70.30 (52.31, 94.10) | 69.52 (51.47, 92.95) | 70.87 (52.50, 95.07) | -0.06 (-0.08, -0.03) | -0.13 (-0.15, -0.11) | -0.11 (-0.14, -0.08) | 0.25 (0.16, 0.33) |
| Switzerland | 4.95 (3.67, 6.59) | 5.48 (4.12, 7.30) | 10.71 | 67.66 (49.94, 89.62) | 66.42 (49.17, 86.88) | 66.14 (48.70, 88.68) | 66.81 (49.50, 89.42) | -0.06 (-0.08, -0.04) | -0.20 (-0.25, -0.15) | -0.04 (-0.08, 0.00) | 0.10 (0.08, 0.12) |
| Syrian Arab Republic | 9.59 (6.99, 12.83) | 11.36 (8.51, 15.23) | 18.36 | 85.06 (63.07, 113.51) | 85.99 (63.68, 114.92) | 84.56 (62.97, 111.87) | 78.60 (58.52, 104.23) | -0.29 (-0.37, -0.20) | 0.10 (0.08, 0.12) | -0.14 (-0.22, -0.06) | -0.89 (-1.06, -0.73) |
| Taiwan (Province of China) | 21.92 (16.14, 29.19) | 22.40 (17.05, 29.24) | 2.21 | 95.35 (71.82, 126.50) | 94.75 (70.65, 124.74) | 93.31 (69.67, 123.16) | 94.53 (70.51, 124.89) | -0.08 (-0.10, -0.06) | -0.07 (-0.12, -0.02) | -0.16 (-0.26, -0.06) | 0.14 (0.09, 0.19) |
| Tajikistan | 2.72 (2.01, 3.57) | 4.94 (3.72, 6.49) | 81.93 | 56.63 (43.61, 73.60) | 58.86 (44.89, 76.32) | 51.08 (39.00, 65.87) | 49.60 (37.85, 64.53) | -0.75 (-0.87, -0.62) | 0.32 (0.07, 0.57) | -1.50 (-1.67, -1.34) | -0.49 (-0.73, -0.25) |
| Thailand | 84.58 (61.09, 113.34) | 86.97 (65.08, 114.25) | 2.83 | 130.94 (95.94, 174.61) | 154.76 (118.97, 197.76) | 120.01 (88.89, 159.49) | 122.20 (89.87, 161.01) | -0.89 (-1.19, -0.60) | 1.53 (0.95, 2.11) | -2.63 (-3.21, -2.05) | 0.26 (0.17, 0.36) |
| Timor-Leste | 0.74 (0.53, 0.98) | 1.23 (0.90, 1.62) | 67.03 | 93.99 (70.06, 124.84) | 94.02 (69.41, 124.95) | 95.29 (71.17, 126.21) | 93.46 (69.68, 122.23) | 0.04 (0.02, 0.05) | 0.00 (0.00, 0.01) | 0.15 (0.12, 0.17) | -0.15 (-0.24, -0.06) |
| Togo | 12.81 (9.22, 16.86) | 27.92 (20.55, 36.58) | 117.97 | 388.18 (286.19, 506.67) | 421.82 (315.50, 549.71) | 344.47 (260.78, 447.65) | 353.02 (263.40, 461.58) | -0.89 (-1.09, -0.68) | 0.70 (0.12, 1.28) | -2.16 (-2.42, -1.89) | 0.32 (0.20, 0.45) |
| Tokelau | 0.00 (0.00, 0.01) | 0.00 (0.00, 0.01) | -9.45 | 299.84 (221.51, 401.58) | 294.13 (219.59, 391.28) | 298.03 (220.48, 393.06) | 302.98 (224.18, 404.13) | 0.04 (0.01, 0.07) | -0.19 (-0.21, -0.17) | 0.17 (0.14, 0.19) | 0.18 (0.11, 0.25) |
| Tonga | 0.31 (0.23, 0.42) | 0.34 (0.25, 0.45) | 7.89 | 335.46 (246.41, 443.13) | 335.92 (251.69, 441.57) | 333.22 (245.68, 438.18) | 337.62 (248.38, 456.95) | 0.00 (-0.04, 0.04) | 0.06 (-0.12, 0.24) | -0.08 (-0.12, -0.05) | 0.06 (-0.17, 0.30) |
| Trinidad and Tobago | 1.58 (1.17, 2.11) | 1.69 (1.31, 2.14) | 6.85 | 127.31 (96.81, 168.54) | 126.75 (96.44, 165.98) | 117.87 (92.02, 150.38) | 118.97 (92.30, 151.49) | -0.31 (-0.39, -0.23) | -0.03 (-0.10, 0.05) | -0.74 (-1.12, -0.36) | 0.15 (0.09, 0.21) |
| Tunisia | 6.90 (4.95, 9.28) | 9.98 (7.41, 13.50) | 44.70 | 82.77 (60.92, 110.97) | 82.46 (60.98, 110.53) | 81.53 (60.39, 109.44) | 81.84 (60.06, 110.21) | -0.06 (-0.07, -0.04) | -0.03 (-0.05, 0.00) | -0.12 (-0.12, -0.11) | 0.06 (0.00, 0.11) |
| Turkey | 29.06 (21.30, 38.71) | 44.73 (33.33, 58.94) | 53.94 | 48.33 (36.32, 63.69) | 45.38 (34.66, 58.37) | 43.07 (32.39, 57.04) | 48.69 (36.39, 64.08) | 0.07 (-0.12, 0.26) | -0.56 (-0.82, -0.30) | -0.55 (-0.81, -0.29) | 1.49 (0.99, 1.99) |
| Turkmenistan | 1.91 (1.41, 2.55) | 2.79 (2.09, 3.67) | 45.92 | 53.90 (40.87, 71.04) | 53.42 (40.51, 70.13) | 51.39 (38.36, 68.36) | 52.15 (39.29, 68.80) | -0.20 (-0.24, -0.15) | -0.09 (-0.18, 0.00) | -0.41 (-0.49, -0.32) | 0.17 (0.14, 0.20) |
| Tuvalu | 0.02 (0.02, 0.03) | 0.03 (0.02, 0.05) | 39.02 | 264.89 (192.08, 356.54) | 265.82 (192.27, 362.56) | 267.30 (194.88, 365.08) | 276.39 (200.67, 374.40) | 0.09 (0.06, 0.11) | 0.04 (0.03, 0.05) | 0.05 (0.01, 0.09) | 0.28 (0.12, 0.44) |
| Uganda | 95.44 (70.12, 125.80) | 322.34 (272.14, 376.20) | 237.72 | 621.45 (470.90, 807.76) | 584.29 (475.25, 714.17) | 611.24 (493.82, 739.04) | 772.73 (648.37, 918.74) | 0.29 (0.05, 0.53) | -0.64 (-0.82, -0.45) | 0.43 (-0.01, 0.88) | 1.82 (0.62, 3.03) |
| Ukraine | 34.22 (26.16, 44.85) | 19.97 (15.20, 26.63) | -41.64 | 62.84 (47.94, 81.46) | 66.24 (50.83, 85.61) | 43.88 (33.48, 57.98) | 44.54 (34.14, 58.70) | -1.89 (-2.21, -1.57) | 0.46 (0.16, 0.75) | -4.37 (-4.85, -3.89) | 0.22 (0.13, 0.31) |
| United Arab Emirates | 2.24 (1.58, 3.13) | 12.63 (8.74, 18.22) | 463.46 | 91.41 (66.95, 123.16) | 93.84 (68.92, 126.21) | 95.88 (70.54, 129.86) | 88.31 (64.98, 118.36) | -0.03 (-0.12, 0.06) | 0.27 (0.21, 0.33) | 0.28 (0.08, 0.48) | -0.99 (-1.13, -0.84) |
| United Kingdom | 42.82 (32.08, 56.79) | 46.18 (34.99, 61.52) | 7.83 | 74.15 (55.32, 98.34) | 72.91 (54.37, 97.34) | 74.75 (55.87, 99.53) | 73.32 (54.45, 97.81) | 0.07 (0.02, 0.12) | -0.16 (-0.17, -0.15) | 0.26 (0.05, 0.48) | -0.08 (-0.26, 0.11) |
| United Republic of Tanzania | 253.58 (207.88, 303.02) | 294.10 (229.94, 364.90) | 15.98 | 1022.81 (838.78, 1220.09) | 740.50 (611.58, 885.05) | 500.24 (401.85, 622.34) | 548.76 (428.91, 681.18) | -2.83 (-3.21, -2.45) | -3.21 (-3.94, -2.46) | -3.83 (-4.26, -3.41) | 1.37 (0.58, 2.17) |
| United States of America | 189.60 (143.24, 252.48) | 234.51 (179.45, 308.64) | 23.69 | 70.09 (53.19, 92.23) | 75.91 (57.56, 100.80) | 73.72 (56.03, 97.10) | 73.96 (55.71, 97.89) | 0.20 (-0.04, 0.44) | 0.77 (0.43, 1.12) | -0.37 (-1.37, 0.64) | 0.06 (0.02, 0.10) |
| United States Virgin Islands | 0.17 (0.13, 0.22) | 0.14 (0.11, 0.18) | -17.22 | 155.96 (118.35, 203.81) | 152.90 (116.21, 199.08) | 154.79 (118.27, 202.73) | 153.87 (116.40, 202.50) | -0.01 (-0.04, 0.01) | -0.21 (-0.27, -0.16) | 0.13 (0.05, 0.22) | -0.08 (-0.18, 0.02) |
| Uruguay | 3.86 (2.85, 5.12) | 3.86 (3.17, 4.64) | -0.13 | 128.05 (93.98, 170.10) | 126.30 (93.28, 166.92) | 134.29 (106.39, 166.77) | 117.91 (96.71, 141.43) | 0.03 (-0.10, 0.16) | -0.17 (-0.39, 0.06) | 0.67 (0.32, 1.01) | -1.06 (-1.74, -0.39) |
| Uzbekistan | 10.94 (8.16, 14.34) | 18.50 (13.90, 24.36) | 69.15 | 55.54 (42.65, 72.54) | 55.88 (42.35, 73.29) | 50.54 (38.29, 66.96) | 50.65 (38.47, 66.57) | -0.55 (-0.63, -0.47) | 0.03 (-0.20, 0.26) | -1.06 (-1.10, -1.01) | 0.01 (-0.03, 0.05) |
| Vanuatu | 0.44 (0.32, 0.59) | 0.91 (0.66, 1.23) | 105.76 | 305.71 (226.03, 405.42) | 308.32 (232.57, 401.49) | 308.83 (229.88, 403.79) | 305.37 (225.29, 411.09) | 0.00 (-0.03, 0.03) | 0.10 (0.02, 0.19) | 0.02 (0.00, 0.03) | -0.14 (-0.29, 0.01) |
| Venezuela (Bolivarian Republic of) | 33.01 (24.03, 43.91) | 52.91 (40.43, 68.10) | 60.28 | 167.37 (124.29, 219.99) | 168.60 (125.69, 221.06) | 185.15 (138.96, 239.08) | 186.66 (141.10, 239.70) | 0.42 (0.34, 0.50) | 0.03 (-0.13, 0.20) | 0.97 (0.71, 1.24) | 0.01 (-0.09, 0.10) |
| Viet Nam | 51.71 (36.98, 70.16) | 84.54 (62.18, 113.15) | 63.48 | 75.60 (55.86, 100.44) | 76.57 (57.41, 102.02) | 76.42 (56.95, 101.34) | 78.22 (57.70, 105.13) | 0.09 (0.06, 0.12) | 0.14 (0.10, 0.19) | -0.02 (-0.12, 0.07) | 0.35 (0.19, 0.50) |
| Yemen | 19.06 (13.77, 25.45) | 53.43 (38.59, 71.69) | 180.37 | 170.75 (123.51, 227.34) | 194.82 (146.10, 251.48) | 172.06 (123.75, 232.06) | 168.91 (122.38, 224.84) | -0.46 (-0.62, -0.29) | 1.18 (0.62, 1.75) | -1.29 (-1.65, -0.93) | -0.24 (-0.34, -0.15) |
| Zambia | 63.74 (49.49, 79.20) | 134.91 (103.88, 170.40) | 111.66 | 852.68 (664.99, 1058.44) | 1008.94 (798.45, 1251.33) | 730.14 (593.14, 882.47) | 736.58 (572.75, 920.96) | -1.12 (-1.46, -0.79) | 1.64 (1.32, 1.97) | -3.38 (-3.94, -2.82) | 0.26 (0.06, 0.46) |
| Zimbabwe | 41.66 (30.63, 55.53) | 63.12 (49.63, 80.80) | 51.49 | 446.30 (335.31, 588.24) | 422.49 (336.57, 528.47) | 412.92 (330.19, 506.80) | 419.57 (329.82, 528.34) | -0.20 (-0.24, -0.15) | -0.50 (-0.71, -0.29) | -0.23 (-0.25, -0.20) | 0.10 (-0.05, 0.24) |
| **Chlamydial infection** | | |  |  |  |  |  |  |  |  |  |
| Afghanistan | 247.03 (185.79, 321.87) | 999.09 (749.41, 1309.91) | 304.44 | 2912.87 (2192.90, 3842.66) | 2922.21 (2191.22, 3815.62) | 2972.97 (2224.47, 3899.45) | 2954.92 (2228.57, 3867.13) | 0.08 (0.06, 0.10) | 0.03 (0.01, 0.06) | 0.18 (0.13, 0.22) | -0.11 (-0.19, -0.03) |
| Albania | 115.03 (84.15, 153.80) | 89.55 (67.71, 118.71) | -22.15 | 3384.51 (2519.64, 4468.16) | 3334.01 (2475.64, 4398.90) | 3293.91 (2437.11, 4319.12) | 3316.46 (2493.88, 4414.47) | -0.08 (-0.10, -0.05) | -0.13 (-0.16, -0.10) | -0.12 (-0.16, -0.09) | 0.09 (0.07, 0.11) |
| Algeria | 725.76 (542.62, 962.76) | 1301.73 (959.56, 1720.03) | 79.36 | 3213.42 (2431.86, 4198.88) | 2647.13 (1965.09, 3469.81) | 2821.25 (2100.53, 3690.38) | 2801.34 (2079.03, 3681.57) | -0.02 (-0.22, 0.18) | -1.73 (-2.47, -0.98) | 0.66 (0.37, 0.95) | -0.08 (-0.13, -0.04) |
| American Samoa | 1.41 (1.05, 1.87) | 1.57 (1.20, 2.06) | 11.64 | 2919.95 (2225.69, 3817.63) | 2886.26 (2192.10, 3762.83) | 2897.14 (2191.97, 3772.32) | 2932.15 (2233.29, 3838.33) | 0.01 (-0.01, 0.03) | -0.13 (-0.15, -0.10) | 0.04 (-0.02, 0.10) | 0.11 (0.07, 0.15) |
| Andorra | 0.23 (0.17, 0.30) | 0.31 (0.24, 0.40) | 37.33 | 348.54 (263.77, 450.60) | 347.01 (262.69, 453.81) | 345.59 (261.59, 455.09) | 344.72 (261.51, 447.79) | -0.04 (-0.05, -0.03) | -0.04 (-0.06, -0.02) | -0.03 (-0.06, 0.00) | 0.02 (-0.03, 0.07) |
| Angola | 197.37 (144.58, 263.97) | 541.93 (401.37, 715.49) | 174.58 | 2242.16 (1651.86, 2938.60) | 2208.05 (1656.34, 2895.09) | 2169.22 (1614.16, 2868.16) | 2135.03 (1589.47, 2817.56) | -0.16 (-0.17, -0.15) | -0.17 (-0.23, -0.10) | -0.18 (-0.23, -0.13) | -0.13 (-0.24, -0.03) |
| Antigua and Barbuda | 2.79 (2.08, 3.68) | 4.44 (3.32, 5.71) | 59.19 | 4484.51 (3384.01, 5882.98) | 4491.86 (3410.12, 5837.19) | 4474.20 (3388.61, 5871.38) | 4488.65 (3352.34, 5835.81) | 0.00 (-0.01, 0.00) | 0.02 (0.01, 0.02) | -0.04 (-0.07, -0.01) | 0.03 (0.01, 0.05) |
| Argentina | 278.14 (211.63, 354.59) | 430.64 (323.90, 557.52) | 54.83 | 877.14 (666.42, 1119.07) | 877.03 (666.51, 1124.22) | 905.60 (678.09, 1188.85) | 910.81 (684.59, 1181.16) | 0.17 (0.13, 0.20) | 0.00 (-0.01, 0.01) | 0.33 (0.16, 0.50) | 0.03 (0.00, 0.06) |
| Armenia | 187.85 (138.30, 249.05) | 168.48 (125.10, 222.33) | -10.31 | 5264.83 (3938.09, 6847.60) | 5316.75 (3995.11, 6904.50) | 5260.44 (3938.27, 6892.02) | 5240.39 (3929.45, 6820.74) | -0.04 (-0.06, -0.03) | 0.10 (0.10, 0.11) | -0.11 (-0.12, -0.10) | -0.04 (-0.09, 0.01) |
| Australia | 171.59 (128.35, 222.78) | 231.74 (172.65, 299.74) | 35.06 | 942.44 (707.23, 1220.29) | 939.63 (727.90, 1203.05) | 915.18 (722.31, 1134.61) | 939.35 (698.76, 1217.89) | -0.07 (-0.12, -0.02) | -0.02 (-0.04, 0.00) | -0.27 (-0.44, -0.11) | 0.28 (0.26, 0.30) |
| Austria | 29.13 (21.95, 38.25) | 31.28 (23.59, 40.21) | 7.38 | 347.47 (263.21, 457.50) | 347.36 (264.70, 458.97) | 347.08 (263.10, 450.30) | 348.23 (264.36, 457.14) | 0.01 (0.00, 0.02) | 0.01 (-0.04, 0.06) | -0.01 (-0.04, 0.03) | 0.02 (0.00, 0.04) |
| Azerbaijan | 401.04 (293.10, 536.76) | 634.34 (469.45, 831.55) | 58.17 | 5380.97 (4066.30, 6995.59) | 5423.36 (4071.69, 7093.25) | 5300.60 (4008.04, 6964.62) | 5295.53 (3990.41, 6929.49) | -0.09 (-0.11, -0.07) | 0.09 (0.07, 0.11) | -0.24 (-0.26, -0.23) | -0.02 (-0.04, -0.01) |
| Bahamas | 12.55 (9.30, 16.61) | 18.92 (14.35, 24.64) | 50.70 | 4495.97 (3379.20, 5837.06) | 4502.17 (3346.93, 5896.63) | 4499.68 (3359.00, 5860.41) | 4503.00 (3408.11, 5894.36) | 0.00 (0.00, 0.00) | 0.01 (0.01, 0.02) | -0.01 (-0.02, 0.01) | 0.01 (0.00, 0.03) |
| Bahrain | 18.92 (13.77, 25.76) | 56.98 (41.80, 75.27) | 201.14 | 2938.03 (2195.59, 3871.90) | 2849.02 (2135.45, 3772.32) | 2938.31 (2167.76, 3862.99) | 2890.11 (2170.64, 3792.84) | 0.04 (0.00, 0.09) | -0.32 (-0.37, -0.27) | 0.32 (0.28, 0.37) | -0.27 (-0.40, -0.14) |
| Bangladesh | 1810.08 (1342.75, 2422.78) | 3217.84 (2408.61, 4233.86) | 77.77 | 1902.47 (1428.91, 2515.05) | 1885.77 (1406.13, 2486.32) | 1883.23 (1409.40, 2481.00) | 1878.52 (1407.68, 2461.84) | -0.02 (-0.03, -0.01) | -0.08 (-0.12, -0.04) | -0.02 (-0.05, 0.02) | -0.03 (-0.07, 0.00) |
| Barbados | 15.35 (11.44, 20.26) | 16.64 (12.57, 21.44) | 8.42 | 5663.54 (4267.90, 7401.34) | 6094.85 (4670.20, 7729.70) | 6056.37 (4641.98, 7695.75) | 5655.81 (4210.24, 7356.17) | 0.00 (-0.14, 0.15) | 0.77 (0.46, 1.08) | -0.07 (-0.07, -0.06) | -0.87 (-1.19, -0.54) |
| Belarus | 408.73 (304.88, 544.12) | 365.02 (270.97, 481.09) | -10.70 | 3780.76 (2823.84, 4997.57) | 3799.89 (2853.12, 5008.45) | 3785.08 (2809.55, 4965.77) | 3775.38 (2813.70, 5023.31) | 0.00 (-0.01, 0.01) | 0.05 (0.03, 0.07) | -0.04 (-0.06, -0.02) | 0.00 (-0.03, 0.03) |
| Belgium | 46.30 (35.45, 60.13) | 48.77 (36.91, 63.11) | 5.33 | 445.38 (341.26, 576.80) | 496.61 (384.94, 627.13) | 449.13 (343.31, 589.48) | 447.18 (338.59, 578.75) | -0.07 (-0.27, 0.13) | 1.15 (0.69, 1.62) | -1.03 (-1.47, -0.59) | -0.06 (-0.08, -0.04) |
| Belize | 7.18 (5.30, 9.48) | 19.81 (14.86, 26.10) | 175.79 | 4534.07 (3372.31, 5917.31) | 4542.97 (3409.79, 5943.30) | 4549.32 (3395.57, 5908.92) | 4550.83 (3412.44, 5956.73) | 0.01 (0.01, 0.01) | 0.02 (0.01, 0.02) | 0.01 (0.00, 0.03) | 0.00 (-0.02, 0.01) |
| Benin | 63.95 (47.17, 85.20) | 185.10 (136.43, 243.53) | 189.43 | 1659.24 (1245.07, 2179.47) | 1597.86 (1307.48, 1913.15) | 1693.45 (1409.72, 2007.24) | 1665.56 (1249.21, 2156.03) | 0.18 (0.10, 0.25) | -0.33 (-0.50, -0.17) | 0.60 (0.37, 0.83) | -0.20 (-0.27, -0.14) |
| Bermuda | 3.16 (2.35, 4.17) | 2.67 (2.04, 3.43) | -15.50 | 4420.22 (3303.39, 5814.25) | 4419.13 (3323.10, 5791.30) | 4415.91 (3296.35, 5779.82) | 4425.82 (3330.71, 5824.27) | 0.00 (-0.01, 0.00) | 0.00 (-0.01, 0.02) | -0.01 (-0.03, 0.01) | 0.01 (-0.03, 0.06) |
| Bhutan | 10.46 (7.80, 13.86) | 16.03 (11.80, 21.07) | 53.29 | 1857.77 (1395.02, 2436.22) | 1842.02 (1361.27, 2421.74) | 1829.08 (1356.40, 2389.62) | 1829.31 (1349.63, 2412.45) | -0.06 (-0.07, -0.05) | -0.09 (-0.10, -0.09) | -0.07 (-0.07, -0.07) | -0.01 (-0.02, 0.01) |
| Bolivia (Plurinational State of) | 135.93 (101.73, 178.29) | 293.03 (220.24, 386.12) | 115.57 | 2417.86 (1803.25, 3193.43) | 2395.39 (1783.37, 3181.30) | 2385.48 (1769.61, 3153.54) | 2418.83 (1825.05, 3180.13) | 0.00 (-0.03, 0.02) | -0.10 (-0.12, -0.08) | -0.04 (-0.10, 0.01) | 0.25 (0.15, 0.34) |
| Bosnia and Herzegovina | 168.14 (124.12, 223.50) | 106.20 (80.11, 136.91) | -36.84 | 3351.00 (2485.17, 4451.91) | 3312.80 (2462.06, 4347.22) | 3304.64 (2436.97, 4381.53) | 3310.50 (2471.74, 4347.53) | -0.04 (-0.05, -0.03) | -0.13 (-0.16, -0.10) | -0.02 (-0.07, 0.02) | 0.02 (0.00, 0.03) |
| Botswana | 38.30 (28.60, 50.39) | 86.14 (64.57, 114.36) | 124.91 | 3326.62 (2511.95, 4354.76) | 3663.17 (2931.59, 4431.68) | 3424.05 (2746.41, 4182.52) | 3246.20 (2460.81, 4279.77) | -0.18 (-0.38, 0.02) | 1.05 (0.83, 1.27) | -0.66 (-1.03, -0.29) | -0.59 (-0.80, -0.38) |
| Brazil | 5992.73 (4453.65, 7976.53) | 9836.53 (7355.87, 12812.83) | 64.14 | 4074.41 (3059.73, 5360.96) | 4310.31 (3246.74, 5634.43) | 3895.08 (2939.87, 5101.92) | 4091.96 (3069.75, 5380.08) | -0.17 (-0.31, -0.04) | 0.60 (0.25, 0.95) | -1.06 (-1.18, -0.94) | 0.69 (0.26, 1.13) |
| Brunei Darussalam | 3.17 (2.33, 4.26) | 5.90 (4.42, 7.84) | 86.37 | 1096.17 (824.69, 1439.52) | 1078.68 (801.32, 1430.31) | 1078.18 (807.64, 1430.72) | 1087.48 (821.15, 1440.71) | -0.02 (-0.04, 0.01) | -0.17 (-0.18, -0.16) | 0.00 (-0.04, 0.04) | 0.11 (0.09, 0.13) |
| Bulgaria | 322.39 (242.72, 422.29) | 242.79 (181.06, 317.36) | -24.69 | 3767.22 (2808.41, 4970.54) | 3898.37 (3011.28, 5008.18) | 3740.84 (2787.56, 4938.03) | 3732.67 (2790.87, 4935.88) | -0.15 (-0.21, -0.10) | 0.30 (0.15, 0.45) | -0.43 (-0.61, -0.25) | 0.01 (-0.03, 0.06) |
| Burkina Faso | 138.79 (103.61, 183.23) | 363.74 (269.33, 483.94) | 162.08 | 1898.33 (1426.16, 2487.61) | 1900.16 (1420.94, 2492.28) | 1866.99 (1394.00, 2437.47) | 1862.78 (1387.19, 2461.37) | -0.07 (-0.09, -0.05) | 0.01 (-0.01, 0.03) | -0.18 (-0.27, -0.10) | 0.01 (-0.05, 0.07) |
| Burundi | 151.42 (111.13, 201.74) | 337.27 (250.77, 443.69) | 122.75 | 3224.40 (2402.40, 4218.90) | 3222.14 (2410.35, 4211.61) | 3233.73 (2429.73, 4191.68) | 3225.14 (2430.16, 4191.59) | 0.02 (0.01, 0.03) | -0.01 (-0.04, 0.01) | 0.04 (-0.02, 0.10) | -0.01 (-0.08, 0.07) |
| Cabo Verde | 5.25 (3.86, 7.04) | 11.31 (8.52, 15.02) | 115.56 | 1841.19 (1385.12, 2399.15) | 1830.91 (1374.20, 2391.95) | 1815.92 (1357.43, 2389.67) | 1810.94 (1364.16, 2385.18) | -0.03 (-0.05, -0.02) | -0.05 (-0.08, -0.02) | -0.09 (-0.15, -0.02) | -0.02 (-0.10, 0.07) |
| Cambodia | 386.31 (284.42, 518.02) | 782.72 (583.01, 1020.73) | 102.62 | 4411.92 (3285.32, 5824.58) | 4477.83 (3389.02, 5867.83) | 4388.77 (3286.95, 5743.98) | 4399.50 (3295.75, 5701.87) | -0.04 (-0.06, -0.01) | 0.16 (0.11, 0.21) | -0.22 (-0.25, -0.19) | -0.01 (-0.07, 0.05) |
| Cameroon | 175.90 (130.78, 234.22) | 551.58 (411.01, 734.36) | 213.56 | 2026.38 (1519.61, 2659.40) | 2079.10 (1630.41, 2596.56) | 2020.83 (1512.88, 2652.08) | 1997.33 (1495.32, 2609.03) | 0.01 (-0.08, 0.09) | 0.31 (0.03, 0.58) | -0.32 (-0.54, -0.10) | -0.09 (-0.17, -0.02) |
| Canada | 100.77 (75.84, 131.67) | 117.37 (89.89, 152.07) | 16.48 | 337.32 (255.98, 439.30) | 336.73 (253.37, 438.25) | 250.62 (198.26, 320.48) | 336.19 (254.78, 434.91) | -0.84 (-1.36, -0.32) | 0.00 (-0.03, 0.02) | -3.06 (-4.30, -1.81) | 4.10 (2.20, 6.04) |
| Central African Republic | 55.17 (40.97, 73.43) | 110.69 (81.99, 146.59) | 100.64 | 2334.59 (1735.51, 3070.71) | 2357.17 (1767.78, 3088.74) | 2343.50 (1765.98, 3068.01) | 2320.97 (1719.72, 3065.63) | -0.04 (-0.07, -0.02) | 0.07 (-0.03, 0.17) | -0.06 (-0.18, 0.06) | -0.10 (-0.16, -0.03) |
| Chad | 90.54 (67.30, 119.81) | 239.63 (178.70, 318.84) | 164.66 | 1910.37 (1445.50, 2484.99) | 1906.74 (1433.30, 2498.06) | 1878.91 (1407.47, 2460.08) | 1875.60 (1412.13, 2456.79) | -0.05 (-0.07, -0.03) | -0.01 (-0.04, 0.02) | -0.16 (-0.24, -0.07) | 0.01 (-0.08, 0.10) |
| Chile | 124.38 (93.79, 163.40) | 176.35 (132.98, 227.51) | 41.79 | 899.65 (682.54, 1167.18) | 904.13 (680.73, 1169.44) | 896.66 (673.91, 1174.65) | 903.33 (678.35, 1171.68) | -0.01 (-0.03, 0.00) | 0.05 (0.04, 0.06) | -0.09 (-0.10, -0.07) | 0.08 (0.06, 0.09) |
| China | 51171.51 (38121.40, 67573.51) | 66494.72 (49692.91, 86668.66) | 29.94 | 4012.21 (2994.24, 5232.57) | 4708.35 (3530.96, 6142.73) | 3479.80 (2629.25, 4559.35) | 4097.98 (3058.95, 5338.10) | -0.66 (-1.09, -0.23) | 1.72 (0.95, 2.49) | -3.03 (-4.22, -1.83) | 1.13 (0.05, 2.23) |
| Colombia | 976.24 (729.58, 1273.04) | 1581.69 (1186.42, 2086.13) | 62.02 | 2969.11 (2250.08, 3828.74) | 2956.12 (2245.34, 3777.61) | 3111.37 (2334.41, 4049.71) | 3113.97 (2325.05, 4114.97) | 0.22 (0.16, 0.28) | -0.05 (-0.07, -0.03) | 0.53 (0.27, 0.80) | 0.00 (-0.04, 0.05) |
| Comoros | 12.16 (9.05, 16.00) | 23.41 (17.50, 30.98) | 92.51 | 3165.78 (2366.93, 4114.74) | 3184.39 (2381.24, 4184.72) | 3201.16 (2408.24, 4162.71) | 3191.64 (2388.45, 4213.20) | 0.04 (0.03, 0.05) | 0.06 (0.06, 0.06) | 0.05 (0.04, 0.07) | -0.05 (-0.07, -0.02) |
| Congo | 44.55 (32.75, 59.16) | 111.52 (83.22, 146.88) | 150.32 | 2185.29 (1606.52, 2890.48) | 2186.61 (1623.47, 2911.66) | 2175.91 (1607.61, 2852.57) | 2143.24 (1606.92, 2826.79) | -0.05 (-0.08, -0.03) | -0.01 (-0.10, 0.08) | -0.05 (-0.20, 0.10) | -0.10 (-0.22, 0.02) |
| Cook Islands | 0.53 (0.40, 0.68) | 0.51 (0.39, 0.66) | -2.76 | 2855.42 (2159.09, 3691.02) | 2892.53 (2193.65, 3797.84) | 2993.55 (2261.65, 3934.19) | 3068.84 (2334.11, 3965.59) | 0.25 (0.22, 0.29) | 0.12 (0.05, 0.20) | 0.34 (0.25, 0.43) | 0.27 (0.22, 0.31) |
| Costa Rica | 110.09 (82.38, 144.34) | 187.06 (140.12, 243.27) | 69.90 | 3626.52 (2759.06, 4687.68) | 3610.47 (2722.14, 4712.72) | 3609.89 (2697.88, 4720.86) | 3619.80 (2711.49, 4687.23) | -0.02 (-0.02, -0.01) | -0.05 (-0.08, -0.02) | 0.00 (-0.03, 0.03) | 0.04 (0.01, 0.07) |
| Croatia | 181.57 (135.59, 239.93) | 140.76 (105.01, 182.17) | -22.47 | 3541.37 (2632.55, 4692.62) | 3517.29 (2627.45, 4634.53) | 3753.57 (2819.33, 4893.77) | 3529.98 (2608.29, 4645.50) | 0.16 (0.05, 0.27) | -0.08 (-0.12, -0.04) | 0.68 (0.42, 0.94) | -0.87 (-1.29, -0.45) |
| Cuba | 534.01 (400.38, 704.52) | 503.86 (379.28, 651.22) | -5.65 | 4432.77 (3327.46, 5794.80) | 4420.86 (3290.82, 5749.41) | 4421.44 (3322.82, 5821.35) | 4429.93 (3288.28, 5795.12) | 0.00 (-0.01, 0.00) | -0.03 (-0.03, -0.02) | 0.00 (-0.02, 0.02) | 0.00 (-0.03, 0.03) |
| Cyprus | 2.88 (2.16, 3.78) | 5.08 (3.86, 6.58) | 76.52 | 353.55 (265.98, 462.80) | 349.90 (265.93, 457.69) | 349.00 (265.46, 457.63) | 348.54 (266.43, 456.18) | -0.03 (-0.04, -0.02) | -0.10 (-0.13, -0.06) | -0.03 (-0.07, 0.01) | 0.00 (-0.02, 0.03) |
| Czechia | 351.91 (261.76, 456.53) | 343.01 (257.10, 442.48) | -2.53 | 3370.70 (2526.10, 4419.41) | 3382.61 (2560.58, 4412.94) | 3353.35 (2489.50, 4396.30) | 3355.27 (2499.87, 4413.37) | -0.02 (-0.03, 0.00) | 0.04 (0.00, 0.08) | -0.09 (-0.15, -0.03) | 0.04 (0.00, 0.07) |
| Côte d'Ivoire | 233.95 (172.72, 308.44) | 537.26 (399.30, 707.68) | 129.65 | 2166.81 (1626.29, 2826.99) | 2247.35 (1729.08, 2857.32) | 2113.25 (1573.23, 2763.80) | 2112.72 (1600.38, 2775.45) | -0.24 (-0.33, -0.16) | 0.28 (0.02, 0.55) | -0.63 (-0.98, -0.28) | 0.04 (-0.08, 0.15) |
| Democratic People's Republic of Korea | 1028.34 (775.73, 1342.28) | 1396.76 (1054.98, 1829.16) | 35.83 | 4805.80 (3637.54, 6279.34) | 4778.32 (3590.71, 6237.76) | 4786.34 (3609.12, 6269.58) | 4755.56 (3556.72, 6272.03) | -0.03 (-0.04, -0.02) | -0.06 (-0.08, -0.05) | 0.02 (0.01, 0.02) | -0.12 (-0.18, -0.06) |
| Democratic Republic of the Congo | 652.92 (482.11, 872.51) | 1627.93 (1195.38, 2193.53) | 149.33 | 2083.50 (1548.88, 2757.91) | 2079.84 (1546.28, 2732.01) | 2107.25 (1575.34, 2804.40) | 2087.52 (1547.92, 2776.03) | 0.03 (0.01, 0.05) | -0.04 (-0.17, 0.09) | 0.13 (0.09, 0.17) | -0.06 (-0.19, 0.08) |
| Denmark | 28.78 (21.84, 37.58) | 29.00 (22.08, 37.23) | 0.77 | 522.40 (398.01, 683.40) | 561.61 (430.06, 723.86) | 519.40 (396.20, 683.72) | 516.44 (392.35, 668.04) | -0.09 (-0.23, 0.05) | 0.77 (0.41, 1.13) | -0.80 (-1.09, -0.52) | 0.01 (-0.08, 0.10) |
| Djibouti | 14.60 (10.76, 19.39) | 41.60 (30.85, 54.49) | 185.01 | 3308.02 (2472.43, 4334.14) | 3303.73 (2480.96, 4332.78) | 3247.27 (2431.66, 4261.55) | 3228.15 (2415.95, 4217.51) | -0.10 (-0.12, -0.08) | -0.01 (-0.03, 0.01) | -0.18 (-0.26, -0.11) | -0.04 (-0.07, -0.01) |
| Dominica | 3.00 (2.23, 4.00) | 3.13 (2.35, 4.10) | 4.10 | 4486.80 (3347.49, 5945.53) | 4494.60 (3350.29, 5929.59) | 4476.62 (3322.72, 5862.53) | 4500.96 (3334.36, 5902.27) | 0.00 (-0.01, 0.00) | 0.02 (0.01, 0.03) | -0.04 (-0.05, -0.03) | 0.05 (0.03, 0.07) |
| Dominican Republic | 311.03 (231.43, 409.20) | 523.41 (390.88, 683.36) | 68.28 | 4515.81 (3401.95, 5820.77) | 4508.21 (3387.23, 5925.78) | 4506.09 (3358.40, 5874.05) | 4521.88 (3375.02, 5914.83) | 0.00 (-0.01, 0.00) | -0.02 (-0.02, -0.01) | -0.01 (-0.01, 0.00) | 0.04 (0.03, 0.06) |
| Ecuador | 190.51 (140.69, 250.59) | 372.26 (273.04, 494.00) | 95.40 | 2042.41 (1509.16, 2685.90) | 2014.47 (1502.04, 2665.36) | 2010.96 (1487.13, 2665.18) | 2049.23 (1507.92, 2717.09) | 0.02 (-0.02, 0.05) | -0.14 (-0.16, -0.12) | -0.02 (-0.14, 0.10) | 0.35 (0.19, 0.50) |
| Egypt | 2067.54 (1516.96, 2744.62) | 3644.78 (2674.98, 4942.83) | 76.29 | 4018.83 (2972.39, 5317.13) | 3922.15 (2965.75, 5146.80) | 3659.00 (2685.62, 4874.16) | 3565.35 (2634.62, 4795.80) | -0.47 (-0.56, -0.38) | -0.21 (-0.28, -0.14) | -0.72 (-1.31, -0.13) | -0.33 (-0.38, -0.27) |
| El Salvador | 175.66 (131.40, 230.32) | 239.50 (179.95, 315.07) | 36.34 | 3732.10 (2812.41, 4820.73) | 3719.19 (2805.11, 4843.98) | 3690.89 (2769.27, 4788.49) | 3674.47 (2760.90, 4828.83) | -0.05 (-0.06, -0.04) | -0.04 (-0.05, -0.02) | -0.08 (-0.09, -0.07) | 0.01 (-0.06, 0.07) |
| Equatorial Guinea | 7.16 (5.29, 9.36) | 29.67 (21.52, 39.65) | 314.26 | 2137.67 (1577.13, 2788.63) | 2106.39 (1574.35, 2769.91) | 2119.94 (1563.92, 2809.16) | 2152.81 (1604.24, 2853.50) | 0.03 (-0.01, 0.07) | -0.17 (-0.29, -0.06) | 0.06 (0.00, 0.12) | 0.24 (0.12, 0.37) |
| Eritrea | 83.53 (62.02, 110.43) | 212.57 (156.08, 281.01) | 154.50 | 3236.49 (2418.81, 4238.44) | 3221.66 (2411.88, 4187.12) | 3222.15 (2434.88, 4211.81) | 3209.61 (2376.05, 4235.26) | -0.02 (-0.03, -0.01) | -0.05 (-0.07, -0.02) | 0.00 (-0.06, 0.06) | -0.02 (-0.06, 0.03) |
| Estonia | 60.18 (44.89, 78.95) | 47.68 (35.28, 62.48) | -20.78 | 3756.96 (2808.33, 4946.89) | 3767.98 (2814.53, 4942.79) | 3752.21 (2792.83, 4929.43) | 3741.71 (2758.31, 4931.67) | -0.01 (-0.02, 0.00) | 0.04 (0.02, 0.06) | -0.04 (-0.06, -0.03) | -0.03 (-0.05, -0.02) |
| Eswatini | 23.09 (17.12, 30.60) | 39.95 (29.51, 53.50) | 72.98 | 3434.44 (2573.27, 4504.29) | 3421.42 (2600.84, 4437.81) | 3397.56 (2554.41, 4401.67) | 3357.71 (2522.80, 4443.93) | -0.07 (-0.08, -0.06) | -0.04 (-0.06, -0.02) | -0.07 (-0.12, -0.02) | -0.13 (-0.19, -0.07) |
| Ethiopia | 1384.48 (1045.17, 1825.28) | 3137.68 (2336.36, 4159.90) | 126.63 | 3378.81 (2551.78, 4405.90) | 3385.62 (2559.03, 4430.17) | 3328.90 (2485.81, 4334.76) | 3303.52 (2451.45, 4312.96) | -0.11 (-0.12, -0.09) | 0.01 (-0.02, 0.05) | -0.17 (-0.21, -0.13) | -0.08 (-0.09, -0.07) |
| Fiji | 44.72 (35.34, 55.62) | 44.18 (34.22, 56.47) | -1.20 | 5680.64 (4509.57, 6992.59) | 5487.37 (4352.87, 6722.42) | 4661.92 (3595.01, 5952.45) | 4675.12 (3611.82, 5987.76) | -0.44 (-0.70, -0.18) | 0.00 (-1.44, 1.47) | -1.65 (-2.32, -0.97) | 0.04 (0.00, 0.07) |
| Finland | 21.07 (15.94, 27.16) | 20.05 (15.39, 25.81) | -4.83 | 394.12 (300.38, 515.70) | 423.80 (333.95, 530.43) | 394.18 (299.19, 515.48) | 393.42 (301.25, 506.04) | -0.04 (-0.18, 0.10) | 0.77 (0.41, 1.13) | -0.75 (-1.10, -0.40) | 0.03 (-0.03, 0.08) |
| France | 212.83 (162.60, 277.52) | 218.04 (167.98, 280.43) | 2.45 | 356.34 (271.45, 465.23) | 354.91 (272.87, 461.95) | 351.95 (278.94, 433.62) | 355.06 (272.29, 458.68) | 0.01 (-0.06, 0.08) | -0.03 (-0.05, -0.01) | -0.09 (-0.44, 0.26) | 0.07 (-0.15, 0.30) |
| Gabon | 18.73 (13.68, 24.93) | 38.02 (28.13, 50.49) | 103.04 | 2214.78 (1643.18, 2917.28) | 2181.53 (1608.45, 2867.55) | 2149.12 (1582.94, 2839.54) | 2112.78 (1573.62, 2785.18) | -0.15 (-0.17, -0.13) | -0.17 (-0.26, -0.09) | -0.15 (-0.22, -0.08) | -0.10 (-0.23, 0.03) |
| Gambia | 11.27 (8.09, 15.67) | 27.35 (19.75, 37.50) | 142.61 | 1327.62 (960.48, 1788.90) | 1281.43 (943.95, 1700.55) | 1288.21 (935.26, 1754.30) | 1296.26 (948.22, 1759.23) | 0.07 (0.01, 0.13) | -0.33 (-0.49, -0.17) | 0.04 (-0.29, 0.38) | 0.13 (-0.23, 0.48) |
| Georgia | 296.38 (223.94, 392.18) | 185.43 (139.35, 241.42) | -37.44 | 5268.40 (3948.70, 6947.93) | 5299.40 (3956.31, 6917.20) | 5223.23 (3966.30, 6822.01) | 5210.08 (3909.95, 6833.37) | -0.08 (-0.09, -0.06) | 0.06 (0.04, 0.08) | -0.15 (-0.19, -0.11) | -0.03 (-0.08, 0.02) |
| Germany | 461.40 (352.15, 593.82) | 434.49 (329.90, 557.54) | -5.83 | 529.21 (401.70, 688.60) | 507.05 (385.57, 652.93) | 572.45 (446.44, 717.30) | 530.93 (402.45, 691.69) | 0.15 (-0.02, 0.32) | -0.46 (-0.66, -0.26) | 1.26 (0.70, 1.83) | -0.86 (-1.26, -0.45) |
| Ghana | 436.82 (324.96, 580.03) | 1058.25 (790.76, 1396.48) | 142.26 | 3328.83 (2513.70, 4353.24) | 3383.04 (2597.48, 4348.84) | 3632.26 (2799.88, 4621.39) | 3258.78 (2466.47, 4282.51) | 0.27 (0.16, 0.38) | 0.20 (0.05, 0.36) | 0.75 (0.43, 1.06) | -0.94 (-1.37, -0.50) |
| Greece | 54.55 (41.89, 70.26) | 51.86 (39.17, 66.06) | -4.93 | 514.08 (391.25, 669.37) | 595.59 (495.42, 717.69) | 573.01 (478.29, 685.40) | 525.78 (399.50, 684.30) | 0.21 (-0.04, 0.47) | 1.59 (0.82, 2.37) | -0.41 (-0.59, -0.22) | -0.75 (-1.07, -0.44) |
| Greenland | 0.36 (0.27, 0.47) | 0.30 (0.23, 0.39) | -16.16 | 525.71 (399.35, 682.56) | 519.58 (392.78, 678.16) | 511.29 (389.43, 669.07) | 511.35 (388.03, 665.32) | -0.11 (-0.13, -0.10) | -0.11 (-0.16, -0.07) | -0.18 (-0.22, -0.14) | -0.02 (-0.08, 0.04) |
| Grenada | 3.40 (2.52, 4.46) | 5.00 (3.75, 6.50) | 47.35 | 4544.02 (3416.41, 5915.36) | 4499.44 (3397.10, 5817.85) | 4509.64 (3368.11, 5875.24) | 4515.82 (3365.11, 5859.94) | -0.02 (-0.03, -0.01) | -0.10 (-0.12, -0.09) | 0.03 (-0.01, 0.06) | 0.01 (0.00, 0.03) |
| Guam | 4.18 (3.11, 5.45) | 4.61 (3.49, 6.00) | 10.27 | 2678.56 (2024.42, 3457.62) | 2810.90 (2120.33, 3657.69) | 2789.45 (2085.50, 3585.00) | 2771.20 (2090.58, 3608.10) | 0.08 (0.01, 0.15) | 0.53 (0.48, 0.57) | -0.10 (-0.25, 0.05) | -0.10 (-0.15, -0.05) |
| Guatemala | 247.39 (195.04, 310.66) | 666.42 (498.99, 877.83) | 169.37 | 3672.48 (2888.23, 4610.81) | 3606.42 (2703.89, 4735.10) | 3568.82 (2663.14, 4670.24) | 3580.71 (2694.47, 4658.63) | -0.12 (-0.14, -0.10) | -0.19 (-0.28, -0.10) | -0.11 (-0.15, -0.07) | 0.07 (0.00, 0.13) |
| Guinea | 96.26 (71.65, 127.82) | 204.49 (151.23, 270.64) | 112.43 | 1893.55 (1418.52, 2488.53) | 1888.91 (1439.05, 2465.41) | 1870.66 (1393.08, 2441.06) | 1865.92 (1393.98, 2443.54) | -0.02 (-0.04, -0.01) | -0.01 (-0.07, 0.05) | -0.10 (-0.18, -0.02) | -0.02 (-0.10, 0.07) |
| Guinea-Bissau | 15.92 (11.85, 21.00) | 34.17 (25.17, 45.84) | 114.71 | 1892.36 (1423.12, 2489.39) | 1895.46 (1416.69, 2480.56) | 1868.57 (1389.24, 2434.69) | 1869.80 (1399.21, 2460.94) | -0.03 (-0.05, -0.02) | 0.02 (0.00, 0.05) | -0.15 (-0.21, -0.10) | 0.04 (-0.05, 0.14) |
| Guyana | 35.12 (25.83, 46.82) | 37.62 (28.13, 49.38) | 7.10 | 4587.88 (3423.28, 6064.17) | 4581.82 (3426.42, 5968.49) | 4573.51 (3426.89, 5985.76) | 4583.81 (3419.81, 6020.67) | -0.01 (-0.01, 0.00) | -0.01 (-0.02, -0.01) | -0.02 (-0.04, 0.00) | 0.04 (0.01, 0.07) |
| Haiti | 222.50 (165.42, 290.80) | 508.12 (374.72, 671.92) | 128.37 | 3963.85 (2963.25, 5179.92) | 3949.44 (2955.05, 5138.81) | 3994.07 (2998.14, 5223.90) | 3943.36 (2936.84, 5188.72) | 0.03 (0.01, 0.05) | -0.04 (-0.07, -0.01) | 0.11 (0.07, 0.16) | -0.06 (-0.18, 0.06) |
| Honduras | 145.18 (107.87, 188.73) | 372.45 (279.71, 485.08) | 156.54 | 3787.72 (2851.11, 4922.11) | 3747.97 (2826.64, 4900.08) | 3717.88 (2767.04, 4938.13) | 3723.78 (2801.73, 4815.42) | -0.07 (-0.08, -0.05) | -0.11 (-0.14, -0.08) | -0.08 (-0.09, -0.07) | 0.06 (0.01, 0.12) |
| Hungary | 338.78 (253.62, 443.76) | 303.83 (229.15, 393.64) | -10.32 | 3262.75 (2455.35, 4305.04) | 3245.97 (2418.63, 4223.95) | 3247.83 (2408.71, 4316.34) | 3249.86 (2431.78, 4284.77) | 0.00 (-0.01, 0.01) | -0.05 (-0.07, -0.03) | 0.01 (-0.05, 0.06) | 0.05 (-0.01, 0.12) |
| Iceland | 0.91 (0.69, 1.21) | 1.20 (0.91, 1.56) | 31.89 | 343.32 (262.35, 451.14) | 343.71 (258.78, 450.51) | 343.22 (262.95, 443.34) | 343.40 (259.71, 445.16) | 0.01 (0.00, 0.03) | 0.01 (-0.01, 0.04) | 0.01 (-0.05, 0.06) | 0.06 (0.01, 0.12) |
| India | 12896.03 (9617.02, 17015.70) | 23788.06 (17562.87, 31356.56) | 84.46 | 1618.53 (1200.08, 2120.03) | 1445.10 (1073.56, 1885.47) | 1427.47 (1062.20, 1865.62) | 1589.82 (1175.06, 2081.70) | -0.28 (-0.50, -0.06) | -1.13 (-1.55, -0.70) | -0.15 (-0.50, 0.20) | 1.02 (0.76, 1.28) |
| Indonesia | 7476.29 (5555.31, 9843.10) | 11997.56 (8951.28, 15554.97) | 60.47 | 4199.54 (3155.62, 5455.12) | 4172.43 (3130.33, 5447.90) | 4121.21 (3083.09, 5372.02) | 4135.57 (3076.45, 5360.04) | -0.05 (-0.07, -0.04) | -0.06 (-0.08, -0.03) | -0.13 (-0.14, -0.12) | 0.00 (-0.11, 0.10) |
| Iran (Islamic Republic of) | 1613.52 (1210.96, 2135.18) | 3440.46 (2550.80, 4498.60) | 113.23 | 3307.25 (2493.16, 4319.57) | 4082.43 (3129.20, 5259.30) | 4045.90 (3157.99, 5164.84) | 3409.21 (2563.38, 4466.48) | 0.49 (0.12, 0.86) | 2.25 (1.15, 3.37) | -0.10 (-0.33, 0.12) | -1.55 (-1.98, -1.11) |
| Iraq | 597.21 (464.36, 741.62) | 1484.74 (1095.39, 1963.19) | 148.61 | 3931.07 (3082.39, 4869.09) | 3349.18 (2532.18, 4393.09) | 3393.90 (2590.44, 4341.23) | 3274.53 (2435.62, 4289.07) | -0.61 (-0.77, -0.45) | -1.64 (-2.34, -0.93) | 0.15 (-0.03, 0.34) | -0.24 (-0.44, -0.05) |
| Ireland | 14.25 (10.81, 18.45) | 19.86 (15.20, 25.81) | 39.34 | 405.32 (306.83, 526.90) | 431.25 (339.01, 550.33) | 436.78 (336.83, 551.89) | 406.86 (309.34, 529.60) | 0.06 (-0.13, 0.25) | 0.60 (0.32, 0.88) | 0.13 (-0.25, 0.51) | -0.77 (-1.14, -0.40) |
| Israel | 24.63 (18.71, 31.87) | 40.32 (31.02, 52.16) | 63.69 | 516.12 (394.84, 666.00) | 451.66 (355.71, 572.08) | 454.22 (347.94, 590.42) | 456.03 (351.74, 592.14) | -0.47 (-0.61, -0.33) | -1.36 (-1.90, -0.82) | 0.06 (0.02, 0.10) | 0.01 (-0.05, 0.07) |
| Italy | 256.86 (194.33, 338.70) | 247.37 (187.26, 319.74) | -3.70 | 425.98 (321.17, 562.14) | 432.91 (323.99, 567.36) | 434.31 (327.23, 569.68) | 423.25 (318.66, 558.91) | 0.00 (-0.05, 0.05) | 0.18 (0.10, 0.25) | 0.03 (-0.03, 0.10) | -0.30 (-0.40, -0.20) |
| Jamaica | 99.85 (73.82, 132.85) | 139.76 (104.92, 183.31) | 39.97 | 4508.57 (3359.17, 5922.47) | 4499.77 (3369.44, 5836.15) | 4513.87 (3350.71, 5988.60) | 4496.76 (3369.88, 5888.66) | -0.01 (-0.02, -0.01) | -0.03 (-0.06, 0.01) | 0.03 (0.02, 0.05) | -0.04 (-0.07, -0.01) |
| Japan | 1335.77 (1005.62, 1722.54) | 1134.69 (858.10, 1469.55) | -15.05 | 997.24 (747.72, 1301.23) | 1005.99 (753.12, 1315.82) | 997.11 (748.40, 1305.81) | 997.75 (749.50, 1309.09) | -0.01 (-0.02, 0.00) | 0.09 (0.08, 0.10) | -0.09 (-0.13, -0.06) | 0.02 (-0.03, 0.06) |
| Jordan | 42.35 (31.14, 56.49) | 155.79 (116.02, 206.93) | 267.83 | 1286.40 (946.79, 1702.13) | 1198.97 (890.10, 1568.43) | 1222.33 (913.64, 1611.83) | 1251.93 (936.13, 1662.31) | 0.02 (-0.08, 0.12) | -0.68 (-0.73, -0.63) | 0.19 (0.07, 0.31) | 0.21 (-0.09, 0.50) |
| Kazakhstan | 883.21 (660.06, 1174.63) | 1033.32 (766.49, 1347.89) | 17.00 | 5236.40 (3903.64, 6846.68) | 5284.98 (3963.72, 6857.70) | 5251.56 (3971.38, 6845.36) | 5261.78 (3940.65, 6886.06) | -0.02 (-0.03, -0.01) | 0.09 (0.06, 0.12) | -0.07 (-0.07, -0.06) | 0.01 (-0.04, 0.06) |
| Kenya | 540.98 (403.03, 721.14) | 1456.09 (1087.20, 1929.35) | 169.16 | 3010.60 (2250.16, 3940.38) | 3060.42 (2301.87, 4055.33) | 3152.53 (2380.18, 4143.40) | 3016.81 (2256.53, 3944.27) | 0.11 (0.03, 0.19) | 0.14 (0.09, 0.19) | 0.29 (0.10, 0.49) | -0.63 (-1.04, -0.22) |
| Kiribati | 2.55 (1.93, 3.34) | 4.24 (3.18, 5.39) | 66.27 | 3511.03 (2695.59, 4538.55) | 3748.43 (2935.03, 4646.89) | 3482.68 (2640.21, 4487.12) | 3511.71 (2676.72, 4480.36) | -0.10 (-0.22, 0.02) | 0.68 (0.47, 0.89) | -0.76 (-1.08, -0.44) | 0.05 (-0.01, 0.12) |
| Kuwait | 65.30 (47.59, 88.46) | 171.11 (125.63, 227.57) | 162.03 | 2960.35 (2213.50, 3925.05) | 2850.36 (2164.92, 3689.52) | 2895.64 (2285.60, 3658.86) | 2705.64 (2019.55, 3580.15) | -0.27 (-0.37, -0.18) | -0.35 (-0.47, -0.23) | 0.22 (-0.06, 0.50) | -0.57 (-1.10, -0.04) |
| Kyrgyzstan | 219.36 (161.59, 292.47) | 361.44 (269.04, 476.00) | 64.77 | 5263.90 (3907.20, 6902.28) | 5276.74 (3944.62, 6920.90) | 5246.34 (3934.72, 6886.10) | 5257.67 (3950.64, 6873.81) | -0.02 (-0.02, -0.01) | 0.02 (0.02, 0.03) | -0.06 (-0.07, -0.05) | 0.04 (0.02, 0.06) |
| Lao People's Democratic Republic | 158.61 (117.53, 210.67) | 345.61 (256.99, 459.41) | 117.89 | 4522.64 (3373.91, 5989.93) | 4532.48 (3395.85, 5959.75) | 4478.70 (3350.38, 5818.60) | 4481.40 (3353.14, 5851.13) | -0.02 (-0.04, -0.01) | 0.03 (-0.01, 0.07) | -0.13 (-0.15, -0.11) | -0.03 (-0.16, 0.09) |
| Latvia | 103.13 (76.86, 134.56) | 67.95 (50.48, 88.70) | -34.11 | 3804.61 (2819.93, 4980.09) | 3781.48 (2832.76, 4955.59) | 3779.52 (2813.38, 5012.89) | 3762.79 (2790.16, 4963.56) | -0.01 (-0.02, 0.00) | -0.05 (-0.09, -0.01) | -0.01 (-0.01, 0.00) | -0.03 (-0.06, 0.00) |
| Lebanon | 66.55 (48.36, 90.32) | 128.10 (92.88, 175.06) | 92.48 | 2291.95 (1664.79, 3112.18) | 2238.13 (1649.27, 3018.84) | 2282.43 (1660.67, 3122.08) | 2257.52 (1652.49, 3057.57) | -0.03 (-0.10, 0.03) | -0.27 (-0.48, -0.07) | 0.20 (0.08, 0.32) | -0.24 (-0.51, 0.04) |
| Lesotho | 54.44 (40.82, 71.69) | 76.69 (56.95, 101.19) | 40.87 | 3432.83 (2572.86, 4514.82) | 3408.98 (2564.39, 4385.80) | 3395.54 (2552.42, 4464.54) | 3370.50 (2542.23, 4380.18) | -0.05 (-0.06, -0.05) | -0.07 (-0.09, -0.05) | -0.04 (-0.04, -0.04) | -0.06 (-0.09, -0.03) |
| Liberia | 29.09 (21.64, 37.95) | 86.76 (64.69, 114.56) | 198.24 | 1877.06 (1398.37, 2448.39) | 1857.30 (1411.40, 2447.80) | 1848.72 (1390.02, 2425.12) | 1838.47 (1385.47, 2431.26) | -0.05 (-0.06, -0.03) | -0.11 (-0.12, -0.10) | -0.05 (-0.14, 0.04) | -0.05 (-0.14, 0.04) |
| Libya | 103.96 (76.87, 137.08) | 231.26 (171.10, 303.19) | 122.45 | 2884.04 (2150.57, 3807.72) | 2774.41 (2069.66, 3661.56) | 2780.37 (2081.50, 3615.25) | 2774.15 (2059.56, 3663.71) | -0.09 (-0.13, -0.06) | -0.38 (-0.44, -0.32) | 0.03 (-0.01, 0.06) | -0.10 (-0.19, -0.01) |
| Lithuania | 144.02 (107.25, 189.72) | 98.99 (74.51, 129.31) | -31.27 | 3778.66 (2819.56, 4973.44) | 3785.49 (2827.95, 4968.59) | 3760.05 (2817.40, 4965.28) | 3750.66 (2806.74, 4930.08) | -0.01 (-0.02, 0.00) | 0.03 (-0.03, 0.10) | -0.07 (-0.09, -0.05) | 0.00 (-0.03, 0.04) |
| Luxembourg | 1.09 (0.83, 1.42) | 1.73 (1.30, 2.23) | 59.05 | 259.91 (199.88, 340.78) | 257.67 (196.87, 332.79) | 261.72 (200.42, 339.50) | 260.40 (195.18, 338.96) | 0.01 (-0.03, 0.06) | -0.08 (-0.14, -0.02) | 0.16 (0.00, 0.32) | -0.18 (-0.35, 0.00) |
| Madagascar | 323.52 (240.79, 430.27) | 808.26 (601.18, 1073.74) | 149.84 | 3206.76 (2417.67, 4201.77) | 3211.51 (2422.44, 4211.83) | 3202.35 (2420.45, 4159.15) | 3197.55 (2391.42, 4201.93) | -0.02 (-0.03, -0.01) | 0.00 (-0.05, 0.06) | -0.03 (-0.04, -0.02) | 0.00 (-0.06, 0.06) |
| Malawi | 284.62 (213.08, 370.93) | 564.67 (423.57, 749.12) | 98.40 | 3464.78 (2626.28, 4483.46) | 3386.80 (2557.53, 4429.57) | 3401.20 (2561.85, 4486.87) | 3376.40 (2560.88, 4441.74) | -0.11 (-0.15, -0.08) | -0.26 (-0.45, -0.07) | 0.05 (0.01, 0.09) | -0.06 (-0.10, -0.01) |
| Malaysia | 759.54 (566.31, 1012.86) | 1538.31 (1137.29, 2027.31) | 102.53 | 4324.05 (3237.59, 5602.20) | 4334.83 (3244.72, 5647.11) | 4287.72 (3213.28, 5617.61) | 4314.67 (3228.69, 5624.35) | 0.00 (-0.02, 0.01) | 0.04 (-0.01, 0.09) | -0.12 (-0.16, -0.08) | 0.06 (-0.01, 0.14) |
| Maldives | 7.65 (5.64, 10.27) | 32.01 (23.00, 42.90) | 318.67 | 4330.12 (3208.33, 5682.94) | 4306.07 (3221.19, 5645.50) | 4301.04 (3197.69, 5648.82) | 4582.15 (3417.70, 6066.28) | 0.19 (0.12, 0.26) | -0.03 (-0.11, 0.05) | -0.05 (-0.16, 0.07) | 0.71 (0.58, 0.84) |
| Mali | 148.27 (111.65, 195.86) | 377.68 (281.19, 492.78) | 154.72 | 2126.41 (1617.72, 2797.60) | 2232.39 (1715.12, 2825.08) | 2095.93 (1574.40, 2736.58) | 2076.26 (1560.34, 2708.70) | -0.25 (-0.32, -0.18) | 0.41 (0.18, 0.64) | -0.66 (-0.81, -0.50) | -0.10 (-0.19, -0.01) |
| Malta | 1.36 (1.02, 1.75) | 1.50 (1.14, 1.98) | 10.58 | 348.95 (262.14, 456.99) | 351.25 (268.21, 455.73) | 349.86 (261.31, 453.70) | 351.44 (267.94, 460.67) | 0.02 (0.00, 0.04) | 0.08 (0.02, 0.13) | -0.04 (-0.10, 0.02) | 0.09 (0.02, 0.17) |
| Marshall Islands | 0.33 (0.24, 0.44) | 0.51 (0.38, 0.68) | 54.82 | 866.31 (645.55, 1148.76) | 867.22 (636.07, 1149.36) | 416.74 (345.03, 492.89) | 856.54 (636.09, 1139.14) | -1.47 (-2.68, -0.25) | 0.02 (0.00, 0.05) | -7.67 (-10.27, -5.01) | 9.85 (8.26, 11.48) |
| Mauritania | 33.08 (24.62, 43.62) | 68.39 (51.41, 89.80) | 106.74 | 1890.37 (1420.80, 2472.61) | 1882.33 (1421.58, 2481.63) | 1859.21 (1396.05, 2452.16) | 1845.81 (1397.65, 2430.07) | -0.07 (-0.08, -0.06) | -0.04 (-0.05, -0.04) | -0.13 (-0.19, -0.07) | -0.07 (-0.14, 0.01) |
| Mauritius | 52.69 (38.56, 70.08) | 58.04 (44.04, 75.71) | 10.16 | 4303.80 (3180.68, 5654.72) | 4308.36 (3186.56, 5687.12) | 4234.06 (3164.42, 5507.49) | 4260.97 (3200.29, 5635.34) | -0.04 (-0.06, -0.02) | 0.02 (-0.02, 0.07) | -0.19 (-0.20, -0.17) | 0.04 (-0.06, 0.14) |
| Mexico | 3365.31 (2540.62, 4437.79) | 5656.60 (4259.04, 7352.16) | 68.09 | 4336.15 (3283.10, 5676.05) | 4317.20 (3256.16, 5620.38) | 4660.21 (3547.46, 6071.48) | 4241.57 (3182.86, 5521.75) | 0.27 (0.15, 0.39) | -0.05 (-0.06, -0.04) | 0.79 (0.43, 1.16) | -0.65 (-1.22, -0.08) |
| Micronesia (Federated States of) | 2.64 (1.98, 3.44) | 3.03 (2.29, 3.97) | 14.80 | 2928.24 (2215.72, 3780.65) | 2961.01 (2243.37, 3867.12) | 2906.20 (2180.23, 3735.81) | 2876.78 (2173.43, 3789.81) | -0.10 (-0.13, -0.08) | 0.11 (0.06, 0.16) | -0.19 (-0.25, -0.14) | -0.11 (-0.12, -0.09) |
| Monaco | 0.10 (0.08, 0.13) | 0.11 (0.09, 0.15) | 10.12 | 344.66 (265.57, 451.48) | 347.35 (264.61, 454.05) | 344.37 (262.65, 450.67) | 345.91 (262.87, 459.98) | 0.01 (-0.01, 0.02) | 0.08 (0.05, 0.12) | -0.09 (-0.14, -0.04) | 0.06 (0.04, 0.08) |
| Mongolia | 103.42 (76.40, 136.85) | 202.93 (150.98, 265.80) | 96.22 | 5305.54 (3967.89, 6984.43) | 5725.74 (4523.04, 7120.63) | 5671.66 (4462.44, 7076.82) | 5303.36 (3997.13, 6898.53) | 0.00 (-0.14, 0.13) | 0.80 (0.52, 1.08) | -0.10 (-0.14, -0.05) | -0.78 (-1.12, -0.44) |
| Montenegro | 21.56 (16.03, 28.39) | 20.14 (15.09, 26.28) | -6.57 | 3294.34 (2448.87, 4330.71) | 3255.96 (2401.58, 4259.97) | 3254.78 (2425.45, 4276.26) | 3265.40 (2425.38, 4320.77) | -0.04 (-0.05, -0.02) | -0.12 (-0.16, -0.09) | 0.00 (-0.01, 0.01) | 0.05 (0.02, 0.07) |
| Morocco | 1273.11 (962.60, 1652.51) | 1642.84 (1278.77, 2072.85) | 29.04 | 5290.77 (4063.42, 6737.20) | 4808.76 (3723.47, 6005.16) | 4171.72 (3242.47, 5269.90) | 4229.41 (3292.20, 5333.28) | -1.00 (-1.16, -0.83) | -0.92 (-1.13, -0.70) | -1.42 (-1.87, -0.98) | 0.14 (0.10, 0.18) |
| Mozambique | 353.62 (264.92, 460.74) | 833.29 (621.25, 1100.34) | 135.65 | 3329.80 (2503.27, 4324.98) | 3499.29 (2727.02, 4428.63) | 3336.35 (2516.88, 4348.90) | 3324.73 (2500.84, 4364.79) | -0.24 (-0.32, -0.15) | 0.39 (-0.10, 0.88) | -0.48 (-0.68, -0.28) | -0.02 (-0.05, 0.02) |
| Myanmar | 1738.25 (1289.69, 2271.44) | 2538.15 (1893.51, 3329.08) | 46.02 | 4477.21 (3350.93, 5840.98) | 4459.53 (3337.11, 5836.02) | 4377.45 (3241.68, 5733.87) | 4382.65 (3267.81, 5748.38) | -0.07 (-0.08, -0.05) | -0.03 (-0.07, 0.01) | -0.20 (-0.21, -0.19) | 0.01 (-0.09, 0.11) |
| Namibia | 42.93 (31.82, 56.28) | 83.33 (61.80, 108.61) | 94.10 | 3418.57 (2567.89, 4459.75) | 3404.06 (2586.71, 4447.07) | 3379.99 (2534.37, 4399.56) | 3356.62 (2502.88, 4366.56) | -0.05 (-0.06, -0.05) | -0.04 (-0.05, -0.03) | -0.07 (-0.09, -0.05) | -0.05 (-0.09, 0.00) |
| Nauru | 0.28 (0.21, 0.36) | 0.32 (0.24, 0.43) | 15.24 | 2859.00 (2157.54, 3691.36) | 2901.36 (2206.56, 3809.42) | 2906.56 (2218.20, 3802.33) | 2921.52 (2222.21, 3818.26) | 0.02 (0.00, 0.04) | 0.12 (-0.01, 0.24) | 0.02 (-0.04, 0.08) | 0.03 (0.00, 0.06) |
| Nepal | 323.88 (241.52, 428.76) | 583.42 (438.73, 771.93) | 80.14 | 1900.15 (1421.28, 2495.93) | 1885.00 (1388.45, 2482.38) | 1882.14 (1405.13, 2460.61) | 1861.75 (1398.94, 2455.92) | -0.07 (-0.08, -0.06) | -0.09 (-0.13, -0.05) | -0.01 (-0.02, -0.01) | -0.14 (-0.17, -0.11) |
| Netherlands | 79.42 (61.60, 103.39) | 67.51 (51.16, 86.86) | -14.99 | 482.17 (374.57, 624.01) | 446.39 (366.75, 531.36) | 429.23 (345.19, 521.24) | 410.71 (311.28, 527.42) | -0.26 (-0.40, -0.11) | -0.53 (-1.55, 0.50) | -0.37 (-0.51, -0.24) | -0.51 (-0.72, -0.30) |
| New Zealand | 61.90 (46.26, 81.16) | 72.83 (55.10, 93.07) | 17.67 | 1709.38 (1281.79, 2236.25) | 1169.03 (882.13, 1521.33) | 1359.99 (1027.83, 1753.28) | 1716.11 (1287.05, 2215.45) | 0.08 (-0.53, 0.70) | -3.74 (-5.34, -2.10) | 1.62 (1.20, 2.04) | 2.75 (1.57, 3.95) |
| Nicaragua | 128.48 (98.77, 166.03) | 259.28 (193.78, 339.65) | 101.80 | 3931.32 (3019.94, 5048.18) | 3772.21 (2828.09, 4915.38) | 3734.97 (2805.86, 4843.38) | 3725.17 (2798.86, 4835.15) | -0.21 (-0.25, -0.17) | -0.43 (-0.58, -0.27) | -0.10 (-0.11, -0.08) | 0.00 (-0.05, 0.05) |
| Niger | 119.44 (89.55, 158.12) | 323.19 (239.57, 432.25) | 170.60 | 1878.88 (1419.30, 2459.59) | 1863.64 (1408.34, 2427.92) | 1833.67 (1382.77, 2399.71) | 1830.70 (1367.94, 2412.83) | -0.12 (-0.14, -0.09) | -0.11 (-0.24, 0.02) | -0.17 (-0.29, -0.05) | -0.01 (-0.15, 0.12) |
| Nigeria | 1884.09 (1411.02, 2496.50) | 4800.80 (3612.23, 6354.47) | 154.81 | 2467.24 (1836.07, 3250.30) | 2520.30 (1883.31, 3307.41) | 3006.96 (2265.26, 3943.57) | 2604.23 (1947.23, 3411.43) | 0.81 (0.58, 1.03) | 0.20 (0.14, 0.26) | 1.85 (1.01, 2.69) | -0.92 (-1.98, 0.16) |
| Niue | 0.06 (0.04, 0.08) | 0.05 (0.04, 0.06) | -22.15 | 2896.52 (2199.61, 3801.15) | 2833.50 (2137.53, 3686.65) | 2813.03 (2139.50, 3663.20) | 2906.23 (2215.43, 3797.45) | -0.03 (-0.09, 0.03) | -0.22 (-0.25, -0.18) | -0.10 (-0.22, 0.01) | 0.36 (0.34, 0.38) |
| North Macedonia | 70.86 (52.91, 93.86) | 76.99 (57.47, 101.28) | 8.66 | 3326.84 (2487.43, 4390.00) | 3301.68 (2461.49, 4348.99) | 3294.79 (2460.53, 4342.39) | 3320.76 (2484.12, 4366.37) | -0.01 (-0.02, 0.00) | -0.05 (-0.08, -0.02) | -0.03 (-0.06, 0.01) | 0.10 (0.07, 0.13) |
| Northern Mariana Islands | 1.63 (1.20, 2.16) | 1.18 (0.91, 1.51) | -27.37 | 2738.89 (2045.44, 3582.99) | 3178.34 (2367.90, 4122.20) | 2910.57 (2195.35, 3777.03) | 2781.47 (2097.40, 3601.87) | -0.07 (-0.23, 0.10) | 1.25 (0.86, 1.65) | -0.77 (-1.11, -0.42) | -0.32 (-0.60, -0.04) |
| Norway | 27.89 (20.93, 36.12) | 34.56 (26.32, 44.23) | 23.91 | 628.26 (470.58, 815.45) | 569.34 (429.10, 745.59) | 636.59 (481.46, 839.32) | 632.95 (475.20, 820.10) | 0.16 (0.03, 0.30) | -1.03 (-1.42, -0.64) | 1.12 (0.64, 1.61) | -0.06 (-0.07, -0.04) |
| Oman | 57.53 (42.08, 77.35) | 202.88 (145.55, 280.43) | 252.67 | 2957.11 (2211.76, 3897.92) | 2868.17 (2129.17, 3756.44) | 2869.82 (2157.52, 3738.31) | 2930.24 (2184.62, 3817.97) | 0.00 (-0.07, 0.07) | -0.29 (-0.34, -0.23) | -0.05 (-0.17, 0.08) | 0.11 (-0.13, 0.35) |
| Pakistan | 2272.32 (1709.78, 3013.01) | 5040.58 (3777.26, 6634.96) | 121.82 | 2501.59 (1878.96, 3264.86) | 2670.55 (2004.53, 3475.35) | 2359.75 (1757.62, 3089.31) | 2432.88 (1815.70, 3174.95) | -0.24 (-0.40, -0.08) | 0.68 (0.44, 0.93) | -1.29 (-1.78, -0.79) | 0.34 (0.19, 0.50) |
| Palau | 0.48 (0.36, 0.63) | 0.49 (0.37, 0.62) | 1.66 | 2863.82 (2141.50, 3751.56) | 2621.13 (1985.47, 3414.03) | 2727.77 (2050.36, 3565.96) | 2532.75 (1905.69, 3256.62) | -0.37 (-0.49, -0.24) | -0.99 (-1.48, -0.49) | 0.54 (0.41, 0.67) | -0.94 (-1.10, -0.78) |
| Palestine | 45.95 (33.54, 61.01) | 135.98 (100.91, 178.69) | 195.92 | 2788.43 (2057.78, 3633.32) | 2774.61 (2088.73, 3608.08) | 2774.36 (2085.06, 3657.45) | 2757.15 (2047.24, 3591.60) | -0.03 (-0.04, -0.02) | -0.05 (-0.07, -0.03) | 0.00 (-0.02, 0.02) | -0.09 (-0.17, -0.01) |
| Panama | 87.17 (65.20, 115.05) | 152.93 (115.45, 200.89) | 75.43 | 3655.87 (2759.14, 4739.39) | 3647.37 (2707.18, 4768.98) | 3635.59 (2751.44, 4730.77) | 3615.41 (2728.06, 4741.32) | -0.04 (-0.05, -0.04) | -0.02 (-0.03, -0.01) | -0.03 (-0.04, -0.03) | -0.07 (-0.11, -0.04) |
| Papua New Guinea | 168.28 (128.30, 211.67) | 375.63 (282.32, 489.74) | 123.22 | 4269.37 (3306.07, 5369.61) | 3755.57 (2813.60, 4830.38) | 3673.76 (2929.33, 4472.88) | 3740.93 (2851.38, 4812.86) | -0.60 (-0.75, -0.46) | -1.31 (-1.91, -0.71) | -0.23 (-0.25, -0.21) | 0.14 (0.03, 0.25) |
| Paraguay | 160.54 (120.61, 212.51) | 324.14 (240.92, 425.93) | 101.90 | 4380.88 (3307.30, 5751.60) | 4340.34 (3274.22, 5651.33) | 4324.25 (3236.15, 5663.92) | 4344.16 (3242.74, 5687.15) | -0.04 (-0.05, -0.03) | -0.09 (-0.11, -0.08) | -0.04 (-0.04, -0.03) | 0.00 (-0.06, 0.07) |
| Peru | 570.31 (422.20, 746.12) | 1005.85 (746.85, 1312.68) | 76.37 | 2828.69 (2094.39, 3682.69) | 2943.77 (2296.75, 3683.08) | 3045.50 (2325.22, 3881.73) | 2791.23 (2074.72, 3633.04) | 0.26 (0.17, 0.35) | 0.41 (0.23, 0.60) | 0.35 (0.20, 0.50) | -0.51 (-1.15, 0.13) |
| Philippines | 2632.71 (1959.58, 3485.41) | 5145.98 (3830.78, 6798.81) | 95.46 | 4582.02 (3417.47, 5980.36) | 4617.56 (3455.48, 6039.71) | 4523.57 (3372.74, 5933.22) | 4512.30 (3358.85, 5905.58) | -0.04 (-0.07, -0.02) | 0.10 (-0.01, 0.20) | -0.22 (-0.23, -0.21) | -0.03 (-0.10, 0.04) |
| Poland | 1321.35 (979.40, 1717.41) | 1314.71 (970.81, 1719.41) | -0.50 | 3294.27 (2435.22, 4339.48) | 3275.21 (2419.49, 4307.01) | 3269.97 (2416.34, 4282.57) | 3250.32 (2412.59, 4270.63) | -0.03 (-0.04, -0.03) | -0.05 (-0.07, -0.03) | -0.02 (-0.03, 0.00) | -0.05 (-0.08, -0.02) |
| Portugal | 46.61 (35.74, 60.55) | 46.10 (35.65, 58.88) | -1.11 | 452.66 (344.97, 592.66) | 484.17 (380.69, 601.09) | 475.16 (366.08, 610.53) | 451.18 (345.81, 583.96) | 0.01 (-0.14, 0.17) | 0.69 (0.33, 1.06) | -0.19 (-0.39, 0.00) | -0.58 (-0.82, -0.33) |
| Puerto Rico | 161.05 (120.71, 210.17) | 146.73 (110.57, 192.59) | -8.89 | 4415.10 (3308.12, 5754.39) | 4405.99 (3303.27, 5742.69) | 4421.32 (3289.70, 5771.59) | 4432.00 (3320.14, 5865.00) | 0.00 (-0.01, 0.01) | -0.03 (-0.05, 0.00) | 0.04 (0.00, 0.07) | 0.00 (-0.05, 0.05) |
| Qatar | 19.95 (14.40, 26.82) | 150.72 (107.60, 203.97) | 655.60 | 3154.52 (2352.61, 4165.61) | 3086.74 (2308.16, 4055.24) | 3207.31 (2429.12, 4155.92) | 3076.71 (2272.16, 4044.12) | 0.05 (-0.01, 0.11) | -0.21 (-0.25, -0.17) | 0.40 (0.24, 0.57) | -0.42 (-0.49, -0.36) |
| Republic of Korea | 520.94 (388.61, 687.45) | 607.26 (454.18, 786.81) | 16.57 | 1011.99 (765.86, 1313.84) | 1085.28 (817.38, 1411.78) | 1083.22 (861.38, 1320.72) | 1073.54 (797.57, 1418.66) | 0.23 (0.10, 0.37) | 0.69 (0.36, 1.01) | -0.07 (-0.36, 0.23) | -0.30 (-0.58, -0.03) |
| Republic of Moldova | 176.90 (129.67, 232.98) | 153.55 (113.61, 201.29) | -13.20 | 3840.91 (2829.02, 5035.11) | 3825.71 (2838.37, 4996.81) | 3815.38 (2824.49, 4995.82) | 3812.03 (2847.32, 4991.93) | -0.01 (-0.02, 0.00) | -0.03 (-0.08, 0.02) | -0.03 (-0.06, 0.00) | 0.01 (-0.01, 0.04) |
| Romania | 760.27 (575.98, 986.88) | 602.25 (450.60, 777.61) | -20.78 | 3307.37 (2492.33, 4323.57) | 3317.77 (2488.74, 4412.09) | 3324.27 (2462.73, 4414.56) | 3313.16 (2482.83, 4339.35) | -0.01 (-0.02, 0.01) | 0.02 (-0.01, 0.05) | 0.02 (-0.03, 0.07) | -0.02 (-0.09, 0.05) |
| Russian Federation | 5880.76 (4364.22, 7686.09) | 5573.54 (4115.87, 7333.08) | -5.22 | 3626.26 (2702.95, 4725.44) | 3640.05 (2715.96, 4752.33) | 3610.66 (2695.74, 4686.71) | 3603.24 (2689.22, 4700.07) | -0.01 (-0.02, 0.00) | 0.05 (-0.01, 0.12) | -0.09 (-0.12, -0.05) | 0.02 (-0.03, 0.06) |
| Rwanda | 184.76 (135.95, 248.59) | 378.50 (279.27, 494.85) | 104.87 | 3067.02 (2310.98, 4039.48) | 3034.65 (2266.51, 3966.28) | 3009.12 (2279.32, 3906.60) | 3030.80 (2260.76, 3961.07) | -0.06 (-0.09, -0.04) | -0.17 (-0.24, -0.09) | -0.09 (-0.13, -0.05) | 0.08 (0.08, 0.09) |
| Saint Kitts and Nevis | 1.73 (1.28, 2.33) | 2.95 (2.22, 3.84) | 70.73 | 4494.60 (3400.38, 5915.93) | 4508.41 (3350.06, 5945.09) | 4482.24 (3361.29, 5793.10) | 4505.30 (3358.99, 5909.77) | -0.01 (-0.02, -0.01) | 0.03 (0.03, 0.04) | -0.06 (-0.07, -0.06) | 0.05 (0.01, 0.09) |
| Saint Lucia | 5.64 (4.20, 7.43) | 8.71 (6.52, 11.21) | 54.52 | 4502.97 (3378.82, 5858.74) | 4509.10 (3385.84, 5918.66) | 4499.64 (3346.97, 5864.19) | 4499.48 (3349.91, 5868.95) | -0.02 (-0.03, -0.02) | 0.01 (-0.02, 0.04) | -0.02 (-0.02, -0.02) | -0.02 (-0.05, 0.01) |
| Saint Vincent and the Grenadines | 4.52 (3.32, 6.00) | 5.27 (3.92, 6.86) | 16.67 | 4506.62 (3345.33, 5925.90) | 4505.49 (3368.22, 5888.13) | 4509.22 (3370.72, 5962.78) | 4521.82 (3348.43, 5892.44) | -0.02 (-0.03, -0.01) | -0.01 (-0.06, 0.03) | 0.01 (0.00, 0.02) | 0.02 (-0.04, 0.09) |
| Samoa | 6.05 (4.60, 7.89) | 8.39 (6.45, 10.77) | 38.64 | 4126.81 (3148.64, 5351.51) | 4832.64 (3859.47, 5973.28) | 4563.93 (3507.14, 5692.60) | 4169.58 (3196.03, 5335.12) | 0.19 (-0.09, 0.46) | 1.69 (0.93, 2.45) | -0.60 (-0.85, -0.34) | -1.26 (-1.93, -0.57) |
| San Marino | 0.09 (0.07, 0.11) | 0.11 (0.09, 0.15) | 30.40 | 349.37 (267.39, 452.94) | 348.06 (265.21, 454.50) | 342.68 (262.44, 447.30) | 342.82 (262.69, 448.84) | -0.07 (-0.08, -0.05) | -0.03 (-0.07, 0.01) | -0.16 (-0.22, -0.10) | 0.03 (0.00, 0.06) |
| Sao Tome and Principe | 1.75 (1.31, 2.31) | 3.78 (2.81, 4.99) | 115.43 | 1851.25 (1383.00, 2432.23) | 1846.94 (1383.37, 2426.59) | 1832.93 (1371.23, 2408.54) | 1831.47 (1373.99, 2394.09) | -0.03 (-0.04, -0.02) | -0.02 (-0.03, -0.01) | -0.08 (-0.16, 0.00) | -0.02 (-0.09, 0.06) |
| Saudi Arabia | 706.71 (536.88, 902.39) | 1830.20 (1424.63, 2295.60) | 158.97 | 4451.69 (3439.99, 5621.60) | 3944.93 (2992.18, 5159.75) | 3865.03 (3045.94, 4851.50) | 3694.69 (2897.17, 4629.89) | -0.38 (-0.48, -0.28) | -1.10 (-1.56, -0.64) | -0.20 (-0.57, 0.17) | -0.57 (-0.75, -0.38) |
| Senegal | 86.24 (64.84, 111.78) | 153.39 (112.12, 203.06) | 77.88 | 1351.81 (1018.44, 1743.19) | 756.50 (591.66, 927.94) | 1100.50 (822.54, 1445.46) | 1086.52 (806.92, 1427.91) | -0.52 (-1.42, 0.38) | -5.59 (-8.00, -3.13) | 4.06 (2.37, 5.77) | -0.13 (-0.16, -0.10) |
| Serbia | 315.84 (233.89, 416.59) | 275.15 (207.79, 357.84) | -12.88 | 3311.34 (2448.32, 4360.46) | 3277.11 (2455.19, 4341.86) | 3270.20 (2425.72, 4302.03) | 3285.01 (2456.71, 4296.91) | -0.04 (-0.06, -0.03) | -0.13 (-0.18, -0.08) | -0.02 (-0.06, 0.02) | 0.07 (0.02, 0.11) |
| Seychelles | 3.12 (2.31, 4.17) | 4.97 (3.69, 6.54) | 59.35 | 4317.75 (3257.91, 5682.36) | 4291.13 (3241.55, 5634.62) | 4339.30 (3231.06, 5701.63) | 4362.37 (3236.75, 5750.68) | 0.07 (0.05, 0.08) | -0.07 (-0.10, -0.04) | 0.11 (0.07, 0.15) | -0.01 (-0.14, 0.12) |
| Sierra Leone | 60.00 (44.62, 79.23) | 147.64 (108.94, 196.62) | 146.05 | 1874.45 (1408.90, 2464.63) | 1882.47 (1403.49, 2486.32) | 1847.02 (1385.81, 2430.85) | 1849.72 (1375.71, 2422.99) | -0.06 (-0.08, -0.04) | 0.05 (0.03, 0.07) | -0.20 (-0.28, -0.12) | 0.00 (-0.07, 0.08) |
| Singapore | 40.52 (30.26, 53.90) | 70.60 (52.57, 91.21) | 74.25 | 1054.26 (797.15, 1387.93) | 1069.11 (807.51, 1406.19) | 1074.12 (802.80, 1417.94) | 1057.41 (795.01, 1378.40) | 0.03 (0.00, 0.05) | 0.18 (0.13, 0.22) | 0.08 (-0.05, 0.22) | -0.16 (-0.24, -0.09) |
| Slovakia | 178.20 (133.58, 232.81) | 184.13 (136.92, 238.87) | 3.33 | 3272.55 (2459.74, 4255.24) | 3259.44 (2419.65, 4292.35) | 3260.78 (2414.90, 4317.25) | 3265.41 (2417.28, 4285.84) | -0.02 (-0.02, -0.01) | -0.04 (-0.06, -0.03) | 0.01 (0.00, 0.01) | 0.02 (-0.02, 0.07) |
| Slovenia | 53.69 (39.47, 71.34) | 49.49 (36.56, 65.07) | -7.82 | 2544.88 (1872.43, 3368.21) | 2441.02 (1889.91, 3104.94) | 2562.40 (1916.61, 3382.18) | 2561.58 (1898.13, 3386.45) | 0.07 (-0.01, 0.14) | -0.44 (-0.60, -0.28) | 0.51 (0.35, 0.67) | -0.03 (-0.06, 0.00) |
| Solomon Islands | 6.89 (5.12, 9.10) | 15.32 (11.43, 20.08) | 122.27 | 2393.57 (1785.55, 3097.60) | 2365.20 (1824.35, 3003.40) | 2354.60 (1820.84, 2973.42) | 2406.58 (1812.74, 3143.82) | 0.00 (-0.02, 0.03) | -0.12 (-0.14, -0.10) | -0.04 (-0.05, -0.02) | 0.16 (0.02, 0.29) |
| Somalia | 265.39 (201.51, 341.98) | 727.69 (540.34, 951.04) | 174.20 | 4573.37 (3470.88, 5913.35) | 4114.83 (3110.41, 5323.29) | 3826.97 (2866.27, 4968.51) | 4149.90 (3101.16, 5401.42) | -0.58 (-0.78, -0.37) | -1.15 (-2.05, -0.25) | -0.74 (-1.12, -0.36) | 1.02 (0.56, 1.48) |
| South Africa | 2331.89 (1764.83, 3052.87) | 3946.85 (2957.90, 5179.24) | 69.26 | 6425.29 (4877.13, 8329.74) | 7020.49 (5465.35, 8992.58) | 7227.47 (5589.70, 9298.38) | 6288.36 (4768.34, 8191.41) | 0.11 (-0.07, 0.29) | 0.74 (0.33, 1.16) | 0.34 (-0.11, 0.79) | -1.02 (-1.76, -0.27) |
| South Sudan | 169.44 (126.02, 225.69) | 243.77 (184.52, 320.52) | 43.87 | 3291.26 (2479.29, 4302.61) | 3260.67 (2465.87, 4288.71) | 3229.81 (2418.30, 4229.68) | 3161.88 (2382.35, 4193.59) | -0.13 (-0.15, -0.12) | -0.10 (-0.14, -0.06) | -0.10 (-0.16, -0.04) | -0.24 (-0.28, -0.21) |
| Spain | 179.41 (135.45, 232.81) | 210.10 (158.27, 269.97) | 17.10 | 452.01 (341.74, 586.07) | 448.91 (341.13, 589.37) | 481.63 (369.96, 616.72) | 454.89 (343.08, 592.91) | 0.22 (0.12, 0.32) | -0.06 (-0.14, 0.01) | 0.75 (0.59, 0.90) | -0.74 (-1.01, -0.48) |
| Sri Lanka | 775.23 (571.73, 1022.87) | 936.46 (697.18, 1224.51) | 20.80 | 4290.73 (3195.85, 5617.98) | 4277.48 (3213.90, 5605.51) | 4189.68 (3114.97, 5529.02) | 4203.73 (3127.19, 5525.07) | -0.07 (-0.09, -0.05) | -0.02 (-0.08, 0.05) | -0.22 (-0.24, -0.20) | 0.00 (-0.14, 0.14) |
| Sudan | 642.93 (484.03, 846.59) | 1653.51 (1269.02, 2097.73) | 157.18 | 3726.96 (2832.15, 4902.70) | 3973.66 (3029.98, 5129.94) | 3995.45 (3125.14, 5065.32) | 4038.57 (3140.86, 5133.56) | 0.08 (-0.14, 0.30) | 0.53 (-0.01, 1.07) | 0.06 (-1.09, 1.22) | 0.08 (-0.08, 0.24) |
| Suriname | 17.48 (13.03, 22.96) | 26.94 (20.36, 34.89) | 54.14 | 4591.79 (3410.42, 5984.23) | 4597.76 (3474.00, 5989.01) | 4583.83 (3405.35, 6030.25) | 4583.96 (3436.31, 5979.96) | -0.02 (-0.03, -0.01) | 0.01 (0.01, 0.01) | -0.03 (-0.04, -0.02) | -0.02 (-0.06, 0.02) |
| Sweden | 73.40 (55.72, 94.45) | 80.63 (61.33, 104.80) | 9.85 | 832.02 (629.68, 1086.76) | 833.33 (634.03, 1081.59) | 728.55 (550.90, 939.33) | 818.52 (615.42, 1071.54) | -0.25 (-0.47, -0.03) | 0.01 (-0.01, 0.03) | -1.38 (-1.90, -0.86) | 1.38 (0.82, 1.94) |
| Switzerland | 24.93 (19.07, 32.21) | 28.90 (21.88, 37.14) | 15.90 | 329.21 (251.96, 426.42) | 331.47 (253.17, 433.26) | 324.76 (247.03, 429.25) | 325.95 (248.83, 424.22) | -0.04 (-0.06, -0.02) | 0.08 (0.00, 0.16) | -0.22 (-0.24, -0.21) | 0.03 (-0.05, 0.11) |
| Syrian Arab Republic | 297.42 (219.84, 392.97) | 368.53 (278.12, 474.20) | 23.91 | 2850.55 (2126.39, 3725.84) | 2781.38 (2071.00, 3622.13) | 2796.64 (2084.38, 3679.75) | 2692.63 (2011.09, 3543.57) | -0.13 (-0.17, -0.10) | -0.25 (-0.26, -0.23) | 0.07 (0.05, 0.09) | -0.49 (-0.56, -0.41) |
| Taiwan (Province of China) | 1273.78 (966.29, 1661.60) | 1317.20 (994.48, 1696.25) | 3.41 | 5547.68 (4259.89, 7176.32) | 5229.73 (3903.55, 6797.21) | 5184.40 (3865.33, 6796.49) | 5195.14 (3874.01, 6716.90) | -0.12 (-0.17, -0.07) | -0.52 (-0.77, -0.27) | -0.09 (-0.10, -0.08) | -0.02 (-0.10, 0.06) |
| Tajikistan | 248.20 (181.80, 331.29) | 531.81 (399.47, 700.69) | 114.27 | 5337.70 (4022.29, 6946.46) | 5385.65 (4072.26, 7100.83) | 5346.87 (4014.00, 6949.81) | 5315.23 (4047.84, 6938.06) | -0.03 (-0.05, -0.02) | 0.10 (0.08, 0.11) | -0.07 (-0.11, -0.04) | -0.07 (-0.09, -0.04) |
| Thailand | 3061.71 (2337.19, 3967.58) | 3513.56 (2676.56, 4534.16) | 14.76 | 4897.23 (3738.47, 6296.60) | 4396.14 (3319.00, 5745.48) | 4829.23 (3672.39, 6211.15) | 4628.26 (3503.29, 6033.98) | 0.16 (0.02, 0.30) | -0.96 (-1.44, -0.47) | 0.99 (0.90, 1.08) | -0.52 (-0.67, -0.37) |
| Timor-Leste | 33.75 (25.09, 44.77) | 52.48 (39.27, 68.96) | 55.49 | 4544.76 (3406.40, 5973.42) | 4540.85 (3387.88, 5901.44) | 4425.48 (3285.87, 5861.47) | 4397.77 (3292.54, 5728.82) | -0.12 (-0.14, -0.10) | 0.00 (-0.03, 0.04) | -0.27 (-0.29, -0.26) | -0.08 (-0.16, 0.00) |
| Togo | 57.40 (42.21, 77.32) | 141.21 (104.94, 185.84) | 146.00 | 1882.92 (1396.63, 2484.22) | 1878.17 (1400.11, 2449.48) | 1857.69 (1392.21, 2434.19) | 1850.41 (1389.12, 2423.35) | -0.04 (-0.05, -0.02) | -0.02 (-0.05, 0.01) | -0.11 (-0.22, -0.01) | -0.02 (-0.14, 0.09) |
| Tokelau | 0.04 (0.03, 0.05) | 0.04 (0.03, 0.05) | -8.96 | 3016.21 (2284.38, 3925.26) | 3030.01 (2294.06, 3905.45) | 2868.54 (2183.92, 3709.16) | 2871.43 (2174.62, 3772.84) | -0.26 (-0.32, -0.20) | 0.05 (-0.02, 0.12) | -0.75 (-0.98, -0.52) | -0.06 (-0.20, 0.08) |
| Tonga | 2.79 (2.12, 3.62) | 3.18 (2.40, 4.09) | 14.00 | 3351.15 (2533.61, 4383.44) | 3624.17 (2835.26, 4491.95) | 3612.38 (2815.10, 4503.18) | 3346.95 (2539.02, 4336.33) | 0.01 (-0.16, 0.18) | 0.82 (0.39, 1.25) | -0.03 (-0.04, -0.02) | -0.94 (-1.42, -0.45) |
| Trinidad and Tobago | 55.16 (40.85, 72.89) | 66.33 (49.27, 85.87) | 20.26 | 4493.55 (3349.46, 5859.06) | 4494.91 (3405.52, 5906.96) | 4501.26 (3391.95, 5936.91) | 4494.37 (3343.76, 5847.70) | -0.01 (-0.02, -0.01) | -0.01 (-0.05, 0.04) | 0.02 (0.00, 0.03) | -0.03 (-0.05, -0.02) |
| Tunisia | 233.46 (173.23, 307.62) | 354.18 (264.50, 463.16) | 51.71 | 2931.86 (2193.63, 3833.81) | 2739.41 (2038.45, 3606.39) | 3021.00 (2309.08, 3925.52) | 2818.72 (2117.08, 3692.40) | 0.22 (0.08, 0.36) | -0.64 (-0.83, -0.44) | 1.08 (1.01, 1.15) | -0.94 (-1.44, -0.44) |
| Turkey | 1984.46 (1512.20, 2547.18) | 2735.34 (2049.59, 3624.36) | 37.84 | 3366.58 (2577.54, 4287.00) | 3023.16 (2394.33, 3832.70) | 3149.03 (2380.77, 4094.34) | 2911.83 (2190.35, 3849.98) | -0.30 (-0.43, -0.17) | -1.05 (-1.16, -0.94) | 0.46 (-0.10, 1.02) | -0.84 (-1.06, -0.63) |
| Turkmenistan | 180.97 (131.84, 240.61) | 281.56 (210.67, 369.80) | 55.58 | 5288.38 (3981.60, 6895.64) | 5307.77 (4002.08, 6943.84) | 5209.73 (3935.27, 6847.12) | 5194.44 (3893.41, 6768.21) | -0.11 (-0.12, -0.09) | 0.03 (0.01, 0.06) | -0.19 (-0.22, -0.16) | -0.03 (-0.08, 0.02) |
| Tuvalu | 0.28 (0.22, 0.37) | 0.33 (0.25, 0.43) | 17.42 | 3162.72 (2401.49, 4085.97) | 3008.93 (2269.85, 3948.20) | 2855.99 (2168.12, 3751.07) | 2776.67 (2103.98, 3564.26) | -0.49 (-0.50, -0.47) | -0.50 (-0.53, -0.47) | -0.52 (-0.55, -0.50) | -0.34 (-0.38, -0.29) |
| Uganda | 489.59 (368.52, 640.44) | 1106.80 (821.03, 1447.71) | 126.07 | 3434.02 (2608.88, 4438.16) | 3375.06 (2638.54, 4204.41) | 3047.59 (2314.61, 3936.38) | 3139.76 (2348.88, 4094.73) | -0.46 (-0.55, -0.37) | -0.18 (-0.33, -0.02) | -1.11 (-1.18, -1.03) | 0.36 (0.10, 0.62) |
| Ukraine | 1946.57 (1468.96, 2533.36) | 1714.42 (1275.28, 2271.95) | -11.93 | 3624.45 (2716.70, 4702.26) | 3631.68 (2703.58, 4731.24) | 3609.55 (2684.20, 4729.22) | 3623.12 (2701.08, 4779.36) | 0.00 (-0.02, 0.01) | 0.03 (-0.01, 0.06) | -0.07 (-0.14, 0.00) | 0.03 (-0.01, 0.06) |
| United Arab Emirates | 74.15 (53.57, 101.15) | 453.27 (320.53, 612.09) | 511.28 | 2909.72 (2152.71, 3860.91) | 2874.25 (2123.38, 3741.44) | 2924.54 (2171.83, 3856.56) | 2889.64 (2140.49, 3832.58) | 0.03 (0.01, 0.06) | -0.12 (-0.14, -0.09) | 0.17 (0.12, 0.22) | -0.14 (-0.24, -0.04) |
| United Kingdom | 176.20 (132.79, 230.14) | 200.82 (152.48, 261.14) | 13.98 | 295.56 (223.60, 385.85) | 295.20 (224.03, 387.02) | 258.31 (197.81, 340.19) | 297.34 (224.72, 390.12) | -0.51 (-0.69, -0.34) | 0.02 (-0.02, 0.06) | -1.40 (-1.72, -1.07) | 1.00 (0.12, 1.89) |
| United Republic of Tanzania | 755.25 (573.10, 973.92) | 1644.97 (1229.78, 2171.04) | 117.81 | 3488.26 (2655.42, 4443.00) | 3085.41 (2346.85, 3930.40) | 3403.00 (2574.96, 4410.52) | 3242.76 (2450.69, 4238.36) | -0.11 (-0.32, 0.10) | -1.30 (-1.95, -0.65) | 1.03 (0.64, 1.43) | -0.28 (-0.58, 0.01) |
| United States of America | 2024.19 (1512.77, 2666.30) | 2522.83 (1914.88, 3252.00) | 24.63 | 735.16 (551.02, 967.82) | 925.06 (700.23, 1215.28) | 686.45 (519.97, 894.89) | 784.76 (589.23, 1024.83) | -0.64 (-0.98, -0.29) | 2.32 (1.98, 2.66) | -3.16 (-3.68, -2.63) | 1.11 (0.53, 1.70) |
| United States Virgin Islands | 4.83 (3.64, 6.30) | 4.08 (3.10, 5.32) | -15.58 | 4465.00 (3348.06, 5812.31) | 4474.24 (3372.99, 5888.57) | 4461.18 (3332.18, 5820.56) | 4456.94 (3338.17, 5845.31) | -0.03 (-0.04, -0.02) | 0.02 (-0.02, 0.05) | -0.03 (-0.05, -0.01) | -0.02 (-0.06, 0.02) |
| Uruguay | 27.23 (20.48, 35.47) | 31.03 (23.52, 39.79) | 13.98 | 901.63 (675.89, 1180.43) | 902.23 (682.82, 1178.54) | 903.24 (676.35, 1174.58) | 904.47 (681.01, 1176.93) | 0.00 (-0.01, 0.00) | 0.01 (0.00, 0.01) | 0.01 (-0.02, 0.05) | 0.00 (-0.05, 0.05) |
| Uzbekistan | 1002.60 (737.89, 1341.51) | 1948.32 (1444.00, 2575.79) | 94.33 | 5257.10 (3963.18, 6980.39) | 5293.35 (3966.56, 6937.71) | 5247.27 (3967.96, 6827.77) | 5273.57 (3931.81, 6894.33) | -0.02 (-0.03, -0.01) | 0.07 (0.05, 0.08) | -0.09 (-0.11, -0.07) | 0.06 (0.03, 0.08) |
| Vanuatu | 5.91 (4.53, 7.46) | 10.64 (8.00, 13.70) | 80.22 | 4238.67 (3292.19, 5296.93) | 3891.35 (3088.84, 4792.05) | 3908.83 (3100.91, 4794.85) | 3703.32 (2814.63, 4744.41) | -0.53 (-0.61, -0.45) | -0.91 (-1.35, -0.46) | 0.06 (0.02, 0.10) | -0.63 (-0.91, -0.36) |
| Venezuela (Bolivarian Republic of) | 686.46 (507.85, 904.41) | 1072.25 (801.73, 1416.20) | 56.20 | 3691.65 (2757.79, 4817.14) | 3677.13 (2791.62, 4832.92) | 3643.15 (2735.11, 4739.82) | 3659.99 (2742.04, 4818.85) | -0.04 (-0.06, -0.03) | -0.04 (-0.05, -0.03) | -0.10 (-0.12, -0.07) | 0.10 (0.04, 0.16) |
| Viet Nam | 2407.77 (1785.65, 3175.45) | 4432.18 (3306.95, 5853.01) | 84.08 | 3804.72 (2865.61, 4975.86) | 3846.89 (2887.10, 5064.76) | 3967.86 (3032.37, 5134.72) | 3954.88 (2956.03, 5212.96) | 0.20 (0.17, 0.23) | 0.13 (0.06, 0.20) | 0.31 (0.21, 0.41) | -0.11 (-0.23, 0.00) |
| Yemen | 298.53 (220.51, 393.18) | 862.69 (628.92, 1140.60) | 188.98 | 2908.05 (2150.15, 3787.25) | 2869.83 (2148.78, 3780.91) | 2892.40 (2159.17, 3811.09) | 2868.75 (2125.57, 3741.34) | -0.01 (-0.03, 0.01) | -0.13 (-0.14, -0.11) | 0.08 (0.07, 0.10) | -0.12 (-0.18, -0.05) |
| Zambia | 158.96 (119.17, 209.51) | 421.12 (313.65, 560.68) | 164.93 | 2435.04 (1844.48, 3203.95) | 2336.10 (1865.66, 2900.27) | 2471.95 (1847.34, 3257.95) | 2451.75 (1844.99, 3219.47) | 0.09 (0.01, 0.18) | -0.44 (-0.67, -0.21) | 0.60 (0.45, 0.75) | -0.08 (-0.14, -0.01) |
| Zimbabwe | 183.67 (136.54, 243.52) | 304.15 (224.17, 402.06) | 65.60 | 2107.69 (1575.00, 2780.88) | 2118.17 (1650.10, 2686.41) | 2119.75 (1595.01, 2750.15) | 2098.19 (1562.92, 2765.41) | -0.03 (-0.05, -0.02) | 0.04 (-0.05, 0.12) | 0.01 (-0.01, 0.03) | -0.11 (-0.13, -0.10) |
| **Gonococcal infection** | | |  |  |  |  |  |  |  |  |  |
| Afghanistan | 132.63 (85.37, 211.58) | 477.83 (316.71, 742.79) | 260.27 | 1147.30 (791.27, 1684.53) | 1180.13 (820.23, 1734.97) | 1168.15 (806.57, 1720.24) | 1132.97 (790.46, 1655.02) | -0.11 (-0.16, -0.05) | 0.24 (0.04, 0.44) | -0.10 (-0.27, 0.07) | -0.35 (-0.38, -0.32) |
| Albania | 72.39 (48.21, 109.25) | 49.62 (33.87, 72.74) | -31.45 | 1947.52 (1331.43, 2887.67) | 1987.76 (1382.12, 2928.69) | 1878.88 (1285.63, 2766.01) | 1879.24 (1274.99, 2773.28) | -0.27 (-0.32, -0.21) | 0.20 (0.16, 0.24) | -0.60 (-0.64, -0.56) | -0.15 (-0.33, 0.03) |
| Algeria | 206.96 (136.36, 317.24) | 311.87 (217.27, 453.58) | 50.69 | 758.28 (528.33, 1120.57) | 741.78 (517.76, 1086.10) | 737.95 (511.02, 1080.61) | 724.58 (501.11, 1061.84) | -0.15 (-0.17, -0.13) | -0.22 (-0.24, -0.21) | -0.05 (-0.12, 0.02) | -0.30 (-0.40, -0.19) |
| American Samoa | 0.56 (0.37, 0.85) | 0.59 (0.40, 0.93) | 4.95 | 1042.99 (720.47, 1522.86) | 1040.88 (716.38, 1545.45) | 1006.29 (697.71, 1473.86) | 976.49 (674.38, 1466.43) | -0.31 (-0.36, -0.26) | -0.08 (-0.29, 0.14) | -0.33 (-0.55, -0.11) | -0.32 (-0.34, -0.31) |
| Andorra | 0.08 (0.06, 0.10) | 0.10 (0.08, 0.14) | 34.02 | 128.41 (92.27, 174.10) | 128.00 (92.92, 174.82) | 127.98 (92.77, 174.91) | 126.09 (90.19, 173.02) | -0.06 (-0.08, -0.04) | -0.07 (-0.12, -0.02) | 0.02 (-0.04, 0.09) | -0.17 (-0.25, -0.09) |
| Angola | 133.38 (90.67, 197.32) | 354.37 (239.04, 523.86) | 165.69 | 1311.35 (939.41, 1868.74) | 1281.03 (916.40, 1819.59) | 1233.37 (882.95, 1781.44) | 1196.41 (850.36, 1713.27) | -0.37 (-0.39, -0.35) | -0.26 (-0.39, -0.12) | -0.39 (-0.42, -0.36) | -0.41 (-0.53, -0.29) |
| Antigua and Barbuda | 0.82 (0.55, 1.24) | 1.10 (0.77, 1.65) | 34.43 | 1231.63 (841.84, 1832.87) | 1247.22 (850.18, 1880.65) | 1165.96 (805.24, 1728.03) | 1201.52 (820.40, 1825.48) | -0.21 (-0.27, -0.15) | 0.14 (0.02, 0.27) | -0.72 (-0.75, -0.68) | 0.31 (0.19, 0.42) |
| Argentina | 141.96 (101.09, 199.89) | 198.77 (143.41, 271.98) | 40.02 | 435.18 (311.45, 610.60) | 435.31 (312.83, 607.95) | 433.37 (308.85, 609.44) | 435.77 (312.70, 606.25) | -0.01 (-0.03, 0.00) | -0.01 (-0.06, 0.04) | -0.05 (-0.11, 0.02) | 0.01 (-0.10, 0.12) |
| Armenia | 91.07 (61.71, 134.75) | 58.74 (41.23, 85.48) | -35.50 | 2526.47 (1723.87, 3748.18) | 2558.58 (1752.75, 3883.94) | 2230.50 (1534.42, 3259.63) | 2178.65 (1485.36, 3262.29) | -0.74 (-0.84, -0.65) | 0.13 (0.09, 0.17) | -1.45 (-1.69, -1.22) | -0.37 (-0.53, -0.22) |
| Australia | 54.58 (39.28, 76.08) | 67.21 (49.05, 91.98) | 23.14 | 314.11 (223.21, 439.68) | 307.62 (219.43, 426.77) | 304.42 (217.10, 423.47) | 299.36 (212.25, 420.88) | -0.19 (-0.21, -0.16) | -0.22 (-0.26, -0.18) | -0.10 (-0.22, 0.03) | -0.26 (-0.36, -0.16) |
| Austria | 10.08 (7.36, 13.62) | 10.01 (7.54, 13.66) | -0.70 | 126.89 (91.23, 173.94) | 122.97 (89.30, 167.69) | 124.33 (90.97, 170.29) | 119.74 (86.61, 168.66) | -0.11 (-0.17, -0.05) | -0.31 (-0.37, -0.24) | 0.12 (-0.09, 0.32) | -0.51 (-0.64, -0.37) |
| Azerbaijan | 213.72 (141.38, 325.12) | 244.57 (168.77, 359.58) | 14.44 | 2567.83 (1765.34, 3864.31) | 2485.55 (1701.96, 3669.25) | 2352.76 (1605.61, 3480.39) | 2315.16 (1580.27, 3486.17) | -0.50 (-0.56, -0.45) | -0.34 (-0.60, -0.08) | -0.54 (-0.67, -0.42) | -0.24 (-0.34, -0.15) |
| Bahamas | 3.71 (2.49, 5.76) | 4.68 (3.22, 6.93) | 26.13 | 1210.42 (833.79, 1834.98) | 1243.48 (849.49, 1870.80) | 1164.34 (799.89, 1778.78) | 1173.71 (799.64, 1756.75) | -0.27 (-0.33, -0.20) | 0.27 (0.23, 0.32) | -0.69 (-0.71, -0.66) | 0.04 (-0.17, 0.25) |
| Bahrain | 6.69 (4.61, 9.78) | 15.35 (11.23, 21.21) | 129.31 | 1118.21 (783.25, 1643.46) | 1124.05 (782.68, 1652.47) | 1116.16 (772.97, 1650.14) | 1060.98 (739.72, 1535.95) | -0.16 (-0.23, -0.08) | 0.04 (0.01, 0.08) | -0.03 (-0.30, 0.24) | -0.55 (-0.57, -0.54) |
| Bangladesh | 871.43 (579.38, 1320.19) | 1155.53 (799.42, 1707.47) | 32.60 | 766.47 (536.72, 1120.72) | 746.19 (522.18, 1089.31) | 682.33 (475.81, 1003.30) | 649.97 (455.95, 952.96) | -0.68 (-0.75, -0.62) | -0.30 (-0.60, 0.01) | -0.91 (-1.00, -0.81) | -0.51 (-0.71, -0.31) |
| Barbados | 3.61 (2.47, 5.45) | 3.39 (2.41, 4.90) | -6.19 | 1313.85 (905.00, 1967.73) | 1393.64 (1018.11, 1887.25) | 1351.99 (990.89, 1839.91) | 1272.38 (874.96, 1890.04) | -0.16 (-0.29, -0.03) | 0.61 (0.43, 0.80) | -0.30 (-0.39, -0.22) | -0.77 (-1.02, -0.53) |
| Belarus | 197.47 (137.19, 290.13) | 134.74 (96.48, 190.61) | -31.77 | 1982.60 (1367.63, 2940.14) | 1964.17 (1352.68, 2918.48) | 1826.58 (1256.76, 2721.00) | 1809.41 (1219.80, 2693.67) | -0.55 (-0.61, -0.49) | -0.14 (-0.42, 0.15) | -0.74 (-0.76, -0.72) | -0.29 (-0.53, -0.04) |
| Belgium | 12.91 (9.49, 17.43) | 12.26 (9.18, 16.42) | -5.02 | 130.18 (93.46, 179.21) | 126.17 (91.30, 170.79) | 122.53 (88.35, 168.79) | 117.56 (85.30, 161.93) | -0.37 (-0.39, -0.34) | -0.33 (-0.42, -0.23) | -0.29 (-0.38, -0.19) | -0.51 (-0.58, -0.44) |
| Belize | 2.32 (1.52, 3.64) | 5.48 (3.66, 8.31) | 135.65 | 1162.43 (799.74, 1739.18) | 1216.12 (834.15, 1834.75) | 1132.15 (779.95, 1671.59) | 1143.34 (782.73, 1693.50) | -0.21 (-0.28, -0.13) | 0.46 (0.38, 0.54) | -0.76 (-0.80, -0.72) | 0.06 (-0.03, 0.15) |
| Benin | 61.66 (42.53, 90.92) | 179.02 (122.88, 268.78) | 190.34 | 1423.18 (1029.28, 2016.56) | 1480.29 (1175.26, 1840.63) | 1332.83 (1014.75, 1810.34) | 1428.01 (1029.60, 2033.85) | -0.18 (-0.33, -0.02) | 0.38 (0.27, 0.50) | -1.10 (-1.49, -0.71) | 0.77 (0.29, 1.26) |
| Bermuda | 0.73 (0.52, 1.06) | 0.54 (0.40, 0.76) | -25.49 | 1173.46 (804.53, 1766.26) | 1183.09 (810.12, 1797.05) | 1099.42 (756.07, 1644.32) | 1111.41 (762.19, 1676.19) | -0.32 (-0.38, -0.26) | 0.11 (0.01, 0.20) | -0.77 (-0.82, -0.71) | 0.09 (-0.08, 0.26) |
| Bhutan | 4.84 (3.17, 7.50) | 5.67 (3.90, 8.44) | 17.27 | 689.75 (477.75, 1026.70) | 678.50 (477.44, 986.99) | 660.56 (453.72, 961.91) | 629.69 (441.05, 927.03) | -0.31 (-0.34, -0.28) | -0.17 (-0.32, -0.01) | -0.27 (-0.30, -0.25) | -0.52 (-0.67, -0.37) |
| Bolivia (Plurinational State of) | 17.41 (11.91, 25.78) | 32.99 (22.72, 48.19) | 89.48 | 270.95 (192.60, 388.50) | 272.94 (193.54, 392.77) | 267.79 (188.38, 381.96) | 264.18 (185.89, 381.12) | -0.11 (-0.13, -0.09) | 0.08 (0.06, 0.11) | -0.20 (-0.25, -0.16) | -0.19 (-0.24, -0.15) |
| Bosnia and Herzegovina | 93.31 (63.94, 137.02) | 51.63 (36.36, 74.05) | -44.66 | 1868.29 (1279.34, 2741.20) | 1891.95 (1295.74, 2797.22) | 1845.29 (1283.92, 2738.60) | 1842.15 (1247.95, 2736.40) | -0.16 (-0.19, -0.13) | 0.11 (0.01, 0.21) | -0.25 (-0.32, -0.19) | -0.13 (-0.30, 0.03) |
| Botswana | 34.38 (22.49, 53.63) | 60.99 (41.93, 89.59) | 77.40 | 2432.61 (1686.76, 3651.71) | 2338.24 (1739.06, 3195.77) | 2528.76 (1865.92, 3475.99) | 2324.78 (1614.58, 3397.80) | -0.07 (-0.19, 0.06) | -0.43 (-0.59, -0.26) | 0.84 (0.67, 1.02) | -1.18 (-1.49, -0.86) |
| Brazil | 1541.66 (1037.79, 2248.01) | 2047.54 (1405.15, 2920.87) | 32.81 | 934.86 (641.51, 1339.39) | 1031.54 (702.08, 1488.49) | 856.13 (591.14, 1218.81) | 915.67 (623.61, 1311.05) | -0.29 (-0.49, -0.09) | 1.05 (0.57, 1.54) | -2.01 (-2.23, -1.79) | 0.76 (0.50, 1.02) |
| Brunei Darussalam | 1.99 (1.39, 2.82) | 3.45 (2.52, 4.78) | 73.20 | 689.23 (498.46, 956.12) | 683.58 (490.41, 964.18) | 682.99 (492.15, 957.16) | 684.98 (496.17, 961.62) | -0.03 (-0.05, 0.00) | -0.12 (-0.25, 0.01) | 0.00 (-0.07, 0.06) | 0.03 (-0.14, 0.19) |
| Bulgaria | 144.78 (102.23, 211.54) | 90.31 (64.90, 127.84) | -37.62 | 1832.17 (1265.10, 2728.24) | 1898.65 (1308.34, 2799.32) | 1835.28 (1251.03, 2679.37) | 1785.88 (1223.46, 2666.60) | -0.17 (-0.23, -0.11) | 0.36 (0.33, 0.39) | -0.35 (-0.45, -0.26) | -0.44 (-0.60, -0.28) |
| Burkina Faso | 136.86 (92.65, 203.12) | 328.91 (220.32, 504.50) | 140.33 | 1574.01 (1128.09, 2249.58) | 1600.84 (1142.80, 2314.86) | 1461.06 (1025.30, 2119.09) | 1456.82 (1025.54, 2126.55) | -0.26 (-0.34, -0.18) | 0.23 (-0.01, 0.47) | -0.98 (-1.05, -0.92) | 0.16 (-0.13, 0.46) |
| Burundi | 71.66 (47.85, 107.82) | 155.10 (104.01, 233.42) | 116.43 | 1313.10 (916.46, 1913.97) | 1306.92 (923.77, 1866.61) | 1282.42 (902.39, 1872.22) | 1263.11 (885.21, 1845.27) | -0.16 (-0.19, -0.13) | -0.06 (-0.12, 0.01) | -0.20 (-0.29, -0.11) | -0.28 (-0.44, -0.11) |
| Cabo Verde | 4.01 (2.67, 6.21) | 7.21 (5.12, 10.50) | 79.69 | 1135.93 (807.71, 1641.23) | 1160.78 (820.55, 1674.92) | 1158.36 (818.78, 1661.41) | 1153.30 (827.44, 1653.19) | 0.04 (0.00, 0.07) | 0.19 (0.10, 0.28) | -0.03 (-0.14, 0.09) | -0.14 (-0.38, 0.10) |
| Cambodia | 131.05 (89.11, 194.58) | 228.69 (159.51, 329.09) | 74.50 | 1285.46 (919.00, 1839.57) | 1340.88 (955.03, 1893.86) | 1265.70 (912.60, 1784.09) | 1260.45 (893.12, 1800.84) | -0.30 (-0.38, -0.22) | 0.31 (-0.12, 0.75) | -0.60 (-0.79, -0.40) | -0.08 (-0.17, 0.00) |
| Cameroon | 201.75 (139.53, 298.06) | 601.97 (416.29, 881.96) | 198.38 | 2026.54 (1470.39, 2844.49) | 2087.98 (1588.58, 2811.01) | 1967.42 (1411.19, 2749.10) | 1945.51 (1404.64, 2691.25) | -0.29 (-0.36, -0.23) | 0.24 (-0.01, 0.49) | -0.62 (-0.74, -0.50) | -0.20 (-0.39, 0.00) |
| Canada | 79.26 (57.06, 109.72) | 88.28 (65.23, 120.06) | 11.37 | 284.73 (202.03, 401.10) | 279.04 (196.58, 393.27) | 276.79 (195.42, 384.82) | 275.74 (194.81, 388.30) | -0.11 (-0.13, -0.10) | -0.21 (-0.27, -0.15) | -0.08 (-0.14, -0.02) | -0.04 (-0.10, 0.01) |
| Central African Republic | 38.26 (25.88, 56.90) | 75.84 (51.55, 113.59) | 98.24 | 1380.56 (976.53, 1986.37) | 1403.08 (1014.76, 1985.19) | 1401.55 (997.73, 1985.09) | 1355.30 (963.93, 1948.73) | -0.10 (-0.15, -0.06) | 0.13 (-0.01, 0.28) | -0.02 (-0.02, -0.01) | -0.44 (-0.51, -0.37) |
| Chad | 74.46 (50.49, 112.25) | 193.71 (131.25, 294.25) | 160.17 | 1342.58 (958.30, 1931.44) | 1344.26 (964.76, 1958.28) | 1286.50 (915.11, 1846.25) | 1256.24 (900.02, 1788.77) | -0.27 (-0.30, -0.23) | 0.01 (-0.03, 0.05) | -0.46 (-0.55, -0.38) | -0.34 (-0.55, -0.14) |
| Chile | 63.67 (44.72, 91.15) | 76.29 (55.20, 105.44) | 19.82 | 442.03 (317.21, 616.79) | 438.62 (314.07, 614.74) | 426.24 (309.31, 596.27) | 419.63 (296.53, 594.86) | -0.21 (-0.24, -0.18) | -0.09 (-0.14, -0.03) | -0.29 (-0.44, -0.14) | -0.23 (-0.33, -0.13) |
| China | 17761.06 (12085.71, 25792.47) | 14506.77 (10295.92, 19854.63) | -18.32 | 1243.22 (864.55, 1763.25) | 1168.20 (813.22, 1648.11) | 1082.69 (757.13, 1532.38) | 1096.25 (756.98, 1530.59) | -0.55 (-0.60, -0.50) | -0.64 (-0.69, -0.60) | -0.75 (-0.91, -0.60) | 0.01 (-0.17, 0.19) |
| Colombia | 183.80 (123.65, 279.63) | 253.98 (175.68, 374.55) | 38.18 | 502.22 (351.30, 741.98) | 514.43 (358.36, 761.54) | 504.99 (351.80, 747.37) | 504.40 (348.08, 741.47) | -0.03 (-0.07, 0.00) | 0.24 (0.21, 0.28) | -0.19 (-0.25, -0.14) | -0.07 (-0.14, 0.00) |
| Comoros | 6.15 (4.02, 9.34) | 9.71 (6.62, 14.50) | 57.96 | 1292.00 (897.47, 1877.61) | 1274.85 (907.48, 1846.98) | 1264.02 (895.22, 1833.92) | 1235.38 (863.09, 1802.91) | -0.16 (-0.19, -0.13) | -0.15 (-0.26, -0.05) | -0.09 (-0.16, -0.01) | -0.39 (-0.61, -0.16) |
| Congo | 31.11 (20.89, 46.88) | 64.78 (45.44, 93.71) | 108.21 | 1247.53 (883.53, 1791.93) | 1265.96 (903.67, 1802.48) | 1227.29 (862.07, 1752.73) | 1190.32 (847.02, 1707.03) | -0.23 (-0.26, -0.19) | 0.12 (-0.04, 0.28) | -0.32 (-0.42, -0.23) | -0.38 (-0.48, -0.27) |
| Cook Islands | 0.20 (0.13, 0.31) | 0.16 (0.11, 0.23) | -22.10 | 979.46 (674.38, 1463.01) | 973.48 (662.59, 1449.80) | 957.93 (660.84, 1416.76) | 951.00 (650.70, 1409.29) | -0.14 (-0.16, -0.13) | -0.08 (-0.17, 0.02) | -0.16 (-0.23, -0.10) | -0.04 (-0.11, 0.02) |
| Costa Rica | 24.96 (16.87, 37.56) | 36.26 (25.49, 52.99) | 45.32 | 751.41 (522.01, 1101.22) | 772.08 (538.85, 1138.17) | 749.85 (526.89, 1101.64) | 734.65 (511.83, 1085.89) | -0.12 (-0.17, -0.07) | 0.27 (0.25, 0.29) | -0.31 (-0.32, -0.29) | -0.28 (-0.36, -0.20) |
| Croatia | 84.76 (59.91, 122.73) | 59.48 (42.03, 85.82) | -29.83 | 1818.09 (1246.72, 2696.10) | 1801.78 (1233.34, 2655.85) | 1788.56 (1231.72, 2645.72) | 1759.02 (1184.71, 2647.64) | -0.17 (-0.20, -0.13) | -0.09 (-0.12, -0.06) | -0.07 (-0.22, 0.09) | -0.37 (-0.59, -0.15) |
| Cuba | 145.97 (97.67, 220.93) | 110.66 (80.24, 158.01) | -24.19 | 1119.68 (768.10, 1638.13) | 1197.79 (824.92, 1814.07) | 1110.90 (761.62, 1659.66) | 1107.05 (771.37, 1646.77) | -0.20 (-0.30, -0.10) | 0.70 (0.54, 0.86) | -0.80 (-0.90, -0.70) | -0.10 (-0.29, 0.09) |
| Cyprus | 1.02 (0.75, 1.42) | 1.63 (1.20, 2.17) | 59.63 | 128.46 (94.38, 178.06) | 130.88 (95.24, 178.52) | 128.40 (92.94, 175.77) | 125.37 (89.65, 173.58) | -0.12 (-0.16, -0.08) | 0.16 (0.08, 0.25) | -0.20 (-0.25, -0.15) | -0.33 (-0.45, -0.22) |
| Czechia | 181.50 (126.62, 264.96) | 139.20 (100.95, 195.60) | -23.31 | 1874.18 (1287.56, 2750.68) | 1804.23 (1232.59, 2653.33) | 1799.03 (1234.83, 2666.08) | 1773.44 (1216.91, 2633.16) | -0.20 (-0.23, -0.17) | -0.38 (-0.40, -0.35) | -0.02 (-0.09, 0.05) | -0.30 (-0.48, -0.12) |
| Côte d'Ivoire | 145.49 (99.58, 217.89) | 309.38 (214.49, 456.05) | 112.65 | 1201.58 (865.50, 1718.48) | 1227.34 (888.87, 1724.37) | 1155.64 (824.47, 1633.76) | 1141.54 (818.18, 1647.56) | -0.31 (-0.38, -0.24) | 0.16 (-0.01, 0.34) | -0.62 (-0.86, -0.38) | -0.24 (-0.44, -0.05) |
| Democratic People's Republic of Korea | 202.86 (139.19, 301.59) | 242.86 (171.59, 346.29) | 19.72 | 906.65 (629.56, 1340.34) | 934.96 (652.59, 1385.88) | 930.03 (641.73, 1362.04) | 909.10 (628.99, 1329.18) | -0.01 (-0.05, 0.04) | 0.29 (0.24, 0.34) | -0.06 (-0.12, 0.01) | -0.26 (-0.28, -0.23) |
| Democratic Republic of the Congo | 451.84 (302.96, 674.40) | 1070.63 (725.61, 1618.84) | 136.95 | 1207.12 (853.19, 1736.07) | 1215.13 (872.32, 1720.53) | 1216.64 (872.07, 1724.73) | 1174.59 (833.77, 1713.55) | -0.09 (-0.13, -0.06) | 0.05 (-0.05, 0.15) | 0.01 (-0.04, 0.07) | -0.46 (-0.58, -0.35) |
| Denmark | 6.55 (4.90, 8.84) | 6.49 (4.84, 8.72) | -0.85 | 125.54 (91.92, 174.10) | 128.50 (92.64, 176.25) | 121.85 (89.11, 167.69) | 120.10 (86.31, 165.75) | -0.27 (-0.32, -0.22) | 0.21 (0.08, 0.35) | -0.55 (-0.66, -0.44) | -0.19 (-0.26, -0.11) |
| Djibouti | 6.92 (4.57, 10.61) | 15.57 (10.88, 22.71) | 124.95 | 1287.34 (903.07, 1870.62) | 1288.06 (916.42, 1863.39) | 1258.07 (888.52, 1825.61) | 1226.13 (862.84, 1780.48) | -0.22 (-0.26, -0.18) | -0.01 (-0.10, 0.09) | -0.24 (-0.32, -0.17) | -0.43 (-0.63, -0.23) |
| Dominica | 1.00 (0.66, 1.59) | 0.85 (0.59, 1.27) | -15.03 | 1261.03 (860.10, 1899.17) | 1297.42 (893.93, 1955.04) | 1211.87 (833.51, 1798.80) | 1235.60 (851.25, 1847.79) | -0.21 (-0.28, -0.14) | 0.31 (0.21, 0.41) | -0.72 (-0.76, -0.69) | 0.22 (0.07, 0.36) |
| Dominican Republic | 106.72 (70.33, 164.92) | 146.44 (98.92, 222.10) | 37.22 | 1287.36 (890.42, 1908.43) | 1294.23 (885.04, 1951.04) | 1224.37 (842.34, 1849.87) | 1242.13 (848.02, 1864.84) | -0.23 (-0.29, -0.18) | 0.08 (-0.02, 0.18) | -0.59 (-0.62, -0.56) | 0.12 (-0.08, 0.32) |
| Ecuador | 27.02 (18.26, 40.42) | 46.48 (32.36, 67.02) | 72.05 | 253.28 (178.32, 362.89) | 259.97 (184.36, 370.84) | 251.16 (176.26, 354.62) | 246.71 (175.01, 353.34) | -0.13 (-0.18, -0.08) | 0.28 (0.23, 0.32) | -0.37 (-0.39, -0.34) | -0.20 (-0.22, -0.18) |
| Egypt | 788.35 (528.73, 1201.49) | 1336.02 (906.94, 1964.04) | 69.47 | 1335.17 (925.57, 1965.37) | 1427.70 (1081.25, 1935.52) | 1279.74 (880.63, 1889.11) | 1249.11 (857.64, 1813.37) | -0.54 (-0.69, -0.40) | 0.53 (0.03, 1.03) | -1.11 (-1.58, -0.63) | -0.28 (-0.34, -0.22) |
| El Salvador | 43.48 (28.70, 68.21) | 50.30 (34.31, 75.45) | 15.69 | 762.14 (526.92, 1138.85) | 771.39 (538.91, 1132.73) | 727.51 (502.75, 1070.49) | 725.94 (504.45, 1064.44) | -0.24 (-0.28, -0.19) | 0.12 (0.11, 0.13) | -0.62 (-0.68, -0.57) | -0.02 (-0.13, 0.09) |
| Equatorial Guinea | 4.95 (3.37, 7.30) | 20.73 (13.70, 31.55) | 318.93 | 1245.74 (884.97, 1766.54) | 1203.01 (851.18, 1710.26) | 1199.99 (843.77, 1750.57) | 1222.12 (856.58, 1752.56) | -0.12 (-0.18, -0.06) | -0.38 (-0.57, -0.18) | -0.03 (-0.09, 0.04) | 0.11 (-0.03, 0.25) |
| Eritrea | 41.93 (27.90, 64.04) | 95.97 (64.83, 145.98) | 128.87 | 1344.76 (942.88, 1983.27) | 1313.74 (934.26, 1894.31) | 1298.57 (912.08, 1896.53) | 1257.36 (887.91, 1826.60) | -0.22 (-0.25, -0.19) | -0.24 (-0.31, -0.18) | -0.12 (-0.16, -0.07) | -0.49 (-0.71, -0.27) |
| Estonia | 29.97 (21.12, 43.60) | 17.84 (12.75, 25.19) | -40.47 | 2055.49 (1419.29, 3063.39) | 1820.84 (1257.65, 2681.82) | 1789.83 (1216.30, 2644.47) | 1773.76 (1207.34, 2641.55) | -0.50 (-0.59, -0.42) | -1.22 (-1.26, -1.17) | -0.16 (-0.22, -0.09) | -0.33 (-0.62, -0.04) |
| Eswatini | 22.66 (15.17, 34.84) | 34.02 (22.69, 50.83) | 50.13 | 2719.99 (1914.04, 3996.35) | 2731.08 (1919.40, 4032.32) | 2670.60 (1858.55, 3909.66) | 2588.02 (1797.77, 3791.22) | -0.20 (-0.25, -0.15) | 0.02 (-0.07, 0.11) | -0.23 (-0.35, -0.11) | -0.49 (-0.72, -0.26) |
| Ethiopia | 511.33 (343.69, 750.92) | 1098.38 (736.68, 1615.74) | 114.81 | 1041.23 (724.94, 1478.10) | 1014.94 (705.67, 1433.66) | 998.46 (693.87, 1418.21) | 949.60 (657.23, 1342.53) | -0.28 (-0.32, -0.24) | -0.26 (-0.29, -0.22) | -0.17 (-0.26, -0.08) | -0.67 (-0.85, -0.49) |
| Fiji | 10.67 (7.47, 15.91) | 10.70 (7.36, 15.76) | 0.33 | 1249.18 (895.76, 1823.25) | 1198.10 (835.71, 1716.51) | 1171.45 (816.95, 1744.60) | 1140.24 (782.77, 1677.45) | -0.28 (-0.31, -0.25) | -0.41 (-0.45, -0.36) | -0.22 (-0.37, -0.07) | -0.27 (-0.32, -0.22) |
| Finland | 6.39 (4.79, 8.72) | 5.71 (4.30, 7.58) | -10.63 | 129.24 (94.03, 176.51) | 125.16 (91.76, 172.23) | 122.17 (89.66, 167.28) | 117.25 (84.61, 161.08) | -0.32 (-0.34, -0.30) | -0.31 (-0.33, -0.29) | -0.24 (-0.25, -0.24) | -0.55 (-0.66, -0.45) |
| France | 73.82 (55.25, 100.82) | 72.09 (53.31, 97.24) | -2.33 | 128.11 (94.41, 175.48) | 127.32 (92.40, 175.22) | 124.99 (91.04, 171.55) | 120.76 (86.50, 164.69) | -0.20 (-0.24, -0.17) | -0.06 (-0.08, -0.05) | -0.19 (-0.27, -0.10) | -0.43 (-0.48, -0.37) |
| Gabon | 12.45 (8.54, 18.37) | 22.39 (15.46, 32.77) | 79.80 | 1258.43 (895.43, 1794.01) | 1253.94 (896.84, 1792.77) | 1210.22 (861.26, 1703.13) | 1154.56 (810.05, 1649.05) | -0.32 (-0.36, -0.29) | -0.05 (-0.10, 0.01) | -0.37 (-0.40, -0.33) | -0.59 (-0.68, -0.50) |
| Gambia | 12.42 (8.40, 18.66) | 29.55 (19.87, 44.43) | 137.84 | 1271.20 (915.59, 1821.29) | 1267.90 (913.02, 1825.47) | 1219.93 (876.63, 1775.76) | 1212.30 (858.10, 1741.01) | -0.21 (-0.24, -0.18) | -0.04 (-0.12, 0.04) | -0.41 (-0.47, -0.34) | -0.15 (-0.39, 0.10) |
| Georgia | 128.43 (88.77, 189.75) | 66.67 (46.61, 97.77) | -48.09 | 2343.84 (1611.63, 3500.40) | 2181.72 (1485.72, 3235.10) | 2226.63 (1536.33, 3301.85) | 2217.48 (1502.86, 3319.71) | -0.21 (-0.28, -0.14) | -0.77 (-1.02, -0.51) | 0.23 (0.16, 0.29) | -0.12 (-0.23, -0.01) |
| Germany | 105.43 (77.82, 142.01) | 94.25 (70.62, 126.57) | -10.61 | 130.64 (95.04, 178.16) | 128.71 (94.12, 175.64) | 126.87 (91.33, 175.40) | 123.38 (88.99, 169.51) | -0.23 (-0.24, -0.21) | -0.17 (-0.29, -0.05) | -0.14 (-0.17, -0.11) | -0.33 (-0.36, -0.30) |
| Ghana | 301.51 (203.97, 453.43) | 660.03 (454.09, 963.92) | 118.91 | 1972.70 (1393.89, 2835.84) | 1987.82 (1436.01, 2797.20) | 2028.88 (1432.99, 2931.74) | 1864.03 (1331.33, 2664.88) | 0.02 (-0.11, 0.14) | 0.21 (-0.31, 0.73) | 0.21 (0.05, 0.38) | -1.07 (-1.24, -0.91) |
| Greece | 12.87 (9.57, 17.21) | 10.68 (7.94, 14.44) | -16.99 | 122.60 (89.26, 166.96) | 121.17 (87.67, 166.32) | 123.09 (89.75, 167.41) | 116.87 (84.65, 162.18) | -0.12 (-0.17, -0.07) | -0.12 (-0.20, -0.05) | 0.18 (0.10, 0.26) | -0.66 (-0.76, -0.57) |
| Greenland | 0.20 (0.14, 0.29) | 0.16 (0.12, 0.22) | -19.02 | 313.53 (222.30, 444.56) | 316.69 (224.32, 441.95) | 300.18 (214.09, 418.18) | 294.41 (209.68, 410.85) | -0.30 (-0.35, -0.25) | 0.08 (0.00, 0.16) | -0.57 (-0.66, -0.49) | -0.23 (-0.30, -0.17) |
| Grenada | 1.13 (0.75, 1.71) | 1.41 (0.97, 2.10) | 23.96 | 1280.27 (876.75, 1904.42) | 1294.89 (889.02, 1963.79) | 1222.36 (842.73, 1821.33) | 1253.19 (867.21, 1868.19) | -0.20 (-0.25, -0.14) | 0.14 (0.03, 0.24) | -0.61 (-0.64, -0.58) | 0.22 (0.06, 0.38) |
| Guam | 1.57 (1.06, 2.33) | 1.56 (1.08, 2.32) | -0.75 | 973.88 (683.08, 1427.32) | 997.74 (685.45, 1488.45) | 954.69 (661.83, 1400.04) | 929.03 (642.71, 1377.00) | -0.28 (-0.34, -0.21) | 0.20 (-0.01, 0.42) | -0.45 (-0.63, -0.27) | -0.27 (-0.30, -0.24) |
| Guatemala | 89.18 (60.70, 132.99) | 208.79 (139.56, 311.72) | 134.11 | 1130.04 (801.57, 1640.90) | 1128.74 (790.49, 1656.09) | 1032.07 (730.86, 1507.89) | 1007.11 (694.44, 1458.30) | -0.48 (-0.54, -0.43) | 0.01 (-0.02, 0.04) | -0.95 (-0.99, -0.90) | -0.29 (-0.38, -0.20) |
| Guinea | 74.03 (50.81, 109.15) | 154.70 (104.42, 231.51) | 108.97 | 1306.31 (931.28, 1876.14) | 1299.49 (933.68, 1860.76) | 1256.98 (903.08, 1813.23) | 1227.56 (873.87, 1749.06) | -0.21 (-0.25, -0.18) | -0.05 (-0.10, -0.01) | -0.35 (-0.47, -0.24) | -0.32 (-0.60, -0.05) |
| Guinea-Bissau | 13.15 (8.86, 19.83) | 25.54 (17.41, 38.59) | 94.29 | 1333.99 (949.08, 1918.87) | 1319.09 (936.69, 1904.65) | 1274.04 (910.54, 1810.11) | 1244.26 (887.01, 1794.26) | -0.25 (-0.28, -0.22) | -0.13 (-0.20, -0.05) | -0.36 (-0.51, -0.22) | -0.32 (-0.54, -0.09) |
| Guyana | 11.77 (7.73, 18.48) | 11.12 (7.51, 16.73) | -5.52 | 1300.15 (896.75, 1958.97) | 1343.87 (937.22, 2026.38) | 1255.61 (865.40, 1873.45) | 1251.94 (860.52, 1851.71) | -0.25 (-0.34, -0.17) | 0.36 (0.20, 0.51) | -0.71 (-0.75, -0.68) | -0.09 (-0.30, 0.12) |
| Haiti | 83.98 (55.95, 129.22) | 170.02 (114.30, 260.80) | 102.44 | 1291.98 (894.36, 1921.09) | 1339.51 (911.62, 2009.36) | 1214.92 (847.78, 1810.12) | 1240.37 (852.63, 1869.28) | -0.36 (-0.47, -0.25) | 0.40 (0.21, 0.59) | -1.01 (-1.07, -0.96) | 0.16 (-0.22, 0.54) |
| Honduras | 38.07 (25.34, 59.02) | 88.91 (59.95, 135.75) | 133.52 | 813.36 (570.27, 1210.09) | 837.72 (589.96, 1232.25) | 795.37 (554.70, 1170.74) | 788.98 (553.30, 1154.47) | -0.17 (-0.23, -0.11) | 0.30 (0.27, 0.33) | -0.55 (-0.67, -0.43) | -0.11 (-0.23, 0.01) |
| Hungary | 183.31 (128.89, 266.10) | 137.46 (99.38, 191.29) | -25.01 | 1932.99 (1322.49, 2854.48) | 1828.03 (1269.05, 2699.27) | 1809.32 (1237.06, 2667.69) | 1808.41 (1256.98, 2645.03) | -0.24 (-0.27, -0.20) | -0.56 (-0.59, -0.54) | -0.10 (-0.12, -0.08) | -0.12 (-0.26, 0.02) |
| Iceland | 0.34 (0.24, 0.46) | 0.40 (0.29, 0.54) | 18.56 | 129.74 (94.30, 176.68) | 127.66 (93.51, 171.53) | 125.47 (90.91, 171.59) | 119.56 (87.16, 166.19) | -0.28 (-0.32, -0.25) | -0.17 (-0.22, -0.12) | -0.14 (-0.17, -0.10) | -0.62 (-0.71, -0.52) |
| India | 12619.37 (8563.63, 18404.48) | 20523.84 (14120.23, 29753.69) | 62.64 | 1410.54 (969.15, 2010.30) | 1339.04 (924.36, 1913.45) | 1515.46 (1016.61, 2182.01) | 1317.80 (911.47, 1883.00) | 0.16 (-0.06, 0.38) | -0.55 (-0.73, -0.38) | 1.29 (0.80, 1.79) | -1.83 (-2.73, -0.93) |
| Indonesia | 2922.27 (2047.14, 4139.90) | 4048.74 (2897.20, 5569.40) | 38.55 | 1463.64 (1045.30, 2025.17) | 1476.79 (1048.16, 2066.06) | 1446.84 (1041.29, 2000.73) | 1440.55 (1023.78, 1993.96) | -0.09 (-0.13, -0.04) | 0.06 (-0.08, 0.20) | -0.21 (-0.47, 0.05) | 0.02 (-0.12, 0.16) |
| Iran (Islamic Republic of) | 722.47 (474.70, 1073.96) | 1014.06 (709.12, 1400.63) | 40.36 | 1197.89 (823.32, 1709.36) | 1099.07 (761.92, 1549.19) | 1152.37 (792.23, 1628.27) | 1174.51 (804.89, 1655.61) | 0.02 (-0.09, 0.12) | -0.88 (-1.10, -0.67) | 0.50 (0.37, 0.63) | 0.19 (0.03, 0.36) |
| Iraq | 196.09 (129.81, 303.91) | 506.36 (338.80, 764.54) | 158.22 | 1099.07 (761.21, 1635.91) | 1102.75 (764.41, 1617.20) | 1059.39 (731.38, 1548.29) | 1017.77 (703.74, 1500.57) | -0.34 (-0.38, -0.30) | -0.01 (-0.19, 0.17) | -0.42 (-0.48, -0.35) | -0.49 (-0.55, -0.42) |
| Ireland | 4.56 (3.28, 6.30) | 5.49 (4.07, 7.30) | 20.38 | 126.12 (91.87, 171.66) | 125.33 (91.21, 172.10) | 121.63 (88.18, 166.96) | 117.84 (84.56, 160.24) | -0.25 (-0.30, -0.19) | -0.06 (-0.07, -0.05) | -0.30 (-0.50, -0.10) | -0.40 (-0.46, -0.33) |
| Israel | 6.29 (4.46, 8.94) | 10.74 (7.78, 14.84) | 70.69 | 128.81 (92.73, 178.69) | 124.24 (89.98, 171.16) | 123.88 (89.88, 169.04) | 121.97 (87.92, 168.87) | -0.23 (-0.27, -0.19) | -0.41 (-0.66, -0.15) | -0.02 (-0.16, 0.13) | -0.21 (-0.26, -0.15) |
| Italy | 83.14 (61.57, 110.76) | 73.95 (55.52, 100.33) | -11.06 | 140.82 (103.56, 187.25) | 141.05 (103.68, 188.40) | 140.49 (103.72, 187.92) | 138.33 (102.33, 185.10) | -0.07 (-0.10, -0.05) | 0.00 (-0.08, 0.08) | -0.04 (-0.13, 0.06) | -0.17 (-0.20, -0.14) |
| Jamaica | 31.70 (20.77, 49.36) | 37.66 (25.76, 57.36) | 18.80 | 1195.05 (821.87, 1787.28) | 1224.77 (842.66, 1840.36) | 1196.66 (825.49, 1803.00) | 1198.01 (822.44, 1802.27) | -0.10 (-0.14, -0.05) | 0.23 (0.21, 0.26) | -0.24 (-0.27, -0.21) | -0.05 (-0.24, 0.14) |
| Japan | 892.99 (658.02, 1201.59) | 709.17 (526.17, 956.82) | -20.59 | 700.83 (504.83, 945.26) | 710.48 (514.61, 961.21) | 714.01 (513.10, 965.94) | 708.74 (510.00, 957.20) | 0.04 (0.00, 0.07) | 0.12 (0.07, 0.17) | 0.05 (-0.12, 0.21) | -0.14 (-0.24, -0.03) |
| Jordan | 42.97 (28.00, 67.64) | 122.70 (83.57, 180.08) | 185.56 | 994.51 (683.31, 1490.77) | 961.94 (664.18, 1443.08) | 946.62 (651.55, 1411.74) | 927.55 (642.03, 1349.08) | -0.23 (-0.25, -0.21) | -0.35 (-0.44, -0.26) | -0.13 (-0.24, -0.03) | -0.20 (-0.26, -0.14) |
| Kazakhstan | 415.02 (281.98, 612.58) | 385.95 (267.87, 561.84) | -7.01 | 2402.85 (1646.10, 3536.50) | 2449.36 (1678.67, 3654.39) | 2246.18 (1539.94, 3330.70) | 2223.18 (1515.25, 3315.43) | -0.41 (-0.48, -0.34) | 0.21 (0.16, 0.26) | -0.92 (-1.10, -0.75) | -0.21 (-0.34, -0.09) |
| Kenya | 298.64 (200.10, 438.13) | 708.62 (475.72, 1023.24) | 137.28 | 1283.01 (889.98, 1822.51) | 1094.57 (778.78, 1523.43) | 1374.99 (955.23, 1982.80) | 1253.00 (864.91, 1762.72) | 0.52 (0.24, 0.80) | -1.53 (-1.85, -1.21) | 2.44 (1.77, 3.11) | -1.27 (-1.54, -0.99) |
| Kiribati | 0.63 (0.43, 0.96) | 1.01 (0.68, 1.53) | 59.65 | 784.53 (546.08, 1172.17) | 841.03 (575.45, 1234.73) | 810.09 (556.09, 1205.75) | 790.22 (542.96, 1173.09) | -0.19 (-0.30, -0.09) | 0.59 (0.21, 0.98) | -0.36 (-0.58, -0.15) | -0.32 (-0.41, -0.22) |
| Kuwait | 25.66 (17.61, 38.39) | 51.22 (36.99, 71.25) | 99.61 | 1231.17 (855.80, 1812.50) | 1338.52 (995.85, 1859.31) | 1299.21 (1016.79, 1708.85) | 1092.03 (757.46, 1602.30) | -0.24 (-0.49, 0.00) | 0.89 (0.56, 1.22) | -0.34 (-0.41, -0.27) | -2.25 (-2.84, -1.66) |
| Kyrgyzstan | 111.14 (73.44, 167.23) | 157.62 (107.24, 236.65) | 41.82 | 2360.66 (1604.89, 3483.81) | 2517.96 (1714.21, 3708.14) | 2353.40 (1601.58, 3449.22) | 2269.04 (1553.36, 3402.59) | -0.30 (-0.41, -0.20) | 0.65 (0.61, 0.69) | -0.71 (-0.76, -0.66) | -0.53 (-0.71, -0.35) |
| Lao People's Democratic Republic | 54.69 (37.70, 81.68) | 105.18 (72.99, 154.25) | 92.32 | 1350.52 (968.04, 1944.54) | 1402.71 (1015.35, 1989.45) | 1353.88 (969.16, 1911.54) | 1307.15 (928.73, 1881.95) | -0.23 (-0.31, -0.15) | 0.31 (0.01, 0.61) | -0.35 (-0.59, -0.11) | -0.42 (-0.52, -0.32) |
| Latvia | 50.23 (35.13, 74.03) | 26.01 (18.62, 36.78) | -48.22 | 2021.99 (1390.33, 3015.35) | 1937.03 (1326.96, 2856.45) | 1841.80 (1258.59, 2709.79) | 1837.53 (1256.21, 2736.13) | -0.62 (-0.71, -0.53) | -0.56 (-1.21, 0.10) | -0.50 (-0.68, -0.31) | -0.22 (-0.47, 0.03) |
| Lebanon | 33.79 (22.62, 51.17) | 49.45 (35.14, 71.07) | 46.37 | 1021.77 (697.74, 1532.40) | 1010.08 (703.92, 1499.35) | 989.67 (680.57, 1457.14) | 981.93 (686.71, 1428.52) | -0.17 (-0.20, -0.14) | -0.14 (-0.30, 0.02) | -0.20 (-0.24, -0.17) | -0.01 (-0.13, 0.10) |
| Lesotho | 52.37 (35.13, 79.75) | 65.68 (44.38, 98.02) | 25.42 | 2780.22 (1949.04, 4107.90) | 2799.19 (1969.04, 4043.23) | 2773.62 (1956.20, 4079.45) | 2683.39 (1866.47, 3913.71) | -0.13 (-0.18, -0.09) | 0.05 (-0.05, 0.14) | -0.09 (-0.20, 0.02) | -0.50 (-0.73, -0.28) |
| Liberia | 22.52 (15.55, 32.73) | 63.39 (43.57, 94.74) | 181.54 | 1307.40 (933.08, 1848.01) | 1299.59 (935.66, 1835.63) | 1251.81 (904.77, 1790.55) | 1225.58 (873.95, 1770.98) | -0.25 (-0.28, -0.22) | -0.08 (-0.15, -0.01) | -0.40 (-0.45, -0.34) | -0.29 (-0.49, -0.09) |
| Libya | 47.61 (31.54, 74.09) | 77.88 (54.73, 113.41) | 63.56 | 1068.04 (744.83, 1571.39) | 1041.62 (724.56, 1515.76) | 1036.11 (712.15, 1512.38) | 1013.26 (702.52, 1489.49) | -0.17 (-0.19, -0.15) | -0.29 (-0.36, -0.21) | -0.04 (-0.12, 0.05) | -0.24 (-0.26, -0.22) |
| Lithuania | 72.17 (50.34, 105.87) | 42.00 (29.79, 60.31) | -41.80 | 1982.67 (1355.76, 2947.29) | 1839.16 (1262.75, 2712.10) | 1878.62 (1294.90, 2795.89) | 1872.78 (1275.25, 2755.43) | -0.23 (-0.30, -0.16) | -0.79 (-1.00, -0.58) | 0.24 (0.09, 0.39) | -0.20 (-0.44, 0.04) |
| Luxembourg | 0.52 (0.39, 0.70) | 0.77 (0.57, 1.04) | 48.33 | 133.93 (96.52, 185.67) | 130.92 (96.13, 177.68) | 128.83 (93.50, 175.67) | 125.65 (90.68, 173.05) | -0.23 (-0.24, -0.21) | -0.24 (-0.31, -0.17) | -0.16 (-0.20, -0.12) | -0.33 (-0.41, -0.25) |
| Madagascar | 157.47 (104.55, 242.38) | 369.23 (245.44, 552.17) | 134.47 | 1297.47 (912.71, 1908.00) | 1297.78 (923.79, 1878.86) | 1292.37 (912.70, 1859.14) | 1259.88 (887.30, 1820.67) | -0.12 (-0.15, -0.08) | -0.02 (-0.14, 0.10) | -0.04 (-0.11, 0.03) | -0.42 (-0.64, -0.20) |
| Malawi | 161.41 (106.17, 248.57) | 336.30 (218.06, 500.28) | 108.34 | 1616.98 (1123.09, 2376.84) | 2064.35 (1736.86, 2432.11) | 1615.89 (1128.80, 2369.42) | 1598.21 (1100.61, 2275.21) | -0.29 (-0.75, 0.16) | 2.61 (1.69, 3.54) | -2.44 (-3.51, -1.36) | -0.18 (-0.26, -0.10) |
| Malaysia | 233.92 (161.57, 334.34) | 441.53 (311.22, 643.48) | 88.75 | 1239.72 (882.41, 1746.87) | 1283.11 (927.20, 1812.83) | 1261.71 (907.79, 1799.14) | 1256.69 (884.13, 1822.49) | -0.10 (-0.16, -0.04) | 0.25 (-0.12, 0.63) | -0.17 (-0.35, 0.02) | -0.10 (-0.19, -0.01) |
| Maldives | 2.88 (1.98, 4.23) | 8.56 (6.08, 12.17) | 197.02 | 1334.07 (965.69, 1895.28) | 1331.36 (945.78, 1871.62) | 1335.49 (951.69, 1894.90) | 1433.67 (1029.24, 2011.99) | 0.11 (-0.02, 0.24) | -0.10 (-0.51, 0.31) | 0.00 (-0.15, 0.15) | 0.79 (0.61, 0.98) |
| Mali | 96.22 (66.28, 143.25) | 248.18 (167.03, 371.32) | 157.94 | 1224.00 (877.37, 1765.72) | 1208.59 (882.04, 1700.45) | 1157.94 (825.78, 1668.21) | 1149.38 (822.64, 1634.71) | -0.25 (-0.28, -0.21) | -0.12 (-0.16, -0.08) | -0.46 (-0.57, -0.35) | -0.19 (-0.47, 0.10) |
| Malta | 0.48 (0.35, 0.66) | 0.49 (0.36, 0.65) | 0.23 | 130.47 (94.24, 183.26) | 128.99 (93.19, 176.85) | 126.71 (92.27, 172.64) | 123.98 (90.45, 171.65) | -0.19 (-0.21, -0.18) | -0.13 (-0.21, -0.04) | -0.18 (-0.24, -0.12) | -0.29 (-0.38, -0.20) |
| Marshall Islands | 0.50 (0.33, 0.76) | 0.63 (0.43, 0.94) | 27.99 | 1071.81 (747.96, 1573.47) | 1094.05 (758.64, 1626.99) | 1061.50 (734.56, 1573.93) | 1028.27 (704.10, 1510.82) | -0.20 (-0.25, -0.16) | 0.18 (0.05, 0.31) | -0.31 (-0.38, -0.23) | -0.35 (-0.38, -0.31) |
| Mauritania | 26.64 (17.96, 39.88) | 51.56 (34.78, 77.17) | 93.57 | 1327.40 (944.17, 1894.41) | 1303.11 (940.62, 1841.30) | 1241.37 (882.85, 1788.44) | 1214.66 (867.42, 1755.04) | -0.35 (-0.38, -0.32) | -0.20 (-0.30, -0.10) | -0.51 (-0.58, -0.44) | -0.31 (-0.51, -0.12) |
| Mauritius | 15.55 (10.84, 22.42) | 14.79 (10.76, 20.88) | -4.90 | 1242.18 (887.66, 1767.62) | 1184.24 (835.62, 1675.22) | 1158.94 (830.65, 1624.88) | 1150.96 (821.22, 1632.15) | -0.30 (-0.34, -0.26) | -0.52 (-0.72, -0.31) | -0.21 (-0.34, -0.09) | -0.08 (-0.12, -0.03) |
| Mexico | 817.98 (553.78, 1200.26) | 1108.51 (760.86, 1573.84) | 35.52 | 870.07 (600.69, 1239.15) | 845.12 (581.58, 1187.96) | 832.11 (572.82, 1178.38) | 833.41 (571.92, 1182.38) | -0.11 (-0.13, -0.08) | -0.27 (-0.37, -0.17) | -0.16 (-0.18, -0.14) | 0.03 (-0.04, 0.10) |
| Micronesia (Federated States of) | 1.17 (0.78, 1.79) | 1.20 (0.81, 1.83) | 2.52 | 1101.97 (760.63, 1605.43) | 1111.28 (781.30, 1684.82) | 1061.37 (731.12, 1577.79) | 1037.55 (718.00, 1540.52) | -0.30 (-0.34, -0.26) | 0.04 (-0.16, 0.23) | -0.47 (-0.58, -0.36) | -0.26 (-0.36, -0.16) |
| Monaco | 0.03 (0.02, 0.04) | 0.04 (0.03, 0.05) | 14.07 | 122.83 (88.20, 170.32) | 122.30 (88.46, 168.97) | 121.74 (88.10, 168.13) | 120.88 (86.53, 167.33) | -0.06 (-0.07, -0.05) | -0.05 (-0.08, -0.01) | -0.05 (-0.07, -0.03) | -0.13 (-0.21, -0.05) |
| Mongolia | 57.56 (37.79, 88.11) | 77.27 (53.76, 112.62) | 34.25 | 2391.59 (1639.97, 3550.55) | 2567.13 (1935.32, 3435.79) | 2523.30 (1907.69, 3446.32) | 2321.01 (1567.22, 3461.88) | -0.13 (-0.28, 0.02) | 0.76 (0.54, 0.98) | -0.17 (-0.23, -0.11) | -1.05 (-1.42, -0.69) |
| Montenegro | 12.40 (8.55, 18.16) | 10.49 (7.38, 15.26) | -15.38 | 1903.34 (1314.73, 2789.76) | 1936.31 (1343.22, 2870.92) | 1894.07 (1287.23, 2792.09) | 1896.25 (1291.88, 2807.11) | -0.12 (-0.16, -0.09) | 0.16 (0.09, 0.23) | -0.23 (-0.26, -0.19) | -0.14 (-0.33, 0.06) |
| Morocco | 329.37 (219.43, 504.41) | 419.38 (292.02, 617.12) | 27.33 | 1181.61 (824.50, 1746.04) | 1179.10 (822.33, 1705.72) | 1113.48 (787.51, 1627.91) | 1098.41 (761.86, 1619.06) | -0.37 (-0.41, -0.33) | -0.05 (-0.16, 0.07) | -0.59 (-0.64, -0.53) | -0.16 (-0.23, -0.10) |
| Mozambique | 201.00 (133.11, 303.99) | 463.41 (307.56, 708.73) | 130.55 | 1597.91 (1103.52, 2346.01) | 1670.95 (1291.10, 2178.97) | 1529.68 (1070.04, 2230.98) | 1494.31 (1051.46, 2166.92) | -0.46 (-0.56, -0.35) | 0.37 (0.05, 0.70) | -0.90 (-1.21, -0.60) | -0.35 (-0.51, -0.20) |
| Myanmar | 593.75 (412.73, 868.92) | 744.54 (519.83, 1052.17) | 25.40 | 1362.60 (977.11, 1939.17) | 1380.15 (991.36, 1953.49) | 1325.11 (944.57, 1861.24) | 1277.92 (893.96, 1807.32) | -0.38 (-0.44, -0.31) | 0.04 (-0.34, 0.42) | -0.40 (-0.60, -0.19) | -0.46 (-0.53, -0.39) |
| Namibia | 42.49 (28.32, 64.76) | 68.86 (46.31, 102.37) | 62.05 | 2788.18 (1974.87, 4045.78) | 2773.94 (1944.60, 4036.39) | 2619.35 (1830.94, 3781.71) | 2563.68 (1772.40, 3732.33) | -0.38 (-0.42, -0.33) | -0.06 (-0.14, 0.02) | -0.60 (-0.71, -0.48) | -0.39 (-0.61, -0.17) |
| Nauru | 0.11 (0.07, 0.16) | 0.12 (0.08, 0.18) | 10.23 | 995.80 (684.54, 1488.06) | 1001.08 (692.89, 1497.80) | 986.74 (676.96, 1457.25) | 968.26 (662.77, 1422.93) | -0.14 (-0.17, -0.11) | 0.03 (-0.03, 0.10) | -0.14 (-0.20, -0.08) | -0.18 (-0.30, -0.06) |
| Nepal | 132.02 (88.54, 198.32) | 209.42 (140.84, 314.81) | 58.62 | 676.11 (474.84, 999.58) | 662.52 (462.56, 963.58) | 627.16 (433.91, 923.14) | 598.52 (417.63, 878.56) | -0.48 (-0.52, -0.44) | -0.23 (-0.47, 0.00) | -0.56 (-0.58, -0.54) | -0.50 (-0.64, -0.35) |
| Netherlands | 20.27 (14.74, 27.40) | 19.77 (14.75, 26.38) | -2.47 | 129.80 (93.88, 177.70) | 132.55 (95.65, 180.93) | 124.36 (89.06, 169.46) | 123.94 (89.68, 167.65) | -0.29 (-0.35, -0.24) | 0.20 (0.11, 0.29) | -0.67 (-0.70, -0.63) | -0.06 (-0.10, -0.02) |
| New Zealand | 13.01 (9.24, 17.71) | 14.03 (10.20, 19.14) | 7.90 | 365.26 (261.22, 500.02) | 354.32 (252.29, 492.20) | 343.57 (242.17, 476.42) | 347.32 (246.78, 476.79) | -0.19 (-0.22, -0.16) | -0.30 (-0.31, -0.29) | -0.30 (-0.42, -0.17) | 0.07 (-0.09, 0.23) |
| Nicaragua | 47.22 (31.02, 71.19) | 75.86 (52.30, 110.79) | 60.64 | 1169.68 (812.07, 1682.16) | 1137.72 (801.58, 1664.57) | 1062.18 (748.04, 1536.81) | 1040.68 (730.60, 1499.07) | -0.42 (-0.45, -0.38) | -0.26 (-0.31, -0.21) | -0.72 (-0.75, -0.69) | -0.13 (-0.26, 0.00) |
| Niger | 123.97 (82.72, 188.07) | 315.14 (199.44, 484.99) | 154.20 | 1665.33 (1167.39, 2412.35) | 1590.43 (1107.77, 2340.26) | 1524.37 (1068.81, 2209.73) | 1431.21 (985.82, 2098.84) | -0.49 (-0.54, -0.44) | -0.48 (-0.67, -0.29) | -0.44 (-0.49, -0.38) | -0.77 (-1.15, -0.39) |
| Nigeria | 1185.93 (825.17, 1657.37) | 2697.32 (1865.09, 3841.27) | 127.44 | 1360.82 (978.59, 1845.27) | 1327.38 (948.07, 1812.93) | 1262.13 (898.29, 1726.38) | 1240.88 (880.72, 1713.02) | -0.35 (-0.37, -0.32) | -0.25 (-0.27, -0.23) | -0.52 (-0.60, -0.45) | -0.27 (-0.47, -0.07) |
| Niue | 0.02 (0.01, 0.03) | 0.01 (0.01, 0.02) | -31.39 | 969.19 (672.11, 1441.76) | 975.25 (676.93, 1436.86) | 955.31 (651.66, 1433.30) | 944.09 (648.56, 1408.14) | -0.15 (-0.17, -0.12) | 0.04 (-0.10, 0.17) | -0.22 (-0.29, -0.15) | -0.09 (-0.14, -0.04) |
| North Macedonia | 38.82 (26.46, 56.67) | 36.11 (25.39, 52.84) | -6.96 | 1833.48 (1247.38, 2683.36) | 1863.54 (1276.91, 2756.44) | 1831.58 (1276.29, 2664.56) | 1831.64 (1255.91, 2729.02) | -0.08 (-0.10, -0.05) | 0.15 (0.12, 0.18) | -0.18 (-0.20, -0.16) | -0.11 (-0.25, 0.02) |
| Northern Mariana Islands | 0.59 (0.40, 0.86) | 0.42 (0.29, 0.64) | -27.76 | 1033.50 (707.73, 1522.52) | 1066.46 (732.81, 1598.22) | 986.55 (686.40, 1458.68) | 955.44 (658.12, 1415.59) | -0.36 (-0.44, -0.29) | 0.38 (0.28, 0.48) | -0.61 (-0.79, -0.44) | -0.32 (-0.36, -0.29) |
| Norway | 6.09 (4.44, 8.21) | 7.75 (5.74, 10.42) | 27.33 | 143.64 (104.09, 193.46) | 150.74 (108.95, 202.04) | 152.88 (110.78, 206.30) | 151.01 (109.64, 204.10) | 0.20 (0.13, 0.26) | 0.51 (0.36, 0.66) | 0.14 (0.09, 0.19) | -0.12 (-0.22, -0.03) |
| Oman | 22.48 (15.15, 33.56) | 62.48 (42.97, 90.22) | 177.94 | 1132.59 (777.83, 1672.36) | 1087.14 (759.15, 1591.56) | 1077.70 (750.98, 1572.66) | 1087.12 (755.47, 1586.76) | -0.13 (-0.19, -0.07) | -0.42 (-0.52, -0.32) | -0.09 (-0.19, 0.00) | -0.02 (-0.20, 0.16) |
| Pakistan | 416.95 (283.49, 599.70) | 898.70 (609.39, 1283.41) | 115.54 | 385.26 (270.66, 538.55) | 390.14 (271.85, 545.50) | 370.24 (259.50, 511.67) | 375.14 (259.26, 524.56) | -0.20 (-0.27, -0.12) | 0.07 (-0.19, 0.33) | -0.54 (-0.62, -0.46) | 0.23 (-0.08, 0.53) |
| Palau | 0.18 (0.12, 0.27) | 0.16 (0.11, 0.23) | -10.01 | 984.23 (669.91, 1474.08) | 973.68 (665.75, 1447.90) | 960.14 (658.85, 1429.11) | 954.18 (655.77, 1413.94) | -0.16 (-0.18, -0.14) | -0.13 (-0.25, -0.02) | -0.12 (-0.22, -0.02) | -0.03 (-0.14, 0.08) |
| Palestine | 22.81 (14.86, 36.01) | 58.21 (38.86, 89.03) | 155.21 | 1074.68 (740.48, 1612.69) | 1090.91 (755.85, 1598.77) | 1055.30 (728.23, 1561.16) | 1030.83 (715.92, 1531.26) | -0.19 (-0.23, -0.14) | 0.14 (0.07, 0.21) | -0.34 (-0.45, -0.23) | -0.22 (-0.27, -0.18) |
| Panama | 19.01 (12.61, 28.75) | 30.86 (21.26, 45.00) | 62.33 | 708.67 (487.02, 1041.39) | 737.15 (510.81, 1095.34) | 740.08 (515.31, 1087.79) | 722.03 (498.95, 1049.92) | 0.10 (0.04, 0.16) | 0.41 (0.33, 0.49) | 0.04 (-0.02, 0.09) | -0.32 (-0.42, -0.21) |
| Papua New Guinea | 90.38 (58.59, 140.98) | 202.95 (132.43, 315.57) | 124.55 | 2019.17 (1377.34, 3029.88) | 2026.76 (1387.12, 3001.03) | 2149.16 (1464.63, 3216.52) | 1872.60 (1251.67, 2877.32) | 0.22 (0.09, 0.35) | 0.07 (-0.05, 0.18) | 0.62 (0.27, 0.97) | -0.70 (-1.73, 0.34) |
| Paraguay | 35.71 (23.82, 54.55) | 63.71 (43.21, 95.52) | 78.40 | 851.36 (588.64, 1266.78) | 882.93 (606.67, 1300.30) | 824.62 (568.27, 1204.48) | 825.91 (568.31, 1221.82) | -0.20 (-0.28, -0.12) | 0.38 (0.31, 0.46) | -0.72 (-0.89, -0.56) | -0.09 (-0.29, 0.12) |
| Peru | 59.89 (40.99, 88.26) | 90.94 (64.09, 130.53) | 51.83 | 259.33 (185.13, 367.71) | 229.15 (169.58, 311.29) | 258.68 (183.57, 363.31) | 255.35 (180.11, 365.34) | 0.02 (-0.23, 0.26) | -1.29 (-1.93, -0.64) | 1.28 (0.65, 1.91) | -0.17 (-0.20, -0.13) |
| Philippines | 889.33 (627.61, 1251.49) | 1691.87 (1206.80, 2356.55) | 90.24 | 1343.43 (969.35, 1845.28) | 1435.01 (1031.93, 1971.01) | 1405.64 (1012.57, 1940.50) | 1406.85 (1009.36, 1939.84) | -0.06 (-0.14, 0.02) | 0.52 (0.00, 1.04) | -0.21 (-0.33, -0.08) | 0.03 (-0.05, 0.10) |
| Poland | 708.82 (496.19, 983.82) | 579.66 (416.32, 793.30) | -18.22 | 1974.83 (1351.80, 2792.51) | 1904.24 (1309.52, 2697.27) | 1841.18 (1263.87, 2585.60) | 1805.44 (1255.82, 2517.37) | -0.36 (-0.39, -0.34) | -0.36 (-0.41, -0.31) | -0.33 (-0.44, -0.23) | -0.32 (-0.45, -0.19) |
| Portugal | 13.65 (10.10, 18.50) | 12.06 (9.06, 16.18) | -11.68 | 131.56 (96.85, 180.12) | 131.30 (95.26, 180.70) | 128.17 (93.01, 174.62) | 125.29 (90.79, 172.54) | -0.22 (-0.26, -0.18) | -0.03 (-0.17, 0.11) | -0.24 (-0.41, -0.07) | -0.28 (-0.34, -0.22) |
| Puerto Rico | 43.64 (30.06, 65.86) | 35.40 (25.08, 52.00) | -18.89 | 1156.97 (804.98, 1731.84) | 1200.53 (832.90, 1803.42) | 1147.78 (794.43, 1755.97) | 1154.29 (800.32, 1732.99) | -0.17 (-0.23, -0.11) | 0.36 (0.32, 0.41) | -0.46 (-0.68, -0.24) | -0.04 (-0.26, 0.18) |
| Qatar | 6.09 (4.27, 8.66) | 44.05 (30.49, 63.60) | 623.04 | 1157.07 (805.91, 1697.85) | 1121.23 (777.25, 1657.64) | 1149.96 (809.83, 1666.22) | 1145.71 (798.65, 1666.13) | 0.00 (-0.06, 0.05) | -0.34 (-0.44, -0.23) | 0.31 (0.21, 0.40) | -0.08 (-0.12, -0.04) |
| Republic of Korea | 331.43 (232.90, 479.25) | 307.45 (227.85, 418.24) | -7.23 | 636.36 (457.28, 906.04) | 591.69 (436.31, 805.58) | 612.31 (440.02, 855.66) | 613.57 (438.53, 861.29) | -0.15 (-0.27, -0.02) | -0.77 (-1.16, -0.38) | 0.35 (0.14, 0.57) | -0.05 (-0.15, 0.05) |
| Republic of Moldova | 86.94 (60.52, 127.29) | 60.98 (43.39, 87.51) | -29.85 | 2021.60 (1398.80, 3013.97) | 1989.40 (1381.44, 2940.78) | 1934.66 (1317.45, 2845.52) | 1913.64 (1297.61, 2834.94) | -0.26 (-0.29, -0.23) | -0.15 (-0.16, -0.13) | -0.28 (-0.38, -0.19) | -0.35 (-0.61, -0.09) |
| Romania | 438.19 (300.11, 651.61) | 270.39 (194.81, 386.06) | -38.29 | 1898.70 (1295.80, 2829.16) | 1956.69 (1348.22, 2879.86) | 1837.24 (1253.03, 2736.26) | 1816.48 (1233.66, 2722.11) | -0.27 (-0.35, -0.19) | 0.32 (0.24, 0.41) | -0.66 (-0.74, -0.57) | -0.25 (-0.42, -0.08) |
| Russian Federation | 3139.99 (2198.37, 4418.83) | 2579.78 (1816.62, 3570.53) | -17.84 | 2158.23 (1475.57, 3078.35) | 2192.85 (1500.06, 3148.55) | 2146.75 (1467.74, 3057.60) | 2168.09 (1464.72, 3121.69) | -0.16 (-0.21, -0.11) | 0.13 (0.01, 0.26) | -0.21 (-0.22, -0.20) | -0.05 (-0.40, 0.30) |
| Rwanda | 95.56 (63.30, 144.87) | 172.92 (116.58, 265.53) | 80.95 | 1327.69 (931.58, 1924.14) | 1318.00 (937.97, 1932.09) | 1256.55 (890.27, 1854.64) | 1218.81 (855.22, 1785.49) | -0.37 (-0.40, -0.33) | -0.09 (-0.19, 0.01) | -0.50 (-0.54, -0.45) | -0.45 (-0.61, -0.30) |
| Saint Kitts and Nevis | 0.53 (0.34, 0.83) | 0.69 (0.49, 1.03) | 31.40 | 1181.89 (815.35, 1780.58) | 1195.85 (824.69, 1836.05) | 1138.63 (784.21, 1718.13) | 1158.65 (797.23, 1751.23) | -0.15 (-0.20, -0.10) | 0.16 (-0.02, 0.34) | -0.51 (-0.57, -0.46) | 0.17 (-0.02, 0.36) |
| Saint Lucia | 1.95 (1.28, 3.04) | 2.21 (1.56, 3.30) | 13.24 | 1274.45 (874.48, 1902.02) | 1303.31 (900.23, 1955.31) | 1220.40 (839.14, 1827.26) | 1228.35 (849.11, 1847.53) | -0.25 (-0.31, -0.18) | 0.25 (0.11, 0.39) | -0.69 (-0.72, -0.66) | 0.02 (-0.16, 0.20) |
| Saint Vincent and the Grenadines | 1.62 (1.07, 2.50) | 1.43 (0.99, 2.12) | -12.05 | 1307.96 (902.87, 1928.54) | 1319.46 (907.07, 1978.27) | 1231.04 (842.58, 1825.51) | 1254.14 (862.17, 1870.19) | -0.25 (-0.31, -0.18) | 0.13 (-0.04, 0.30) | -0.74 (-0.77, -0.70) | 0.20 (0.06, 0.35) |
| Samoa | 2.19 (1.44, 3.36) | 2.65 (1.75, 4.04) | 21.27 | 1176.60 (817.03, 1723.71) | 1223.52 (852.15, 1789.60) | 1158.18 (803.45, 1706.56) | 1149.59 (788.89, 1710.11) | -0.17 (-0.25, -0.09) | 0.38 (0.34, 0.42) | -0.57 (-0.81, -0.32) | -0.09 (-0.17, -0.02) |
| San Marino | 0.03 (0.02, 0.04) | 0.04 (0.03, 0.05) | 24.50 | 123.77 (89.47, 170.76) | 120.96 (87.53, 166.88) | 120.19 (86.55, 164.10) | 119.35 (86.17, 165.68) | -0.11 (-0.13, -0.10) | -0.23 (-0.25, -0.21) | -0.06 (-0.07, -0.05) | -0.10 (-0.15, -0.05) |
| Sao Tome and Principe | 1.45 (0.98, 2.20) | 2.72 (1.87, 3.99) | 86.78 | 1245.56 (893.82, 1785.73) | 1243.82 (889.24, 1775.35) | 1225.33 (876.41, 1753.29) | 1208.12 (859.91, 1735.23) | -0.10 (-0.13, -0.08) | -0.03 (-0.10, 0.03) | -0.16 (-0.24, -0.07) | -0.22 (-0.45, 0.01) |
| Saudi Arabia | 177.11 (112.14, 280.08) | 413.85 (281.97, 606.81) | 133.67 | 1011.28 (672.06, 1536.95) | 992.21 (654.77, 1513.53) | 964.04 (636.83, 1448.78) | 948.15 (640.90, 1435.99) | -0.24 (-0.27, -0.21) | -0.22 (-0.32, -0.12) | -0.28 (-0.40, -0.16) | -0.02 (-0.21, 0.17) |
| Senegal | 38.74 (26.90, 57.18) | 74.96 (50.15, 111.34) | 93.51 | 528.03 (383.39, 748.78) | 323.31 (258.78, 408.06) | 471.75 (330.47, 669.52) | 469.51 (334.52, 667.37) | -0.16 (-0.99, 0.67) | -4.78 (-6.85, -2.67) | 4.10 (2.30, 5.93) | -0.21 (-0.38, -0.03) |
| Serbia | 168.16 (117.93, 244.46) | 138.05 (96.95, 199.88) | -17.91 | 1888.82 (1297.80, 2808.69) | 1915.20 (1300.90, 2813.78) | 1877.32 (1296.15, 2762.84) | 1872.03 (1268.87, 2788.27) | -0.13 (-0.16, -0.09) | 0.13 (0.06, 0.19) | -0.20 (-0.25, -0.16) | -0.16 (-0.34, 0.02) |
| Seychelles | 1.05 (0.72, 1.53) | 1.43 (1.04, 2.03) | 36.61 | 1311.29 (938.66, 1877.70) | 1351.44 (964.74, 1906.64) | 1367.81 (982.56, 1905.20) | 1376.70 (984.24, 1976.42) | 0.09 (0.05, 0.13) | 0.20 (-0.12, 0.52) | 0.13 (-0.03, 0.29) | 0.07 (-0.06, 0.19) |
| Sierra Leone | 45.24 (31.03, 67.46) | 110.34 (74.93, 166.49) | 143.93 | 1278.27 (912.75, 1828.38) | 1279.57 (912.72, 1838.42) | 1232.11 (888.04, 1784.39) | 1222.90 (876.75, 1771.17) | -0.20 (-0.23, -0.16) | -0.01 (-0.08, 0.06) | -0.41 (-0.52, -0.29) | -0.17 (-0.42, 0.07) |
| Singapore | 22.52 (16.12, 31.53) | 31.00 (23.02, 42.09) | 37.66 | 616.41 (447.46, 859.87) | 607.45 (434.49, 845.65) | 584.52 (418.62, 821.09) | 566.75 (408.20, 800.88) | -0.35 (-0.38, -0.31) | -0.14 (-0.32, 0.03) | -0.36 (-0.43, -0.29) | -0.43 (-0.66, -0.19) |
| Slovakia | 96.00 (66.77, 139.37) | 83.68 (59.59, 119.14) | -12.83 | 1861.67 (1284.55, 2743.47) | 1891.97 (1305.71, 2795.42) | 1878.75 (1275.65, 2780.98) | 1866.73 (1254.96, 2800.61) | -0.05 (-0.08, -0.02) | 0.15 (0.13, 0.18) | -0.07 (-0.08, -0.06) | -0.19 (-0.34, -0.05) |
| Slovenia | 35.31 (24.40, 51.31) | 25.44 (18.34, 36.24) | -27.96 | 1806.08 (1222.24, 2676.27) | 1787.70 (1211.32, 2655.59) | 1693.33 (1159.20, 2493.33) | 1669.31 (1131.59, 2487.25) | -0.38 (-0.41, -0.35) | -0.10 (-0.14, -0.06) | -0.56 (-0.57, -0.54) | -0.28 (-0.44, -0.12) |
| Solomon Islands | 3.21 (1.95, 5.25) | 6.05 (3.71, 9.85) | 88.33 | 905.96 (582.19, 1411.45) | 904.77 (586.34, 1393.64) | 867.58 (561.45, 1345.80) | 864.34 (544.86, 1373.06) | -0.35 (-0.41, -0.29) | -0.11 (-0.52, 0.31) | -0.42 (-0.63, -0.20) | -0.06 (-0.11, -0.01) |
| Somalia | 54.01 (35.68, 84.49) | 165.46 (107.91, 256.44) | 206.35 | 804.49 (554.27, 1204.50) | 738.33 (542.04, 1025.76) | 756.43 (553.49, 1069.52) | 777.66 (535.52, 1140.10) | -0.05 (-0.20, 0.09) | -0.83 (-0.90, -0.76) | 0.26 (-0.15, 0.67) | -0.02 (-0.50, 0.46) |
| South Africa | 2013.10 (1405.57, 2825.46) | 2749.73 (1954.97, 3834.07) | 36.59 | 4819.31 (3419.87, 6643.62) | 5382.73 (3840.66, 7365.16) | 4861.91 (3448.32, 6644.42) | 4544.89 (3207.20, 6294.96) | -0.47 (-0.65, -0.29) | 0.92 (0.23, 1.61) | -1.04 (-1.46, -0.61) | -0.52 (-0.87, -0.16) |
| South Sudan | 86.85 (57.58, 132.92) | 125.19 (83.75, 192.66) | 44.15 | 1376.32 (970.99, 2017.64) | 1374.46 (979.78, 2033.20) | 1337.54 (942.43, 1924.51) | 1303.21 (921.19, 1898.06) | -0.22 (-0.26, -0.19) | -0.03 (-0.13, 0.07) | -0.28 (-0.34, -0.22) | -0.37 (-0.53, -0.21) |
| Spain | 65.70 (49.32, 89.22) | 63.23 (47.65, 84.82) | -3.76 | 163.59 (121.64, 222.45) | 158.15 (115.45, 213.76) | 156.88 (114.36, 215.21) | 150.66 (109.89, 205.78) | -0.23 (-0.27, -0.19) | -0.34 (-0.39, -0.28) | -0.07 (-0.20, 0.06) | -0.47 (-0.60, -0.33) |
| Sri Lanka | 264.75 (186.49, 378.26) | 301.88 (219.15, 422.75) | 14.03 | 1399.12 (1011.75, 1965.87) | 1447.38 (1038.74, 2057.42) | 1426.71 (1020.41, 2006.31) | 1396.19 (996.66, 1976.35) | -0.06 (-0.12, 0.01) | 0.30 (0.22, 0.37) | -0.14 (-0.33, 0.05) | -0.25 (-0.26, -0.23) |
| Sudan | 291.19 (192.45, 440.77) | 606.05 (399.16, 917.92) | 108.13 | 1396.66 (970.88, 2053.92) | 1446.70 (1038.74, 2038.25) | 1381.01 (957.60, 2003.33) | 1311.51 (898.25, 1906.38) | -0.30 (-0.39, -0.21) | 0.37 (0.33, 0.40) | -0.48 (-0.57, -0.39) | -0.59 (-0.70, -0.48) |
| Suriname | 5.67 (3.75, 8.78) | 7.02 (4.84, 10.45) | 23.82 | 1282.69 (885.44, 1924.92) | 1313.25 (899.07, 1929.94) | 1218.32 (833.57, 1821.96) | 1214.22 (828.08, 1816.67) | -0.33 (-0.41, -0.25) | 0.25 (0.16, 0.35) | -0.78 (-0.85, -0.72) | -0.07 (-0.27, 0.13) |
| Sweden | 12.19 (8.90, 16.69) | 13.20 (9.67, 18.03) | 8.31 | 146.87 (106.63, 200.89) | 147.79 (106.87, 198.43) | 145.22 (106.14, 196.33) | 142.57 (103.27, 195.46) | -0.11 (-0.14, -0.07) | 0.07 (0.02, 0.12) | -0.18 (-0.26, -0.10) | -0.24 (-0.29, -0.20) |
| Switzerland | 8.80 (6.46, 11.78) | 9.97 (7.48, 13.32) | 13.30 | 126.02 (90.66, 170.49) | 126.66 (92.08, 173.78) | 124.21 (90.74, 168.57) | 122.31 (88.13, 171.22) | -0.15 (-0.17, -0.13) | 0.03 (-0.08, 0.13) | -0.20 (-0.24, -0.16) | -0.21 (-0.29, -0.14) |
| Syrian Arab Republic | 143.43 (93.85, 219.34) | 156.31 (104.82, 245.42) | 8.98 | 1076.65 (741.61, 1605.44) | 1078.29 (748.10, 1581.23) | 1018.90 (701.38, 1507.39) | 969.49 (672.82, 1456.81) | -0.44 (-0.51, -0.38) | -0.01 (-0.10, 0.09) | -0.57 (-0.72, -0.42) | -0.54 (-0.65, -0.42) |
| Taiwan (Province of China) | 206.16 (142.29, 302.59) | 179.95 (129.53, 259.37) | -12.72 | 890.35 (624.32, 1292.65) | 882.55 (616.23, 1296.65) | 862.27 (598.36, 1250.55) | 856.24 (595.30, 1271.78) | -0.17 (-0.18, -0.15) | -0.10 (-0.19, -0.02) | -0.24 (-0.29, -0.19) | -0.06 (-0.13, 0.00) |
| Tajikistan | 150.02 (97.95, 228.62) | 270.63 (183.34, 402.36) | 80.39 | 2623.06 (1785.88, 3872.40) | 2667.38 (1836.33, 3934.60) | 2591.08 (1783.57, 3794.62) | 2552.87 (1750.36, 3757.90) | -0.18 (-0.21, -0.14) | 0.15 (0.07, 0.23) | -0.30 (-0.38, -0.22) | -0.25 (-0.34, -0.15) |
| Thailand | 815.99 (567.71, 1186.84) | 824.92 (604.24, 1144.98) | 1.09 | 1228.69 (877.69, 1757.32) | 1247.59 (890.74, 1772.19) | 1140.92 (829.60, 1571.74) | 1213.19 (865.08, 1729.28) | -0.29 (-0.44, -0.14) | 0.07 (-0.34, 0.48) | -0.91 (-1.18, -0.64) | 0.66 (0.24, 1.09) |
| Timor-Leste | 11.20 (7.65, 16.42) | 18.49 (12.59, 27.38) | 65.09 | 1380.77 (976.54, 1951.68) | 1401.70 (1005.09, 1976.95) | 1327.38 (954.49, 1893.70) | 1287.66 (922.53, 1845.85) | -0.42 (-0.49, -0.35) | 0.06 (-0.36, 0.49) | -0.54 (-0.71, -0.37) | -0.38 (-0.49, -0.27) |
| Togo | 46.07 (31.12, 70.34) | 99.38 (68.30, 148.15) | 115.74 | 1277.88 (911.44, 1864.05) | 1286.72 (924.49, 1823.37) | 1235.81 (885.47, 1793.50) | 1200.10 (857.11, 1734.95) | -0.24 (-0.28, -0.19) | 0.05 (0.00, 0.11) | -0.42 (-0.50, -0.34) | -0.39 (-0.63, -0.15) |
| Tokelau | 0.02 (0.01, 0.02) | 0.01 (0.01, 0.02) | -16.88 | 1006.64 (692.56, 1495.85) | 1007.47 (694.20, 1501.16) | 977.15 (673.01, 1434.43) | 968.56 (660.25, 1460.67) | -0.22 (-0.25, -0.19) | -0.03 (-0.14, 0.08) | -0.32 (-0.36, -0.28) | -0.03 (-0.20, 0.15) |
| Tonga | 1.29 (0.84, 1.98) | 1.26 (0.84, 1.92) | -2.34 | 1231.02 (850.12, 1802.89) | 1284.37 (927.64, 1820.87) | 1240.46 (881.81, 1791.43) | 1182.66 (809.00, 1755.04) | -0.21 (-0.29, -0.13) | 0.42 (0.38, 0.46) | -0.35 (-0.39, -0.31) | -0.58 (-0.68, -0.48) |
| Trinidad and Tobago | 14.85 (10.00, 22.65) | 14.57 (10.47, 21.40) | -1.86 | 1150.45 (788.63, 1739.88) | 1251.24 (854.79, 1897.43) | 1115.33 (765.15, 1680.11) | 1144.13 (786.71, 1727.38) | -0.22 (-0.37, -0.07) | 0.89 (0.62, 1.17) | -1.24 (-1.40, -1.07) | 0.25 (0.11, 0.39) |
| Tunisia | 139.72 (99.35, 197.88) | 140.89 (100.35, 199.94) | 0.84 | 1485.49 (1086.08, 2043.94) | 1280.51 (892.89, 1872.82) | 1279.85 (880.65, 1894.68) | 1255.23 (875.33, 1810.43) | -0.54 (-0.68, -0.41) | -1.54 (-1.85, -1.22) | 0.01 (-0.04, 0.06) | -0.22 (-0.25, -0.19) |
| Turkey | 1279.17 (891.16, 1843.70) | 1323.46 (930.96, 1889.37) | 3.46 | 1904.99 (1363.31, 2678.83) | 1566.29 (1093.84, 2287.86) | 1550.09 (1069.62, 2282.78) | 1519.36 (1052.83, 2207.97) | -0.33 (-0.51, -0.16) | -1.68 (-2.73, -0.62) | -0.10 (-0.14, -0.07) | -0.20 (-0.28, -0.13) |
| Turkmenistan | 101.45 (67.76, 156.71) | 111.24 (75.39, 166.83) | 9.65 | 2495.25 (1719.51, 3730.59) | 2366.82 (1617.01, 3535.41) | 2167.70 (1475.59, 3204.25) | 2120.13 (1429.39, 3195.75) | -0.79 (-0.86, -0.72) | -0.57 (-0.99, -0.14) | -0.88 (-0.93, -0.84) | -0.35 (-0.49, -0.21) |
| Tuvalu | 0.10 (0.07, 0.15) | 0.13 (0.08, 0.19) | 31.09 | 1014.47 (704.55, 1530.59) | 1000.38 (681.77, 1492.74) | 973.17 (673.95, 1430.66) | 971.29 (662.27, 1458.81) | -0.19 (-0.22, -0.17) | -0.15 (-0.22, -0.08) | -0.28 (-0.39, -0.16) | 0.04 (-0.03, 0.12) |
| Uganda | 464.69 (317.20, 688.04) | 1187.99 (791.09, 1792.93) | 155.65 | 2723.24 (1958.27, 3769.68) | 3213.54 (2430.20, 4311.27) | 2651.30 (1908.34, 3703.62) | 2740.24 (1951.00, 3900.50) | -0.18 (-0.47, 0.11) | 1.76 (1.08, 2.45) | -2.00 (-2.58, -1.41) | 0.38 (0.23, 0.53) |
| Ukraine | 1083.75 (750.31, 1533.89) | 747.23 (531.75, 1027.30) | -31.05 | 2214.94 (1511.47, 3171.91) | 2279.26 (1544.14, 3332.04) | 1997.04 (1352.75, 2840.21) | 2062.33 (1399.73, 2951.39) | -0.61 (-0.73, -0.50) | 0.24 (-0.05, 0.53) | -1.41 (-1.49, -1.33) | 0.22 (0.03, 0.41) |
| United Arab Emirates | 24.60 (16.94, 35.14) | 100.59 (72.34, 138.51) | 308.84 | 1138.42 (785.84, 1667.50) | 1142.08 (790.26, 1683.81) | 1154.25 (798.23, 1708.17) | 1085.89 (756.95, 1567.86) | -0.12 (-0.18, -0.06) | 0.01 (-0.10, 0.11) | 0.16 (0.09, 0.23) | -0.72 (-0.77, -0.66) |
| United Kingdom | 78.93 (61.73, 102.18) | 87.05 (67.14, 113.06) | 10.29 | 138.62 (108.04, 179.55) | 135.24 (105.37, 174.48) | 108.09 (84.11, 137.61) | 138.14 (106.24, 179.42) | -0.84 (-1.18, -0.51) | -0.23 (-0.26, -0.20) | -2.31 (-3.28, -1.34) | 1.93 (0.71, 3.16) |
| United Republic of Tanzania | 441.16 (292.45, 672.56) | 890.89 (604.52, 1333.13) | 101.94 | 1695.44 (1202.73, 2458.01) | 1507.99 (1122.07, 2049.71) | 1612.29 (1151.69, 2351.83) | 1520.59 (1083.95, 2162.68) | -0.29 (-0.49, -0.09) | -1.26 (-2.00, -0.51) | 0.70 (0.36, 1.03) | -0.48 (-0.79, -0.17) |
| United States of America | 984.56 (708.27, 1330.55) | 1177.84 (857.22, 1599.88) | 19.63 | 382.38 (273.75, 519.22) | 343.85 (246.88, 469.31) | 344.06 (244.90, 469.44) | 385.65 (276.75, 525.63) | -0.30 (-0.45, -0.14) | -1.09 (-1.56, -0.62) | 0.01 (-0.17, 0.18) | 0.83 (0.14, 1.53) |
| United States Virgin Islands | 1.33 (0.92, 1.98) | 1.02 (0.72, 1.48) | -23.53 | 1244.13 (857.03, 1863.51) | 1281.97 (887.49, 1912.68) | 1191.01 (824.18, 1793.52) | 1218.21 (835.96, 1815.11) | -0.23 (-0.30, -0.15) | 0.31 (0.21, 0.41) | -0.78 (-0.82, -0.75) | 0.23 (0.09, 0.37) |
| Uruguay | 13.87 (10.07, 19.38) | 13.99 (10.17, 19.54) | 0.85 | 454.35 (327.93, 638.21) | 446.73 (321.96, 629.47) | 430.33 (306.99, 600.15) | 425.40 (303.86, 603.56) | -0.28 (-0.30, -0.26) | -0.18 (-0.26, -0.10) | -0.39 (-0.43, -0.34) | -0.21 (-0.34, -0.08) |
| Uzbekistan | 567.16 (371.68, 876.56) | 819.80 (547.93, 1231.44) | 44.55 | 2504.94 (1703.77, 3792.05) | 2537.04 (1731.72, 3808.74) | 2271.14 (1547.77, 3357.10) | 2224.38 (1500.06, 3334.86) | -0.59 (-0.67, -0.51) | 0.13 (0.12, 0.15) | -1.17 (-1.35, -1.00) | -0.31 (-0.42, -0.21) |
| Vanuatu | 1.94 (1.29, 2.99) | 3.73 (2.51, 5.58) | 92.25 | 1234.04 (861.93, 1835.69) | 1277.29 (888.19, 1863.06) | 1243.53 (866.57, 1818.67) | 1180.43 (817.15, 1716.21) | -0.21 (-0.30, -0.13) | 0.33 (0.29, 0.38) | -0.26 (-0.35, -0.17) | -0.66 (-0.79, -0.53) |
| Venezuela (Bolivarian Republic of) | 158.11 (106.16, 239.55) | 206.89 (146.01, 300.46) | 30.85 | 757.29 (526.71, 1117.48) | 800.24 (552.78, 1175.02) | 743.53 (513.84, 1087.95) | 742.07 (517.84, 1089.69) | -0.19 (-0.28, -0.10) | 0.57 (0.50, 0.65) | -0.79 (-0.86, -0.71) | -0.08 (-0.20, 0.03) |
| Viet Nam | 806.08 (551.37, 1171.71) | 1147.14 (819.54, 1587.80) | 42.31 | 1113.07 (798.21, 1567.80) | 1161.28 (823.20, 1642.63) | 1124.24 (797.67, 1595.25) | 1122.22 (799.80, 1594.18) | -0.18 (-0.25, -0.10) | 0.33 (-0.15, 0.82) | -0.33 (-0.50, -0.16) | 0.01 (-0.04, 0.06) |
| Yemen | 140.45 (92.89, 217.67) | 368.97 (244.25, 559.97) | 162.71 | 1130.45 (781.01, 1668.15) | 1121.24 (772.48, 1654.03) | 1110.84 (760.52, 1623.93) | 1090.06 (751.14, 1610.08) | -0.15 (-0.17, -0.13) | -0.11 (-0.25, 0.03) | -0.09 (-0.19, 0.01) | -0.18 (-0.22, -0.14) |
| Zambia | 111.02 (72.81, 173.04) | 255.58 (169.54, 391.82) | 130.20 | 1309.43 (909.70, 1935.10) | 1325.03 (1033.23, 1699.74) | 1300.93 (905.91, 1894.80) | 1259.24 (875.16, 1857.24) | -0.14 (-0.19, -0.10) | 0.09 (0.00, 0.17) | -0.19 (-0.30, -0.07) | -0.43 (-0.61, -0.24) |
| Zimbabwe | 233.06 (154.15, 362.62) | 336.83 (223.74, 514.54) | 44.52 | 2181.14 (1512.24, 3210.49) | 2161.23 (1598.39, 2911.84) | 2170.04 (1495.28, 3217.47) | 2062.80 (1426.17, 3029.59) | -0.13 (-0.18, -0.07) | -0.09 (-0.10, -0.08) | 0.05 (0.04, 0.06) | -0.71 (-0.98, -0.44) |
| **Trichomoniasis** | |  |  |  |  |  |  |  |  |  |  |
| Afghanistan | 269.03 (203.37, 347.90) | 954.51 (706.22, 1264.88) | 254.80 | 3456.46 (2585.67, 4470.58) | 3486.94 (2618.04, 4533.94) | 3506.42 (2580.15, 4578.90) | 3508.06 (2614.47, 4498.59) | 0.07 (0.06, 0.09) | 0.09 (0.08, 0.11) | 0.05 (-0.02, 0.13) | 0.08 (-0.01, 0.18) |
| Albania | 100.30 (74.59, 133.49) | 98.79 (74.39, 126.46) | -1.51 | 3332.79 (2476.20, 4352.47) | 3326.95 (2465.49, 4350.58) | 3301.80 (2457.89, 4308.17) | 3318.85 (2438.37, 4349.74) | -0.05 (-0.07, -0.04) | -0.01 (-0.02, 0.00) | -0.08 (-0.16, 0.01) | 0.02 (-0.07, 0.12) |
| Algeria | 645.39 (479.34, 866.59) | 1575.44 (1145.18, 2068.86) | 144.11 | 3537.95 (2644.24, 4625.49) | 3564.53 (2673.08, 4608.98) | 3530.84 (2628.00, 4539.87) | 3490.42 (2574.41, 4501.08) | -0.05 (-0.07, -0.04) | 0.08 (0.07, 0.09) | -0.10 (-0.17, -0.03) | -0.07 (-0.15, 0.02) |
| American Samoa | 2.75 (2.06, 3.69) | 3.54 (2.64, 4.54) | 28.46 | 6655.53 (5003.81, 8662.95) | 6771.04 (5063.50, 8804.58) | 6937.28 (5161.02, 9106.84) | 6834.30 (5067.61, 8876.51) | 0.07 (0.04, 0.10) | 0.16 (0.08, 0.23) | 0.25 (0.13, 0.38) | -0.21 (-0.35, -0.08) |
| Andorra | 1.60 (1.19, 2.13) | 2.52 (1.86, 3.26) | 57.28 | 2405.21 (1794.72, 3124.23) | 2381.26 (1778.47, 3101.06) | 2380.43 (1768.20, 3081.33) | 2356.15 (1743.06, 3083.15) | -0.11 (-0.12, -0.09) | -0.14 (-0.23, -0.04) | 0.04 (-0.01, 0.09) | -0.07 (-0.14, 0.00) |
| Angola | 421.27 (311.33, 569.26) | 1189.73 (881.16, 1594.82) | 182.42 | 5612.05 (4167.57, 7340.38) | 5443.08 (4026.45, 7167.98) | 5441.72 (4049.10, 7100.66) | 5422.23 (4006.28, 7060.75) | -0.11 (-0.15, -0.07) | -0.33 (-0.49, -0.17) | 0.01 (-0.05, 0.06) | 0.01 (-0.10, 0.13) |
| Antigua and Barbuda | 2.85 (2.13, 3.79) | 5.42 (3.98, 7.01) | 89.88 | 5049.33 (3769.93, 6533.48) | 5055.77 (3796.41, 6603.78) | 5094.51 (3766.75, 6686.74) | 5213.91 (3815.14, 6777.31) | 0.09 (0.06, 0.12) | 0.01 (-0.02, 0.03) | 0.08 (0.07, 0.09) | 0.37 (0.20, 0.55) |
| Argentina | 942.32 (699.40, 1217.33) | 1427.57 (1060.86, 1858.26) | 51.50 | 2972.91 (2207.68, 3842.30) | 2914.82 (2186.85, 3810.99) | 2940.06 (2170.74, 3844.27) | 2953.76 (2193.16, 3856.79) | -0.01 (-0.04, 0.01) | -0.21 (-0.25, -0.16) | 0.10 (0.08, 0.11) | 0.08 (0.04, 0.11) |
| Armenia | 130.23 (97.24, 171.72) | 136.87 (101.87, 177.08) | 5.10 | 3900.63 (2913.66, 5064.43) | 3993.70 (2980.04, 5210.92) | 4005.81 (2986.36, 5235.10) | 3971.86 (2952.31, 5200.95) | 0.01 (-0.02, 0.05) | 0.25 (0.22, 0.27) | 0.03 (-0.11, 0.17) | -0.13 (-0.36, 0.09) |
| Australia | 513.28 (381.44, 670.78) | 763.48 (576.91, 971.74) | 48.74 | 2774.96 (2067.93, 3636.85) | 2776.90 (2060.18, 3649.00) | 2776.66 (2087.02, 3589.55) | 2772.66 (2061.69, 3605.91) | -0.01 (-0.02, 0.00) | 0.01 (-0.01, 0.03) | 0.00 (-0.08, 0.08) | -0.03 (-0.10, 0.04) |
| Austria | 202.32 (151.11, 264.21) | 242.81 (180.81, 310.67) | 20.01 | 2306.38 (1704.36, 3055.37) | 2318.59 (1730.48, 3015.37) | 2337.08 (1737.44, 3055.84) | 2337.01 (1724.02, 3072.33) | 0.01 (-0.01, 0.03) | 0.03 (-0.07, 0.13) | 0.09 (0.04, 0.14) | 0.01 (-0.06, 0.07) |
| Azerbaijan | 262.00 (192.81, 349.26) | 478.62 (345.65, 631.31) | 82.68 | 3961.26 (2911.98, 5220.59) | 4005.86 (2988.02, 5261.10) | 4023.73 (3000.54, 5245.06) | 3982.95 (2890.71, 5196.84) | -0.03 (-0.06, 0.00) | 0.11 (0.09, 0.13) | 0.05 (-0.08, 0.17) | -0.16 (-0.39, 0.07) |
| Bahamas | 12.39 (9.15, 16.79) | 22.60 (16.30, 29.35) | 82.43 | 5090.14 (3801.91, 6660.44) | 5088.66 (3748.62, 6628.53) | 5135.51 (3803.92, 6706.62) | 5219.39 (3794.96, 6817.03) | 0.08 (0.05, 0.11) | -0.01 (-0.03, 0.01) | 0.10 (0.06, 0.13) | 0.30 (0.12, 0.47) |
| Bahrain | 19.47 (13.96, 27.29) | 81.41 (57.09, 108.49) | 318.03 | 3754.51 (2786.85, 4888.10) | 3761.43 (2824.04, 4874.12) | 3813.51 (2828.08, 4904.77) | 3802.48 (2814.07, 4901.66) | 0.09 (0.07, 0.11) | 0.02 (-0.04, 0.09) | 0.14 (0.06, 0.23) | -0.02 (-0.06, 0.01) |
| Bangladesh | 2269.03 (1690.28, 3062.00) | 4489.30 (3347.09, 5899.26) | 97.85 | 2854.92 (2123.78, 3704.91) | 2871.88 (2139.91, 3748.14) | 2803.58 (2085.65, 3663.58) | 2772.76 (2067.74, 3588.61) | -0.13 (-0.16, -0.11) | 0.06 (0.04, 0.08) | -0.25 (-0.27, -0.22) | -0.06 (-0.14, 0.02) |
| Barbados | 13.16 (9.74, 17.46) | 17.73 (13.28, 22.55) | 34.73 | 5093.56 (3753.47, 6647.55) | 5105.03 (3788.77, 6643.13) | 5136.50 (3842.46, 6642.33) | 5234.37 (3871.81, 6751.24) | 0.07 (0.04, 0.10) | 0.01 (-0.04, 0.06) | 0.07 (0.06, 0.07) | 0.32 (0.14, 0.50) |
| Belarus | 328.82 (245.66, 423.41) | 331.95 (246.57, 424.54) | 0.95 | 2910.60 (2157.40, 3795.59) | 2891.30 (2145.84, 3778.04) | 2929.58 (2164.47, 3830.89) | 2954.74 (2163.71, 3850.80) | 0.05 (0.02, 0.08) | -0.08 (-0.13, -0.03) | 0.14 (0.05, 0.23) | 0.08 (0.06, 0.10) |
| Belgium | 241.75 (180.95, 313.08) | 273.72 (207.79, 346.24) | 13.22 | 2146.97 (1600.31, 2808.70) | 2052.31 (1527.71, 2663.10) | 1981.52 (1476.95, 2572.16) | 2147.46 (1602.47, 2772.13) | -0.06 (-0.22, 0.09) | -0.43 (-0.59, -0.27) | -0.34 (-0.57, -0.11) | 0.97 (0.62, 1.32) |
| Belize | 6.71 (4.94, 9.08) | 20.75 (15.26, 27.35) | 209.07 | 5075.49 (3761.60, 6669.10) | 5099.83 (3821.63, 6697.07) | 5135.63 (3823.08, 6684.49) | 5196.53 (3848.77, 6730.78) | 0.07 (0.04, 0.09) | 0.04 (0.00, 0.08) | 0.07 (0.07, 0.08) | 0.26 (0.05, 0.47) |
| Benin | 193.28 (142.11, 263.58) | 564.07 (416.63, 761.72) | 191.84 | 5989.41 (4400.41, 7857.68) | 5375.29 (4087.62, 6888.91) | 5510.85 (4025.14, 7159.35) | 6115.22 (4514.47, 7974.74) | 0.21 (0.02, 0.39) | -1.06 (-1.22, -0.89) | 0.24 (0.20, 0.28) | 1.33 (1.05, 1.61) |
| Bermuda | 3.63 (2.68, 4.87) | 3.98 (3.00, 5.08) | 9.45 | 5093.52 (3802.20, 6725.55) | 5117.10 (3786.14, 6676.74) | 5151.35 (3834.79, 6681.19) | 5255.61 (3892.20, 6828.38) | 0.10 (0.08, 0.12) | 0.05 (0.04, 0.06) | 0.07 (0.05, 0.08) | 0.30 (0.18, 0.41) |
| Bhutan | 12.99 (9.56, 17.67) | 22.33 (16.34, 29.93) | 71.88 | 2809.63 (2079.53, 3691.03) | 2818.47 (2111.41, 3650.58) | 2774.78 (2079.06, 3610.99) | 2785.96 (2039.64, 3660.55) | -0.05 (-0.07, -0.04) | 0.03 (0.01, 0.06) | -0.17 (-0.20, -0.14) | 0.07 (0.04, 0.10) |
| Bolivia (Plurinational State of) | 221.86 (162.55, 296.30) | 509.65 (375.04, 671.97) | 129.72 | 4499.62 (3320.57, 5891.84) | 4441.37 (3282.89, 5844.70) | 4404.09 (3242.54, 5732.14) | 4493.91 (3325.66, 5819.01) | -0.01 (-0.05, 0.03) | -0.11 (-0.18, -0.04) | -0.09 (-0.17, -0.01) | 0.32 (0.15, 0.49) |
| Bosnia and Herzegovina | 158.53 (117.50, 210.28) | 127.49 (95.63, 162.58) | -19.58 | 3228.42 (2394.33, 4269.28) | 3248.30 (2402.97, 4259.82) | 3281.62 (2438.14, 4313.89) | 3299.35 (2441.41, 4310.60) | 0.05 (0.02, 0.07) | 0.06 (0.04, 0.07) | 0.11 (-0.04, 0.25) | 0.03 (-0.10, 0.16) |
| Botswana | 71.14 (54.49, 93.53) | 172.06 (125.84, 230.46) | 141.86 | 7368.98 (5632.70, 9484.99) | 6629.70 (5054.14, 8512.47) | 6890.83 (5156.55, 8875.11) | 6980.62 (5197.79, 9109.28) | -0.30 (-0.46, -0.13) | -1.20 (-1.97, -0.42) | 0.43 (0.38, 0.48) | 0.23 (0.11, 0.35) |
| Brazil | 7641.88 (5542.49, 10236.47) | 14500.39 (10433.56, 18998.74) | 89.75 | 5756.61 (4151.82, 7560.72) | 5732.05 (4149.85, 7476.13) | 5734.48 (4215.62, 7473.02) | 5902.62 (4262.89, 7727.57) | 0.02 (-0.03, 0.06) | -0.06 (-0.13, 0.02) | 0.01 (0.00, 0.02) | 0.42 (0.15, 0.68) |
| Brunei Darussalam | 7.60 (5.49, 10.58) | 16.31 (11.82, 21.79) | 114.56 | 3076.81 (2297.92, 4016.29) | 2997.72 (2234.07, 3898.36) | 3031.44 (2270.23, 3991.15) | 3082.88 (2281.18, 4044.51) | 0.04 (0.00, 0.07) | -0.26 (-0.27, -0.25) | 0.12 (0.04, 0.19) | 0.22 (0.17, 0.27) |
| Bulgaria | 314.41 (234.36, 406.88) | 270.14 (201.79, 344.55) | -14.08 | 3253.26 (2387.23, 4251.51) | 3260.78 (2439.90, 4268.54) | 3289.68 (2418.60, 4328.68) | 3309.39 (2443.80, 4300.90) | 0.05 (0.03, 0.07) | 0.03 (0.00, 0.06) | 0.09 (-0.02, 0.20) | 0.03 (-0.06, 0.12) |
| Burkina Faso | 584.29 (454.59, 755.08) | 1356.70 (1005.63, 1820.63) | 132.20 | 9050.09 (6995.19, 11464.25) | 8056.94 (6031.66, 10464.40) | 8208.34 (6176.12, 10709.53) | 8085.38 (5987.85, 10490.24) | -0.51 (-0.66, -0.36) | -1.30 (-2.11, -0.48) | 0.20 (0.08, 0.33) | -0.12 (-0.20, -0.04) |
| Burundi | 373.56 (279.24, 500.19) | 850.20 (632.98, 1135.90) | 127.59 | 9579.86 (7152.21, 12349.32) | 9432.37 (7067.13, 12371.45) | 9498.17 (7046.33, 12236.95) | 9706.68 (7251.07, 12536.54) | 0.02 (-0.03, 0.08) | -0.20 (-0.39, 0.00) | 0.07 (0.06, 0.08) | 0.35 (0.22, 0.49) |
| Cabo Verde | 16.54 (12.51, 22.08) | 41.03 (30.35, 54.94) | 148.14 | 7018.53 (5240.58, 9185.83) | 6816.08 (5163.64, 8888.34) | 6999.14 (5207.07, 9124.78) | 7038.07 (5235.75, 9197.60) | -0.02 (-0.08, 0.03) | -0.35 (-0.61, -0.08) | 0.29 (0.27, 0.32) | 0.11 (-0.01, 0.22) |
| Cambodia | 317.19 (234.58, 429.40) | 707.63 (521.66, 946.93) | 123.09 | 4251.17 (3160.71, 5583.42) | 4294.14 (3191.59, 5661.28) | 4266.22 (3151.30, 5649.83) | 4283.42 (3170.51, 5653.34) | -0.03 (-0.05, -0.01) | 0.11 (0.10, 0.11) | -0.06 (-0.19, 0.06) | -0.03 (-0.17, 0.11) |
| Cameroon | 578.31 (437.47, 766.63) | 1810.78 (1355.03, 2440.15) | 213.12 | 7772.44 (5888.42, 10034.99) | 7777.23 (5895.70, 9979.60) | 7730.24 (5846.74, 10086.51) | 7714.20 (5815.66, 10087.85) | -0.15 (-0.20, -0.09) | -0.08 (-0.45, 0.29) | -0.05 (-0.12, 0.01) | 0.01 (-0.05, 0.08) |
| Canada | 1232.27 (909.58, 1625.96) | 1596.77 (1190.17, 2039.65) | 29.58 | 3945.17 (2928.07, 5169.17) | 3904.32 (2891.72, 5080.28) | 3892.03 (2873.61, 5103.04) | 3927.07 (2870.06, 5151.52) | -0.02 (-0.03, 0.00) | -0.11 (-0.11, -0.10) | -0.03 (-0.11, 0.04) | 0.11 (0.09, 0.12) |
| Central African Republic | 111.86 (83.31, 150.85) | 229.01 (169.36, 304.66) | 104.73 | 5516.34 (4110.39, 7154.11) | 5378.97 (3980.93, 6998.81) | 5518.97 (4114.71, 7197.63) | 5515.93 (4078.05, 7196.03) | 0.06 (0.02, 0.09) | -0.28 (-0.34, -0.21) | 0.27 (0.17, 0.38) | 0.03 (-0.04, 0.09) |
| Chad | 287.24 (217.01, 384.08) | 748.67 (557.59, 993.60) | 160.64 | 7061.98 (5309.64, 9174.91) | 6917.27 (5195.68, 8928.03) | 7148.17 (5336.36, 9363.09) | 7125.21 (5287.21, 9188.28) | 0.01 (-0.04, 0.06) | -0.27 (-0.55, 0.01) | 0.36 (0.26, 0.45) | 0.02 (-0.10, 0.13) |
| Chile | 383.29 (283.76, 514.56) | 615.34 (459.74, 793.79) | 60.54 | 2973.04 (2188.55, 3913.33) | 2942.97 (2200.21, 3827.49) | 2956.33 (2198.46, 3868.38) | 2988.22 (2218.06, 3877.20) | 0.01 (-0.01, 0.02) | -0.11 (-0.13, -0.09) | 0.05 (0.03, 0.06) | 0.10 (0.06, 0.14) |
| China | 52823.55 (38590.82, 70575.33) | 78716.45 (57322.86, 102075.45) | 49.02 | 4490.53 (3272.11, 5899.95) | 4346.05 (3153.82, 5698.15) | 4359.23 (3147.19, 5712.93) | 4413.14 (3197.98, 5778.01) | -0.18 (-0.26, -0.10) | -0.33 (-0.43, -0.23) | 0.04 (-0.13, 0.22) | 0.23 (-0.47, 0.93) |
| Colombia | 1714.26 (1269.24, 2305.41) | 3207.19 (2343.29, 4165.29) | 87.09 | 6003.15 (4452.84, 7868.99) | 5977.82 (4473.73, 7776.21) | 6070.22 (4507.48, 7848.59) | 6267.84 (4576.18, 8174.83) | 0.11 (0.07, 0.16) | -0.07 (-0.20, 0.06) | 0.16 (0.08, 0.24) | 0.52 (0.30, 0.74) |
| Comoros | 30.93 (23.31, 40.76) | 64.49 (47.64, 85.43) | 108.48 | 9609.68 (7268.84, 12430.07) | 9293.51 (6979.73, 12030.42) | 9344.74 (7028.20, 12105.77) | 9575.99 (7079.07, 12492.55) | -0.02 (-0.09, 0.05) | -0.37 (-0.53, -0.20) | 0.06 (0.02, 0.10) | 0.39 (0.25, 0.52) |
| Congo | 93.74 (69.86, 125.80) | 263.48 (194.43, 350.14) | 181.07 | 5436.32 (4065.96, 7090.64) | 5372.73 (4018.80, 6965.11) | 5480.58 (4080.47, 7155.33) | 5518.77 (4104.67, 7166.99) | 0.05 (0.01, 0.08) | -0.15 (-0.30, 0.00) | 0.21 (0.18, 0.25) | 0.09 (0.06, 0.13) |
| Cook Islands | 1.13 (0.82, 1.49) | 1.28 (0.94, 1.64) | 13.53 | 6738.91 (4899.62, 8794.60) | 6890.61 (5089.74, 8934.11) | 7166.13 (5275.10, 9430.96) | 7020.62 (5143.39, 9195.30) | 0.15 (0.10, 0.19) | 0.20 (0.06, 0.33) | 0.41 (0.20, 0.61) | -0.25 (-0.30, -0.20) |
| Costa Rica | 158.64 (116.29, 214.02) | 328.66 (244.29, 426.51) | 107.17 | 6015.01 (4441.55, 7806.36) | 5968.25 (4513.96, 7750.12) | 6113.67 (4536.10, 7964.63) | 6295.55 (4677.57, 8170.15) | 0.13 (0.09, 0.17) | -0.10 (-0.19, -0.01) | 0.25 (0.18, 0.32) | 0.45 (0.20, 0.70) |
| Croatia | 181.88 (135.05, 233.82) | 162.32 (120.89, 207.02) | -10.76 | 3259.84 (2410.11, 4249.08) | 3258.36 (2426.99, 4241.78) | 3298.84 (2443.65, 4295.92) | 3328.88 (2436.85, 4412.44) | 0.05 (0.02, 0.08) | -0.01 (-0.06, 0.03) | 0.13 (0.00, 0.26) | 0.09 (0.00, 0.17) |
| Cuba | 567.29 (420.88, 751.94) | 695.88 (518.58, 889.64) | 22.67 | 5067.43 (3743.98, 6682.31) | 5087.12 (3742.54, 6634.51) | 5134.85 (3849.16, 6674.36) | 5256.17 (3880.92, 6931.91) | 0.10 (0.07, 0.13) | 0.02 (-0.02, 0.07) | 0.10 (0.07, 0.13) | 0.36 (0.19, 0.54) |
| Cyprus | 19.01 (14.16, 25.15) | 37.63 (27.91, 49.12) | 97.98 | 2311.78 (1719.63, 3053.68) | 2307.89 (1732.54, 3006.82) | 2325.59 (1728.72, 3041.44) | 2307.69 (1714.54, 3046.88) | -0.03 (-0.04, -0.01) | -0.03 (-0.11, 0.04) | 0.09 (0.03, 0.14) | -0.12 (-0.18, -0.07) |
| Czechia | 373.22 (274.31, 486.30) | 420.34 (312.25, 541.14) | 12.62 | 3272.67 (2399.15, 4286.76) | 3278.85 (2428.61, 4279.36) | 3302.08 (2468.63, 4315.09) | 3338.12 (2468.66, 4432.63) | 0.06 (0.05, 0.08) | 0.03 (-0.03, 0.10) | 0.07 (-0.03, 0.17) | 0.06 (-0.02, 0.14) |
| Côte d'Ivoire | 586.14 (436.22, 796.62) | 1477.98 (1084.59, 2001.59) | 152.16 | 6655.49 (4967.96, 8680.95) | 6324.69 (4786.63, 8093.38) | 6622.89 (4921.73, 8682.07) | 6715.93 (4994.21, 8782.95) | 0.08 (-0.01, 0.16) | -0.55 (-0.69, -0.41) | 0.50 (0.43, 0.57) | 0.20 (0.11, 0.30) |
| Democratic People's Republic of Korea | 834.58 (616.60, 1103.96) | 1277.51 (946.32, 1658.74) | 53.07 | 4045.59 (2968.12, 5317.52) | 4037.08 (3006.22, 5288.25) | 4073.04 (3010.21, 5352.97) | 4115.95 (3039.79, 5411.03) | 0.07 (0.05, 0.08) | -0.02 (-0.05, 0.01) | 0.09 (0.02, 0.16) | 0.10 (0.06, 0.13) |
| Democratic Republic of the Congo | 1464.37 (1083.67, 1964.72) | 3696.61 (2731.17, 4956.64) | 152.44 | 5486.31 (4105.13, 7154.35) | 5391.51 (4027.90, 7010.68) | 5517.25 (4083.69, 7239.84) | 5525.30 (4080.70, 7196.78) | 0.02 (-0.02, 0.06) | -0.22 (-0.43, -0.01) | 0.25 (0.21, 0.29) | 0.04 (-0.02, 0.09) |
| Denmark | 137.25 (103.07, 178.54) | 148.64 (111.45, 188.41) | 8.30 | 2331.68 (1747.94, 3070.44) | 2325.86 (1746.34, 3029.53) | 2341.09 (1749.18, 3056.25) | 2330.13 (1714.10, 3063.31) | -0.01 (-0.02, 0.01) | -0.04 (-0.10, 0.03) | 0.07 (0.05, 0.10) | -0.06 (-0.08, -0.04) |
| Djibouti | 34.28 (25.44, 45.46) | 115.32 (85.64, 151.57) | 236.46 | 9920.69 (7428.26, 12751.42) | 9700.92 (7288.02, 12578.96) | 9720.60 (7297.59, 12559.06) | 9783.87 (7377.60, 12681.57) | -0.06 (-0.11, -0.01) | -0.26 (-0.44, -0.08) | 0.02 (-0.01, 0.05) | 0.15 (0.03, 0.27) |
| Dominica | 3.14 (2.36, 4.18) | 3.83 (2.85, 4.92) | 22.24 | 5077.49 (3755.47, 6666.31) | 5085.20 (3791.62, 6566.85) | 5132.19 (3823.25, 6752.50) | 5207.47 (3839.06, 6767.66) | 0.07 (0.04, 0.10) | 0.00 (-0.04, 0.05) | 0.10 (0.07, 0.12) | 0.30 (0.06, 0.54) |
| Dominican Republic | 295.64 (218.82, 394.84) | 580.84 (423.04, 765.55) | 96.47 | 5066.08 (3784.39, 6588.35) | 5077.37 (3769.01, 6617.22) | 5119.17 (3782.65, 6674.79) | 5234.21 (3834.92, 6834.52) | 0.08 (0.05, 0.11) | 0.01 (-0.02, 0.05) | 0.09 (0.07, 0.10) | 0.36 (0.14, 0.59) |
| Ecuador | 362.48 (269.08, 484.41) | 780.06 (576.31, 1030.66) | 115.20 | 4487.83 (3355.54, 5811.05) | 4424.16 (3287.21, 5761.92) | 4421.64 (3286.85, 5762.46) | 4484.49 (3304.27, 5863.91) | 0.00 (-0.02, 0.03) | -0.13 (-0.17, -0.10) | -0.01 (-0.01, 0.00) | 0.22 (0.11, 0.34) |
| Egypt | 1796.60 (1340.69, 2359.39) | 3628.70 (2657.58, 4824.15) | 101.98 | 3981.86 (2984.03, 5126.93) | 4246.77 (3203.06, 5484.68) | 3943.73 (2958.87, 5078.83) | 3856.06 (2837.41, 5031.48) | -0.29 (-0.38, -0.19) | 0.57 (0.30, 0.85) | -0.78 (-0.92, -0.63) | -0.14 (-0.28, 0.01) |
| El Salvador | 244.74 (184.74, 323.06) | 389.93 (293.92, 504.01) | 59.32 | 6031.18 (4478.60, 7811.00) | 5972.61 (4472.44, 7710.91) | 6114.87 (4580.38, 8056.49) | 6320.60 (4734.87, 8163.55) | 0.13 (0.08, 0.19) | -0.13 (-0.27, 0.01) | 0.25 (0.16, 0.34) | 0.58 (0.25, 0.90) |
| Equatorial Guinea | 16.22 (12.09, 21.60) | 60.17 (44.02, 82.85) | 270.97 | 5459.60 (4046.55, 7154.38) | 5215.64 (3908.27, 6739.57) | 5329.06 (3941.36, 7033.50) | 5422.52 (4070.85, 7112.17) | -0.02 (-0.09, 0.05) | -0.50 (-0.70, -0.30) | 0.23 (0.21, 0.24) | 0.21 (0.16, 0.26) |
| Eritrea | 203.42 (151.48, 272.28) | 529.34 (393.03, 707.32) | 160.22 | 9605.58 (7206.22, 12450.82) | 9266.67 (6952.09, 12022.92) | 9322.23 (6929.42, 12117.54) | 9518.31 (7046.16, 12463.99) | -0.05 (-0.12, 0.02) | -0.40 (-0.60, -0.20) | 0.06 (0.05, 0.08) | 0.36 (0.22, 0.50) |
| Estonia | 49.96 (37.14, 64.58) | 44.63 (33.85, 57.37) | -10.66 | 2908.86 (2153.77, 3815.99) | 2913.19 (2163.61, 3799.56) | 2944.98 (2169.25, 3882.64) | 2980.54 (2228.12, 3937.74) | 0.09 (0.07, 0.11) | 0.00 (-0.01, 0.02) | 0.11 (0.05, 0.17) | 0.10 (0.05, 0.15) |
| Eswatini | 41.54 (31.21, 55.79) | 75.95 (56.64, 101.77) | 82.85 | 7472.19 (5550.98, 9704.84) | 7071.20 (5316.79, 9018.13) | 7421.99 (5650.08, 9647.95) | 7365.49 (5480.66, 9504.27) | -0.04 (-0.12, 0.03) | -0.60 (-0.91, -0.30) | 0.52 (0.41, 0.64) | -0.06 (-0.20, 0.08) |
| Ethiopia | 3679.45 (2718.52, 4876.49) | 8362.50 (6087.84, 11186.36) | 127.28 | 10517.51 (7756.58, 13681.72) | 10375.15 (7623.86, 13444.93) | 10257.53 (7494.15, 13291.23) | 10400.59 (7544.13, 13495.23) | -0.09 (-0.13, -0.04) | -0.16 (-0.33, 0.00) | -0.12 (-0.15, -0.08) | 0.23 (0.14, 0.32) |
| Fiji | 41.22 (31.14, 54.74) | 58.74 (42.82, 78.18) | 42.50 | 6004.96 (4558.48, 7724.36) | 6134.46 (4569.42, 8078.75) | 6252.99 (4649.48, 8176.75) | 6255.32 (4582.17, 8276.88) | 0.09 (0.06, 0.12) | 0.18 (0.02, 0.33) | 0.20 (0.09, 0.31) | -0.03 (-0.09, 0.04) |
| Finland | 135.95 (100.26, 177.78) | 142.16 (108.39, 179.91) | 4.57 | 2322.58 (1715.99, 3053.80) | 2325.71 (1727.45, 3050.66) | 2338.60 (1754.02, 3034.65) | 2348.08 (1732.40, 3067.34) | 0.03 (0.02, 0.04) | 0.01 (-0.03, 0.04) | 0.06 (0.05, 0.07) | 0.04 (0.02, 0.07) |
| France | 1559.52 (1174.28, 2020.16) | 1728.90 (1315.81, 2196.01) | 10.86 | 2464.26 (1849.02, 3211.87) | 2434.43 (1824.22, 3183.62) | 2447.67 (1818.51, 3201.71) | 2428.17 (1822.11, 3175.69) | -0.04 (-0.06, -0.03) | -0.13 (-0.15, -0.10) | 0.06 (0.02, 0.11) | -0.10 (-0.13, -0.07) |
| Gabon | 41.34 (30.54, 56.31) | 89.88 (67.08, 118.98) | 117.43 | 5578.02 (4087.60, 7384.95) | 5458.98 (4092.76, 7052.04) | 5471.73 (4043.40, 7133.49) | 5494.35 (4118.13, 7136.08) | -0.07 (-0.11, -0.04) | -0.24 (-0.40, -0.09) | 0.03 (0.00, 0.06) | 0.06 (0.02, 0.10) |
| Gambia | 49.33 (36.51, 67.32) | 124.67 (92.14, 168.21) | 152.71 | 7077.78 (5295.45, 9307.71) | 6854.11 (5115.28, 8886.29) | 7059.74 (5259.32, 9262.16) | 7042.79 (5192.46, 9162.21) | -0.03 (-0.09, 0.03) | -0.37 (-0.62, -0.12) | 0.33 (0.30, 0.35) | 0.06 (-0.11, 0.23) |
| Georgia | 225.41 (168.85, 294.01) | 160.58 (120.16, 205.13) | -28.76 | 3923.50 (2926.81, 5135.93) | 3980.90 (2971.35, 5250.13) | 4001.99 (2984.07, 5251.53) | 3967.46 (2940.37, 5166.82) | 0.00 (-0.03, 0.02) | 0.15 (0.14, 0.16) | 0.06 (-0.06, 0.17) | -0.15 (-0.35, 0.05) |
| Germany | 2167.17 (1621.13, 2791.31) | 2261.40 (1728.84, 2861.63) | 4.35 | 2322.96 (1722.94, 3043.96) | 2331.63 (1723.24, 3069.56) | 2342.77 (1762.02, 3049.39) | 2338.55 (1744.71, 3074.21) | 0.01 (0.00, 0.01) | 0.02 (-0.03, 0.08) | 0.06 (0.05, 0.07) | -0.03 (-0.05, 0.00) |
| Ghana | 787.69 (588.73, 1066.16) | 2002.49 (1491.51, 2663.49) | 154.22 | 7028.18 (5242.53, 9176.39) | 6849.32 (5134.88, 8975.75) | 7004.30 (5187.65, 9188.19) | 7018.50 (5213.55, 9161.32) | -0.03 (-0.08, 0.02) | -0.30 (-0.54, -0.06) | 0.25 (0.20, 0.29) | 0.11 (-0.03, 0.26) |
| Greece | 292.70 (222.02, 375.80) | 306.81 (230.06, 389.14) | 4.82 | 2577.32 (1918.12, 3398.12) | 2596.66 (1939.81, 3410.71) | 2844.28 (2159.00, 3660.32) | 2568.58 (1885.34, 3353.49) | 0.09 (-0.08, 0.26) | 0.05 (-0.04, 0.14) | 0.95 (0.58, 1.32) | -1.24 (-1.63, -0.84) |
| Greenland | 2.42 (1.77, 3.25) | 2.42 (1.80, 3.11) | -0.21 | 3833.97 (2839.80, 5020.78) | 3802.01 (2811.81, 4945.74) | 3806.87 (2837.29, 4908.95) | 3838.17 (2817.88, 4992.50) | 0.03 (0.01, 0.04) | -0.08 (-0.11, -0.06) | 0.02 (-0.02, 0.05) | 0.10 (0.03, 0.18) |
| Grenada | 3.41 (2.54, 4.55) | 5.85 (4.36, 7.58) | 71.67 | 5060.68 (3766.01, 6604.28) | 5077.20 (3802.34, 6637.07) | 5117.14 (3816.43, 6677.38) | 5207.69 (3838.02, 6808.09) | 0.09 (0.08, 0.11) | 0.03 (0.02, 0.04) | 0.08 (0.05, 0.11) | 0.28 (0.16, 0.40) |
| Guam | 9.07 (6.75, 12.25) | 11.49 (8.58, 14.99) | 26.70 | 6503.38 (4878.88, 8549.99) | 6737.18 (5039.24, 8779.22) | 6890.07 (5119.70, 8958.72) | 6747.83 (5003.07, 8904.49) | 0.11 (0.07, 0.15) | 0.35 (0.29, 0.41) | 0.23 (0.09, 0.36) | -0.27 (-0.37, -0.17) |
| Guatemala | 344.49 (256.45, 461.11) | 1024.24 (752.11, 1383.93) | 197.32 | 6012.08 (4468.76, 7873.57) | 5964.06 (4493.23, 7720.99) | 6206.85 (4758.52, 7863.71) | 6298.45 (4623.63, 8301.34) | 0.15 (0.11, 0.20) | -0.10 (-0.20, 0.00) | 0.43 (0.28, 0.58) | 0.28 (-0.03, 0.59) |
| Guinea | 319.76 (238.41, 430.75) | 658.73 (488.98, 874.68) | 106.00 | 7063.60 (5268.28, 9278.75) | 6839.21 (5170.32, 8871.45) | 7051.58 (5292.06, 9200.14) | 7090.42 (5267.48, 9182.45) | 0.01 (-0.04, 0.06) | -0.37 (-0.59, -0.15) | 0.33 (0.21, 0.45) | 0.12 (0.02, 0.21) |
| Guinea-Bissau | 49.33 (36.69, 66.66) | 106.90 (79.26, 145.06) | 116.68 | 7008.39 (5227.75, 9099.50) | 6796.24 (5111.69, 8954.59) | 6986.90 (5231.84, 9072.91) | 7022.08 (5198.44, 9115.10) | -0.02 (-0.07, 0.03) | -0.35 (-0.58, -0.12) | 0.30 (0.22, 0.38) | 0.11 (-0.04, 0.26) |
| Guyana | 32.86 (24.22, 44.62) | 40.33 (30.03, 52.59) | 22.73 | 5086.67 (3758.03, 6681.37) | 5056.65 (3797.11, 6573.55) | 5104.40 (3772.31, 6679.08) | 5193.70 (3823.74, 6765.50) | 0.07 (0.04, 0.10) | -0.06 (-0.08, -0.03) | 0.10 (0.09, 0.11) | 0.29 (0.10, 0.47) |
| Haiti | 251.15 (186.33, 332.51) | 602.31 (438.94, 810.98) | 139.82 | 5066.61 (3763.67, 6565.72) | 5053.29 (3778.47, 6601.46) | 5073.91 (3766.94, 6642.82) | 5182.88 (3794.76, 6789.19) | 0.05 (0.02, 0.08) | -0.04 (-0.08, 0.01) | 0.04 (0.03, 0.06) | 0.37 (0.15, 0.58) |
| Honduras | 195.51 (147.58, 262.46) | 553.69 (412.56, 739.36) | 183.20 | 5987.80 (4493.37, 7798.62) | 5897.95 (4401.38, 7591.99) | 6033.24 (4463.25, 7812.40) | 6252.10 (4628.20, 8176.44) | 0.11 (0.06, 0.17) | -0.18 (-0.32, -0.04) | 0.24 (0.14, 0.35) | 0.55 (0.29, 0.81) |
| Hungary | 377.47 (279.97, 488.16) | 378.81 (282.16, 486.27) | 0.36 | 3251.46 (2409.29, 4245.73) | 3246.20 (2399.25, 4212.13) | 3278.27 (2435.34, 4282.48) | 3314.50 (2429.43, 4366.32) | 0.05 (0.04, 0.07) | -0.01 (-0.03, 0.01) | 0.10 (0.06, 0.14) | 0.08 (-0.02, 0.18) |
| Iceland | 6.17 (4.60, 8.17) | 8.93 (6.76, 11.29) | 44.86 | 2334.35 (1737.56, 3059.46) | 2330.11 (1733.04, 3060.75) | 2371.24 (1769.24, 3130.71) | 2344.86 (1741.71, 3030.81) | 0.02 (0.00, 0.05) | -0.03 (-0.08, 0.01) | 0.24 (0.17, 0.32) | -0.13 (-0.18, -0.08) |
| India | 22294.91 (16231.37, 30037.98) | 43092.52 (31372.82, 57028.64) | 93.28 | 3056.56 (2225.51, 4022.57) | 3072.50 (2242.05, 4015.27) | 3017.30 (2210.13, 3960.23) | 3004.65 (2184.21, 3925.56) | -0.18 (-0.22, -0.13) | 0.06 (0.05, 0.07) | -0.18 (-0.23, -0.12) | -0.18 (-0.56, 0.20) |
| Indonesia | 7397.04 (5395.67, 9964.56) | 13385.73 (9523.33, 17639.24) | 80.96 | 4611.20 (3347.96, 6038.43) | 4592.81 (3310.43, 6034.44) | 4639.44 (3380.41, 6099.29) | 4625.51 (3344.52, 6048.62) | -0.01 (-0.03, 0.01) | -0.04 (-0.07, -0.02) | 0.11 (0.05, 0.17) | -0.10 (-0.23, 0.02) |
| Iran (Islamic Republic of) | 1605.95 (1185.98, 2128.40) | 3487.93 (2500.82, 4593.17) | 117.19 | 3909.46 (2867.25, 5057.73) | 3795.62 (2776.16, 4917.85) | 3708.03 (2718.63, 4802.30) | 3475.24 (2533.27, 4488.30) | -0.20 (-0.37, -0.03) | -0.13 (-0.94, 0.70) | -0.24 (-0.44, -0.04) | -0.74 (-1.14, -0.33) |
| Iraq | 550.06 (418.59, 726.13) | 1553.31 (1156.88, 2037.05) | 182.39 | 4468.44 (3393.22, 5696.19) | 5241.77 (3991.18, 6635.97) | 4766.44 (3556.26, 6020.76) | 3969.43 (2976.98, 5071.86) | -0.47 (-0.77, -0.17) | 1.72 (1.16, 2.27) | -0.98 (-1.43, -0.53) | -2.05 (-2.73, -1.36) |
| Ireland | 81.52 (60.71, 106.37) | 128.09 (95.00, 165.78) | 57.12 | 2308.01 (1703.22, 3020.16) | 2306.58 (1731.23, 3007.28) | 2339.44 (1725.71, 3070.56) | 2315.36 (1713.37, 3030.36) | 0.01 (-0.01, 0.03) | -0.03 (-0.10, 0.05) | 0.17 (0.13, 0.21) | -0.11 (-0.17, -0.05) |
| Israel | 106.37 (79.10, 139.93) | 213.63 (159.59, 277.79) | 100.85 | 2279.88 (1699.10, 2985.21) | 2293.13 (1690.69, 3008.45) | 2326.30 (1718.98, 3038.72) | 2317.90 (1718.33, 3040.70) | 0.05 (0.03, 0.06) | 0.05 (-0.01, 0.10) | 0.16 (0.11, 0.21) | -0.04 (-0.11, 0.03) |
| Italy | 1608.78 (1204.04, 2059.27) | 1780.80 (1320.78, 2246.92) | 10.69 | 2519.02 (1872.42, 3307.04) | 2530.67 (1868.75, 3304.64) | 2568.22 (1885.67, 3379.58) | 2559.58 (1891.93, 3341.82) | 0.03 (0.02, 0.05) | 0.03 (-0.03, 0.09) | 0.16 (0.12, 0.21) | -0.02 (-0.16, 0.11) |
| Jamaica | 98.30 (73.53, 130.24) | 158.84 (117.03, 208.87) | 61.58 | 5057.74 (3783.07, 6558.40) | 5065.51 (3760.31, 6614.91) | 5125.16 (3810.08, 6678.71) | 5232.92 (3812.09, 6881.80) | 0.08 (0.05, 0.11) | 0.00 (-0.07, 0.07) | 0.13 (0.12, 0.13) | 0.31 (0.13, 0.49) |
| Japan | 4771.61 (3488.86, 6157.44) | 4702.30 (3524.12, 6020.98) | -1.45 | 3286.01 (2378.01, 4313.52) | 3256.24 (2351.72, 4253.12) | 3310.64 (2411.46, 4347.90) | 3337.25 (2429.74, 4361.35) | 0.05 (0.03, 0.07) | -0.11 (-0.20, -0.02) | 0.17 (0.11, 0.24) | 0.11 (0.07, 0.14) |
| Jordan | 77.93 (57.83, 104.94) | 348.91 (257.04, 458.45) | 347.71 | 3060.29 (2291.04, 3958.73) | 3024.52 (2264.60, 3882.91) | 3102.81 (2306.79, 4018.23) | 3112.81 (2321.59, 4014.08) | 0.04 (-0.02, 0.10) | -0.15 (-0.33, 0.02) | 0.29 (0.07, 0.51) | 0.08 (-0.04, 0.21) |
| Kazakhstan | 611.66 (450.44, 809.26) | 789.20 (583.41, 1026.60) | 29.03 | 3872.92 (2866.35, 5016.15) | 3932.93 (2956.72, 5125.19) | 3996.54 (2963.42, 5235.01) | 3963.04 (2931.45, 5149.50) | 0.04 (0.01, 0.07) | 0.15 (0.11, 0.18) | 0.17 (0.05, 0.29) | -0.14 (-0.37, 0.09) |
| Kenya | 1419.45 (1046.09, 1905.85) | 4206.01 (3069.24, 5600.97) | 196.31 | 9546.90 (7000.53, 12381.05) | 10212.46 (7532.14, 13197.18) | 9566.52 (6921.29, 12418.40) | 10000.33 (7248.11, 13010.21) | -0.13 (-0.23, -0.03) | 0.63 (0.39, 0.87) | -0.68 (-0.98, -0.37) | 0.42 (0.28, 0.55) |
| Kiribati | 4.13 (3.07, 5.52) | 7.48 (5.60, 9.84) | 81.29 | 6567.29 (4900.18, 8533.61) | 6696.33 (5033.97, 8741.09) | 6809.62 (5050.32, 8853.30) | 6754.42 (5034.58, 8768.89) | 0.07 (0.04, 0.09) | 0.18 (0.11, 0.25) | 0.17 (0.05, 0.30) | -0.10 (-0.23, 0.04) |
| Kuwait | 63.48 (46.15, 87.48) | 202.79 (146.81, 272.53) | 219.45 | 3587.14 (2678.50, 4663.79) | 3578.06 (2725.43, 4494.70) | 3469.69 (2568.46, 4463.02) | 3324.26 (2475.37, 4262.57) | -0.25 (-0.28, -0.22) | -0.01 (-0.04, 0.02) | -0.31 (-0.42, -0.20) | -0.43 (-0.48, -0.37) |
| Kyrgyzstan | 143.96 (106.74, 193.61) | 254.52 (187.51, 341.61) | 76.79 | 3869.73 (2879.86, 5084.28) | 3928.52 (2945.12, 5114.26) | 3980.72 (2937.56, 5208.42) | 3953.26 (2909.40, 5207.17) | 0.03 (0.00, 0.06) | 0.15 (0.14, 0.16) | 0.14 (0.01, 0.26) | -0.13 (-0.37, 0.11) |
| Lao People's Democratic Republic | 133.33 (98.86, 177.84) | 302.69 (222.76, 403.92) | 127.03 | 4306.35 (3182.77, 5629.35) | 4337.58 (3229.36, 5756.08) | 4362.71 (3259.10, 5675.59) | 4335.21 (3208.98, 5629.59) | 0.01 (-0.01, 0.03) | 0.08 (0.05, 0.11) | 0.06 (-0.01, 0.14) | -0.14 (-0.27, -0.01) |
| Latvia | 85.25 (64.00, 109.39) | 64.51 (48.37, 81.84) | -24.32 | 2918.56 (2184.39, 3799.13) | 2891.35 (2142.59, 3766.35) | 2940.72 (2170.89, 3841.78) | 2965.05 (2198.62, 3864.91) | 0.07 (0.04, 0.09) | -0.10 (-0.13, -0.08) | 0.18 (0.12, 0.23) | 0.07 (0.04, 0.10) |
| Lebanon | 83.70 (62.64, 108.09) | 168.67 (124.17, 218.92) | 101.51 | 3076.04 (2282.92, 3956.42) | 2740.96 (2090.92, 3505.17) | 2521.61 (1983.68, 3132.51) | 3023.93 (2241.29, 3887.44) | -0.19 (-0.49, 0.11) | -1.15 (-1.56, -0.75) | -0.86 (-0.97, -0.75) | 2.29 (1.49, 3.10) |
| Lesotho | 100.02 (76.01, 132.30) | 144.78 (108.44, 192.97) | 44.75 | 7264.05 (5452.76, 9403.91) | 6971.12 (5284.55, 8956.07) | 7287.27 (5486.92, 9406.66) | 7174.40 (5383.02, 9267.18) | -0.02 (-0.08, 0.04) | -0.46 (-0.74, -0.19) | 0.49 (0.40, 0.57) | -0.10 (-0.24, 0.03) |
| Liberia | 101.44 (75.61, 134.05) | 290.54 (214.58, 391.37) | 186.42 | 7060.60 (5301.53, 9177.35) | 6846.19 (5131.67, 8844.85) | 7012.42 (5237.91, 9103.11) | 7045.14 (5231.20, 9226.39) | -0.04 (-0.09, 0.02) | -0.37 (-0.63, -0.10) | 0.26 (0.19, 0.34) | 0.11 (-0.01, 0.23) |
| Libya | 78.80 (58.58, 104.92) | 205.30 (150.69, 269.89) | 160.56 | 2660.50 (2001.41, 3443.44) | 2254.25 (1721.87, 2901.09) | 2240.13 (1719.91, 2883.84) | 2546.17 (1900.17, 3292.94) | -0.15 (-0.45, 0.14) | -1.70 (-2.14, -1.26) | -0.07 (-0.17, 0.02) | 1.63 (1.06, 2.19) |
| Lithuania | 115.04 (86.60, 149.44) | 93.46 (69.87, 117.65) | -18.75 | 2909.03 (2172.82, 3821.63) | 2895.17 (2132.82, 3767.63) | 2941.17 (2198.89, 3838.99) | 2969.72 (2188.61, 3882.90) | 0.05 (0.03, 0.08) | -0.06 (-0.10, -0.02) | 0.16 (0.07, 0.26) | 0.06 (-0.01, 0.14) |
| Luxembourg | 10.52 (7.82, 13.68) | 17.69 (13.07, 22.57) | 68.19 | 2328.95 (1732.18, 3051.12) | 2332.27 (1746.74, 3058.49) | 2351.36 (1739.01, 3052.12) | 2356.49 (1733.20, 3066.20) | 0.02 (0.01, 0.04) | 0.00 (-0.06, 0.06) | 0.09 (0.06, 0.12) | 0.03 (-0.02, 0.07) |
| Madagascar | 808.59 (609.06, 1085.03) | 2048.33 (1502.39, 2726.36) | 153.32 | 9673.25 (7214.64, 12452.88) | 9425.44 (7038.78, 12213.21) | 9434.12 (7028.55, 12262.07) | 9542.21 (7034.67, 12386.38) | -0.05 (-0.11, 0.00) | -0.30 (-0.47, -0.12) | 0.01 (-0.01, 0.04) | 0.25 (0.10, 0.41) |
| Malawi | 559.27 (415.52, 735.56) | 1151.95 (839.78, 1538.96) | 105.97 | 8437.00 (6224.36, 10836.08) | 7604.11 (5636.78, 9729.21) | 8342.33 (6106.22, 10763.09) | 8461.33 (6170.47, 11044.24) | 0.09 (-0.11, 0.28) | -1.09 (-1.56, -0.61) | 0.95 (0.59, 1.31) | 0.26 (0.13, 0.40) |
| Malaysia | 664.93 (489.25, 899.12) | 1475.31 (1089.32, 1961.64) | 121.88 | 4290.00 (3177.84, 5592.66) | 4322.14 (3216.35, 5630.15) | 4355.48 (3224.57, 5712.73) | 4338.09 (3207.72, 5689.50) | 0.01 (-0.01, 0.03) | 0.07 (0.01, 0.12) | 0.08 (-0.02, 0.18) | -0.10 (-0.19, -0.01) |
| Maldives | 6.41 (4.82, 8.57) | 28.97 (20.78, 39.98) | 351.95 | 4308.60 (3206.41, 5603.02) | 4288.89 (3209.06, 5563.93) | 4378.72 (3249.81, 5735.10) | 4503.00 (3346.77, 5914.31) | 0.13 (0.08, 0.17) | -0.05 (-0.08, -0.02) | 0.21 (0.08, 0.34) | 0.25 (0.16, 0.34) |
| Mali | 518.71 (393.70, 682.81) | 1265.02 (952.99, 1686.10) | 143.88 | 8405.38 (6414.25, 10814.68) | 8698.98 (6585.99, 11133.39) | 8439.21 (6342.78, 10943.63) | 8338.64 (6231.17, 10798.55) | -0.29 (-0.39, -0.19) | 0.19 (-0.50, 0.87) | -0.28 (-0.44, -0.12) | -0.08 (-0.16, 0.01) |
| Malta | 9.54 (7.13, 12.49) | 11.91 (8.90, 14.99) | 24.87 | 2305.84 (1730.14, 3006.83) | 2320.35 (1709.29, 3038.63) | 2353.42 (1750.94, 3092.05) | 2342.35 (1728.29, 3040.32) | 0.06 (0.05, 0.07) | 0.05 (0.02, 0.09) | 0.15 (0.12, 0.18) | -0.06 (-0.08, -0.04) |
| Marshall Islands | 2.09 (1.56, 2.86) | 3.78 (2.73, 5.00) | 80.63 | 6546.54 (4926.61, 8563.66) | 6625.07 (4960.11, 8569.30) | 6786.09 (4981.02, 8924.85) | 6654.25 (4871.49, 8647.14) | 0.05 (0.02, 0.08) | 0.10 (0.00, 0.19) | 0.25 (0.11, 0.39) | -0.19 (-0.32, -0.06) |
| Mauritania | 107.34 (80.74, 142.93) | 228.39 (169.62, 303.50) | 112.77 | 7147.84 (5316.39, 9271.54) | 6995.16 (5236.16, 9072.46) | 7107.82 (5311.55, 9205.00) | 7117.35 (5243.70, 9282.44) | -0.06 (-0.10, -0.02) | -0.27 (-0.51, -0.02) | 0.18 (0.12, 0.24) | 0.06 (-0.06, 0.17) |
| Mauritius | 46.93 (34.61, 63.40) | 64.72 (48.71, 83.78) | 37.90 | 4270.49 (3169.03, 5567.37) | 4274.72 (3185.04, 5557.54) | 4336.18 (3232.51, 5667.59) | 4325.01 (3236.08, 5711.95) | 0.02 (0.00, 0.05) | 0.01 (-0.01, 0.02) | 0.15 (0.06, 0.24) | -0.14 (-0.29, 0.01) |
| Mexico | 4448.34 (3263.34, 5939.87) | 8935.46 (6405.96, 11754.50) | 100.87 | 6538.21 (4734.61, 8527.21) | 6517.55 (4741.20, 8480.73) | 6613.30 (4805.83, 8634.18) | 6747.95 (4862.47, 8868.79) | 0.09 (0.05, 0.12) | -0.06 (-0.16, 0.04) | 0.15 (0.08, 0.23) | 0.34 (0.17, 0.51) |
| Micronesia (Federated States of) | 5.06 (3.80, 6.81) | 6.55 (4.90, 8.58) | 29.39 | 6548.54 (4970.37, 8524.97) | 6718.48 (4986.58, 8766.21) | 6815.84 (5085.93, 8902.10) | 6684.43 (4974.76, 8729.07) | 0.04 (0.00, 0.07) | 0.24 (0.15, 0.33) | 0.15 (0.04, 0.27) | -0.23 (-0.36, -0.11) |
| Monaco | 0.83 (0.62, 1.06) | 0.96 (0.74, 1.21) | 15.84 | 2315.10 (1703.97, 3014.08) | 2337.71 (1729.14, 3077.68) | 2355.59 (1761.28, 3058.98) | 2314.58 (1740.85, 3012.58) | -0.02 (-0.04, 0.01) | 0.09 (0.01, 0.16) | 0.09 (0.04, 0.13) | -0.21 (-0.27, -0.16) |
| Mongolia | 63.45 (47.41, 85.39) | 147.17 (106.58, 195.50) | 131.96 | 3891.68 (2903.93, 5058.18) | 3915.80 (3039.97, 4951.67) | 3964.51 (3088.36, 5085.95) | 3934.71 (2910.14, 5136.42) | 0.02 (0.00, 0.05) | 0.07 (0.05, 0.09) | 0.13 (0.00, 0.26) | -0.13 (-0.31, 0.04) |
| Montenegro | 21.32 (15.91, 27.88) | 23.52 (17.55, 30.29) | 10.35 | 3265.77 (2429.56, 4259.47) | 3284.18 (2451.38, 4287.77) | 3312.34 (2444.72, 4356.95) | 3323.29 (2471.48, 4354.45) | 0.03 (0.01, 0.05) | 0.05 (0.02, 0.08) | 0.09 (0.00, 0.18) | 0.04 (-0.10, 0.18) |
| Morocco | 758.45 (565.81, 1003.88) | 1382.73 (1027.60, 1808.31) | 82.31 | 3722.67 (2803.99, 4808.97) | 3656.25 (2754.20, 4667.76) | 3963.61 (2979.20, 5050.66) | 3602.30 (2693.40, 4692.07) | 0.18 (0.05, 0.32) | -0.17 (-0.23, -0.10) | 0.89 (0.76, 1.02) | -1.19 (-1.67, -0.70) |
| Mozambique | 1068.56 (805.12, 1389.55) | 2144.55 (1610.20, 2845.94) | 100.70 | 11216.69 (8483.99, 14397.76) | 10384.75 (7821.95, 13415.07) | 10265.41 (7689.36, 13196.93) | 10335.54 (7782.31, 13249.63) | -0.36 (-0.48, -0.24) | -0.86 (-1.37, -0.34) | -0.11 (-0.15, -0.08) | 0.18 (0.01, 0.34) |
| Myanmar | 1462.50 (1088.60, 1961.64) | 2423.53 (1782.25, 3175.25) | 65.71 | 4283.05 (3196.73, 5617.25) | 4248.81 (3163.06, 5566.24) | 4306.70 (3186.86, 5646.96) | 4254.28 (3139.71, 5551.18) | -0.03 (-0.05, 0.00) | -0.09 (-0.13, -0.04) | 0.15 (0.06, 0.23) | -0.20 (-0.29, -0.11) |
| Namibia | 76.84 (58.19, 101.79) | 161.95 (120.15, 214.39) | 110.76 | 7275.43 (5463.96, 9460.85) | 6983.84 (5276.92, 9042.80) | 7360.03 (5549.48, 9580.59) | 7327.11 (5414.01, 9515.33) | 0.03 (-0.04, 0.10) | -0.46 (-0.76, -0.16) | 0.57 (0.52, 0.63) | 0.01 (-0.17, 0.18) |
| Nauru | 0.58 (0.42, 0.78) | 0.67 (0.49, 0.91) | 16.90 | 6759.51 (4947.99, 8820.88) | 6960.79 (5070.47, 9136.50) | 7059.35 (5244.44, 9166.56) | 6927.20 (5106.31, 9110.24) | 0.04 (0.00, 0.08) | 0.27 (0.17, 0.38) | 0.15 (-0.03, 0.33) | -0.28 (-0.37, -0.18) |
| Nepal | 420.53 (311.86, 567.33) | 776.18 (577.78, 1032.03) | 84.57 | 2807.56 (2099.89, 3699.48) | 2817.01 (2115.62, 3696.42) | 2734.09 (2034.04, 3552.23) | 2707.73 (2016.10, 3555.21) | -0.16 (-0.19, -0.14) | 0.04 (0.03, 0.05) | -0.31 (-0.34, -0.27) | -0.09 (-0.13, -0.05) |
| Netherlands | 370.64 (278.85, 487.12) | 412.58 (316.61, 521.25) | 11.31 | 2155.25 (1609.85, 2830.99) | 2031.36 (1525.24, 2658.40) | 2036.55 (1515.82, 2640.25) | 2162.71 (1612.03, 2841.64) | 0.12 (0.01, 0.22) | -0.53 (-0.75, -0.30) | 0.02 (-0.07, 0.10) | 0.73 (0.51, 0.95) |
| New Zealand | 108.86 (79.87, 142.45) | 142.86 (107.18, 183.40) | 31.24 | 2999.22 (2192.03, 3914.24) | 2980.90 (2169.10, 3904.60) | 2925.02 (2136.92, 3823.85) | 2988.44 (2181.60, 3901.09) | -0.06 (-0.11, -0.01) | -0.06 (-0.08, -0.04) | -0.20 (-0.37, -0.02) | 0.27 (0.22, 0.31) |
| Nicaragua | 161.58 (118.91, 220.83) | 400.78 (295.37, 535.92) | 148.03 | 6086.16 (4500.36, 7981.99) | 5998.75 (4469.01, 7800.23) | 6070.46 (4534.35, 7859.17) | 6259.34 (4620.32, 8246.36) | 0.07 (0.02, 0.11) | -0.16 (-0.23, -0.09) | 0.13 (0.02, 0.24) | 0.49 (0.22, 0.76) |
| Niger | 384.34 (288.24, 520.76) | 1020.63 (764.97, 1365.67) | 165.55 | 7164.15 (5339.75, 9380.32) | 6960.41 (5235.21, 9010.29) | 7122.17 (5354.42, 9395.07) | 7100.25 (5294.57, 9229.14) | -0.05 (-0.10, 0.00) | -0.34 (-0.57, -0.10) | 0.26 (0.20, 0.31) | 0.02 (-0.09, 0.12) |
| Nigeria | 5225.26 (3869.04, 6923.98) | 13272.72 (9768.12, 17687.62) | 154.01 | 7750.34 (5685.65, 10081.66) | 8033.73 (5885.64, 10407.25) | 8028.34 (5882.50, 10465.79) | 8162.22 (5968.34, 10656.20) | -0.05 (-0.12, 0.03) | 0.21 (-0.37, 0.80) | 0.00 (-0.19, 0.19) | 0.23 (0.11, 0.35) |
| Niue | 0.13 (0.10, 0.18) | 0.12 (0.09, 0.15) | -13.11 | 6817.82 (4985.95, 8982.36) | 6894.23 (5010.90, 9074.47) | 6945.80 (5049.91, 9132.08) | 6897.66 (5116.77, 9040.12) | 0.01 (-0.03, 0.04) | 0.10 (0.02, 0.17) | 0.07 (-0.14, 0.29) | -0.14 (-0.21, -0.07) |
| North Macedonia | 68.62 (50.28, 90.92) | 86.55 (64.11, 112.37) | 26.14 | 3254.87 (2393.59, 4295.10) | 3262.56 (2412.70, 4259.57) | 3294.88 (2446.67, 4294.29) | 3319.03 (2442.89, 4342.55) | 0.05 (0.04, 0.07) | 0.03 (0.02, 0.04) | 0.10 (0.04, 0.17) | 0.06 (-0.03, 0.15) |
| Northern Mariana Islands | 3.45 (2.51, 4.73) | 3.13 (2.31, 4.08) | -9.18 | 6529.17 (4883.40, 8559.44) | 6958.93 (5216.66, 9254.84) | 6992.20 (5202.26, 9213.57) | 6760.39 (4960.14, 8872.51) | 0.07 (0.01, 0.13) | 0.55 (0.47, 0.63) | 0.06 (-0.15, 0.27) | -0.38 (-0.49, -0.26) |
| Norway | 116.87 (85.74, 152.14) | 153.13 (113.99, 195.29) | 31.03 | 2507.27 (1835.43, 3293.06) | 2493.51 (1833.44, 3255.06) | 2540.58 (1857.77, 3310.94) | 2543.15 (1853.88, 3323.57) | 0.05 (0.03, 0.08) | -0.07 (-0.15, 0.01) | 0.20 (0.12, 0.27) | 0.01 (-0.01, 0.02) |
| Oman | 61.15 (45.13, 83.16) | 221.11 (158.24, 304.45) | 261.57 | 3818.29 (2849.65, 4942.68) | 3818.45 (2871.81, 4895.86) | 3760.06 (2776.64, 4813.54) | 3833.62 (2839.29, 4928.99) | 0.03 (-0.04, 0.10) | 0.03 (-0.04, 0.10) | -0.23 (-0.48, 0.01) | 0.14 (-0.17, 0.45) |
| Pakistan | 2505.68 (1840.25, 3338.59) | 5749.91 (4199.40, 7701.65) | 129.48 | 3115.09 (2257.03, 4086.57) | 3087.22 (2280.93, 4060.74) | 3086.82 (2250.39, 4057.38) | 3104.35 (2275.29, 4063.31) | -0.05 (-0.07, -0.02) | -0.09 (-0.13, -0.06) | 0.00 (-0.09, 0.10) | 0.09 (-0.07, 0.24) |
| Palau | 1.03 (0.75, 1.38) | 1.45 (1.05, 1.87) | 40.18 | 6790.75 (4950.24, 8855.56) | 6681.55 (4890.94, 8673.54) | 6856.27 (5015.88, 9005.01) | 6568.41 (4775.35, 8518.40) | -0.11 (-0.17, -0.05) | -0.23 (-0.49, 0.04) | 0.31 (0.16, 0.46) | -0.55 (-0.63, -0.48) |
| Palestine | 51.44 (38.35, 69.24) | 162.36 (120.07, 214.12) | 215.63 | 3905.51 (2910.12, 5087.79) | 4337.41 (3293.66, 5574.89) | 4281.39 (3264.50, 5461.38) | 3876.23 (2861.68, 5006.03) | 0.01 (-0.17, 0.19) | 1.12 (0.77, 1.47) | -0.14 (-0.22, -0.05) | -1.09 (-1.43, -0.74) |
| Panama | 125.61 (93.35, 167.16) | 263.82 (194.02, 342.14) | 110.02 | 5982.04 (4415.25, 7735.53) | 5949.62 (4416.92, 7768.81) | 6071.71 (4536.19, 7943.07) | 6254.16 (4587.42, 8134.21) | 0.11 (0.07, 0.15) | -0.08 (-0.19, 0.03) | 0.21 (0.12, 0.31) | 0.44 (0.22, 0.65) |
| Papua New Guinea | 235.02 (176.01, 317.22) | 647.51 (481.46, 848.98) | 175.51 | 7065.21 (5349.94, 9229.99) | 7249.66 (5455.61, 9395.02) | 7734.34 (5992.52, 9740.11) | 7187.42 (5378.95, 9208.44) | 0.24 (0.15, 0.32) | 0.24 (0.15, 0.33) | 0.67 (0.36, 0.99) | -0.72 (-0.99, -0.44) |
| Paraguay | 167.73 (124.96, 224.39) | 378.03 (281.30, 502.93) | 125.38 | 5247.53 (3904.48, 6815.30) | 5217.81 (3876.25, 6770.24) | 5270.12 (3928.84, 6858.56) | 5417.54 (4040.11, 7084.52) | 0.03 (-0.01, 0.08) | -0.10 (-0.29, 0.09) | 0.11 (-0.02, 0.23) | 0.40 (0.20, 0.60) |
| Peru | 789.50 (580.92, 1059.57) | 1590.67 (1160.48, 2104.24) | 101.48 | 4503.19 (3323.24, 5862.95) | 4393.58 (3270.27, 5653.48) | 4418.75 (3283.38, 5827.48) | 4491.89 (3286.86, 5915.92) | -0.02 (-0.07, 0.02) | -0.25 (-0.32, -0.18) | 0.07 (0.03, 0.10) | 0.27 (0.11, 0.44) |
| Philippines | 2450.69 (1789.95, 3301.62) | 5190.17 (3772.77, 6870.13) | 111.78 | 4820.03 (3507.75, 6273.68) | 4801.57 (3484.65, 6267.63) | 4818.16 (3498.27, 6298.35) | 4801.82 (3467.91, 6257.34) | -0.03 (-0.05, -0.02) | -0.04 (-0.05, -0.02) | 0.04 (-0.06, 0.14) | -0.10 (-0.20, 0.00) |
| Poland | 1509.40 (1086.95, 1989.70) | 1718.12 (1275.60, 2225.06) | 13.83 | 3641.73 (2647.25, 4777.23) | 3648.24 (2635.83, 4752.98) | 3692.20 (2695.20, 4821.13) | 3732.22 (2734.00, 4920.96) | 0.06 (0.04, 0.08) | 0.02 (0.00, 0.05) | 0.13 (0.03, 0.22) | 0.07 (-0.08, 0.23) |
| Portugal | 263.46 (196.46, 339.69) | 303.03 (229.07, 383.04) | 15.02 | 2442.98 (1800.93, 3214.23) | 2456.24 (1837.35, 3198.65) | 2492.98 (1865.87, 3253.85) | 2448.15 (1812.26, 3195.74) | 0.00 (-0.03, 0.02) | 0.03 (-0.07, 0.14) | 0.16 (0.12, 0.20) | -0.24 (-0.29, -0.20) |
| Puerto Rico | 182.28 (136.85, 236.29) | 203.78 (154.38, 257.93) | 11.80 | 5056.07 (3789.93, 6550.50) | 5103.45 (3811.07, 6678.72) | 5136.97 (3828.28, 6740.06) | 5266.45 (3896.74, 6811.61) | 0.11 (0.08, 0.14) | 0.08 (0.04, 0.12) | 0.07 (0.04, 0.09) | 0.40 (0.19, 0.62) |
| Qatar | 21.01 (14.91, 29.02) | 165.82 (118.93, 229.53) | 689.11 | 4019.89 (2995.64, 5201.54) | 4035.78 (3007.20, 5204.13) | 4139.41 (3065.51, 5351.47) | 4026.75 (2997.02, 5202.16) | 0.08 (0.05, 0.11) | 0.04 (0.02, 0.06) | 0.28 (0.19, 0.38) | -0.29 (-0.34, -0.25) |
| Republic of Korea | 1430.66 (1052.20, 1931.38) | 2067.72 (1533.49, 2641.71) | 44.53 | 3038.35 (2266.47, 3974.08) | 2995.92 (2245.12, 3958.24) | 2960.74 (2197.27, 3860.08) | 3093.46 (2297.32, 4040.52) | -0.02 (-0.06, 0.03) | -0.15 (-0.19, -0.11) | -0.12 (-0.29, 0.04) | 0.46 (0.37, 0.54) |
| Republic of Moldova | 134.26 (99.31, 176.42) | 133.42 (98.69, 172.26) | -0.62 | 2904.00 (2149.36, 3794.24) | 2893.58 (2160.84, 3746.51) | 2940.01 (2159.15, 3850.19) | 2958.34 (2178.41, 3857.89) | 0.07 (0.05, 0.10) | -0.04 (-0.05, -0.03) | 0.16 (0.06, 0.27) | 0.02 (-0.04, 0.08) |
| Romania | 799.58 (592.67, 1038.23) | 744.45 (557.03, 955.52) | -6.90 | 3254.49 (2405.95, 4269.73) | 3294.26 (2468.27, 4288.73) | 3319.64 (2473.14, 4362.83) | 3327.29 (2481.90, 4356.66) | 0.05 (0.04, 0.06) | 0.12 (0.11, 0.13) | 0.08 (0.00, 0.16) | -0.02 (-0.11, 0.07) |
| Russian Federation | 5252.95 (3823.47, 6849.25) | 5494.80 (4002.32, 7105.36) | 4.60 | 3144.16 (2277.72, 4096.44) | 3145.10 (2261.24, 4126.81) | 3121.68 (2258.74, 4089.18) | 3181.37 (2279.57, 4164.99) | 0.01 (-0.03, 0.04) | 0.00 (-0.02, 0.02) | -0.08 (-0.19, 0.02) | 0.21 (0.19, 0.22) |
| Rwanda | 477.22 (359.53, 645.13) | 1016.87 (757.78, 1350.56) | 113.08 | 9639.20 (7202.68, 12561.88) | 9372.07 (7169.57, 12195.60) | 9270.67 (6852.86, 12095.66) | 9483.78 (7072.72, 12316.60) | -0.10 (-0.16, -0.03) | -0.33 (-0.50, -0.16) | -0.11 (-0.15, -0.08) | 0.36 (0.23, 0.48) |
| Saint Kitts and Nevis | 1.78 (1.30, 2.39) | 3.72 (2.73, 4.83) | 108.81 | 5186.30 (3766.02, 6712.87) | 5182.47 (3792.98, 6770.60) | 5220.99 (3868.63, 6744.26) | 5309.55 (3895.46, 6958.14) | 0.07 (0.04, 0.09) | -0.02 (-0.05, 0.02) | 0.08 (0.02, 0.13) | 0.28 (0.14, 0.41) |
| Saint Lucia | 5.45 (4.04, 7.36) | 10.71 (7.95, 13.90) | 96.71 | 5041.15 (3741.71, 6651.02) | 5066.10 (3755.89, 6534.21) | 5128.63 (3800.89, 6720.70) | 5221.69 (3880.86, 6831.82) | 0.09 (0.06, 0.12) | 0.03 (-0.06, 0.11) | 0.13 (0.11, 0.15) | 0.30 (0.12, 0.49) |
| Saint Vincent and the Grenadines | 4.34 (3.23, 5.82) | 6.40 (4.78, 8.19) | 47.46 | 5061.85 (3770.88, 6560.68) | 5084.67 (3769.42, 6634.71) | 5112.40 (3792.07, 6656.20) | 5190.30 (3846.96, 6718.64) | 0.08 (0.06, 0.11) | 0.05 (0.03, 0.07) | 0.05 (0.04, 0.07) | 0.29 (0.10, 0.49) |
| Samoa | 8.17 (6.14, 11.21) | 12.51 (9.32, 16.30) | 53.19 | 6591.25 (4912.31, 8738.48) | 6693.40 (5025.32, 8742.75) | 6850.37 (5076.30, 8959.79) | 6754.43 (5035.87, 8793.27) | 0.06 (0.03, 0.10) | 0.13 (0.03, 0.22) | 0.24 (0.10, 0.38) | -0.20 (-0.30, -0.10) |
| San Marino | 0.60 (0.45, 0.78) | 0.86 (0.64, 1.08) | 41.72 | 2362.92 (1738.76, 3092.67) | 2341.65 (1730.01, 3085.04) | 2311.03 (1724.13, 3018.36) | 2279.51 (1708.41, 2969.10) | -0.15 (-0.16, -0.13) | -0.10 (-0.14, -0.05) | -0.13 (-0.20, -0.05) | -0.17 (-0.20, -0.14) |
| Sao Tome and Principe | 5.59 (4.19, 7.39) | 12.92 (9.51, 17.14) | 131.16 | 6979.00 (5193.50, 9087.57) | 6775.29 (5097.95, 8709.48) | 7017.49 (5247.61, 9199.73) | 7032.67 (5196.93, 9120.12) | 0.01 (-0.05, 0.07) | -0.36 (-0.65, -0.07) | 0.38 (0.34, 0.43) | 0.09 (-0.04, 0.22) |
| Saudi Arabia | 648.61 (478.54, 866.49) | 2331.35 (1666.03, 3105.86) | 259.44 | 5167.58 (3842.33, 6651.74) | 5505.08 (4161.83, 6991.00) | 5362.94 (4042.09, 6888.34) | 4971.41 (3700.13, 6385.41) | -0.11 (-0.24, 0.02) | 0.67 (0.39, 0.94) | -0.26 (-0.29, -0.23) | -0.87 (-1.07, -0.68) |
| Senegal | 372.81 (279.84, 499.16) | 853.58 (631.78, 1136.49) | 128.96 | 7148.42 (5395.20, 9293.64) | 6916.55 (5183.57, 9017.28) | 7042.53 (5301.37, 9148.63) | 7053.45 (5195.06, 9116.30) | -0.07 (-0.13, -0.02) | -0.38 (-0.62, -0.14) | 0.20 (0.18, 0.23) | 0.06 (-0.03, 0.15) |
| Serbia | 337.23 (254.31, 436.05) | 325.13 (243.68, 413.63) | -3.59 | 3262.48 (2432.42, 4277.91) | 3262.54 (2435.67, 4251.26) | 3278.62 (2456.37, 4282.52) | 3304.07 (2427.71, 4318.97) | 0.02 (0.00, 0.03) | 0.00 (-0.01, 0.01) | 0.05 (-0.05, 0.15) | 0.07 (-0.04, 0.18) |
| Seychelles | 2.77 (2.06, 3.69) | 5.28 (3.89, 6.87) | 90.34 | 4309.58 (3191.97, 5610.78) | 4300.77 (3199.16, 5630.53) | 4373.78 (3233.85, 5671.93) | 4374.45 (3245.63, 5701.81) | 0.06 (0.04, 0.08) | -0.03 (-0.04, -0.02) | 0.18 (0.10, 0.26) | -0.05 (-0.13, 0.03) |
| Sierra Leone | 197.01 (149.10, 264.78) | 469.62 (346.29, 628.10) | 138.37 | 7039.77 (5330.42, 9142.22) | 6839.59 (5133.52, 8937.51) | 7002.25 (5278.25, 9192.99) | 7024.52 (5224.79, 9150.97) | -0.05 (-0.10, 0.01) | -0.34 (-0.62, -0.06) | 0.26 (0.22, 0.30) | 0.09 (-0.03, 0.21) |
| Singapore | 107.45 (79.17, 143.32) | 236.13 (171.89, 308.83) | 119.77 | 3018.54 (2253.26, 3914.80) | 2998.14 (2231.88, 3882.28) | 3073.76 (2285.53, 4017.94) | 3097.38 (2308.92, 4068.85) | 0.11 (0.09, 0.13) | -0.07 (-0.15, 0.02) | 0.27 (0.20, 0.33) | 0.10 (0.04, 0.16) |
| Slovakia | 181.40 (133.18, 237.23) | 219.26 (161.46, 282.98) | 20.87 | 3235.01 (2375.46, 4217.98) | 3244.04 (2415.34, 4239.05) | 3292.19 (2451.24, 4311.40) | 3316.57 (2442.11, 4341.21) | 0.06 (0.05, 0.08) | 0.02 (0.00, 0.05) | 0.16 (0.07, 0.24) | 0.04 (-0.06, 0.14) |
| Slovenia | 72.43 (53.50, 93.86) | 80.89 (60.72, 102.81) | 11.69 | 3264.30 (2409.80, 4267.17) | 3272.29 (2428.33, 4281.91) | 3310.47 (2443.87, 4332.11) | 3333.55 (2456.15, 4331.79) | 0.06 (0.05, 0.08) | 0.03 (0.02, 0.03) | 0.12 (0.05, 0.20) | 0.04 (-0.03, 0.12) |
| Solomon Islands | 15.72 (11.87, 21.28) | 38.66 (28.32, 51.46) | 145.97 | 6558.12 (4891.33, 8594.57) | 6666.36 (5037.93, 8631.02) | 6808.22 (5051.67, 8891.40) | 6750.57 (5007.32, 8787.32) | 0.06 (0.03, 0.09) | 0.14 (0.04, 0.24) | 0.22 (0.10, 0.34) | -0.13 (-0.28, 0.01) |
| Somalia | 499.34 (368.14, 670.19) | 1381.37 (1023.41, 1844.65) | 176.64 | 9728.52 (7282.51, 12705.48) | 9384.05 (7087.23, 12086.14) | 9396.27 (7000.93, 12224.77) | 9574.72 (7057.40, 12336.29) | -0.08 (-0.14, -0.01) | -0.40 (-0.59, -0.21) | 0.02 (-0.02, 0.05) | 0.31 (0.20, 0.43) |
| South Africa | 2832.35 (2070.93, 3721.52) | 4777.07 (3488.87, 6323.89) | 68.66 | 8720.77 (6412.97, 11307.48) | 7992.96 (5841.74, 10366.49) | 7320.00 (5343.18, 9483.02) | 7945.06 (5848.53, 10354.39) | -0.59 (-0.76, -0.42) | -0.90 (-1.35, -0.44) | -0.91 (-1.11, -0.71) | 1.17 (0.80, 1.54) |
| South Sudan | 413.27 (307.04, 553.66) | 653.48 (479.55, 855.65) | 58.12 | 10036.97 (7406.59, 13095.22) | 9745.36 (7338.99, 12704.96) | 9624.77 (7203.58, 12447.28) | 9660.87 (7164.45, 12478.59) | -0.15 (-0.20, -0.11) | -0.32 (-0.48, -0.17) | -0.12 (-0.16, -0.09) | 0.13 (0.02, 0.24) |
| Spain | 951.79 (718.72, 1229.83) | 1278.21 (936.24, 1655.00) | 34.30 | 2313.99 (1717.24, 3031.96) | 2312.63 (1740.13, 3027.26) | 2356.79 (1762.51, 3074.85) | 2330.95 (1728.49, 3065.81) | 0.05 (0.03, 0.07) | -0.01 (-0.04, 0.01) | 0.21 (0.17, 0.24) | -0.13 (-0.17, -0.09) |
| Sri Lanka | 814.58 (603.16, 1089.06) | 1170.09 (866.94, 1521.12) | 43.64 | 4957.81 (3692.73, 6485.84) | 5006.18 (3766.49, 6551.65) | 5071.99 (3800.98, 6551.34) | 4947.37 (3660.58, 6483.21) | -0.03 (-0.07, 0.01) | 0.08 (0.01, 0.15) | 0.14 (0.10, 0.19) | -0.41 (-0.57, -0.25) |
| Sudan | 560.78 (422.96, 744.76) | 1399.25 (1033.12, 1862.67) | 149.52 | 3891.41 (2931.08, 5021.89) | 4902.90 (3780.45, 6271.80) | 4268.20 (3203.19, 5472.82) | 4111.54 (3070.33, 5291.38) | -0.38 (-0.62, -0.13) | 2.05 (1.14, 2.96) | -1.44 (-1.97, -0.91) | -0.34 (-0.48, -0.20) |
| Suriname | 17.25 (13.05, 22.86) | 31.94 (23.77, 41.63) | 85.19 | 5098.57 (3808.84, 6657.27) | 5131.75 (3783.88, 6737.79) | 5146.20 (3854.74, 6716.30) | 5243.14 (3895.76, 6832.04) | 0.06 (0.04, 0.09) | 0.05 (0.01, 0.09) | 0.03 (0.00, 0.05) | 0.30 (0.13, 0.48) |
| Sweden | 240.68 (178.01, 311.50) | 283.77 (210.49, 367.14) | 17.90 | 2513.75 (1835.95, 3313.70) | 2513.84 (1837.07, 3295.03) | 2551.17 (1869.35, 3364.10) | 2543.46 (1860.04, 3377.23) | 0.02 (0.00, 0.04) | -0.02 (-0.12, 0.08) | 0.16 (0.10, 0.22) | -0.03 (-0.10, 0.04) |
| Switzerland | 189.53 (139.97, 246.97) | 241.50 (181.64, 307.44) | 27.42 | 2344.46 (1731.59, 3081.83) | 2320.41 (1729.98, 3018.55) | 2341.69 (1736.91, 3073.14) | 2341.30 (1751.45, 3071.47) | 0.01 (-0.01, 0.02) | -0.11 (-0.15, -0.08) | 0.10 (0.08, 0.12) | -0.01 (-0.04, 0.01) |
| Syrian Arab Republic | 300.43 (223.09, 400.63) | 487.19 (358.97, 633.69) | 62.17 | 3572.53 (2691.09, 4605.34) | 3594.89 (2685.42, 4628.25) | 3553.08 (2639.73, 4561.51) | 3424.58 (2561.24, 4428.29) | -0.12 (-0.15, -0.09) | 0.07 (0.06, 0.07) | -0.11 (-0.16, -0.06) | -0.35 (-0.43, -0.26) |
| Taiwan (Province of China) | 868.43 (655.16, 1157.15) | 1212.67 (906.46, 1555.68) | 39.64 | 4073.77 (3126.61, 5285.41) | 4184.02 (3090.64, 5528.82) | 4165.55 (3089.57, 5435.62) | 4125.91 (3085.42, 5402.93) | 0.09 (0.05, 0.13) | 0.30 (0.09, 0.51) | -0.05 (-0.10, 0.00) | -0.09 (-0.11, -0.06) |
| Tajikistan | 153.17 (112.96, 204.93) | 357.46 (263.70, 480.16) | 133.37 | 3902.56 (2882.64, 5090.37) | 3956.05 (2936.09, 5188.31) | 4015.99 (2988.70, 5244.43) | 3972.74 (2928.82, 5203.07) | 0.02 (-0.02, 0.05) | 0.13 (0.11, 0.16) | 0.16 (0.01, 0.31) | -0.17 (-0.43, 0.08) |
| Thailand | 2394.06 (1783.32, 3213.99) | 3716.40 (2741.75, 4747.21) | 55.23 | 4298.67 (3218.35, 5612.95) | 4292.80 (3197.80, 5625.25) | 4326.47 (3198.06, 5704.12) | 4313.85 (3209.82, 5645.64) | -0.02 (-0.04, 0.00) | -0.02 (-0.05, 0.02) | 0.09 (0.02, 0.17) | -0.11 (-0.24, 0.02) |
| Timor-Leste | 27.40 (20.15, 37.05) | 45.49 (33.87, 61.18) | 65.98 | 4355.72 (3240.55, 5658.25) | 4359.58 (3261.39, 5751.09) | 4396.37 (3246.29, 5780.90) | 4333.61 (3200.01, 5730.76) | -0.04 (-0.06, -0.01) | 0.01 (-0.03, 0.04) | 0.10 (-0.06, 0.26) | -0.23 (-0.35, -0.12) |
| Togo | 151.58 (110.21, 205.76) | 417.17 (308.73, 564.30) | 175.22 | 6107.95 (4474.20, 7983.90) | 5908.02 (4426.37, 7735.76) | 5927.69 (4495.88, 7693.77) | 6181.87 (4601.36, 8120.35) | -0.04 (-0.13, 0.04) | -0.35 (-0.48, -0.22) | 0.05 (-0.06, 0.17) | 0.62 (0.21, 1.02) |
| Tokelau | 0.09 (0.06, 0.12) | 0.09 (0.06, 0.12) | 1.56 | 6903.51 (5061.68, 9116.58) | 7022.40 (5161.56, 9168.23) | 7037.11 (5216.49, 9208.53) | 6864.42 (4990.77, 9007.38) | -0.06 (-0.09, -0.02) | 0.13 (0.02, 0.24) | -0.03 (-0.22, 0.17) | -0.34 (-0.41, -0.26) |
| Tonga | 4.80 (3.65, 6.31) | 6.12 (4.53, 8.01) | 27.43 | 6640.34 (5014.06, 8624.12) | 6694.62 (5048.73, 8691.45) | 6844.45 (5078.10, 8941.19) | 6834.70 (5038.69, 8947.71) | 0.06 (0.02, 0.09) | 0.04 (-0.11, 0.19) | 0.23 (0.08, 0.38) | -0.08 (-0.17, 0.01) |
| Trinidad and Tobago | 56.24 (41.55, 74.72) | 84.39 (62.34, 107.99) | 50.05 | 5042.34 (3726.99, 6546.17) | 5061.77 (3802.11, 6561.16) | 5130.36 (3787.93, 6756.51) | 5226.18 (3881.66, 6757.53) | 0.10 (0.07, 0.13) | 0.03 (-0.02, 0.07) | 0.14 (0.12, 0.17) | 0.31 (0.11, 0.52) |
| Tunisia | 278.74 (209.09, 364.36) | 518.40 (376.53, 666.29) | 85.98 | 4064.79 (3027.50, 5213.99) | 4437.07 (3411.07, 5684.81) | 4306.63 (3255.52, 5588.96) | 3912.92 (2862.19, 5020.96) | -0.20 (-0.33, -0.06) | 0.78 (0.44, 1.12) | -0.31 (-0.39, -0.23) | -1.25 (-1.78, -0.71) |
| Turkey | 1604.13 (1199.60, 2121.98) | 2709.94 (2022.48, 3484.27) | 68.94 | 3175.17 (2385.66, 4106.60) | 3020.48 (2273.26, 3862.60) | 2782.17 (2093.28, 3561.12) | 2825.29 (2127.37, 3611.17) | -0.64 (-0.74, -0.54) | -0.55 (-0.99, -0.11) | -0.84 (-0.91, -0.77) | 0.29 (0.08, 0.51) |
| Turkmenistan | 112.41 (82.51, 151.23) | 206.10 (151.42, 273.12) | 83.35 | 3871.19 (2866.78, 5040.90) | 3923.97 (2881.40, 5124.37) | 3953.42 (2947.33, 5215.34) | 3924.18 (2898.76, 5117.92) | 0.00 (-0.03, 0.03) | 0.13 (0.10, 0.16) | 0.08 (-0.08, 0.24) | -0.09 (-0.30, 0.12) |
| Tuvalu | 0.60 (0.45, 0.80) | 0.78 (0.57, 1.02) | 29.45 | 7053.03 (5231.16, 9216.03) | 7060.46 (5116.28, 9215.85) | 7021.27 (5099.19, 9233.28) | 6811.07 (4963.51, 8921.09) | -0.17 (-0.20, -0.14) | -0.02 (-0.16, 0.11) | -0.05 (-0.14, 0.04) | -0.41 (-0.50, -0.32) |
| Uganda | 880.91 (665.02, 1194.02) | 2195.52 (1634.37, 2938.20) | 149.23 | 7784.49 (5839.61, 10163.50) | 8015.25 (6158.42, 10204.01) | 7452.48 (5548.37, 9660.02) | 7703.18 (5737.70, 9930.65) | -0.11 (-0.19, -0.03) | 0.31 (0.19, 0.44) | -0.79 (-0.85, -0.74) | 0.48 (0.30, 0.66) |
| Ukraine | 1811.13 (1330.83, 2356.41) | 1713.70 (1260.50, 2215.47) | -5.38 | 3143.10 (2298.78, 4156.84) | 3140.31 (2289.08, 4104.88) | 3161.05 (2287.28, 4142.06) | 3221.74 (2352.81, 4229.55) | 0.05 (0.02, 0.08) | -0.01 (-0.03, 0.01) | 0.07 (-0.04, 0.17) | 0.14 (0.05, 0.23) |
| United Arab Emirates | 82.83 (59.10, 114.47) | 652.50 (446.80, 891.82) | 687.71 | 4014.95 (2997.29, 5153.09) | 4105.91 (3059.86, 5277.92) | 4053.24 (3011.20, 5321.21) | 4065.18 (3017.42, 5213.86) | -0.01 (-0.03, 0.02) | 0.23 (0.20, 0.27) | -0.14 (-0.24, -0.04) | 0.00 (-0.04, 0.05) |
| United Kingdom | 1548.92 (1146.81, 1995.70) | 1857.98 (1384.50, 2370.70) | 19.95 | 2451.60 (1798.01, 3206.32) | 2452.86 (1809.23, 3211.69) | 2472.90 (1818.49, 3227.00) | 2466.97 (1812.16, 3223.18) | 0.03 (0.02, 0.03) | 0.00 (-0.02, 0.02) | 0.09 (0.06, 0.12) | -0.04 (-0.05, -0.03) |
| United Republic of Tanzania | 2078.24 (1559.29, 2721.30) | 4939.75 (3688.18, 6469.13) | 137.69 | 11926.85 (8968.90, 15335.06) | 12200.40 (9573.34, 15255.68) | 11649.00 (8675.37, 14992.64) | 11552.60 (8518.32, 14853.91) | -0.15 (-0.22, -0.08) | 0.21 (0.19, 0.23) | -0.49 (-0.78, -0.19) | 0.02 (-0.12, 0.16) |
| United States of America | 12305.65 (8875.56, 16228.22) | 15098.75 (11151.49, 19342.75) | 22.70 | 4371.75 (3162.47, 5718.76) | 4272.34 (3088.25, 5592.12) | 4259.30 (3083.99, 5574.78) | 4258.18 (3065.61, 5593.67) | -0.10 (-0.13, -0.08) | -0.24 (-0.36, -0.13) | -0.03 (-0.05, -0.01) | -0.01 (-0.07, 0.05) |
| United States Virgin Islands | 5.54 (4.08, 7.28) | 5.80 (4.36, 7.34) | 4.73 | 5084.48 (3769.40, 6635.64) | 5094.08 (3761.71, 6621.58) | 5166.56 (3805.30, 6765.36) | 5239.13 (3876.71, 6773.64) | 0.09 (0.06, 0.12) | 0.01 (-0.01, 0.03) | 0.15 (0.13, 0.17) | 0.29 (0.03, 0.54) |
| Uruguay | 92.18 (69.18, 119.07) | 109.13 (82.14, 139.83) | 18.39 | 2947.04 (2188.87, 3852.25) | 2906.43 (2175.78, 3807.32) | 2942.83 (2191.10, 3901.19) | 2962.87 (2200.13, 3855.06) | 0.02 (0.00, 0.04) | -0.15 (-0.19, -0.10) | 0.13 (0.08, 0.18) | 0.06 (0.03, 0.09) |
| Uzbekistan | 628.93 (464.46, 847.67) | 1364.28 (1010.25, 1823.35) | 116.92 | 3869.71 (2866.96, 5045.24) | 3920.48 (2910.79, 5124.79) | 3979.94 (2912.08, 5170.56) | 3937.94 (2922.19, 5157.52) | 0.02 (-0.01, 0.05) | 0.12 (0.09, 0.16) | 0.16 (0.04, 0.28) | -0.17 (-0.38, 0.04) |
| Vanuatu | 7.74 (5.79, 10.51) | 17.39 (12.86, 23.23) | 124.74 | 6610.31 (4952.92, 8751.70) | 6715.93 (5025.93, 8799.16) | 6833.85 (5073.36, 9008.90) | 6743.37 (4926.89, 8762.43) | 0.05 (0.02, 0.08) | 0.14 (0.07, 0.21) | 0.18 (0.05, 0.32) | -0.20 (-0.29, -0.11) |
| Venezuela (Bolivarian Republic of) | 968.81 (720.06, 1305.51) | 1914.02 (1398.95, 2482.17) | 97.56 | 6036.32 (4481.78, 7870.55) | 5999.68 (4507.27, 7774.69) | 6082.69 (4534.53, 7857.33) | 6296.61 (4616.45, 8149.04) | 0.11 (0.06, 0.15) | -0.08 (-0.18, 0.01) | 0.14 (0.07, 0.22) | 0.53 (0.30, 0.75) |
| Viet Nam | 2064.31 (1513.23, 2796.06) | 4388.97 (3177.16, 5714.29) | 112.61 | 3796.66 (2840.59, 4963.57) | 3746.41 (2767.43, 4944.10) | 3812.99 (2832.32, 5013.13) | 3865.35 (2830.49, 5028.70) | 0.05 (0.01, 0.09) | -0.12 (-0.14, -0.11) | 0.19 (0.01, 0.37) | 0.10 (-0.01, 0.21) |
| Yemen | 302.23 (222.45, 409.86) | 873.87 (644.40, 1172.07) | 189.14 | 3563.85 (2646.82, 4635.74) | 3560.42 (2660.99, 4579.90) | 3522.99 (2635.17, 4549.62) | 3478.89 (2595.69, 4483.50) | -0.05 (-0.07, -0.03) | 0.00 (-0.01, 0.01) | -0.11 (-0.20, -0.02) | -0.03 (-0.16, 0.11) |
| Zambia | 572.41 (436.79, 758.15) | 1524.31 (1135.40, 2022.67) | 166.30 | 10887.84 (8203.19, 14101.51) | 10775.62 (8310.57, 13713.46) | 10969.59 (8312.65, 14113.80) | 10736.85 (7987.94, 13801.84) | -0.06 (-0.11, -0.02) | -0.18 (-0.51, 0.15) | 0.19 (0.10, 0.27) | -0.22 (-0.36, -0.08) |
| Zimbabwe | 548.59 (418.78, 735.19) | 968.15 (724.86, 1288.66) | 76.48 | 7666.85 (5793.43, 9992.74) | 7771.95 (6138.24, 9754.13) | 7736.17 (5855.37, 9993.03) | 7686.29 (5743.40, 9969.37) | -0.22 (-0.32, -0.13) | -0.03 (-0.70, 0.65) | -0.03 (-0.42, 0.35) | -0.02 (-0.18, 0.15) |
| **Genital herpes** | |  |  |  |  |  |  |  |  |  |  |
| Afghanistan | 75.69 (63.38, 91.44) | 274.06 (226.53, 331.62) | 262.10 | 764.08 (642.11, 918.67) | 744.95 (623.23, 883.71) | 733.35 (618.40, 870.21) | 736.72 (618.81, 876.82) | -0.11 (-0.13, -0.09) | -0.24 (-0.34, -0.14) | -0.15 (-0.18, -0.13) | 0.08 (0.03, 0.13) |
| Albania | 17.62 (14.56, 21.05) | 14.16 (11.98, 16.84) | -19.67 | 518.17 (432.73, 617.05) | 522.39 (440.23, 624.69) | 521.95 (438.94, 619.72) | 517.24 (431.30, 617.69) | 0.00 (-0.02, 0.01) | 0.06 (0.04, 0.09) | -0.01 (-0.02, -0.01) | -0.11 (-0.14, -0.08) |
| Algeria | 195.49 (161.74, 237.84) | 353.31 (294.53, 422.11) | 80.72 | 795.45 (669.00, 948.25) | 794.78 (664.39, 946.57) | 797.74 (672.52, 949.50) | 795.80 (667.31, 950.40) | -0.01 (-0.01, 0.00) | -0.01 (-0.04, 0.01) | 0.04 (0.03, 0.05) | -0.03 (-0.07, 0.00) |
| American Samoa | 0.68 (0.57, 0.82) | 0.75 (0.63, 0.88) | 9.06 | 1336.32 (1120.99, 1575.98) | 1340.12 (1124.37, 1571.32) | 1347.42 (1136.06, 1582.07) | 1339.43 (1123.43, 1576.58) | 0.02 (0.00, 0.03) | 0.03 (0.02, 0.04) | 0.06 (0.05, 0.06) | -0.13 (-0.22, -0.04) |
| Andorra | 0.43 (0.36, 0.52) | 0.56 (0.47, 0.66) | 28.13 | 678.40 (568.13, 808.47) | 686.66 (575.61, 814.85) | 686.02 (576.41, 816.83) | 684.74 (575.66, 814.66) | 0.03 (0.02, 0.05) | 0.14 (0.12, 0.16) | -0.03 (-0.09, 0.02) | -0.04 (-0.07, -0.01) |
| Angola | 238.07 (205.52, 272.26) | 704.25 (615.59, 810.71) | 195.82 | 2239.86 (1971.72, 2516.91) | 2275.83 (2008.08, 2559.82) | 2290.20 (2016.56, 2584.90) | 2278.53 (2020.35, 2579.70) | 0.08 (0.05, 0.10) | 0.16 (0.14, 0.19) | 0.06 (0.01, 0.11) | -0.05 (-0.08, -0.03) |
| Antigua and Barbuda | 1.07 (0.90, 1.26) | 1.52 (1.30, 1.76) | 41.44 | 1636.65 (1393.22, 1904.44) | 1655.31 (1406.42, 1938.21) | 1654.16 (1402.98, 1929.82) | 1623.81 (1386.98, 1891.36) | -0.03 (-0.05, 0.00) | 0.12 (0.11, 0.13) | -0.01 (-0.01, 0.00) | -0.18 (-0.27, -0.09) |
| Argentina | 407.62 (384.09, 432.97) | 560.75 (476.46, 651.28) | 37.57 | 1254.46 (1180.06, 1333.65) | 1252.32 (1166.31, 1337.63) | 1217.76 (1034.52, 1424.98) | 1218.53 (1033.30, 1420.30) | -0.14 (-0.16, -0.11) | -0.02 (-0.03, -0.01) | -0.28 (-0.42, -0.15) | 0.00 (-0.02, 0.03) |
| Armenia | 24.79 (20.50, 29.67) | 21.88 (18.40, 26.04) | -11.75 | 690.01 (577.52, 821.37) | 695.88 (581.59, 827.41) | 695.17 (584.37, 829.49) | 690.21 (579.61, 819.28) | 0.00 (-0.01, 0.01) | 0.09 (0.08, 0.09) | -0.01 (-0.05, 0.02) | -0.09 (-0.10, -0.08) |
| Australia | 183.58 (148.58, 223.68) | 186.01 (156.34, 219.80) | 1.32 | 1053.56 (852.59, 1285.43) | 897.57 (781.65, 1015.69) | 823.58 (687.35, 986.43) | 824.64 (690.83, 980.40) | -0.69 (-1.19, -0.18) | -1.42 (-2.08, -0.74) | -0.91 (-3.81, 2.07) | 0.02 (0.00, 0.05) |
| Austria | 52.15 (43.82, 61.99) | 53.68 (45.29, 63.24) | 2.93 | 642.87 (540.56, 760.84) | 651.01 (546.57, 772.59) | 643.33 (540.77, 762.83) | 638.72 (534.69, 758.04) | -0.03 (-0.05, -0.01) | 0.12 (0.10, 0.14) | -0.13 (-0.15, -0.11) | -0.08 (-0.18, 0.03) |
| Azerbaijan | 52.45 (43.07, 62.71) | 80.99 (67.80, 96.48) | 54.42 | 691.49 (577.79, 822.76) | 694.92 (585.57, 822.23) | 692.25 (581.89, 825.78) | 688.70 (579.94, 816.92) | -0.02 (-0.03, -0.01) | 0.05 (0.03, 0.07) | -0.04 (-0.07, 0.00) | -0.08 (-0.11, -0.04) |
| Bahamas | 4.89 (4.08, 5.80) | 6.60 (5.64, 7.68) | 35.10 | 1636.20 (1397.48, 1909.49) | 1639.70 (1393.49, 1902.17) | 1649.12 (1405.84, 1913.69) | 1634.72 (1391.51, 1908.26) | 0.01 (0.00, 0.03) | 0.03 (-0.01, 0.06) | 0.06 (0.04, 0.08) | -0.07 (-0.15, 0.01) |
| Bahrain | 4.48 (3.66, 5.47) | 12.74 (10.62, 15.50) | 184.35 | 755.42 (634.68, 902.17) | 756.63 (631.81, 893.90) | 739.83 (619.95, 880.77) | 758.44 (634.22, 904.07) | -0.06 (-0.09, -0.03) | 0.01 (-0.03, 0.05) | -0.27 (-0.31, -0.23) | 0.27 (0.22, 0.32) |
| Bangladesh | 554.18 (455.69, 662.39) | 980.21 (820.45, 1167.06) | 76.88 | 569.62 (480.09, 672.61) | 571.90 (478.94, 680.16) | 573.73 (484.04, 684.38) | 574.42 (483.59, 682.52) | 0.02 (0.02, 0.03) | 0.03 (0.01, 0.06) | 0.03 (0.03, 0.03) | 0.01 (0.01, 0.01) |
| Barbados | 4.52 (3.81, 5.31) | 4.44 (3.83, 5.16) | -1.61 | 1627.78 (1382.04, 1905.47) | 1629.66 (1381.21, 1904.41) | 1642.87 (1391.93, 1920.70) | 1626.67 (1388.08, 1899.37) | 0.02 (0.01, 0.04) | 0.02 (-0.02, 0.05) | 0.08 (0.08, 0.09) | -0.09 (-0.16, -0.02) |
| Belarus | 99.70 (84.14, 118.80) | 81.60 (68.89, 96.56) | -18.15 | 953.65 (806.20, 1129.04) | 949.37 (791.95, 1128.76) | 952.00 (799.72, 1128.01) | 950.24 (800.35, 1127.11) | 0.00 (-0.01, 0.00) | -0.04 (-0.06, -0.03) | 0.03 (0.02, 0.04) | -0.01 (-0.06, 0.03) |
| Belgium | 68.99 (58.21, 81.60) | 71.05 (60.33, 83.69) | 2.97 | 683.45 (571.84, 812.29) | 696.98 (651.36, 743.76) | 688.58 (579.12, 816.49) | 687.73 (577.89, 818.09) | 0.16 (0.08, 0.24) | 0.35 (-0.19, 0.89) | -0.13 (-0.18, -0.08) | 0.01 (-0.02, 0.04) |
| Belize | 2.98 (2.49, 3.53) | 7.50 (6.33, 8.81) | 151.45 | 1624.09 (1385.55, 1886.83) | 1635.74 (1390.18, 1915.45) | 1646.90 (1399.26, 1918.99) | 1634.11 (1392.73, 1909.25) | 0.03 (0.02, 0.05) | 0.08 (0.05, 0.11) | 0.07 (0.07, 0.08) | -0.07 (-0.14, 0.01) |
| Benin | 67.48 (56.15, 79.79) | 191.49 (160.09, 227.68) | 183.76 | 1541.57 (1299.88, 1799.71) | 1432.88 (1358.21, 1505.12) | 1643.29 (1484.96, 1821.37) | 1526.62 (1298.25, 1776.72) | 0.32 (0.12, 0.51) | -0.65 (-0.97, -0.32) | 1.41 (0.79, 2.03) | -0.86 (-1.16, -0.56) |
| Bermuda | 1.10 (0.93, 1.30) | 0.85 (0.74, 0.98) | -22.51 | 1629.11 (1382.68, 1896.32) | 1633.15 (1384.24, 1904.09) | 1648.04 (1398.38, 1912.22) | 1628.84 (1389.95, 1897.38) | 0.02 (0.00, 0.04) | 0.03 (0.01, 0.05) | 0.10 (0.09, 0.10) | -0.10 (-0.19, -0.02) |
| Bhutan | 3.25 (2.69, 3.91) | 4.86 (4.04, 5.82) | 49.41 | 563.65 (472.40, 673.40) | 562.95 (471.45, 669.63) | 561.43 (472.38, 666.10) | 566.30 (472.09, 676.45) | -0.01 (-0.02, 0.01) | -0.03 (-0.08, 0.01) | -0.02 (-0.04, -0.01) | 0.10 (0.09, 0.11) |
| Bolivia (Plurinational State of) | 112.57 (95.28, 132.02) | 219.14 (186.24, 254.68) | 94.66 | 1757.66 (1499.49, 2032.16) | 1755.56 (1506.96, 2038.10) | 1745.44 (1498.10, 2020.08) | 1742.24 (1487.14, 2014.24) | -0.03 (-0.04, -0.03) | -0.01 (-0.01, -0.01) | -0.06 (-0.06, -0.06) | -0.01 (-0.02, 0.01) |
| Bosnia and Herzegovina | 25.83 (21.66, 30.70) | 16.87 (14.24, 20.09) | -34.69 | 518.03 (432.96, 617.31) | 518.64 (437.91, 620.78) | 519.56 (436.92, 616.34) | 518.50 (432.71, 619.39) | -0.01 (-0.02, 0.01) | 0.01 (-0.06, 0.09) | 0.02 (-0.01, 0.05) | -0.03 (-0.06, 0.00) |
| Botswana | 29.41 (24.83, 34.48) | 61.90 (53.03, 71.25) | 110.46 | 2170.12 (1870.47, 2504.93) | 2295.54 (1985.38, 2632.52) | 2448.73 (2104.89, 2791.07) | 2279.26 (1965.19, 2612.87) | 0.19 (0.04, 0.34) | 0.53 (0.40, 0.66) | 0.65 (0.33, 0.97) | -0.86 (-1.16, -0.57) |
| Brazil | 3103.56 (2639.83, 3610.88) | 4400.73 (3793.27, 5083.25) | 41.80 | 1931.48 (1653.34, 2229.44) | 1980.47 (1701.60, 2272.29) | 2122.40 (1825.11, 2438.21) | 1911.34 (1637.74, 2215.36) | 0.05 (-0.09, 0.19) | 0.25 (0.17, 0.32) | 0.74 (0.61, 0.86) | -1.25 (-1.76, -0.73) |
| Brunei Darussalam | 2.08 (1.70, 2.50) | 3.76 (3.13, 4.48) | 80.99 | 710.77 (599.89, 847.57) | 731.13 (614.67, 867.78) | 723.39 (603.00, 861.30) | 715.41 (602.68, 842.01) | 0.00 (-0.04, 0.03) | 0.30 (0.29, 0.31) | -0.12 (-0.13, -0.10) | -0.12 (-0.13, -0.11) |
| Bulgaria | 58.25 (49.27, 69.41) | 42.96 (36.43, 50.90) | -26.24 | 682.48 (574.29, 813.68) | 762.91 (627.65, 920.36) | 680.41 (575.77, 805.33) | 678.73 (573.00, 805.38) | -0.42 (-0.58, -0.27) | 0.96 (0.43, 1.50) | -1.18 (-1.67, -0.68) | -0.04 (-0.06, -0.02) |
| Burkina Faso | 133.62 (111.81, 158.03) | 344.55 (290.01, 408.89) | 157.85 | 1601.49 (1361.15, 1874.10) | 1637.39 (1543.53, 1736.75) | 1587.63 (1339.06, 1843.99) | 1572.29 (1337.09, 1835.56) | -0.17 (-0.21, -0.13) | 0.17 (-0.03, 0.37) | -0.32 (-0.40, -0.24) | -0.11 (-0.13, -0.08) |
| Burundi | 110.76 (92.76, 132.17) | 245.68 (206.91, 289.26) | 121.82 | 2011.14 (1712.05, 2347.53) | 2048.51 (1754.16, 2400.05) | 2013.76 (1714.44, 2343.80) | 1998.36 (1705.97, 2312.58) | -0.08 (-0.10, -0.05) | 0.16 (0.07, 0.25) | -0.18 (-0.22, -0.14) | -0.09 (-0.12, -0.05) |
| Cabo Verde | 5.29 (4.37, 6.34) | 9.84 (8.24, 11.53) | 86.11 | 1581.20 (1338.07, 1842.17) | 1562.93 (1329.29, 1820.67) | 1555.09 (1314.39, 1805.57) | 1545.75 (1305.69, 1795.05) | -0.08 (-0.09, -0.07) | -0.12 (-0.13, -0.10) | -0.05 (-0.09, 0.00) | -0.08 (-0.10, -0.06) |
| Cambodia | 112.21 (93.19, 134.91) | 207.73 (171.96, 245.79) | 85.12 | 1151.19 (969.26, 1357.21) | 1149.31 (967.02, 1359.50) | 1145.35 (963.38, 1344.20) | 1145.86 (963.37, 1350.19) | -0.03 (-0.04, -0.03) | -0.02 (-0.05, 0.01) | -0.03 (-0.05, -0.02) | -0.04 (-0.09, 0.02) |
| Cameroon | 191.29 (161.67, 223.58) | 601.91 (508.24, 700.39) | 214.66 | 1906.99 (1636.15, 2191.15) | 2398.62 (2258.51, 2546.13) | 1931.85 (1658.14, 2219.52) | 1908.94 (1638.28, 2206.08) | -0.74 (-1.04, -0.43) | 2.02 (1.09, 2.97) | -2.25 (-3.18, -1.31) | -0.12 (-0.15, -0.10) |
| Canada | 200.37 (166.26, 238.86) | 229.61 (195.16, 270.87) | 14.59 | 691.68 (576.84, 824.00) | 636.93 (563.92, 718.89) | 810.86 (762.63, 855.25) | 692.56 (581.24, 821.80) | 0.74 (0.43, 1.05) | -0.72 (-1.11, -0.33) | 2.63 (2.10, 3.17) | -2.15 (-3.15, -1.13) |
| Central African Republic | 65.42 (56.51, 74.68) | 131.03 (113.59, 150.24) | 100.28 | 2282.37 (2009.91, 2578.00) | 2375.21 (2095.69, 2640.96) | 2285.81 (2004.09, 2565.97) | 2281.64 (2009.94, 2577.33) | -0.11 (-0.16, -0.06) | 0.35 (0.19, 0.51) | -0.39 (-0.55, -0.24) | 0.00 (-0.07, 0.08) |
| Chad | 73.95 (61.61, 88.12) | 205.12 (169.79, 246.64) | 177.38 | 1387.31 (1167.33, 1627.42) | 1400.02 (1176.03, 1640.56) | 1309.55 (1223.20, 1395.26) | 1394.37 (1171.85, 1638.66) | -0.07 (-0.19, 0.05) | 0.09 (0.06, 0.11) | -0.70 (-1.00, -0.39) | 0.78 (0.42, 1.13) |
| Chile | 177.38 (148.74, 208.66) | 221.74 (188.74, 259.26) | 25.01 | 1215.63 (1032.26, 1418.04) | 1208.62 (1023.40, 1407.40) | 1212.62 (1027.52, 1425.92) | 1209.25 (1026.57, 1418.06) | -0.01 (-0.02, 0.00) | -0.07 (-0.08, -0.06) | 0.03 (-0.02, 0.09) | -0.04 (-0.08, -0.01) |
| China | 9991.37 (8262.67, 11868.13) | 11703.77 (9848.45, 13923.68) | 17.14 | 761.40 (636.83, 905.81) | 753.89 (634.83, 890.19) | 671.83 (566.28, 795.80) | 761.69 (640.86, 908.90) | -0.44 (-0.67, -0.22) | -0.35 (-1.39, 0.70) | -1.17 (-1.75, -0.59) | 1.49 (0.86, 2.13) |
| Colombia | 710.68 (643.02, 785.80) | 861.10 (739.44, 1002.46) | 21.17 | 1950.57 (1773.88, 2140.08) | 1870.43 (1656.91, 2106.86) | 1726.74 (1477.65, 1992.40) | 1719.94 (1475.86, 2002.02) | -0.80 (-0.93, -0.66) | -0.59 (-1.52, 0.34) | -0.82 (-0.93, -0.70) | -0.03 (-0.05, -0.02) |
| Comoros | 9.27 (7.74, 11.07) | 15.47 (13.12, 18.14) | 66.98 | 1979.19 (1685.33, 2312.31) | 1999.42 (1707.56, 2332.76) | 1987.18 (1694.40, 2316.10) | 1975.74 (1684.10, 2297.41) | -0.03 (-0.04, -0.01) | 0.09 (0.04, 0.14) | -0.06 (-0.08, -0.05) | -0.06 (-0.08, -0.04) |
| Congo | 59.12 (51.05, 67.60) | 127.00 (111.18, 144.26) | 114.82 | 2275.92 (2005.51, 2554.97) | 2288.45 (2020.18, 2577.78) | 2288.04 (2007.34, 2582.33) | 2267.96 (1989.88, 2565.61) | 0.01 (-0.01, 0.02) | 0.06 (0.03, 0.10) | 0.00 (-0.03, 0.03) | -0.08 (-0.10, -0.06) |
| Cook Islands | 0.26 (0.22, 0.31) | 0.22 (0.18, 0.26) | -16.75 | 1337.23 (1122.15, 1576.28) | 1341.47 (1126.92, 1569.69) | 1346.88 (1135.88, 1584.85) | 1339.57 (1124.86, 1583.65) | 0.01 (0.00, 0.03) | 0.03 (0.02, 0.05) | 0.04 (0.03, 0.05) | -0.13 (-0.22, -0.04) |
| Costa Rica | 55.39 (50.45, 60.79) | 82.24 (70.30, 95.57) | 48.47 | 1693.17 (1543.57, 1852.42) | 1633.93 (1391.51, 1890.28) | 1639.75 (1394.80, 1905.43) | 1639.34 (1401.59, 1908.02) | -0.19 (-0.25, -0.12) | -0.44 (-0.80, -0.07) | 0.04 (0.03, 0.05) | 0.02 (-0.01, 0.05) |
| Croatia | 28.95 (24.23, 34.40) | 22.69 (19.22, 26.82) | -21.62 | 566.88 (474.96, 673.73) | 506.16 (429.47, 600.72) | 557.47 (479.03, 646.08) | 567.96 (480.22, 672.09) | 0.20 (0.02, 0.37) | -1.17 (-1.60, -0.74) | 1.00 (0.63, 1.38) | 0.10 (-0.61, 0.81) |
| Cuba | 200.95 (169.76, 237.21) | 165.64 (142.28, 191.65) | -17.57 | 1616.21 (1371.04, 1892.66) | 1616.59 (1370.40, 1879.36) | 1623.22 (1376.66, 1900.61) | 1607.43 (1359.12, 1870.77) | 0.00 (-0.02, 0.01) | 0.00 (-0.01, 0.02) | 0.04 (0.04, 0.05) | -0.07 (-0.16, 0.01) |
| Cyprus | 5.54 (4.63, 6.54) | 9.39 (7.85, 11.13) | 69.58 | 686.24 (575.40, 810.43) | 687.20 (573.15, 812.52) | 686.26 (575.55, 807.70) | 685.81 (574.50, 811.50) | 0.02 (0.01, 0.02) | 0.02 (0.00, 0.04) | -0.02 (-0.05, 0.01) | 0.01 (-0.04, 0.07) |
| Czechia | 39.82 (37.29, 42.23) | 47.65 (39.98, 56.82) | 19.68 | 388.12 (365.96, 410.02) | 469.04 (393.64, 559.40) | 468.98 (394.59, 554.58) | 467.80 (394.56, 554.50) | 0.26 (0.10, 0.42) | 1.65 (0.83, 2.48) | 0.00 (-0.01, 0.01) | -0.04 (-0.06, -0.02) |
| Côte d'Ivoire | 190.95 (159.27, 228.57) | 431.84 (364.24, 506.40) | 126.15 | 1566.60 (1322.79, 1835.36) | 1587.36 (1348.81, 1837.45) | 1580.42 (1335.79, 1845.23) | 1566.34 (1330.49, 1822.59) | -0.04 (-0.06, -0.02) | 0.11 (0.00, 0.21) | -0.04 (-0.09, 0.00) | -0.09 (-0.11, -0.08) |
| Democratic People's Republic of Korea | 165.72 (139.43, 197.15) | 213.42 (178.99, 251.43) | 28.79 | 771.83 (653.13, 918.71) | 770.76 (647.31, 914.09) | 758.40 (633.38, 893.71) | 752.28 (629.78, 884.49) | -0.11 (-0.12, -0.10) | -0.01 (-0.02, 0.00) | -0.17 (-0.18, -0.15) | -0.11 (-0.15, -0.08) |
| Democratic Republic of the Congo | 877.97 (759.57, 1008.44) | 2122.94 (1833.11, 2434.11) | 141.80 | 2250.45 (1971.52, 2539.94) | 2240.61 (1974.09, 2532.90) | 2230.02 (1965.96, 2520.69) | 2232.36 (1953.40, 2518.73) | -0.02 (-0.03, -0.01) | -0.04 (-0.06, -0.02) | -0.05 (-0.08, -0.02) | 0.04 (0.01, 0.07) |
| Denmark | 42.26 (35.51, 50.27) | 39.73 (33.87, 46.72) | -6.01 | 814.79 (681.42, 971.61) | 737.49 (680.64, 805.31) | 738.97 (679.18, 806.16) | 753.83 (635.39, 894.03) | -0.11 (-0.21, -0.01) | -0.96 (-1.17, -0.75) | 0.02 (0.01, 0.03) | 0.27 (0.10, 0.43) |
| Djibouti | 8.78 (7.29, 10.57) | 22.06 (18.68, 26.00) | 151.30 | 1681.68 (1418.86, 1978.48) | 1707.15 (1443.87, 2002.12) | 1709.29 (1443.04, 2005.70) | 1696.93 (1440.01, 1988.47) | 0.05 (0.03, 0.06) | 0.15 (0.12, 0.18) | 0.02 (0.01, 0.03) | -0.04 (-0.09, 0.01) |
| Dominica | 1.19 (1.00, 1.40) | 1.10 (0.94, 1.28) | -7.15 | 1607.59 (1367.56, 1873.87) | 1615.39 (1368.22, 1877.96) | 1629.94 (1380.68, 1901.89) | 1609.33 (1365.66, 1869.29) | 0.03 (0.02, 0.05) | 0.05 (0.02, 0.09) | 0.09 (0.08, 0.10) | -0.11 (-0.17, -0.05) |
| Dominican Republic | 126.94 (106.22, 150.67) | 189.76 (160.88, 222.43) | 49.49 | 1647.88 (1398.75, 1926.27) | 1639.07 (1387.94, 1909.67) | 1635.10 (1393.04, 1894.31) | 1622.93 (1385.97, 1896.76) | -0.06 (-0.07, -0.05) | -0.06 (-0.07, -0.04) | -0.02 (-0.04, 0.00) | -0.07 (-0.14, 0.00) |
| Ecuador | 187.30 (157.69, 221.75) | 328.20 (279.95, 382.12) | 75.23 | 1755.87 (1502.05, 2043.49) | 1759.87 (1506.57, 2039.66) | 1753.21 (1507.10, 2022.19) | 1745.89 (1493.09, 2019.64) | -0.02 (-0.03, -0.01) | 0.03 (0.01, 0.05) | -0.04 (-0.06, -0.02) | -0.05 (-0.05, -0.04) |
| Egypt | 434.19 (361.58, 519.33) | 822.60 (688.04, 984.13) | 89.45 | 793.90 (666.33, 938.78) | 793.22 (666.81, 938.29) | 795.58 (669.22, 947.68) | 792.00 (667.37, 941.72) | -0.01 (-0.02, -0.01) | -0.01 (-0.04, 0.01) | 0.03 (0.02, 0.04) | -0.06 (-0.09, -0.04) |
| El Salvador | 89.99 (75.02, 106.09) | 112.68 (95.05, 131.82) | 25.21 | 1676.76 (1426.34, 1929.76) | 1690.24 (1445.48, 1969.29) | 1688.93 (1440.57, 1953.96) | 1679.83 (1425.95, 1947.53) | 0.01 (0.00, 0.03) | 0.09 (0.06, 0.12) | -0.01 (-0.01, 0.00) | -0.04 (-0.07, -0.02) |
| Equatorial Guinea | 9.40 (8.16, 10.74) | 37.04 (31.99, 42.62) | 294.18 | 2303.24 (2027.87, 2589.85) | 2264.43 (2007.49, 2543.65) | 2178.12 (1917.25, 2451.68) | 2128.06 (1868.49, 2405.64) | -0.31 (-0.33, -0.29) | -0.16 (-0.17, -0.16) | -0.39 (-0.43, -0.35) | -0.26 (-0.31, -0.21) |
| Eritrea | 50.92 (43.75, 59.63) | 128.14 (107.30, 153.74) | 151.63 | 1684.25 (1457.48, 1931.01) | 1709.79 (1471.28, 1962.52) | 1719.37 (1453.44, 2011.58) | 1713.04 (1451.43, 2024.87) | 0.05 (0.03, 0.06) | 0.13 (0.08, 0.19) | 0.06 (0.00, 0.11) | -0.05 (-0.06, -0.03) |
| Estonia | 14.51 (12.25, 17.14) | 10.83 (9.17, 12.65) | -25.40 | 948.38 (799.64, 1121.68) | 989.10 (904.35, 1075.91) | 948.91 (797.44, 1115.37) | 947.51 (794.99, 1124.21) | -0.15 (-0.22, -0.09) | 0.37 (0.15, 0.58) | -0.41 (-0.60, -0.23) | -0.02 (-0.04, -0.01) |
| Eswatini | 17.22 (14.63, 20.23) | 31.03 (26.59, 35.96) | 80.17 | 2158.62 (1857.24, 2483.19) | 2295.52 (1989.19, 2635.03) | 2361.33 (2047.48, 2682.11) | 2349.80 (2034.90, 2689.52) | 0.32 (0.26, 0.39) | 0.63 (0.56, 0.71) | 0.28 (0.19, 0.36) | -0.05 (-0.06, -0.04) |
| Ethiopia | 747.29 (620.91, 895.49) | 1743.40 (1449.20, 2092.51) | 133.30 | 1574.21 (1320.95, 1848.66) | 2057.91 (1759.96, 2397.15) | 1263.38 (1077.47, 1464.92) | 1568.06 (1323.24, 1843.29) | -1.55 (-2.20, -0.90) | 2.32 (1.15, 3.51) | -5.06 (-7.00, -3.09) | 3.05 (1.60, 4.53) |
| Fiji | 11.00 (9.18, 13.11) | 12.64 (10.65, 14.92) | 14.90 | 1336.34 (1123.21, 1571.39) | 1341.83 (1127.27, 1570.45) | 1348.05 (1135.87, 1583.82) | 1341.67 (1128.50, 1586.35) | 0.02 (0.01, 0.03) | 0.04 (0.03, 0.05) | 0.05 (0.03, 0.06) | -0.10 (-0.17, -0.03) |
| Finland | 41.60 (36.95, 46.61) | 32.61 (27.64, 38.43) | -21.61 | 836.29 (747.82, 933.03) | 706.72 (655.24, 754.36) | 671.57 (566.72, 799.25) | 669.78 (563.97, 795.95) | -0.65 (-0.98, -0.31) | -1.60 (-1.87, -1.33) | -0.47 (-2.08, 1.17) | -0.03 (-0.08, 0.01) |
| France | 399.61 (336.75, 473.92) | 399.66 (339.78, 473.88) | 0.01 | 682.78 (572.91, 813.42) | 710.90 (669.10, 753.93) | 682.87 (573.10, 815.02) | 683.30 (574.22, 815.46) | -0.14 (-0.19, -0.08) | 0.38 (0.20, 0.55) | -0.41 (-0.59, -0.23) | 0.01 (0.00, 0.02) |
| Gabon | 23.34 (20.30, 26.78) | 45.64 (39.67, 52.01) | 95.59 | 2266.32 (1999.05, 2566.80) | 2499.29 (2279.78, 2714.11) | 2302.05 (2027.49, 2598.71) | 2305.69 (2016.06, 2603.99) | -0.23 (-0.37, -0.09) | 0.87 (0.47, 1.27) | -0.84 (-1.20, -0.47) | 0.04 (0.01, 0.06) |
| Gambia | 13.66 (11.35, 16.21) | 34.05 (28.49, 40.39) | 149.17 | 1410.19 (1198.09, 1642.67) | 1377.18 (1276.16, 1486.60) | 1431.08 (1213.55, 1655.88) | 1422.50 (1202.47, 1650.09) | 0.16 (0.12, 0.21) | -0.20 (-0.36, -0.04) | 0.40 (0.28, 0.52) | -0.06 (-0.09, -0.03) |
| Georgia | 39.09 (32.57, 46.33) | 24.19 (20.42, 28.60) | -38.12 | 691.16 (578.59, 822.51) | 695.96 (586.15, 825.47) | 691.16 (580.70, 822.76) | 686.62 (577.65, 815.69) | -0.03 (-0.04, -0.02) | 0.07 (0.07, 0.08) | -0.07 (-0.10, -0.04) | -0.08 (-0.10, -0.06) |
| Germany | 793.51 (655.30, 941.06) | 605.82 (509.87, 714.55) | -23.65 | 987.47 (814.69, 1174.08) | 903.66 (811.69, 1002.39) | 1030.94 (877.80, 1185.94) | 812.90 (681.34, 957.25) | -0.40 (-0.76, -0.03) | -0.78 (-1.18, -0.38) | 1.37 (0.80, 1.94) | -2.75 (-4.44, -1.03) |
| Ghana | 233.16 (195.31, 276.55) | 552.59 (463.37, 652.14) | 137.00 | 1576.16 (1337.31, 1832.24) | 1583.23 (1342.13, 1838.78) | 1595.53 (1352.72, 1853.56) | 1583.02 (1343.64, 1838.58) | 0.03 (0.02, 0.04) | 0.04 (0.00, 0.07) | 0.08 (0.06, 0.10) | -0.09 (-0.12, -0.05) |
| Greece | 61.06 (51.61, 72.22) | 53.74 (45.55, 63.79) | -11.99 | 584.70 (489.80, 695.01) | 551.22 (511.74, 591.57) | 550.94 (512.23, 592.83) | 585.06 (488.11, 692.51) | 0.01 (-0.12, 0.13) | -0.61 (-0.81, -0.40) | -0.01 (-0.03, 0.02) | 0.71 (0.35, 1.07) |
| Greenland | 0.57 (0.47, 0.69) | 0.48 (0.40, 0.58) | -15.26 | 859.73 (716.61, 1021.37) | 864.86 (723.00, 1033.67) | 867.86 (725.26, 1025.32) | 870.02 (727.57, 1041.05) | 0.04 (0.04, 0.05) | 0.06 (0.04, 0.09) | 0.04 (0.03, 0.05) | 0.02 (0.01, 0.03) |
| Grenada | 1.35 (1.14, 1.59) | 1.76 (1.49, 2.06) | 29.85 | 1618.44 (1370.98, 1887.07) | 1618.81 (1373.34, 1891.36) | 1625.63 (1380.06, 1904.40) | 1604.34 (1355.32, 1875.06) | -0.02 (-0.03, 0.00) | 0.00 (-0.01, 0.01) | 0.05 (0.04, 0.06) | -0.13 (-0.20, -0.06) |
| Guam | 2.14 (1.78, 2.55) | 2.20 (1.85, 2.59) | 2.77 | 1337.19 (1118.69, 1580.45) | 1344.02 (1127.06, 1573.92) | 1346.07 (1129.05, 1574.06) | 1340.69 (1128.68, 1583.21) | 0.02 (0.01, 0.03) | 0.06 (0.03, 0.09) | 0.01 (-0.01, 0.04) | -0.09 (-0.16, -0.03) |
| Guatemala | 124.85 (104.98, 147.30) | 332.92 (281.02, 392.35) | 166.66 | 1667.43 (1427.25, 1923.75) | 1684.69 (1436.30, 1959.56) | 1675.95 (1427.54, 1949.74) | 1661.97 (1422.05, 1929.25) | -0.01 (-0.03, 0.01) | 0.11 (0.08, 0.14) | -0.06 (-0.07, -0.04) | -0.08 (-0.10, -0.07) |
| Guinea | 89.09 (74.94, 105.38) | 200.24 (167.17, 236.80) | 124.75 | 1588.98 (1348.44, 1851.16) | 1599.54 (1355.93, 1873.06) | 1623.20 (1373.04, 1893.34) | 1609.32 (1366.81, 1869.72) | 0.08 (0.06, 0.09) | 0.05 (0.03, 0.08) | 0.15 (0.13, 0.17) | -0.10 (-0.12, -0.08) |
| Guinea-Bissau | 15.29 (12.78, 18.26) | 32.53 (27.34, 38.28) | 112.82 | 1584.82 (1344.05, 1851.14) | 1588.02 (1349.42, 1850.97) | 1602.39 (1372.21, 1867.07) | 1589.09 (1350.68, 1840.56) | 0.03 (0.02, 0.04) | 0.02 (0.01, 0.02) | 0.10 (0.07, 0.12) | -0.10 (-0.12, -0.07) |
| Guyana | 13.91 (11.66, 16.46) | 13.79 (11.66, 16.14) | -0.84 | 1634.51 (1396.27, 1902.90) | 1632.90 (1386.31, 1908.38) | 1647.84 (1409.78, 1919.70) | 1633.50 (1388.02, 1898.14) | 0.02 (0.00, 0.03) | -0.01 (-0.01, -0.01) | 0.10 (0.07, 0.13) | -0.08 (-0.13, -0.04) |
| Haiti | 89.89 (74.78, 106.86) | 195.86 (163.89, 232.33) | 117.90 | 1471.79 (1239.67, 1735.95) | 1470.91 (1235.13, 1733.47) | 1476.22 (1236.31, 1736.26) | 1457.33 (1231.88, 1717.02) | -0.04 (-0.05, -0.02) | -0.01 (-0.05, 0.03) | 0.04 (0.03, 0.04) | -0.11 (-0.20, -0.02) |
| Honduras | 90.57 (76.24, 106.79) | 219.08 (186.62, 253.95) | 141.88 | 1994.53 (1715.32, 2299.13) | 1991.14 (1716.78, 2292.01) | 2481.39 (2225.51, 2743.32) | 1996.56 (1721.42, 2290.46) | 0.32 (-0.08, 0.72) | -0.02 (-0.05, 0.02) | 2.27 (1.31, 3.24) | -2.59 (-3.58, -1.58) |
| Hungary | 43.67 (36.55, 52.28) | 38.95 (32.72, 46.47) | -10.80 | 412.07 (344.29, 492.27) | 328.78 (294.44, 366.30) | 412.24 (343.41, 490.89) | 410.77 (344.94, 489.64) | 0.76 (0.44, 1.07) | -2.00 (-2.95, -1.05) | 2.33 (1.34, 3.33) | -0.04 (-0.05, -0.02) |
| Iceland | 1.92 (1.60, 2.28) | 2.41 (2.02, 2.83) | 25.17 | 723.78 (605.25, 859.61) | 837.86 (774.97, 905.04) | 831.13 (767.57, 901.59) | 721.65 (601.98, 852.10) | 0.03 (-0.26, 0.31) | 1.54 (0.97, 2.11) | -0.11 (-0.14, -0.08) | -1.64 (-2.24, -1.03) |
| India | 4867.86 (4045.92, 5853.63) | 9777.43 (8204.95, 11672.39) | 100.86 | 602.41 (505.25, 720.10) | 648.74 (547.37, 769.72) | 666.87 (562.20, 789.83) | 651.70 (548.25, 774.61) | 0.33 (0.24, 0.43) | 0.77 (0.49, 1.05) | 0.28 (0.21, 0.35) | -0.27 (-0.37, -0.18) |
| Indonesia | 1994.81 (1660.66, 2362.17) | 2937.13 (2475.77, 3441.51) | 47.24 | 1038.23 (868.50, 1217.13) | 1031.33 (861.37, 1217.56) | 1036.13 (868.95, 1224.55) | 1037.63 (873.76, 1214.02) | -0.01 (-0.02, 0.00) | -0.07 (-0.10, -0.04) | 0.05 (0.02, 0.08) | -0.04 (-0.10, 0.02) |
| Iran (Islamic Republic of) | 525.29 (436.35, 626.76) | 910.84 (767.82, 1090.88) | 73.40 | 963.10 (809.63, 1128.97) | 954.96 (808.16, 1125.37) | 952.55 (806.04, 1115.30) | 961.48 (816.43, 1130.07) | -0.02 (-0.04, 0.00) | -0.10 (-0.19, -0.02) | -0.02 (-0.08, 0.03) | 0.11 (0.04, 0.18) |
| Iraq | 128.27 (106.35, 154.66) | 367.32 (304.76, 443.22) | 186.36 | 794.17 (666.56, 939.47) | 787.73 (661.90, 932.31) | 792.59 (666.78, 944.44) | 791.66 (667.46, 940.88) | 0.00 (-0.01, 0.01) | -0.07 (-0.09, -0.06) | 0.08 (0.06, 0.10) | -0.01 (-0.04, 0.02) |
| Ireland | 24.66 (20.70, 29.09) | 31.96 (26.97, 38.26) | 29.59 | 689.15 (578.30, 814.32) | 690.19 (576.41, 816.12) | 689.56 (578.68, 816.68) | 689.26 (575.22, 825.49) | 0.02 (0.01, 0.03) | 0.02 (0.01, 0.03) | -0.02 (-0.06, 0.02) | 0.02 (-0.04, 0.08) |
| Israel | 32.78 (27.34, 38.93) | 57.10 (48.23, 68.13) | 74.16 | 670.82 (559.73, 791.28) | 672.27 (571.43, 786.25) | 655.03 (545.32, 777.26) | 650.93 (548.13, 778.48) | -0.20 (-0.24, -0.16) | -0.04 (-0.29, 0.21) | -0.27 (-0.30, -0.23) | -0.02 (-0.08, 0.04) |
| Italy | 395.01 (335.12, 465.47) | 359.09 (304.69, 422.65) | -9.09 | 670.62 (568.73, 793.39) | 673.51 (568.61, 794.81) | 678.23 (572.09, 801.54) | 675.98 (572.76, 799.33) | 0.04 (0.03, 0.05) | 0.05 (0.03, 0.06) | 0.07 (0.04, 0.10) | -0.01 (-0.05, 0.03) |
| Jamaica | 51.41 (43.84, 58.28) | 56.35 (47.92, 64.99) | 9.62 | 2000.29 (1738.80, 2243.87) | 2009.30 (1756.69, 2251.70) | 1820.58 (1551.19, 2090.43) | 1803.69 (1542.58, 2073.23) | -0.49 (-0.57, -0.40) | 0.06 (0.02, 0.09) | -1.02 (-1.43, -0.60) | -0.09 (-0.14, -0.04) |
| Japan | 741.73 (622.21, 884.76) | 633.45 (531.87, 755.73) | -14.60 | 563.15 (473.96, 672.43) | 560.97 (468.10, 670.32) | 579.75 (487.85, 692.71) | 577.93 (482.43, 688.91) | 0.07 (0.01, 0.13) | -0.08 (-0.31, 0.14) | 0.34 (0.15, 0.54) | -0.04 (-0.06, -0.03) |
| Jordan | 34.08 (28.20, 41.34) | 115.56 (96.69, 137.43) | 239.03 | 904.92 (763.30, 1073.67) | 922.86 (775.56, 1085.58) | 909.91 (767.32, 1084.10) | 905.56 (759.79, 1070.14) | -0.08 (-0.10, -0.05) | 0.18 (0.09, 0.26) | -0.16 (-0.24, -0.08) | -0.10 (-0.19, -0.02) |
| Kazakhstan | 116.82 (96.73, 138.56) | 133.30 (111.71, 159.71) | 14.11 | 688.22 (573.73, 810.48) | 692.34 (582.55, 824.71) | 693.19 (582.17, 827.23) | 690.45 (578.58, 828.79) | 0.01 (0.00, 0.02) | 0.06 (0.05, 0.07) | 0.01 (-0.03, 0.05) | -0.06 (-0.09, -0.04) |
| Kenya | 478.01 (400.84, 562.83) | 1161.69 (984.30, 1359.48) | 143.03 | 2076.41 (1780.13, 2393.55) | 2492.34 (2163.95, 2851.87) | 2128.05 (1829.23, 2451.01) | 2070.80 (1775.72, 2390.82) | -0.63 (-0.87, -0.40) | 1.60 (0.71, 2.51) | -1.68 (-2.00, -1.36) | -0.29 (-0.38, -0.20) |
| Kiribati | 1.03 (0.86, 1.24) | 1.68 (1.40, 2.00) | 63.35 | 1334.94 (1120.24, 1571.68) | 1340.12 (1124.20, 1571.94) | 1346.79 (1133.57, 1581.14) | 1339.24 (1123.75, 1577.25) | 0.02 (0.00, 0.04) | 0.04 (0.03, 0.05) | 0.05 (0.05, 0.05) | -0.13 (-0.22, -0.03) |
| Kuwait | 15.71 (12.87, 19.01) | 42.84 (35.57, 52.39) | 172.68 | 761.17 (640.34, 907.52) | 749.60 (626.96, 890.58) | 765.23 (641.80, 907.11) | 787.35 (661.98, 937.48) | 0.12 (0.07, 0.17) | -0.18 (-0.25, -0.12) | 0.21 (0.16, 0.26) | 0.31 (0.29, 0.33) |
| Kyrgyzstan | 29.44 (24.42, 35.05) | 47.21 (39.25, 56.37) | 60.39 | 688.47 (573.97, 811.15) | 690.71 (579.22, 820.25) | 691.12 (581.82, 823.23) | 689.66 (580.06, 818.93) | 0.00 (-0.01, 0.01) | 0.03 (0.02, 0.04) | 0.01 (-0.04, 0.06) | -0.05 (-0.08, -0.02) |
| Lao People's Democratic Republic | 44.87 (37.27, 53.70) | 91.25 (76.18, 108.83) | 103.35 | 1148.66 (969.25, 1354.02) | 1143.71 (961.27, 1354.95) | 1142.84 (956.13, 1354.33) | 1144.44 (966.57, 1352.93) | -0.03 (-0.04, -0.02) | -0.05 (-0.06, -0.03) | -0.01 (-0.04, 0.03) | -0.02 (-0.07, 0.02) |
| Latvia | 24.59 (20.81, 28.98) | 15.32 (13.02, 18.10) | -37.73 | 951.37 (801.12, 1120.43) | 949.28 (793.76, 1112.74) | 949.54 (799.31, 1127.08) | 947.52 (797.97, 1116.58) | -0.01 (-0.01, -0.01) | -0.02 (-0.04, -0.01) | 0.00 (0.00, 0.01) | -0.02 (-0.02, -0.01) |
| Lebanon | 24.74 (20.84, 29.36) | 42.36 (35.30, 50.79) | 71.21 | 799.49 (672.32, 948.95) | 797.00 (669.92, 945.56) | 803.40 (676.91, 958.68) | 795.48 (668.22, 950.13) | -0.02 (-0.04, 0.00) | -0.04 (-0.05, -0.03) | 0.09 (0.07, 0.10) | -0.10 (-0.19, -0.01) |
| Lesotho | 39.09 (33.28, 45.72) | 57.07 (49.10, 65.84) | 46.01 | 2140.91 (1845.56, 2459.23) | 2267.43 (1963.89, 2600.38) | 2307.42 (2001.62, 2641.60) | 2316.65 (2014.35, 2652.08) | 0.26 (0.21, 0.30) | 0.59 (0.57, 0.61) | 0.17 (0.11, 0.22) | 0.03 (0.01, 0.05) |
| Liberia | 27.77 (23.37, 32.62) | 80.75 (68.50, 95.04) | 190.81 | 1602.83 (1359.42, 1868.76) | 1577.24 (1334.07, 1841.16) | 1579.24 (1352.21, 1852.68) | 1571.29 (1336.69, 1831.18) | -0.05 (-0.07, -0.04) | -0.17 (-0.23, -0.11) | 0.00 (-0.03, 0.04) | -0.04 (-0.07, -0.01) |
| Libya | 31.34 (25.93, 37.96) | 62.69 (52.46, 74.85) | 100.02 | 777.63 (654.45, 925.36) | 790.20 (660.77, 940.45) | 787.60 (658.92, 935.72) | 790.41 (663.91, 941.09) | 0.01 (0.00, 0.03) | 0.17 (0.09, 0.24) | -0.04 (-0.05, -0.03) | 0.05 (0.03, 0.07) |
| Lithuania | 35.33 (29.87, 41.96) | 22.92 (19.40, 26.84) | -35.13 | 951.08 (804.16, 1126.52) | 950.79 (793.27, 1130.22) | 950.75 (801.48, 1133.01) | 946.89 (799.47, 1120.05) | -0.01 (-0.01, 0.00) | 0.00 (-0.01, 0.01) | 0.00 (0.00, 0.00) | -0.04 (-0.05, -0.04) |
| Luxembourg | 2.76 (2.31, 3.29) | 4.23 (3.56, 5.00) | 53.41 | 689.09 (578.34, 819.52) | 689.67 (578.62, 817.79) | 687.34 (574.83, 813.87) | 684.77 (575.14, 810.47) | -0.01 (-0.02, 0.00) | 0.02 (0.00, 0.03) | -0.04 (-0.07, 0.00) | -0.01 (-0.05, 0.03) |
| Madagascar | 239.45 (200.36, 285.19) | 575.99 (485.36, 684.43) | 140.54 | 1980.12 (1685.61, 2313.56) | 2001.91 (1710.68, 2320.77) | 1992.45 (1699.73, 2311.97) | 1979.84 (1684.19, 2316.17) | -0.02 (-0.03, -0.01) | 0.10 (0.04, 0.15) | -0.05 (-0.06, -0.03) | -0.07 (-0.09, -0.04) |
| Malawi | 242.08 (209.20, 270.31) | 478.00 (405.76, 561.12) | 97.45 | 2386.75 (2106.03, 2639.78) | 2861.41 (2673.90, 3068.57) | 2440.02 (2148.46, 2723.40) | 2297.28 (1980.24, 2639.12) | -0.67 (-0.94, -0.40) | 1.64 (0.96, 2.32) | -1.61 (-1.96, -1.26) | -0.63 (-0.85, -0.40) |
| Malaysia | 212.91 (177.89, 254.54) | 402.66 (335.08, 476.86) | 89.12 | 1142.04 (962.19, 1352.92) | 1138.12 (960.03, 1337.88) | 1137.27 (954.55, 1343.29) | 1137.55 (952.21, 1339.22) | -0.02 (-0.02, -0.01) | -0.04 (-0.04, -0.03) | -0.01 (-0.03, 0.02) | -0.01 (-0.03, 0.01) |
| Maldives | 2.33 (1.92, 2.81) | 7.09 (5.86, 8.57) | 204.23 | 1143.12 (964.68, 1347.63) | 1140.02 (958.89, 1343.44) | 1129.28 (946.14, 1328.86) | 1111.92 (942.82, 1302.66) | -0.12 (-0.14, -0.10) | -0.04 (-0.08, 0.00) | -0.09 (-0.14, -0.03) | -0.20 (-0.25, -0.15) |
| Mali | 159.66 (147.13, 173.35) | 376.64 (316.65, 437.82) | 135.90 | 1996.95 (1841.10, 2167.68) | 1987.68 (1834.87, 2163.30) | 1771.05 (1512.75, 2042.50) | 1754.01 (1502.71, 2024.46) | -0.59 (-0.70, -0.48) | -0.06 (-0.08, -0.04) | -1.19 (-1.76, -0.62) | -0.11 (-0.15, -0.07) |
| Malta | 2.60 (2.18, 3.10) | 2.74 (2.31, 3.26) | 5.34 | 688.91 (577.08, 820.55) | 688.09 (578.38, 817.69) | 684.62 (574.53, 808.07) | 684.68 (574.96, 816.33) | -0.02 (-0.02, -0.01) | -0.01 (-0.02, 0.01) | -0.05 (-0.08, -0.03) | 0.03 (0.00, 0.06) |
| Marshall Islands | 0.58 (0.48, 0.70) | 0.82 (0.69, 0.97) | 42.21 | 1336.51 (1120.55, 1581.80) | 1343.42 (1126.92, 1572.93) | 1348.73 (1135.92, 1589.85) | 1342.38 (1131.76, 1574.71) | 0.02 (0.00, 0.03) | 0.05 (0.04, 0.06) | 0.04 (0.04, 0.04) | -0.11 (-0.17, -0.04) |
| Mauritania | 30.91 (25.89, 36.65) | 65.54 (55.31, 77.51) | 112.03 | 1566.41 (1334.10, 1819.63) | 1579.55 (1336.05, 1834.71) | 1587.11 (1344.73, 1835.94) | 1576.57 (1337.29, 1837.36) | 0.03 (0.02, 0.04) | 0.08 (0.06, 0.10) | 0.05 (0.03, 0.07) | -0.06 (-0.08, -0.04) |
| Mauritius | 14.35 (11.94, 17.12) | 14.76 (12.48, 17.32) | 2.81 | 1139.77 (961.71, 1343.70) | 1138.80 (955.99, 1338.47) | 1141.53 (955.20, 1344.86) | 1143.84 (957.48, 1347.78) | 0.01 (0.00, 0.01) | -0.01 (-0.03, 0.01) | 0.02 (0.01, 0.04) | 0.00 (-0.05, 0.04) |
| Mexico | 1131.38 (950.20, 1341.89) | 1709.11 (1450.19, 1997.00) | 51.06 | 1290.22 (1097.23, 1505.57) | 1089.09 (923.67, 1274.72) | 1245.11 (1059.23, 1450.61) | 1290.27 (1094.20, 1507.46) | 0.53 (0.27, 0.80) | -1.52 (-2.25, -0.79) | 1.38 (0.75, 2.02) | 0.43 (0.24, 0.61) |
| Micronesia (Federated States of) | 1.34 (1.12, 1.62) | 1.47 (1.23, 1.75) | 9.72 | 1336.13 (1122.96, 1577.72) | 1341.27 (1126.71, 1590.54) | 1348.49 (1133.00, 1581.84) | 1344.86 (1120.02, 1586.61) | 0.04 (0.02, 0.05) | 0.04 (0.02, 0.06) | 0.05 (0.05, 0.06) | -0.08 (-0.17, 0.00) |
| Monaco | 0.19 (0.16, 0.23) | 0.21 (0.18, 0.24) | 9.15 | 690.54 (580.04, 821.89) | 688.27 (576.99, 815.41) | 687.95 (576.36, 815.95) | 686.99 (576.92, 812.67) | 0.00 (0.00, 0.01) | -0.03 (-0.05, 0.00) | -0.01 (-0.03, 0.02) | 0.01 (-0.03, 0.04) |
| Mongolia | 13.88 (11.37, 16.69) | 25.69 (21.41, 30.80) | 85.11 | 687.61 (575.08, 818.34) | 692.29 (583.42, 821.40) | 691.47 (584.45, 815.79) | 690.40 (580.25, 819.96) | 0.01 (0.00, 0.01) | 0.07 (0.07, 0.08) | -0.01 (-0.06, 0.03) | -0.04 (-0.06, -0.02) |
| Montenegro | 3.39 (2.85, 4.04) | 3.23 (2.72, 3.82) | -4.96 | 518.62 (433.45, 618.12) | 519.92 (437.02, 622.21) | 521.01 (436.79, 618.09) | 518.70 (436.32, 614.30) | 0.00 (-0.01, 0.01) | 0.02 (0.01, 0.03) | 0.02 (0.01, 0.03) | -0.07 (-0.09, -0.05) |
| Morocco | 289.16 (270.24, 313.31) | 331.47 (281.49, 393.77) | 14.63 | 1076.55 (998.82, 1173.54) | 962.30 (865.38, 1060.13) | 867.05 (731.61, 1023.43) | 862.80 (732.85, 1024.08) | -0.68 (-0.76, -0.59) | -0.98 (-1.48, -0.47) | -1.13 (-1.22, -1.04) | -0.07 (-0.09, -0.05) |
| Mozambique | 275.86 (235.10, 325.20) | 696.99 (595.87, 822.36) | 152.66 | 2178.56 (1881.45, 2506.86) | 2247.61 (1950.16, 2564.90) | 2236.49 (1937.15, 2556.98) | 2243.06 (1951.62, 2584.10) | 0.05 (0.02, 0.07) | 0.26 (0.16, 0.36) | -0.05 (-0.10, -0.01) | 0.04 (0.00, 0.08) |
| Myanmar | 483.09 (402.45, 577.80) | 667.49 (561.66, 788.66) | 38.17 | 1143.55 (963.57, 1354.79) | 1144.75 (960.82, 1343.43) | 1147.70 (964.33, 1349.77) | 1151.79 (968.98, 1361.23) | 0.01 (0.00, 0.02) | 0.00 (-0.03, 0.03) | 0.03 (0.01, 0.04) | 0.01 (-0.03, 0.04) |
| Namibia | 31.31 (26.54, 36.83) | 60.29 (51.60, 70.38) | 92.54 | 2135.75 (1839.74, 2462.19) | 2225.10 (1927.05, 2551.74) | 2248.53 (1941.50, 2575.64) | 2248.49 (1935.52, 2592.96) | 0.16 (0.12, 0.20) | 0.42 (0.41, 0.43) | 0.10 (0.07, 0.13) | -0.01 (-0.02, 0.00) |
| Nauru | 0.14 (0.11, 0.16) | 0.16 (0.13, 0.18) | 13.14 | 1337.02 (1121.42, 1576.42) | 1341.49 (1127.11, 1570.44) | 1348.68 (1138.23, 1588.11) | 1340.41 (1124.63, 1578.10) | 0.02 (0.00, 0.03) | 0.04 (0.02, 0.05) | 0.06 (0.05, 0.06) | -0.13 (-0.22, -0.05) |
| Nepal | 99.82 (82.93, 118.28) | 183.57 (153.00, 218.08) | 83.89 | 573.98 (483.54, 677.35) | 574.07 (483.05, 684.61) | 577.47 (484.32, 682.01) | 580.17 (487.36, 688.15) | 0.03 (0.02, 0.04) | -0.01 (-0.05, 0.03) | 0.06 (0.06, 0.07) | 0.04 (0.01, 0.08) |
| Netherlands | 132.24 (111.40, 157.30) | 126.99 (107.97, 149.93) | -3.97 | 827.89 (693.93, 983.66) | 835.51 (714.24, 972.44) | 783.43 (662.40, 921.30) | 827.21 (694.40, 983.28) | 0.30 (0.11, 0.48) | 0.41 (-0.87, 1.72) | -0.68 (-0.91, -0.45) | 0.66 (0.38, 0.95) |
| New Zealand | 22.44 (18.63, 26.68) | 25.45 (21.51, 29.92) | 13.45 | 623.78 (519.59, 741.05) | 649.57 (546.26, 769.01) | 628.77 (523.41, 740.31) | 629.02 (526.55, 743.75) | -0.04 (-0.09, 0.01) | 0.42 (0.40, 0.44) | -0.35 (-0.44, -0.27) | 0.01 (-0.03, 0.06) |
| Nicaragua | 62.99 (52.51, 75.18) | 118.93 (100.44, 139.38) | 88.81 | 1672.27 (1427.40, 1944.64) | 1666.25 (1423.27, 1933.23) | 1658.31 (1413.60, 1939.24) | 1650.03 (1401.22, 1921.12) | -0.04 (-0.05, -0.04) | -0.03 (-0.04, -0.02) | -0.05 (-0.06, -0.04) | -0.04 (-0.06, -0.03) |
| Niger | 114.65 (96.01, 137.08) | 330.99 (276.32, 395.30) | 188.70 | 1577.44 (1335.01, 1846.21) | 1578.63 (1332.61, 1827.29) | 1585.53 (1341.80, 1840.69) | 1570.92 (1335.51, 1843.86) | -0.01 (-0.02, 0.00) | 0.00 (-0.04, 0.03) | 0.05 (0.04, 0.05) | -0.11 (-0.13, -0.10) |
| Nigeria | 1435.00 (1210.39, 1684.29) | 3592.63 (3037.08, 4223.83) | 150.36 | 1645.80 (1405.78, 1909.16) | 1697.73 (1455.39, 1957.62) | 1672.07 (1432.37, 1933.13) | 1679.17 (1439.83, 1938.98) | -0.01 (-0.04, 0.02) | 0.27 (0.15, 0.39) | -0.16 (-0.19, -0.14) | 0.05 (0.02, 0.08) |
| Niue | 0.03 (0.02, 0.03) | 0.02 (0.02, 0.02) | -27.32 | 1336.47 (1121.70, 1575.07) | 1343.20 (1128.30, 1571.22) | 1348.93 (1137.99, 1588.88) | 1339.32 (1124.09, 1576.63) | 0.01 (0.00, 0.03) | 0.05 (0.04, 0.06) | 0.04 (0.03, 0.06) | -0.14 (-0.22, -0.06) |
| North Macedonia | 11.02 (9.20, 13.13) | 11.80 (9.89, 14.13) | 7.04 | 519.11 (433.66, 618.62) | 519.29 (438.07, 621.54) | 518.79 (434.89, 613.76) | 515.64 (430.44, 615.82) | -0.02 (-0.03, -0.02) | -0.02 (-0.06, 0.01) | 0.00 (-0.03, 0.02) | -0.08 (-0.09, -0.06) |
| Northern Mariana Islands | 0.78 (0.65, 0.93) | 0.54 (0.46, 0.63) | -31.51 | 1338.26 (1121.86, 1578.15) | 1339.71 (1124.90, 1571.60) | 1343.39 (1125.14, 1568.95) | 1339.50 (1121.92, 1583.31) | 0.01 (0.00, 0.03) | 0.03 (0.01, 0.05) | 0.03 (0.02, 0.05) | -0.11 (-0.21, 0.00) |
| Norway | 30.60 (25.75, 36.00) | 34.66 (29.28, 40.99) | 13.26 | 712.72 (598.94, 840.02) | 707.53 (595.65, 841.75) | 712.98 (600.53, 847.32) | 674.37 (565.49, 803.85) | -0.18 (-0.23, -0.13) | -0.07 (-0.13, -0.02) | 0.08 (0.04, 0.12) | -0.64 (-0.85, -0.43) |
| Oman | 14.23 (11.74, 17.23) | 42.61 (34.37, 52.21) | 199.41 | 736.27 (619.30, 877.28) | 759.05 (638.30, 901.78) | 752.09 (633.79, 903.89) | 725.28 (604.00, 861.66) | -0.10 (-0.19, -0.02) | 0.30 (0.26, 0.33) | -0.08 (-0.23, 0.08) | -0.32 (-0.57, -0.08) |
| Pakistan | 454.21 (368.68, 555.76) | 1047.09 (854.32, 1292.61) | 130.53 | 486.67 (400.28, 597.63) | 489.07 (401.12, 599.35) | 479.24 (395.36, 580.73) | 495.59 (409.45, 607.51) | 0.01 (-0.04, 0.06) | 0.04 (0.00, 0.09) | -0.21 (-0.30, -0.12) | 0.40 (0.29, 0.51) |
| Palau | 0.24 (0.20, 0.28) | 0.23 (0.20, 0.27) | -0.41 | 1337.81 (1123.03, 1576.44) | 1342.15 (1125.71, 1574.74) | 1349.50 (1137.00, 1590.22) | 1345.00 (1126.23, 1588.18) | 0.02 (0.01, 0.03) | 0.04 (0.02, 0.05) | 0.06 (0.04, 0.07) | -0.10 (-0.17, -0.02) |
| Palestine | 14.73 (12.22, 17.84) | 41.20 (34.11, 49.82) | 179.65 | 798.21 (671.05, 942.56) | 790.45 (661.30, 941.29) | 792.46 (664.02, 940.39) | 794.00 (669.67, 943.77) | -0.02 (-0.02, -0.01) | -0.10 (-0.12, -0.09) | 0.03 (0.01, 0.05) | 0.01 (0.00, 0.02) |
| Panama | 42.64 (35.69, 50.12) | 69.50 (59.40, 80.76) | 62.98 | 1650.78 (1402.65, 1920.37) | 1651.68 (1404.09, 1922.18) | 1648.52 (1406.09, 1906.34) | 1644.31 (1404.61, 1910.51) | 0.00 (-0.01, 0.00) | 0.01 (-0.02, 0.04) | -0.02 (-0.03, -0.01) | -0.02 (-0.03, -0.01) |
| Papua New Guinea | 60.91 (51.17, 72.72) | 153.01 (128.70, 180.67) | 151.22 | 1442.24 (1219.58, 1703.55) | 1589.37 (1446.02, 1738.90) | 1456.49 (1230.66, 1706.90) | 1454.88 (1227.28, 1703.00) | -0.08 (-0.28, 0.12) | 0.95 (0.69, 1.22) | -0.94 (-1.59, -0.29) | -0.05 (-0.10, 0.00) |
| Paraguay | 75.99 (64.37, 89.62) | 145.19 (123.08, 169.90) | 91.05 | 1898.81 (1628.45, 2215.09) | 1905.09 (1623.61, 2210.70) | 1894.24 (1615.77, 2190.08) | 1895.93 (1613.02, 2202.04) | -0.01 (-0.02, 0.00) | 0.04 (0.02, 0.06) | -0.06 (-0.06, -0.06) | 0.01 (0.01, 0.02) |
| Peru | 405.70 (360.99, 457.13) | 621.34 (530.96, 719.81) | 53.15 | 1734.75 (1550.96, 1950.38) | 1851.85 (1672.83, 2049.33) | 1748.71 (1497.67, 2023.91) | 1737.64 (1487.37, 2011.27) | -0.19 (-0.31, -0.07) | 0.58 (0.29, 0.88) | -0.60 (-1.04, -0.15) | -0.06 (-0.08, -0.04) |
| Philippines | 669.67 (555.80, 801.83) | 1263.73 (1051.15, 1503.02) | 88.71 | 1059.70 (886.80, 1245.69) | 1081.38 (901.96, 1281.69) | 1071.07 (898.02, 1259.97) | 1073.54 (901.21, 1272.06) | 0.02 (-0.01, 0.04) | 0.21 (0.19, 0.22) | -0.10 (-0.13, -0.08) | 0.01 (0.00, 0.03) |
| Poland | 181.67 (150.03, 222.54) | 180.63 (149.99, 218.82) | -0.57 | 459.38 (378.90, 555.97) | 438.07 (363.51, 530.48) | 454.31 (378.07, 548.66) | 454.95 (376.40, 550.89) | 0.00 (-0.09, 0.09) | -0.50 (-0.68, -0.32) | 0.38 (0.17, 0.59) | -0.03 (-0.10, 0.04) |
| Portugal | 70.79 (59.48, 84.28) | 65.36 (55.21, 77.95) | -7.68 | 691.55 (577.38, 823.17) | 691.90 (581.36, 822.75) | 692.39 (577.80, 828.55) | 690.84 (577.51, 822.29) | 0.01 (0.01, 0.02) | 0.01 (-0.01, 0.03) | 0.01 (-0.03, 0.04) | 0.00 (-0.03, 0.04) |
| Puerto Rico | 60.72 (51.29, 70.82) | 50.53 (43.38, 58.54) | -16.78 | 1639.43 (1389.60, 1909.39) | 1632.10 (1382.00, 1903.27) | 1639.90 (1391.56, 1918.53) | 1628.31 (1375.33, 1900.77) | -0.02 (-0.03, 0.00) | -0.04 (-0.06, -0.03) | 0.05 (0.04, 0.07) | -0.06 (-0.15, 0.03) |
| Qatar | 3.87 (3.13, 4.72) | 26.98 (21.94, 33.39) | 596.44 | 706.55 (593.96, 841.89) | 724.27 (604.52, 863.23) | 681.59 (572.60, 806.86) | 677.44 (568.57, 806.03) | -0.25 (-0.32, -0.18) | 0.25 (0.24, 0.27) | -0.67 (-0.82, -0.52) | -0.06 (-0.11, 0.00) |
| Republic of Korea | 689.25 (649.86, 727.63) | 454.04 (388.21, 537.86) | -34.13 | 1293.27 (1220.03, 1369.25) | 959.13 (812.62, 1116.09) | 888.48 (757.60, 1036.63) | 874.57 (734.58, 1033.17) | -0.88 (-1.10, -0.66) | -2.70 (-3.85, -1.55) | -0.77 (-1.19, -0.34) | -0.19 (-0.21, -0.17) |
| Republic of Moldova | 43.10 (36.36, 51.17) | 34.20 (28.97, 40.31) | -20.65 | 956.09 (805.32, 1131.22) | 947.22 (792.47, 1114.18) | 950.17 (805.11, 1120.12) | 947.86 (796.10, 1125.53) | -0.01 (-0.02, 0.00) | -0.09 (-0.11, -0.08) | 0.03 (0.03, 0.04) | -0.03 (-0.04, -0.03) |
| Romania | 122.47 (103.60, 145.78) | 94.86 (80.08, 112.77) | -22.54 | 520.68 (436.08, 620.99) | 519.26 (434.36, 619.06) | 519.63 (435.38, 620.48) | 518.50 (437.16, 613.94) | -0.02 (-0.03, -0.02) | -0.03 (-0.04, -0.02) | 0.01 (0.00, 0.01) | -0.05 (-0.07, -0.02) |
| Russian Federation | 1451.38 (1207.69, 1727.98) | 1293.92 (1094.14, 1528.55) | -10.85 | 932.77 (783.84, 1102.23) | 930.39 (782.93, 1097.56) | 933.54 (789.59, 1098.64) | 935.52 (788.14, 1100.26) | 0.01 (0.01, 0.02) | -0.02 (-0.03, -0.01) | 0.04 (0.03, 0.04) | 0.01 (-0.01, 0.03) |
| Rwanda | 140.72 (117.47, 166.61) | 277.67 (234.55, 327.56) | 97.32 | 1957.86 (1660.56, 2292.84) | 2013.64 (1711.28, 2360.97) | 2098.81 (1923.49, 2277.80) | 1963.40 (1665.01, 2290.35) | 0.04 (-0.06, 0.15) | 0.28 (0.19, 0.36) | 0.43 (0.27, 0.59) | -0.81 (-1.01, -0.61) |
| Saint Kitts and Nevis | 0.70 (0.58, 0.82) | 1.00 (0.86, 1.17) | 43.68 | 1626.70 (1380.32, 1894.41) | 1626.14 (1385.91, 1893.39) | 1634.49 (1390.59, 1904.09) | 1629.64 (1391.62, 1904.52) | 0.02 (0.01, 0.03) | 0.00 (-0.03, 0.03) | 0.05 (0.03, 0.07) | -0.01 (-0.09, 0.08) |
| Saint Lucia | 2.31 (1.94, 2.72) | 2.96 (2.53, 3.44) | 28.27 | 1631.54 (1392.79, 1900.19) | 1633.17 (1392.70, 1904.19) | 1633.05 (1390.36, 1906.12) | 1620.22 (1370.97, 1890.42) | -0.03 (-0.04, -0.02) | 0.01 (0.01, 0.01) | 0.00 (-0.01, 0.01) | -0.07 (-0.13, -0.01) |
| Saint Vincent and the Grenadines | 1.86 (1.55, 2.21) | 1.82 (1.56, 2.12) | -2.02 | 1620.15 (1374.76, 1889.10) | 1617.66 (1365.62, 1889.61) | 1628.15 (1385.00, 1899.14) | 1613.80 (1364.78, 1881.92) | 0.01 (0.00, 0.02) | -0.01 (-0.04, 0.02) | 0.07 (0.05, 0.09) | -0.06 (-0.15, 0.03) |
| Samoa | 2.24 (1.85, 2.70) | 2.89 (2.42, 3.42) | 29.01 | 1336.54 (1118.74, 1579.03) | 1343.56 (1126.84, 1571.99) | 1348.48 (1135.27, 1589.38) | 1341.19 (1130.19, 1573.68) | 0.01 (0.00, 0.03) | 0.05 (0.05, 0.06) | 0.04 (0.03, 0.04) | -0.12 (-0.20, -0.05) |
| San Marino | 0.17 (0.14, 0.20) | 0.21 (0.18, 0.25) | 27.30 | 688.79 (578.61, 817.46) | 697.40 (583.78, 825.41) | 695.73 (583.31, 820.99) | 693.57 (582.62, 821.28) | 0.03 (0.02, 0.04) | 0.13 (0.12, 0.14) | -0.03 (-0.05, -0.01) | -0.02 (-0.08, 0.04) |
| Sao Tome and Principe | 1.74 (1.46, 2.08) | 3.48 (2.91, 4.10) | 99.51 | 1559.95 (1326.22, 1823.56) | 1563.78 (1325.27, 1811.27) | 1567.08 (1326.48, 1822.14) | 1554.12 (1314.11, 1808.16) | -0.01 (-0.02, 0.00) | 0.02 (-0.01, 0.05) | 0.02 (0.00, 0.04) | -0.09 (-0.10, -0.07) |
| Saudi Arabia | 123.58 (102.29, 149.86) | 347.36 (289.37, 419.33) | 181.09 | 757.18 (637.38, 901.65) | 762.53 (636.38, 907.22) | 764.77 (644.86, 907.86) | 758.57 (638.92, 901.19) | 0.01 (-0.01, 0.03) | 0.08 (0.07, 0.08) | 0.03 (-0.01, 0.07) | -0.09 (-0.13, -0.05) |
| Senegal | 98.58 (81.66, 117.61) | 217.22 (181.12, 257.14) | 120.35 | 1403.45 (1179.60, 1648.14) | 1395.93 (1186.14, 1635.41) | 1406.97 (1188.61, 1649.31) | 1398.30 (1183.00, 1634.31) | 0.01 (-0.01, 0.03) | -0.06 (-0.08, -0.04) | 0.08 (-0.01, 0.18) | -0.08 (-0.14, -0.03) |
| Serbia | 50.14 (42.30, 59.62) | 43.69 (37.01, 51.86) | -12.86 | 520.12 (435.65, 620.31) | 519.79 (437.02, 622.08) | 520.01 (435.02, 616.55) | 518.13 (434.58, 616.00) | -0.01 (-0.02, 0.00) | 0.01 (-0.01, 0.03) | 0.00 (0.00, 0.01) | -0.06 (-0.10, -0.02) |
| Seychelles | 0.89 (0.73, 1.07) | 1.20 (1.01, 1.41) | 35.01 | 1141.63 (953.94, 1347.19) | 1137.84 (956.36, 1331.47) | 1131.19 (948.59, 1326.12) | 1132.31 (953.66, 1327.60) | -0.05 (-0.06, -0.04) | -0.03 (-0.06, -0.01) | -0.06 (-0.08, -0.04) | -0.02 (-0.06, 0.02) |
| Sierra Leone | 55.80 (46.93, 66.10) | 141.29 (118.64, 167.63) | 153.22 | 1584.09 (1346.80, 1850.94) | 1605.30 (1357.27, 1869.13) | 1598.28 (1352.74, 1871.52) | 1581.35 (1342.86, 1843.32) | -0.04 (-0.06, -0.02) | 0.13 (0.05, 0.20) | -0.03 (-0.06, 0.01) | -0.11 (-0.12, -0.11) |
| Singapore | 27.44 (22.81, 32.96) | 45.00 (37.71, 53.94) | 63.96 | 728.56 (614.33, 868.58) | 723.74 (607.99, 852.63) | 718.62 (604.23, 853.23) | 731.53 (614.39, 860.44) | -0.01 (-0.03, 0.02) | -0.11 (-0.17, -0.04) | -0.11 (-0.19, -0.03) | 0.18 (0.11, 0.25) |
| Slovakia | 28.27 (23.79, 33.80) | 28.73 (24.08, 34.57) | 1.66 | 520.32 (434.67, 620.14) | 519.70 (438.67, 622.28) | 520.31 (435.93, 615.49) | 517.48 (432.41, 617.53) | -0.02 (-0.02, -0.01) | -0.02 (-0.03, 0.00) | 0.01 (0.00, 0.03) | -0.07 (-0.08, -0.06) |
| Slovenia | 10.88 (9.13, 12.99) | 10.02 (8.43, 11.98) | -7.90 | 519.46 (434.00, 618.82) | 518.77 (436.21, 620.81) | 518.38 (434.57, 613.33) | 515.56 (430.43, 613.20) | -0.02 (-0.03, -0.02) | -0.02 (-0.03, 0.00) | -0.01 (-0.02, 0.01) | -0.07 (-0.09, -0.05) |
| Solomon Islands | 4.33 (3.58, 5.22) | 9.02 (7.53, 10.66) | 108.02 | 1336.26 (1120.18, 1581.55) | 1343.19 (1126.59, 1572.45) | 1347.77 (1134.09, 1587.99) | 1340.52 (1129.33, 1573.65) | 0.01 (0.00, 0.03) | 0.05 (0.04, 0.06) | 0.04 (0.03, 0.04) | -0.12 (-0.18, -0.05) |
| Somalia | 133.73 (113.26, 160.41) | 416.27 (348.92, 494.25) | 211.28 | 1964.14 (1670.36, 2286.82) | 1988.81 (1698.23, 2332.98) | 1979.83 (1690.58, 2315.31) | 1972.26 (1683.60, 2295.01) | 0.00 (-0.02, 0.01) | 0.11 (0.06, 0.15) | -0.05 (-0.07, -0.03) | -0.03 (-0.06, 0.00) |
| South Africa | 877.46 (751.28, 1011.69) | 1400.02 (1214.63, 1601.38) | 59.55 | 2138.35 (1868.68, 2435.16) | 2232.24 (1954.70, 2530.29) | 2340.59 (2071.41, 2627.41) | 2230.74 (1945.42, 2548.26) | 0.34 (0.21, 0.46) | 0.52 (-0.05, 1.10) | 0.49 (0.37, 0.62) | -0.58 (-0.85, -0.32) |
| South Sudan | 122.82 (102.77, 146.10) | 185.28 (156.88, 219.27) | 50.86 | 1963.73 (1671.74, 2289.68) | 1999.80 (1699.64, 2314.84) | 1987.43 (1690.15, 2324.34) | 1984.30 (1696.49, 2294.57) | 0.01 (-0.01, 0.02) | 0.17 (0.12, 0.22) | -0.06 (-0.09, -0.04) | 0.02 (-0.02, 0.06) |
| Spain | 232.58 (197.73, 269.07) | 259.80 (219.79, 307.61) | 11.70 | 583.41 (494.36, 677.61) | 611.50 (525.70, 705.10) | 621.77 (523.76, 741.43) | 619.91 (521.12, 736.27) | 0.21 (0.13, 0.28) | 0.50 (0.47, 0.52) | 0.16 (-0.36, 0.69) | -0.03 (-0.08, 0.03) |
| Sri Lanka | 214.62 (179.80, 254.89) | 248.72 (211.38, 290.70) | 15.89 | 1141.77 (963.32, 1345.62) | 1140.76 (959.52, 1343.84) | 1146.17 (959.28, 1343.64) | 1146.72 (965.43, 1344.68) | 0.01 (0.00, 0.02) | -0.01 (-0.04, 0.01) | 0.05 (0.05, 0.05) | -0.03 (-0.08, 0.02) |
| Sudan | 150.71 (125.16, 182.78) | 339.59 (282.95, 410.90) | 125.33 | 803.89 (675.22, 960.16) | 801.99 (677.26, 954.08) | 804.48 (675.07, 952.72) | 801.83 (673.63, 949.24) | -0.01 (-0.02, 0.00) | -0.03 (-0.04, -0.01) | 0.03 (0.01, 0.05) | -0.06 (-0.09, -0.03) |
| Suriname | 6.65 (5.61, 7.81) | 9.38 (8.05, 10.88) | 41.09 | 1610.12 (1375.53, 1882.93) | 1617.69 (1372.84, 1886.03) | 1633.64 (1381.69, 1908.70) | 1630.33 (1391.41, 1900.64) | 0.05 (0.04, 0.06) | 0.05 (0.03, 0.07) | 0.10 (0.10, 0.11) | -0.02 (-0.10, 0.06) |
| Sweden | 66.47 (56.03, 78.47) | 74.58 (63.18, 88.99) | 12.19 | 827.09 (692.30, 982.26) | 817.55 (690.10, 964.14) | 771.42 (645.41, 917.94) | 808.06 (678.91, 965.30) | -0.19 (-0.31, -0.06) | -0.11 (-0.25, 0.04) | -0.57 (-0.93, -0.20) | 0.54 (0.28, 0.81) |
| Switzerland | 59.21 (55.01, 63.06) | 60.66 (51.40, 72.36) | 2.44 | 818.85 (763.93, 873.45) | 759.22 (647.95, 890.00) | 756.12 (633.62, 904.71) | 746.24 (624.54, 882.83) | -0.34 (-0.42, -0.27) | -0.79 (-1.10, -0.48) | -0.04 (-0.05, -0.03) | -0.13 (-0.16, -0.09) |
| Syrian Arab Republic | 77.28 (63.87, 93.06) | 103.89 (87.18, 123.49) | 34.43 | 667.70 (560.85, 803.14) | 666.99 (558.30, 791.55) | 674.83 (566.20, 804.30) | 693.67 (582.87, 824.04) | 0.13 (0.11, 0.16) | -0.02 (-0.07, 0.03) | 0.11 (0.04, 0.17) | 0.34 (0.25, 0.43) |
| Taiwan (Province of China) | 173.85 (144.42, 206.40) | 182.79 (153.35, 217.24) | 5.15 | 757.81 (639.35, 897.83) | 760.08 (634.57, 901.27) | 760.78 (635.98, 902.22) | 756.82 (630.65, 895.87) | 0.00 (-0.01, 0.01) | 0.03 (0.02, 0.04) | 0.01 (0.01, 0.01) | -0.07 (-0.09, -0.06) |
| Tajikistan | 33.23 (27.43, 39.88) | 68.92 (57.26, 83.17) | 107.38 | 688.14 (573.74, 811.02) | 691.49 (579.58, 821.24) | 690.53 (579.84, 824.10) | 687.27 (578.30, 825.37) | -0.01 (-0.02, 0.00) | 0.05 (0.04, 0.07) | -0.01 (-0.06, 0.04) | -0.07 (-0.09, -0.05) |
| Thailand | 860.77 (787.03, 941.77) | 830.69 (713.07, 972.75) | -3.49 | 1303.81 (1195.24, 1415.90) | 1398.42 (1265.94, 1532.52) | 1215.73 (1028.38, 1427.85) | 1219.93 (1030.95, 1445.55) | -0.64 (-0.80, -0.48) | 0.56 (0.02, 1.11) | -1.46 (-1.95, -0.96) | 0.01 (-0.03, 0.05) |
| Timor-Leste | 9.99 (8.29, 11.94) | 16.99 (14.09, 20.41) | 70.09 | 1249.79 (1053.28, 1472.73) | 1308.35 (1125.93, 1515.57) | 1252.66 (1052.66, 1473.34) | 1253.23 (1052.62, 1480.60) | -0.03 (-0.12, 0.05) | 0.48 (0.29, 0.66) | -0.45 (-0.63, -0.27) | -0.02 (-0.06, 0.02) |
| Togo | 55.76 (46.78, 66.19) | 130.50 (110.00, 154.05) | 134.05 | 1582.90 (1338.97, 1839.60) | 1599.21 (1361.62, 1853.38) | 1607.60 (1366.83, 1870.83) | 1582.23 (1346.64, 1838.09) | 0.02 (0.00, 0.04) | 0.10 (0.09, 0.10) | 0.06 (0.05, 0.06) | -0.17 (-0.20, -0.14) |
| Tokelau | 0.02 (0.02, 0.02) | 0.02 (0.01, 0.02) | -11.24 | 1335.68 (1120.82, 1572.32) | 1341.42 (1126.71, 1570.61) | 1348.66 (1139.66, 1584.55) | 1340.00 (1124.48, 1578.04) | 0.02 (0.00, 0.03) | 0.04 (0.03, 0.05) | 0.06 (0.04, 0.08) | -0.13 (-0.21, -0.05) |
| Tonga | 1.26 (1.05, 1.50) | 1.34 (1.13, 1.60) | 6.70 | 1335.40 (1121.10, 1570.71) | 1340.67 (1124.31, 1572.06) | 1345.13 (1130.40, 1579.44) | 1337.50 (1118.51, 1581.76) | 0.01 (0.00, 0.03) | 0.04 (0.03, 0.05) | 0.03 (0.03, 0.04) | -0.13 (-0.22, -0.03) |
| Trinidad and Tobago | 20.91 (17.59, 24.52) | 22.24 (19.01, 26.00) | 6.37 | 1623.89 (1386.09, 1886.69) | 1625.51 (1380.10, 1886.32) | 1631.03 (1381.84, 1903.60) | 1623.15 (1371.52, 1894.76) | 0.00 (-0.02, 0.01) | 0.01 (0.00, 0.03) | 0.04 (0.03, 0.04) | -0.05 (-0.13, 0.03) |
| Tunisia | 67.73 (56.41, 81.03) | 96.13 (80.89, 114.60) | 41.94 | 798.96 (670.35, 945.46) | 797.61 (666.79, 949.87) | 802.41 (676.18, 954.86) | 800.20 (671.00, 954.74) | 0.01 (0.00, 0.01) | -0.02 (-0.04, -0.01) | 0.06 (0.05, 0.07) | -0.04 (-0.06, -0.01) |
| Turkey | 423.64 (352.83, 508.62) | 614.72 (518.11, 736.43) | 45.10 | 693.11 (580.20, 821.47) | 606.76 (533.92, 686.31) | 652.21 (576.85, 738.62) | 679.45 (571.58, 810.89) | -0.04 (-0.22, 0.14) | -1.36 (-2.00, -0.72) | 0.75 (0.39, 1.11) | 0.51 (0.33, 0.69) |
| Turkmenistan | 24.35 (20.00, 29.47) | 36.63 (30.65, 43.65) | 50.42 | 690.09 (579.41, 820.55) | 689.44 (578.26, 822.52) | 684.87 (575.11, 817.88) | 681.85 (574.49, 810.11) | -0.06 (-0.06, -0.05) | -0.01 (-0.02, 0.00) | -0.07 (-0.12, -0.02) | -0.06 (-0.09, -0.04) |
| Tuvalu | 0.12 (0.10, 0.15) | 0.17 (0.14, 0.20) | 34.60 | 1335.54 (1119.71, 1577.29) | 1340.44 (1125.38, 1569.49) | 1348.66 (1138.36, 1588.73) | 1341.43 (1125.99, 1581.09) | 0.03 (0.01, 0.04) | 0.04 (0.02, 0.05) | 0.06 (0.05, 0.07) | -0.12 (-0.20, -0.04) |
| Uganda | 567.79 (529.12, 603.55) | 1004.18 (848.41, 1178.64) | 76.86 | 3036.67 (2828.25, 3247.69) | 2754.98 (2574.06, 2954.00) | 2538.48 (2306.45, 2765.31) | 2250.65 (1940.23, 2585.59) | -1.39 (-1.58, -1.20) | -0.99 (-1.48, -0.49) | -0.76 (-0.84, -0.67) | -1.13 (-2.84, 0.62) |
| Ukraine | 482.19 (406.67, 569.39) | 392.24 (329.58, 465.57) | -18.65 | 932.71 (782.36, 1098.07) | 933.90 (785.55, 1100.24) | 933.31 (785.50, 1101.43) | 933.83 (781.13, 1103.14) | 0.00 (-0.01, 0.00) | 0.02 (0.00, 0.04) | -0.01 (-0.02, 0.01) | -0.01 (-0.05, 0.03) |
| United Arab Emirates | 16.03 (13.03, 19.61) | 84.26 (67.99, 106.44) | 425.67 | 720.31 (604.39, 854.35) | 711.46 (592.60, 849.69) | 687.51 (579.84, 816.41) | 725.74 (606.07, 866.69) | -0.07 (-0.13, 0.00) | -0.13 (-0.13, -0.12) | -0.44 (-0.66, -0.21) | 0.60 (0.58, 0.62) |
| United Kingdom | 347.69 (294.33, 409.33) | 390.65 (330.91, 462.33) | 12.36 | 601.43 (505.64, 708.16) | 604.98 (511.18, 715.97) | 608.29 (511.21, 719.14) | 609.41 (514.42, 718.17) | 0.04 (0.03, 0.06) | 0.06 (0.04, 0.08) | 0.05 (-0.02, 0.12) | 0.01 (-0.02, 0.04) |
| United Republic of Tanzania | 528.40 (442.04, 632.89) | 1111.65 (933.37, 1317.04) | 110.38 | 1997.57 (1698.97, 2335.08) | 1891.20 (1692.22, 2104.70) | 1930.60 (1645.58, 2261.56) | 1891.99 (1601.91, 2206.51) | 0.24 (0.08, 0.39) | -0.30 (-1.49, 0.91) | 0.20 (-0.04, 0.43) | -0.21 (-0.28, -0.14) |
| United States of America | 3415.99 (2875.29, 4026.80) | 3392.11 (2897.48, 3962.93) | -0.70 | 1286.25 (1087.05, 1513.46) | 1249.97 (1062.54, 1466.03) | 1138.77 (960.86, 1342.58) | 1118.46 (941.32, 1316.91) | -0.60 (-0.71, -0.49) | -0.29 (-0.37, -0.22) | -0.92 (-1.51, -0.33) | -0.16 (-0.25, -0.06) |
| United States Virgin Islands | 1.75 (1.50, 2.02) | 1.39 (1.20, 1.61) | -20.23 | 1643.39 (1396.63, 1913.30) | 1651.44 (1402.74, 1915.04) | 1664.94 (1412.66, 1941.95) | 1636.53 (1394.21, 1909.32) | 0.01 (-0.02, 0.03) | 0.06 (0.04, 0.07) | 0.09 (0.08, 0.10) | -0.17 (-0.25, -0.10) |
| Uruguay | 36.59 (31.16, 42.38) | 39.68 (33.76, 46.24) | 8.42 | 1213.05 (1032.54, 1407.53) | 1210.48 (1026.43, 1420.74) | 1216.55 (1028.23, 1423.03) | 1218.59 (1032.26, 1432.19) | 0.02 (0.01, 0.03) | -0.03 (-0.05, -0.01) | 0.05 (0.01, 0.09) | 0.02 (0.00, 0.04) |
| Uzbekistan | 135.43 (110.96, 162.68) | 253.29 (210.73, 304.14) | 87.02 | 688.05 (574.80, 818.97) | 691.27 (581.59, 823.33) | 690.98 (578.53, 821.30) | 690.02 (580.47, 826.61) | 0.00 (0.00, 0.01) | 0.05 (0.04, 0.06) | -0.01 (-0.04, 0.03) | -0.03 (-0.06, -0.01) |
| Vanuatu | 1.82 (1.51, 2.16) | 3.75 (3.10, 4.44) | 105.55 | 1236.32 (1037.49, 1458.80) | 1252.23 (1047.79, 1462.65) | 1251.35 (1053.34, 1470.19) | 1237.77 (1033.96, 1452.88) | 0.01 (-0.02, 0.04) | 0.13 (0.12, 0.15) | -0.01 (-0.05, 0.03) | -0.20 (-0.32, -0.07) |
| Venezuela (Bolivarian Republic of) | 336.37 (281.98, 393.23) | 471.53 (406.80, 547.18) | 40.18 | 1656.10 (1407.78, 1906.85) | 1659.92 (1410.21, 1932.23) | 1653.39 (1409.31, 1921.89) | 1663.88 (1426.04, 1936.35) | 0.01 (0.00, 0.02) | 0.03 (0.00, 0.06) | -0.04 (-0.05, -0.03) | 0.10 (0.03, 0.17) |
| Viet Nam | 770.20 (650.19, 909.14) | 1168.95 (981.81, 1389.22) | 51.77 | 1099.41 (939.64, 1272.38) | 1091.71 (934.36, 1265.61) | 1104.60 (930.51, 1299.61) | 1105.02 (924.63, 1309.18) | 0.04 (0.02, 0.05) | -0.07 (-0.07, -0.07) | 0.13 (0.09, 0.17) | -0.02 (-0.06, 0.01) |
| Yemen | 91.31 (75.75, 111.12) | 253.08 (209.45, 306.82) | 177.18 | 798.98 (671.17, 954.85) | 793.99 (665.17, 942.18) | 797.97 (672.56, 950.13) | 797.06 (668.33, 951.43) | -0.01 (-0.01, 0.00) | -0.07 (-0.08, -0.06) | 0.05 (0.04, 0.07) | -0.02 (-0.05, 0.02) |
| Zambia | 189.61 (159.06, 224.65) | 469.99 (399.46, 548.05) | 147.87 | 2257.14 (1937.58, 2597.21) | 2684.44 (2505.63, 2855.73) | 2608.36 (2364.18, 2848.89) | 2295.54 (1980.73, 2634.72) | -0.19 (-0.45, 0.08) | 1.64 (1.34, 1.94) | -0.32 (-0.47, -0.17) | -1.48 (-2.03, -0.92) |
| Zimbabwe | 235.81 (199.38, 277.85) | 387.63 (333.36, 449.41) | 64.38 | 2262.90 (1951.22, 2590.32) | 2516.52 (2241.11, 2819.62) | 2536.46 (2294.30, 2783.99) | 2388.79 (2071.34, 2753.15) | -0.02 (-0.14, 0.10) | 0.97 (0.54, 1.39) | 0.09 (-0.22, 0.40) | -0.73 (-1.02, -0.44) |

Note: EAPC, estimated annual percentage change; CIs, confidence intervals; UIs, uncertainty intervals

**Table S4 The age-standardized incidence rates (ASR, per 100 000 population) of sexually transmitted infections, and their temporal trends from 1990 to 2019.**

| Characteristics | Age-standardized incidence rate (ASR, per 100 000) | |  |  |  |
| --- | --- | --- | --- | --- | --- |
|  | 1990 (95% UI) | 2000 (95% UI) | 2019 (95% UI) | EAPC (95% CI) 1990-2019 | EAPC (95% CI) 1990-2000 |
| **Syphilis** |  |  |  |  |  |
| Global | 160.03 (120.66, 208.10) | 166.27 (125.58, 213.78) | 178.48 (134.94, 232.34) | 0.16 (0.06, 0.26) | 0.41 (0.38, 0.44) |
| Sex |  |  |  |  |  |
| Female | 119.95 (90.17, 156.34) | 123.28 (93.98, 157.74) | 124.98 (96.68, 160.25) | -0.30 (-0.50, -0.10) | 0.32 (0.27, 0.36) |
| Male | 199.14 (149.81, 260.75) | 208.66 (157.31, 272.68) | 231.31 (171.88, 305.30) | 0.43 (0.38, 0.47) | 0.48 (0.45, 0.51) |
| SDI region |  |  |  |  |  |
| Low | 467.40 (356.95, 593.73) | 408.24 (317.03, 512.29) | 372.53 (287.40, 475.57) | -0.67 (-0.75, -0.58) | -0.81 (-1.36, -0.26) |
| Low-middle | 224.35 (166.26, 294.69) | 228.72 (171.63, 295.61) | 214.66 (160.77, 280.80) | -0.33 (-0.46, -0.20) | 0.34 (0.12, 0.56) |
| Middle | 136.26 (102.10, 179.16) | 124.45 (93.46, 162.90) | 130.13 (96.65, 171.91) | -0.14 (-0.28, 0.00) | -0.51 (-0.96, -0.06) |
| High-middle | 82.22 (61.68, 108.01) | 85.37 (64.10, 112.78) | 90.96 (68.81, 118.20) | 0.09 (-0.01, 0.20) | 0.41 (0.38, 0.44) |
| High | 71.52 (54.07, 94.72) | 72.67 (54.73, 97.16) | 74.05 (55.61, 98.92) | 0.16 (0.10, 0.22) | 0.16 (0.00, 0.32) |
| GBD region |  |  |  |  |  |
| Andean Latin America | 221.73 (167.05, 293.67) | 231.87 (181.22, 298.52) | 207.84 (158.51, 270.68) | -0.45 (-0.55, -0.35) | 0.39 (0.17, 0.61) |
| Australasia | 65.58 (48.71, 87.54) | 65.15 (48.70, 86.86) | 64.07 (47.75, 85.84) | -0.12 (-0.13, -0.10) | -0.07 (-0.10, -0.04) |
| Caribbean | 165.87 (125.65, 216.09) | 175.76 (134.33, 227.29) | 191.99 (156.21, 235.61) | 0.49 (0.43, 0.55) | 0.57 (0.47, 0.67) |
| Central Asia | 54.85 (41.88, 71.72) | 55.81 (42.53, 73.31) | 52.10 (40.26, 67.34) | -0.36 (-0.42, -0.30) | 0.14 (-0.03, 0.31) |
| Central Europe | 43.38 (32.95, 56.96) | 42.88 (32.62, 56.88) | 43.28 (32.96, 56.54) | -0.02 (-0.05, 0.01) | -0.12 (-0.15, -0.09) |
| Central Latin America | 118.92 (89.05, 156.63) | 116.20 (87.60, 152.30) | 111.77 (86.08, 145.02) | -0.27 (-0.33, -0.21) | -0.23 (-0.25, -0.22) |
| Central Sub-Saharan Africa | 1153.28 (880.53, 1482.29) | 1159.65 (878.24, 1470.11) | 1048.40 (803.52, 1344.82) | -0.50 (-0.59, -0.40) | -0.01 (-0.33, 0.32) |
| East Asia | 89.28 (66.65, 118.27) | 87.51 (65.45, 116.19) | 93.43 (69.27, 123.92) | -0.08 (-0.22, 0.05) | -0.20 (-0.23, -0.16) |
| Eastern Europe | 53.10 (40.90, 68.98) | 53.09 (40.96, 68.94) | 47.97 (37.05, 62.56) | -0.56 (-0.65, -0.47) | -0.02 (-0.08, 0.05) |
| Eastern Sub-Saharan Africa | 669.08 (522.83, 834.07) | 581.30 (465.28, 718.82) | 492.63 (386.33, 619.27) | -1.42 (-1.62, -1.22) | -1.39 (-1.63, -1.14) |
| High-income Asia Pacific | 78.31 (59.18, 103.74) | 75.79 (57.24, 101.33) | 81.36 (60.92, 109.14) | 0.28 (0.22, 0.34) | -0.26 (-0.53, 0.01) |
| High-income North America | 68.27 (51.74, 89.69) | 73.46 (55.70, 97.50) | 71.76 (54.06, 95.06) | 0.18 (-0.04, 0.40) | 0.71 (0.38, 1.03) |
| North Africa and Middle East | 79.67 (59.21, 106.06) | 81.84 (61.87, 106.20) | 84.20 (62.65, 111.70) | 0.15 (0.06, 0.24) | 0.17 (-0.23, 0.56) |
| Oceania | 430.43 (320.80, 560.81) | 483.40 (365.20, 613.56) | 415.80 (301.62, 551.31) | -0.56 (-0.74, -0.37) | 1.05 (0.63, 1.47) |
| South Asia | 219.04 (161.13, 289.42) | 228.99 (168.89, 302.01) | 190.99 (140.95, 254.13) | -0.71 (-0.96, -0.46) | 0.54 (0.11, 0.98) |
| Southeast Asia | 101.17 (74.45, 134.79) | 103.49 (77.69, 136.06) | 99.61 (73.45, 131.74) | -0.14 (-0.19, -0.09) | 0.19 (0.03, 0.35) |
| Southern Latin America | 117.88 (88.25, 155.84) | 116.11 (90.76, 146.47) | 131.89 (106.80, 163.69) | 0.26 (0.07, 0.45) | -0.22 (-0.71, 0.27) |
| Southern Sub-Saharan Africa | 916.80 (697.82, 1174.15) | 628.79 (491.31, 792.21) | 665.35 (496.08, 872.21) | -1.19 (-1.71, -0.67) | -3.74 (-4.01, -3.46) |
| Tropical Latin America | 119.04 (88.38, 158.58) | 123.86 (91.26, 166.52) | 139.68 (109.18, 174.26) | -0.66 (-1.53, 0.21) | 0.36 (-0.14, 0.87) |
| Western Europe | 68.34 (51.17, 90.38) | 67.68 (50.38, 90.60) | 67.63 (50.12, 90.19) | -0.03 (-0.04, -0.02) | -0.10 (-0.11, -0.09) |
| Western Sub-Saharan Africa | 461.93 (345.94, 598.09) | 440.34 (336.85, 558.49) | 427.97 (318.21, 556.32) | -0.26 (-0.31, -0.20) | -0.49 (-0.55, -0.43) |
| **Chlamydial infection** | |  |  |  |  |
| Global | 2867.67 (2150.60, 3741.43) | 3083.25 (2319.33, 4006.82) | 2883.87 (2161.21, 3762.80) | -0.21 (-0.36, -0.06) | 0.77 (0.52, 1.03) |
| Sex |  |  |  |  |  |
| Female | 2579.57 (1964.61, 3360.43) | 2779.49 (2122.28, 3604.37) | 2677.33 (2027.51, 3505.38) | -0.10 (-0.25, 0.05) | 0.79 (0.50, 1.09) |
| Male | 3147.30 (2334.47, 4129.53) | 3381.46 (2515.44, 4448.60) | 3088.09 (2286.90, 4039.30) | -0.29 (-0.45, -0.14) | 0.76 (0.54, 0.99) |
| SDI region |  |  |  |  |  |
| Low | 2501.78 (1887.37, 3273.67) | 2308.12 (1749.61, 3006.30) | 2359.48 (1770.16, 3098.25) | -0.04 (-0.12, 0.04) | -0.49 (-0.79, -0.19) |
| Low-middle | 2613.36 (1957.08, 3396.20) | 2624.46 (1979.48, 3412.47) | 2524.34 (1894.78, 3281.85) | -0.26 (-0.33, -0.19) | 0.12 (0.04, 0.21) |
| Middle | 3795.24 (2844.39, 4961.53) | 3770.57 (2837.26, 4896.10) | 3477.61 (2601.94, 4553.28) | -0.28 (-0.45, -0.11) | 0.42 (-0.30, 1.14) |
| High-middle | 3252.41 (2425.07, 4244.58) | 3540.95 (2659.46, 4617.09) | 3262.50 (2430.97, 4273.94) | -0.36 (-0.59, -0.14) | 0.92 (0.50, 1.34) |
| High | 1066.96 (809.15, 1396.85) | 1183.75 (894.38, 1549.34) | 1241.28 (936.52, 1617.46) | 0.37 (0.29, 0.46) | 1.08 (0.94, 1.21) |
| GBD region |  |  |  |  |  |
| Andean Latin America | 2554.10 (1884.77, 3342.26) | 2601.00 (2007.77, 3307.74) | 2520.26 (1870.63, 3304.31) | 0.14 (0.09, 0.19) | 0.19 (0.09, 0.29) |
| Australasia | 1068.87 (804.24, 1386.40) | 977.68 (753.29, 1252.00) | 1051.10 (784.76, 1364.78) | -0.09 (-0.24, 0.07) | -0.90 (-1.27, -0.52) |
| Caribbean | 4394.72 (3283.30, 5756.82) | 4379.71 (3271.68, 5704.71) | 4340.05 (3228.30, 5660.33) | -0.04 (-0.04, -0.04) | -0.04 (-0.05, -0.02) |
| Central Asia | 5277.11 (3947.90, 6912.30) | 5331.46 (3994.38, 6919.09) | 5271.28 (3946.14, 6857.60) | -0.03 (-0.05, -0.02) | 0.10 (0.10, 0.11) |
| Central Europe | 3343.90 (2499.19, 4405.11) | 3324.84 (2502.79, 4375.42) | 3308.59 (2464.68, 4333.19) | -0.04 (-0.05, -0.04) | -0.06 (-0.08, -0.04) |
| Central Latin America | 3868.04 (2927.51, 5035.54) | 3855.76 (2909.64, 5035.82) | 3850.00 (2903.85, 5041.57) | 0.19 (0.12, 0.26) | -0.03 (-0.05, -0.02) |
| Central Sub-Saharan Africa | 2134.79 (1580.51, 2827.54) | 2127.03 (1594.66, 2789.84) | 2111.27 (1568.66, 2799.00) | -0.02 (-0.04, 0.00) | -0.06 (-0.17, 0.05) |
| East Asia | 4049.64 (3024.98, 5270.00) | 4718.45 (3538.74, 6159.79) | 4128.96 (3082.92, 5375.67) | -0.63 (-1.05, -0.22) | 1.64 (0.89, 2.40) |
| Eastern Europe | 3644.40 (2722.06, 4737.11) | 3653.06 (2719.44, 4788.43) | 3625.05 (2701.70, 4753.04) | -0.01 (-0.02, 0.00) | 0.04 (-0.02, 0.09) |
| Eastern Sub-Saharan Africa | 3328.85 (2535.14, 4331.98) | 3262.62 (2483.19, 4208.26) | 3223.05 (2422.13, 4236.64) | -0.14 (-0.18, -0.10) | -0.24 (-0.47, 0.00) |
| High-income Asia Pacific | 1005.98 (753.84, 1306.50) | 1030.44 (773.81, 1346.38) | 1025.36 (767.38, 1355.75) | 0.09 (0.05, 0.13) | 0.24 (0.13, 0.35) |
| High-income North America | 695.29 (521.41, 915.65) | 867.76 (657.02, 1140.15) | 740.09 (554.75, 968.78) | -0.64 (-0.99, -0.30) | 2.24 (1.92, 2.55) |
| North Africa and Middle East | 3595.96 (2747.60, 4638.74) | 3495.74 (2682.90, 4517.80) | 3264.95 (2470.04, 4246.76) | -0.23 (-0.27, -0.19) | -0.26 (-0.45, -0.07) |
| Oceania | 4212.36 (3273.43, 5296.76) | 3831.27 (2924.20, 4862.43) | 3701.31 (2826.22, 4759.19) | -0.52 (-0.59, -0.44) | -0.91 (-1.11, -0.70) |
| South Asia | 1731.73 (1290.19, 2262.77) | 1612.88 (1202.99, 2108.48) | 1711.93 (1270.93, 2239.34) | -0.22 (-0.37, -0.07) | -0.71 (-0.98, -0.44) |
| Southeast Asia | 4330.29 (3251.49, 5622.52) | 4251.31 (3200.02, 5557.87) | 4252.09 (3180.39, 5558.88) | 0.00 (-0.02, 0.03) | -0.15 (-0.27, -0.03) |
| Southern Latin America | 885.29 (671.59, 1144.00) | 886.82 (672.61, 1145.16) | 908.41 (686.00, 1182.24) | 0.10 (0.08, 0.13) | 0.02 (0.02, 0.02) |
| Southern Sub-Saharan Africa | 5439.95 (4127.48, 7073.07) | 5890.00 (4593.74, 7540.13) | 5324.43 (4039.48, 6940.61) | 0.09 (-0.07, 0.26) | 0.67 (0.32, 1.03) |
| Tropical Latin America | 4082.07 (3067.76, 5372.83) | 4311.13 (3250.51, 5635.92) | 4102.22 (3075.72, 5393.45) | -0.17 (-0.30, -0.03) | 0.58 (0.24, 0.92) |
| Western Europe | 435.95 (331.57, 568.59) | 434.50 (335.70, 560.53) | 429.93 (327.62, 559.92) | -0.04 (-0.09, 0.01) | -0.02 (-0.09, 0.04) |
| Western Sub-Saharan Africa | 2291.53 (1725.68, 3009.75) | 2307.63 (1760.38, 2997.46) | 2316.40 (1741.46, 3047.29) | 0.41 (0.28, 0.54) | 0.06 (-0.01, 0.12) |
| **Gonococcal infection** | |  |  |  |  |
| Global | 1178.58 (912.29, 1536.00) | 1171.43 (910.47, 1519.18) | 1124.39 (872.97, 1441.08) | -0.14 (-0.19, -0.08) | -0.08 (-0.17, 0.00) |
| Sex |  |  |  |  |  |
| Female | 843.96 (641.86, 1118.68) | 828.37 (643.70, 1076.90) | 746.62 (573.55, 970.89) | -0.52 (-0.58, -0.47) | -0.21 (-0.23, -0.18) |
| Male | 1505.50 (1165.86, 1957.26) | 1508.73 (1172.48, 1962.99) | 1494.21 (1154.10, 1922.58) | 0.07 (0.00, 0.14) | 0.00 (-0.12, 0.11) |
| SDI region |  |  |  |  |  |
| Low | 1262.07 (994.05, 1617.96) | 1166.82 (935.68, 1459.39) | 1121.20 (876.87, 1461.59) | -0.11 (-0.19, -0.04) | -0.39 (-0.80, 0.03) |
| Low-middle | 1238.37 (955.62, 1614.98) | 1191.93 (924.61, 1539.36) | 1141.32 (883.23, 1474.10) | -0.08 (-0.20, 0.03) | -0.35 (-0.43, -0.27) |
| Middle | 1367.36 (1036.82, 1830.88) | 1274.29 (981.92, 1686.96) | 1225.96 (936.47, 1618.37) | -0.20 (-0.25, -0.14) | -0.38 (-0.70, -0.05) |
| High-middle | 1285.36 (993.00, 1683.57) | 1263.43 (961.25, 1672.92) | 1167.61 (896.78, 1523.30) | -0.39 (-0.44, -0.34) | -0.14 (-0.21, -0.06) |
| High | 462.86 (369.07, 587.06) | 460.13 (365.01, 577.98) | 458.63 (365.83, 581.52) | -0.13 (-0.17, -0.09) | -0.07 (-0.28, 0.14) |
| GBD region |  |  |  |  |  |
| Andean Latin America | 259.59 (184.84, 369.47) | 245.05 (179.18, 335.93) | 254.51 (180.90, 363.56) | -0.05 (-0.16, 0.07) | -0.61 (-0.94, -0.27) |
| Australasia | 322.63 (245.25, 424.84) | 315.24 (240.84, 418.93) | 306.23 (230.23, 408.85) | -0.20 (-0.22, -0.18) | -0.24 (-0.27, -0.21) |
| Caribbean | 1205.31 (832.24, 1791.07) | 1263.01 (869.10, 1904.58) | 1199.47 (829.79, 1791.33) | -0.18 (-0.27, -0.10) | 0.49 (0.37, 0.62) |
| Central Asia | 2472.40 (1688.06, 3659.72) | 2494.48 (1716.54, 3708.69) | 2269.24 (1542.52, 3364.37) | -0.44 (-0.50, -0.39) | 0.09 (0.03, 0.15) |
| Central Europe | 1912.48 (1442.88, 2618.76) | 1892.05 (1434.88, 2528.26) | 1816.54 (1375.73, 2454.40) | -0.24 (-0.26, -0.23) | -0.11 (-0.12, -0.09) |
| Central Latin America | 788.91 (615.39, 1011.34) | 791.13 (615.74, 1012.26) | 772.79 (604.45, 993.38) | -0.08 (-0.10, -0.06) | 0.04 (-0.03, 0.12) |
| Central Sub-Saharan Africa | 1238.79 (878.90, 1760.98) | 1240.68 (892.67, 1744.89) | 1187.74 (842.19, 1721.81) | -0.16 (-0.19, -0.13) | 0.00 (-0.12, 0.11) |
| East Asia | 1232.18 (870.03, 1743.36) | 1159.74 (822.14, 1624.76) | 1088.78 (765.14, 1508.29) | -0.54 (-0.59, -0.49) | -0.63 (-0.67, -0.59) |
| Eastern Europe | 2155.48 (1564.29, 3003.93) | 2186.94 (1573.90, 3078.48) | 2115.43 (1511.49, 2972.55) | -0.28 (-0.33, -0.23) | 0.11 (-0.06, 0.28) |
| Eastern Sub-Saharan Africa | 1434.45 (1116.98, 1888.56) | 1439.49 (1167.23, 1797.00) | 1363.62 (1054.26, 1804.90) | -0.16 (-0.21, -0.10) | 0.02 (-0.03, 0.08) |
| High-income Asia Pacific | 679.57 (539.62, 856.22) | 672.03 (533.51, 839.84) | 671.08 (530.73, 845.19) | -0.06 (-0.08, -0.03) | -0.13 (-0.28, 0.02) |
| High-income North America | 372.72 (277.25, 498.14) | 337.54 (252.09, 454.62) | 375.03 (277.70, 501.02) | -0.28 (-0.43, -0.14) | -1.02 (-1.46, -0.58) |
| North Africa and Middle East | 1302.37 (973.98, 1769.34) | 1225.85 (933.31, 1655.77) | 1164.03 (853.33, 1642.04) | -0.34 (-0.37, -0.31) | -0.56 (-0.66, -0.47) |
| Oceania | 1719.15 (1184.77, 2563.92) | 1750.69 (1202.14, 2571.57) | 1693.15 (1139.36, 2590.01) | 0.37 (0.26, 0.48) | 0.20 (0.13, 0.27) |
| South Asia | 1237.69 (885.46, 1730.36) | 1174.18 (856.28, 1634.05) | 1134.27 (814.83, 1598.03) | 0.05 (-0.14, 0.25) | -0.56 (-0.73, -0.39) |
| Southeast Asia | 1341.76 (1073.29, 1687.69) | 1375.22 (1096.70, 1731.81) | 1335.19 (1061.49, 1687.72) | -0.14 (-0.19, -0.09) | 0.18 (-0.12, 0.48) |
| Southern Latin America | 438.40 (315.10, 616.12) | 436.87 (314.28, 613.75) | 430.95 (309.16, 610.91) | -0.08 (-0.10, -0.07) | -0.05 (-0.10, 0.00) |
| Southern Sub-Saharan Africa | 4141.94 (3158.45, 5461.20) | 4515.26 (3453.11, 5939.45) | 3869.55 (2945.69, 5086.97) | -0.42 (-0.57, -0.27) | 0.70 (0.15, 1.26) |
| Tropical Latin America | 932.92 (651.29, 1331.07) | 1027.67 (707.44, 1473.02) | 912.93 (631.86, 1293.01) | -0.29 (-0.49, -0.09) | 1.04 (0.57, 1.51) |
| Western Europe | 136.33 (108.60, 172.43) | 134.69 (107.29, 171.12) | 130.45 (104.25, 165.78) | -0.29 (-0.33, -0.25) | -0.13 (-0.20, -0.06) |
| Western Sub-Saharan Africa | 1413.27 (1132.45, 1780.93) | 1394.78 (1130.39, 1732.43) | 1318.35 (1046.77, 1674.62) | -0.25 (-0.27, -0.22) | -0.13 (-0.14, -0.11) |
| **Trichomoniasis** |  |  |  |  |  |
| Global | 4157.14 (3061.97, 5439.34) | 4186.35 (3083.69, 5458.95) | 4327.29 (3176.53, 5645.76) | 0.06 (0.03, 0.09) | 0.06 (-0.03, 0.15) |
| Sex |  |  |  |  |  |
| Female | 3526.67 (2549.78, 4680.89) | 3526.77 (2562.30, 4650.06) | 3781.34 (2719.77, 5010.59) | 0.08 (0.00, 0.16) | -0.03 (-0.25, 0.19) |
| Male | 4783.30 (3546.74, 6265.41) | 4843.00 (3586.19, 6349.38) | 4879.67 (3610.00, 6366.99) | 0.06 (0.05, 0.07) | 0.13 (0.12, 0.13) |
| SDI region |  |  |  |  |  |
| Low | 6205.09 (4630.64, 8055.23) | 5540.55 (4147.65, 7180.19) | 5748.06 (4236.67, 7502.65) | -0.05 (-0.15, 0.05) | -0.66 (-1.11, -0.20) |
| Low-middle | 3943.19 (2908.65, 5161.43) | 3959.13 (2933.69, 5145.98) | 4051.78 (2978.09, 5277.78) | 0.04 (0.02, 0.07) | 0.10 (0.02, 0.18) |
| Middle | 4561.33 (3350.11, 5960.63) | 4194.99 (3072.36, 5464.94) | 4267.97 (3116.15, 5565.46) | -0.08 (-0.15, -0.01) | -0.39 (-0.84, 0.06) |
| High-middle | 3762.35 (2754.87, 4936.43) | 3758.28 (2749.51, 4933.69) | 3829.67 (2796.45, 4987.86) | -0.03 (-0.07, 0.02) | 0.00 (-0.09, 0.08) |
| High | 3395.55 (2497.77, 4457.18) | 3390.04 (2488.12, 4442.00) | 3481.39 (2552.86, 4554.16) | 0.07 (0.06, 0.09) | -0.03 (-0.09, 0.03) |
| GBD region |  |  |  |  |  |
| Andean Latin America | 4498.62 (3322.38, 5854.37) | 4409.82 (3281.53, 5701.06) | 4490.13 (3298.29, 5860.57) | -0.01 (-0.05, 0.02) | -0.20 (-0.22, -0.18) |
| Australasia | 2811.07 (2087.71, 3670.93) | 2810.75 (2081.79, 3694.06) | 2803.25 (2080.30, 3648.48) | -0.03 (-0.04, -0.01) | 0.00 (-0.01, 0.02) |
| Caribbean | 5065.27 (3751.82, 6583.66) | 5078.91 (3766.07, 6603.44) | 5228.27 (3855.21, 6815.48) | 0.08 (0.05, 0.11) | 0.02 (-0.03, 0.06) |
| Central Asia | 3890.46 (2890.29, 5068.60) | 3944.22 (2947.10, 5122.80) | 3954.09 (2912.62, 5161.83) | 0.01 (-0.02, 0.04) | 0.13 (0.11, 0.16) |
| Central Europe | 3379.12 (2494.82, 4423.50) | 3392.01 (2515.84, 4402.16) | 3463.90 (2553.06, 4533.86) | 0.06 (0.04, 0.08) | 0.04 (0.03, 0.05) |
| Central Latin America | 6280.82 (4635.10, 8164.21) | 6252.66 (4613.33, 8102.32) | 6519.08 (4766.17, 8493.79) | 0.10 (0.06, 0.14) | -0.07 (-0.18, 0.04) |
| Central Sub-Saharan Africa | 5510.93 (4103.42, 7183.52) | 5399.63 (4023.69, 7068.97) | 5500.77 (4075.69, 7157.30) | 0.00 (-0.04, 0.03) | -0.24 (-0.43, -0.06) |
| East Asia | 4475.04 (3264.13, 5874.81) | 4338.44 (3155.02, 5683.44) | 4403.83 (3195.76, 5762.80) | -0.17 (-0.25, -0.09) | -0.31 (-0.41, -0.22) |
| Eastern Europe | 3120.96 (2275.14, 4081.92) | 3119.98 (2253.81, 4096.27) | 3170.49 (2289.66, 4152.30) | 0.02 (-0.01, 0.05) | -0.01 (-0.03, 0.01) |
| Eastern Sub-Saharan Africa | 10141.35 (7559.88, 13080.32) | 10094.23 (7664.31, 12884.63) | 10014.29 (7336.36, 12908.25) | -0.12 (-0.16, -0.07) | -0.08 (-0.27, 0.11) |
| High-income Asia Pacific | 3219.60 (2363.31, 4214.67) | 3172.89 (2336.66, 4167.99) | 3247.76 (2375.14, 4239.47) | 0.01 (-0.02, 0.03) | -0.16 (-0.23, -0.09) |
| High-income North America | 4328.49 (3134.41, 5671.09) | 4235.13 (3067.24, 5535.79) | 4224.42 (3049.86, 5536.13) | -0.09 (-0.12, -0.07) | -0.23 (-0.33, -0.13) |
| North Africa and Middle East | 3757.47 (2797.83, 4844.18) | 3887.06 (2933.92, 4956.65) | 3633.77 (2699.73, 4675.87) | -0.17 (-0.23, -0.12) | 0.33 (0.30, 0.36) |
| Oceania | 6821.62 (5163.17, 8868.33) | 7012.73 (5245.51, 9124.38) | 7046.62 (5258.47, 9082.33) | 0.24 (0.17, 0.32) | 0.25 (0.16, 0.35) |
| South Asia | 3040.75 (2225.98, 3995.74) | 3050.51 (2233.86, 3995.18) | 2989.08 (2182.21, 3904.48) | -0.16 (-0.20, -0.13) | 0.04 (0.03, 0.05) |
| Southeast Asia | 4449.71 (3303.43, 5796.64) | 4424.34 (3261.56, 5793.71) | 4455.60 (3257.20, 5836.66) | -0.02 (-0.04, 0.00) | -0.06 (-0.09, -0.03) |
| Southern Latin America | 2970.93 (2193.77, 3869.12) | 2922.52 (2188.21, 3790.97) | 2963.95 (2200.04, 3863.44) | 0.00 (-0.02, 0.02) | -0.17 (-0.21, -0.13) |
| Southern Sub-Saharan Africa | 8424.86 (6211.63, 10932.88) | 7869.53 (5829.00, 10111.74) | 7838.36 (5733.84, 10163.46) | -0.49 (-0.63, -0.35) | -0.73 (-1.21, -0.25) |
| Tropical Latin America | 5744.78 (4146.62, 7543.43) | 5719.73 (4146.26, 7461.74) | 5889.37 (4255.92, 7701.51) | 0.02 (-0.03, 0.06) | -0.06 (-0.14, 0.02) |
| Western Europe | 2395.05 (1786.28, 3123.24) | 2386.75 (1779.99, 3115.39) | 2404.86 (1790.97, 3136.99) | 0.02 (0.00, 0.03) | -0.04 (-0.08, 0.00) |
| Western Sub-Saharan Africa | 7520.49 (5610.73, 9722.11) | 7524.26 (5616.51, 9763.05) | 7656.26 (5652.53, 9981.63) | -0.08 (-0.14, -0.02) | -0.10 (-0.55, 0.35) |
| **Genital herpes** |  |  |  |  |  |
| Global | 960.29 (822.81, 1116.81) | 991.27 (852.64, 1142.47) | 1021.68 (869.15, 1191.20) | 0.09 (0.05, 0.13) | 0.27 (0.07, 0.47) |
| Sex |  |  |  |  |  |
| Female | 1192.43 (1027.73, 1373.81) | 1238.59 (1072.00, 1419.65) | 1272.13 (1084.26, 1479.17) | 0.10 (0.06, 0.13) | 0.33 (0.12, 0.54) |
| Male | 733.92 (620.59, 864.86) | 749.35 (640.03, 875.01) | 778.33 (657.90, 917.03) | 0.09 (0.04, 0.14) | 0.16 (-0.03, 0.35) |
| SDI region |  |  |  |  |  |
| Low | 1381.15 (1192.61, 1585.82) | 1385.60 (1211.14, 1571.60) | 1332.24 (1139.14, 1537.39) | -0.19 (-0.26, -0.11) | 0.27 (0.04, 0.49) |
| Low-middle | 887.22 (756.91, 1034.59) | 938.71 (807.42, 1087.82) | 951.22 (810.95, 1108.43) | 0.20 (0.15, 0.24) | 0.59 (0.52, 0.65) |
| Middle | 970.48 (830.83, 1125.14) | 951.20 (815.23, 1102.17) | 983.59 (834.60, 1149.82) | 0.04 (0.00, 0.09) | -0.06 (-0.33, 0.20) |
| High-middle | 856.48 (728.85, 1001.48) | 855.83 (730.79, 993.95) | 871.68 (739.91, 1022.12) | -0.10 (-0.18, -0.02) | -0.08 (-0.49, 0.33) |
| High | 943.30 (808.92, 1097.75) | 902.05 (782.05, 1038.57) | 862.23 (729.35, 1013.89) | -0.27 (-0.33, -0.21) | -0.40 (-0.56, -0.25) |
| GBD region |  |  |  |  |  |
| Andean Latin America | 1744.10 (1537.00, 1976.47) | 1808.88 (1617.65, 2034.55) | 1741.01 (1491.84, 2008.59) | -0.12 (-0.18, -0.05) | 0.32 (0.16, 0.49) |
| Australasia | 978.95 (793.60, 1182.83) | 856.23 (742.03, 974.46) | 794.25 (666.11, 945.00) | -0.59 (-1.03, -0.15) | -1.17 (-1.77, -0.58) |
| Caribbean | 1629.47 (1388.57, 1889.43) | 1620.51 (1376.55, 1878.36) | 1581.10 (1347.15, 1842.73) | -0.11 (-0.12, -0.10) | -0.05 (-0.06, -0.05) |
| Central Asia | 689.13 (579.09, 816.46) | 692.41 (582.67, 822.21) | 689.14 (579.50, 820.98) | -0.01 (-0.01, 0.00) | 0.05 (0.04, 0.06) |
| Central Europe | 494.38 (418.21, 583.25) | 487.70 (412.95, 579.24) | 494.18 (415.42, 589.23) | 0.00 (-0.04, 0.03) | -0.16 (-0.29, -0.04) |
| Central Latin America | 1534.24 (1337.35, 1752.73) | 1415.31 (1219.73, 1624.93) | 1502.79 (1282.43, 1746.05) | 0.09 (-0.02, 0.20) | -0.78 (-0.87, -0.69) |
| Central Sub-Saharan Africa | 2252.02 (1977.50, 2538.88) | 2260.52 (1999.82, 2541.53) | 2245.43 (1973.68, 2530.90) | -0.01 (-0.02, 0.00) | 0.04 (0.03, 0.05) |
| East Asia | 761.34 (637.11, 905.63) | 754.28 (635.50, 890.60) | 761.71 (640.16, 907.48) | -0.43 (-0.65, -0.21) | -0.33 (-1.34, 0.68) |
| Eastern Europe | 935.17 (786.53, 1100.05) | 933.07 (784.87, 1098.77) | 936.47 (787.28, 1100.87) | 0.01 (0.00, 0.01) | -0.02 (-0.03, -0.01) |
| Eastern Sub-Saharan Africa | 2024.14 (1760.53, 2312.94) | 2211.21 (1958.75, 2490.58) | 1939.71 (1662.66, 2250.26) | -0.60 (-0.77, -0.43) | 0.78 (0.39, 1.16) |
| High-income Asia Pacific | 797.06 (717.29, 890.86) | 687.72 (578.75, 808.82) | 680.41 (571.07, 805.51) | -0.31 (-0.42, -0.19) | -1.36 (-1.80, -0.90) |
| High-income North America | 1227.60 (1037.68, 1443.43) | 1191.51 (1018.26, 1390.79) | 1077.77 (906.01, 1267.90) | -0.52 (-0.62, -0.42) | -0.30 (-0.35, -0.24) |
| North Africa and Middle East | 817.35 (694.65, 957.94) | 791.01 (678.65, 918.85) | 799.14 (675.26, 948.70) | -0.06 (-0.09, -0.03) | -0.33 (-0.38, -0.27) |
| Oceania | 1404.58 (1186.23, 1657.29) | 1514.66 (1369.53, 1676.40) | 1428.70 (1205.75, 1669.20) | -0.01 (-0.17, 0.14) | 0.74 (0.54, 0.94) |
| South Asia | 589.20 (494.54, 704.83) | 624.83 (526.79, 742.79) | 626.23 (527.02, 744.30) | 0.25 (0.19, 0.32) | 0.60 (0.39, 0.82) |
| Southeast Asia | 1103.72 (945.57, 1275.96) | 1108.52 (949.31, 1288.23) | 1088.42 (915.94, 1276.73) | -0.11 (-0.12, -0.09) | 0.02 (-0.08, 0.11) |
| Southern Latin America | 1241.94 (1135.52, 1359.36) | 1240.75 (1132.94, 1356.88) | 1216.03 (1031.26, 1419.28) | -0.10 (-0.12, -0.08) | -0.01 (-0.03, 0.01) |
| Southern Sub-Saharan Africa | 2166.56 (1884.67, 2475.94) | 2288.51 (2021.83, 2578.41) | 2275.42 (1990.26, 2605.72) | 0.25 (0.15, 0.35) | 0.59 (0.31, 0.87) |
| Tropical Latin America | 1930.74 (1651.19, 2229.14) | 1978.72 (1699.57, 2270.38) | 1911.52 (1637.13, 2214.89) | 0.05 (-0.09, 0.19) | 0.24 (0.17, 0.31) |
| Western Europe | 732.69 (621.91, 863.74) | 717.19 (637.90, 807.91) | 696.52 (586.28, 821.86) | -0.11 (-0.18, -0.04) | -0.16 (-0.31, -0.02) |
| Western Sub-Saharan Africa | 1631.96 (1399.92, 1890.87) | 1689.49 (1478.73, 1919.29) | 1639.92 (1404.48, 1895.52) | -0.08 (-0.12, -0.04) | 0.31 (0.18, 0.44) |

Note: EAPC, estimated annual percentage change; CIs, confidence intervals; UIs, uncertainty intervals


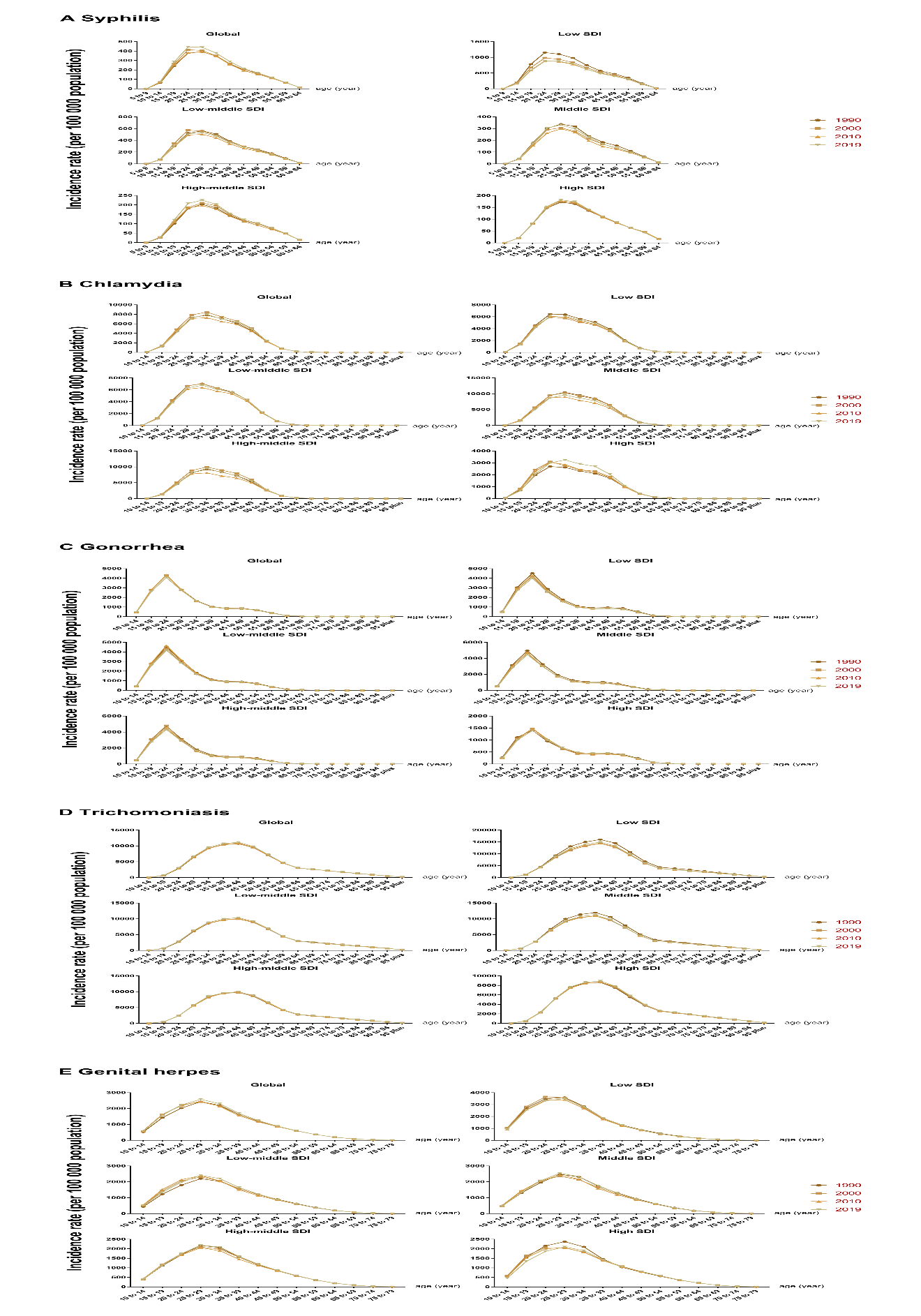


**Figure S3 Incidence rate of STIs by age group and SDI region in 1990, 2000, 2010 and 2019. A: syphilis, B: chlamydia, C: gonorrhea, D: trichomoniasis, E: genital herpes; SDI socio-demographic index**


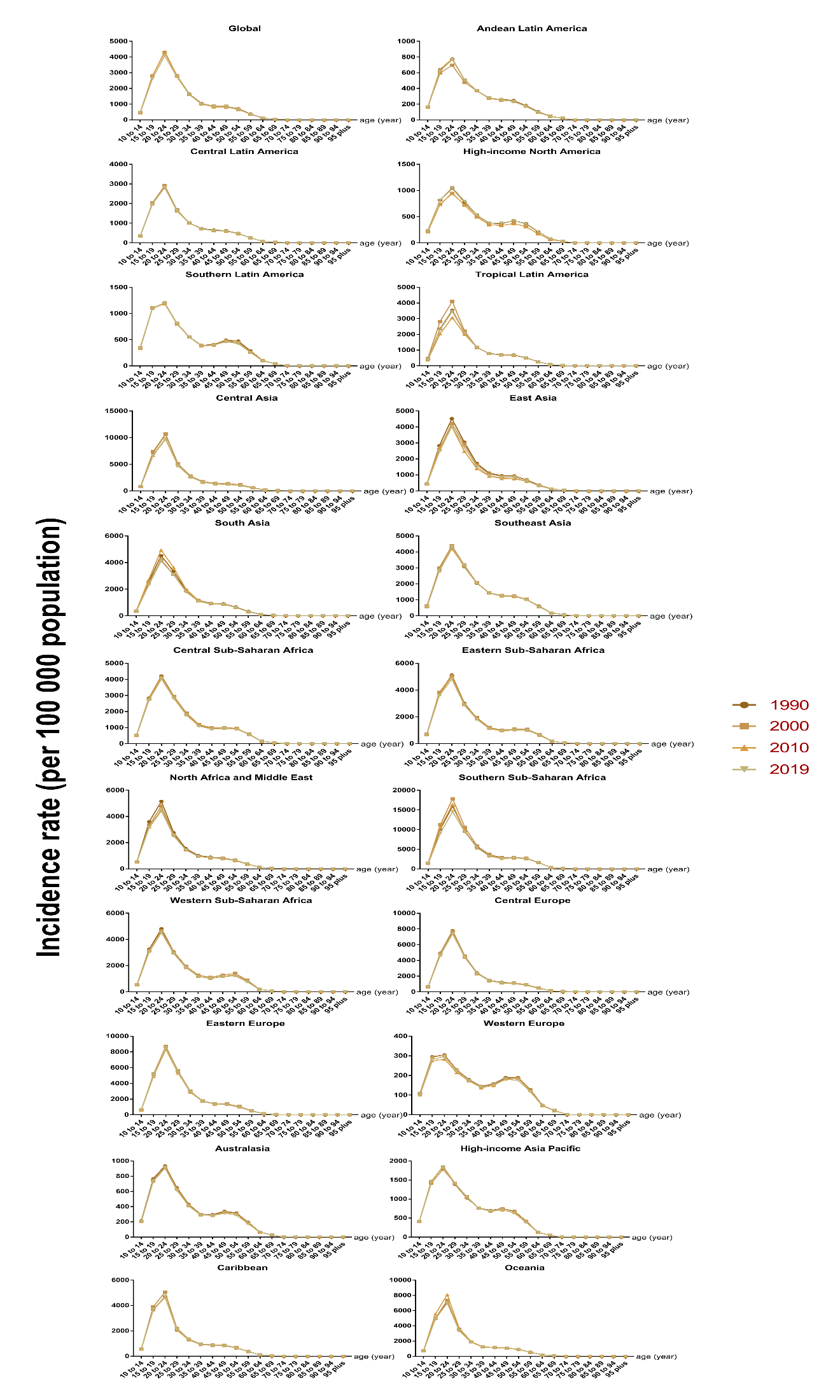


**Figure S4 Incidence rate of gonorrhoea by age and GBD region in 1990, 2000, 2010 and 2019. GBD: Global Burden of Disease Study**


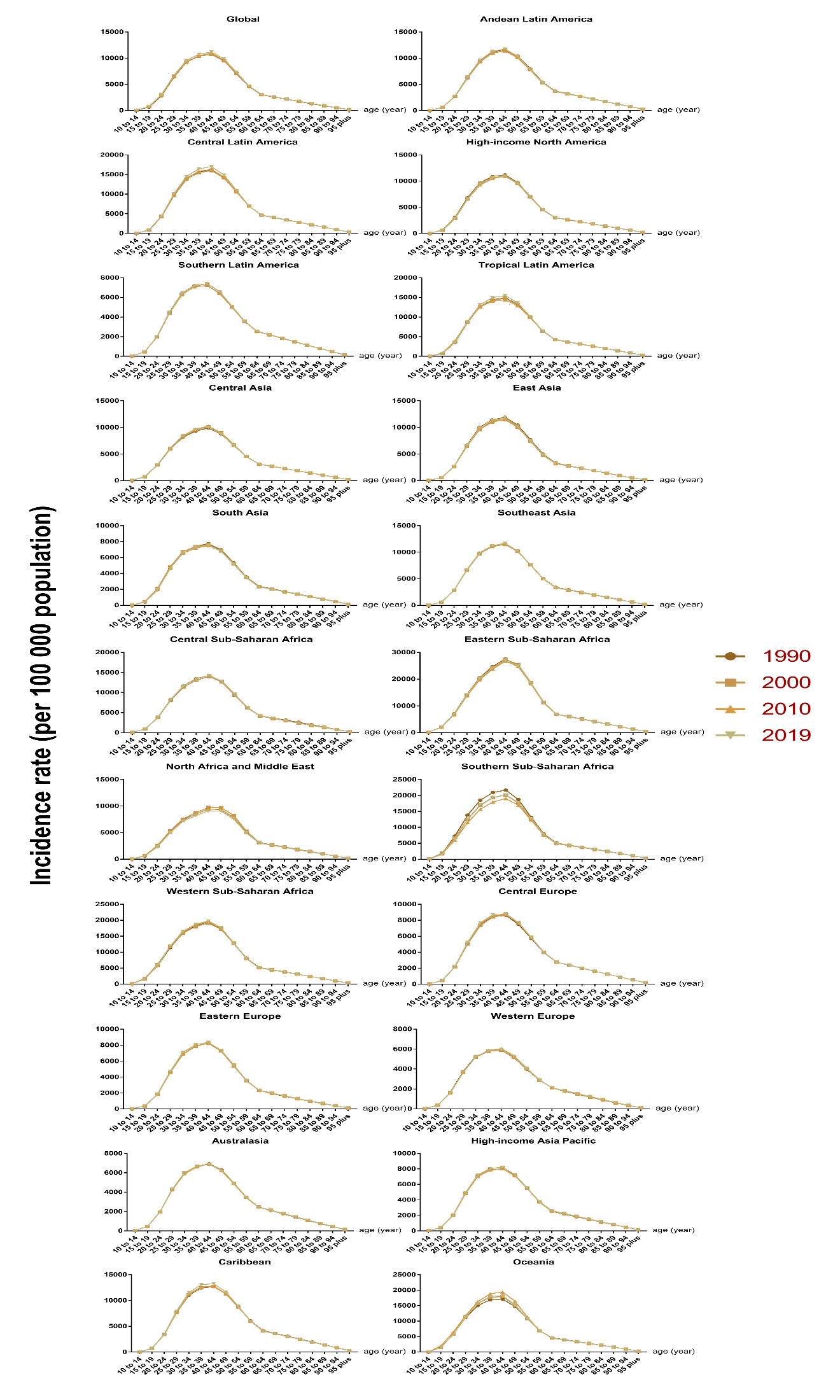


**Figure S5 Incidence rate of trichomoniasis by age and GBD region in 1990, 2000, 2010 and 2019. GBD: Global Burden of Disease Study**


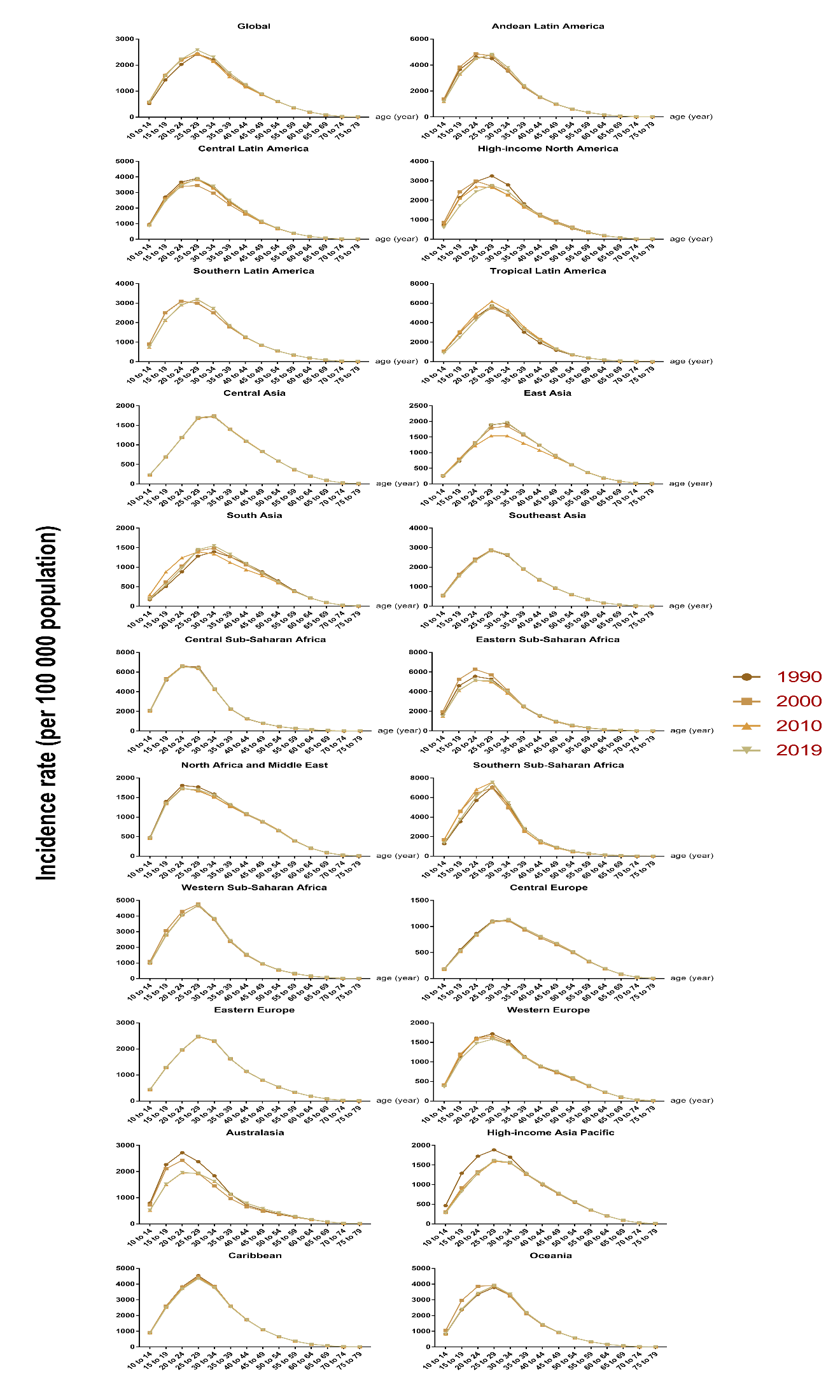


**Figure S6 Incidence rate of genital herpes by age and GBD region in 1990, 2000, 2010 and 2019. GBD: Global Burden of Disease Study**
